# Biocatalytic Heteroaromatic Amide Formation in Water Enabled by a Catalytic Tetrad and Two Access Tunnels

**DOI:** 10.1021/acscatal.4c01268

**Published:** 2024-05-25

**Authors:** Erna Zukic, Daniel Mokos, Melanie Weber, Niklas Stix, Klaus Ditrich, Valerio Ferrario, Henrik Müller, Christian Willrodt, Karl Gruber, Bastian Daniel, Wolfgang Kroutil

**Affiliations:** † Austrian Centre of Industrial Biotechnology Acib GmbH c/o University of Graz, Heinrichstrasse 28, 8010 Graz, Austria; ‡ Institute of Molecular Biosciences, University of Graz, Humboldtstraße 50, 8010 Graz, Austria; § Institute of Chemistry, University of Graz, NAWI Graz, Heinrichstraße 28, 8010 Graz, Austria; ∥ Group Research BASF SE, A030, Carl-Bosch-Strasse 38, 67056 Ludwigshafen am Rhein, Germany; ⊥ Field of Excellence BioHealth, University of Graz, 8010 Graz, Austria; # BioTechMed Graz, 8010 Graz, Austria

**Keywords:** amide bond formation, biocatalysis, lipase, heteroaromatic ester, preparative scale, SpL

## Abstract

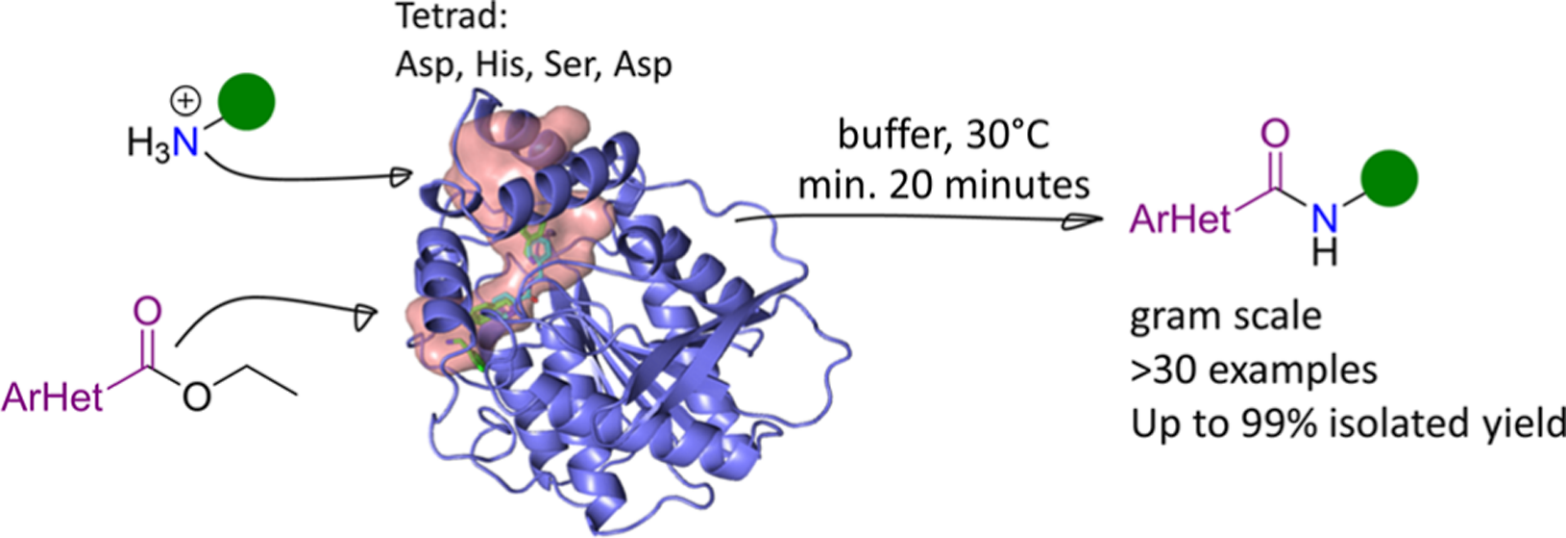

The amide moiety belongs to the most common motives in pharmaceutical chemistry, present in many prescribed small-molecule pharmaceuticals. Methods for its manufacture are still in high demand, especially using water/buffer as a solvent and avoiding stoichiometric amounts of activation reagents. Herein, we identified from a library of lipases/esterases/acyltransferases and variants thereof a lipase originating from *Sphingomonas* sp. HXN-200 (SpL) able to form amides in aqueous solution starting from a broad scope of sterically demanding heteroaromatic ethyl esters as well as aliphatic amines, reaching isolated yields up to 99% on preparative scale and space time yields of up to 864 g L^–1^ d^–1^; thus, in selected cases, the amide was formed within minutes. The enzyme features an aspartate next to the canonical serine of the catalytic triad, which was essential for amide formation. Furthermore, the enzyme structure revealed two tunnels to the active site, presumably one for the ester and one for the amine, which permit the bringing together of the sterically demanding heteroaromatic esters and the amine in the active site. This work shows that biocatalytic amide formation starting from various five- and six-membered heteroaromatic ethyl esters in the buffer can serve as a platform for preparative amide synthesis.

## Introduction

**Scheme 1:**
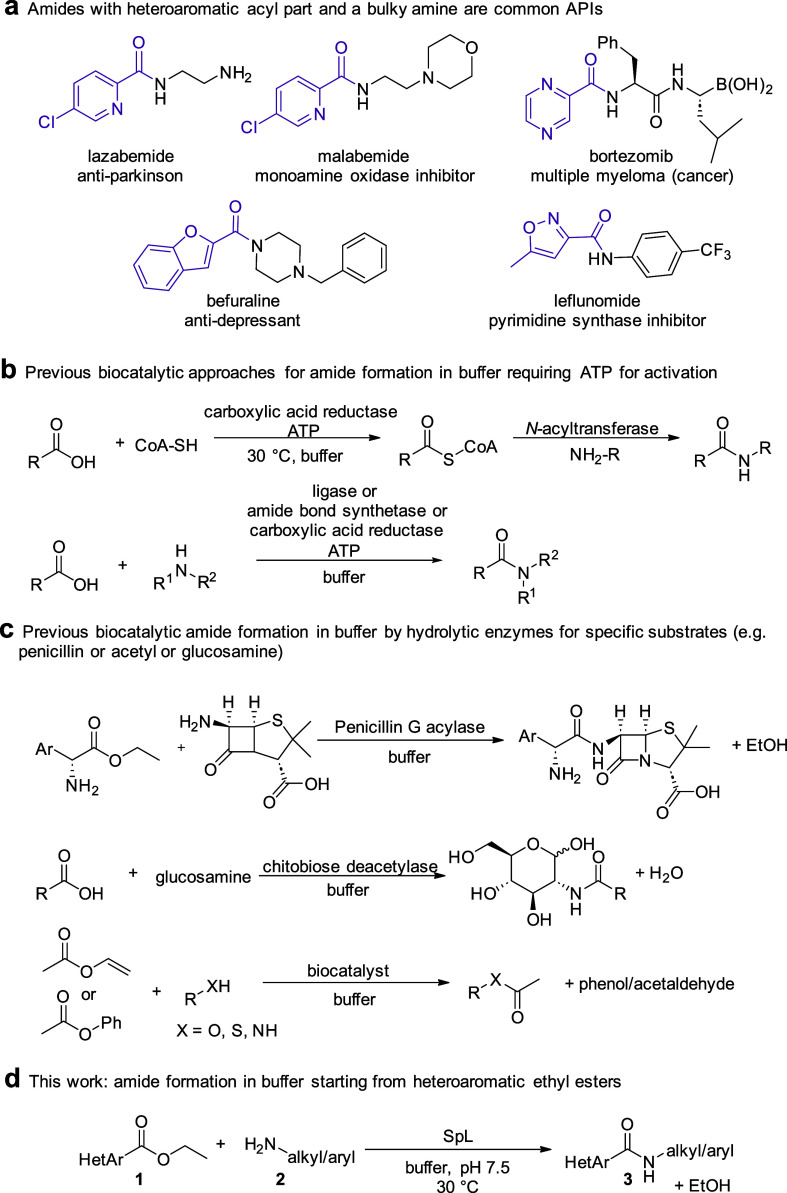
Examples of APIs with Amide Functionality and Biocatalytic Reactions Leading to Amides in an Aqueous Buffer^,^^,^^,^ The amide functionality as a common motif in pharmaceuticals. Biocatalytic amide formation requiring ATP for substrate activation and consequently additional enzymes and reagents for recycling are needed. Hydrolytic enzymes for the acylation of (+)-6-aminopenicillanic, glucosamine, and acetylation of selected amines. This work: amide formation in buffer using heteroaromatic ethyl esters and amine.

Although various methods for amide formation have been described,^1−5^ amide synthesis has been voted as a top challenge to be addressed to reduce the number/amount of reagents and the waste which results from, e.g., stoichiometric equivalents of activation reagents needed.^6−8^ Considering selected APIs with amide functionality (Scheme 1a), a sterically demanding heteroaromatic group is attached to the carbonyl of the amide functionality, including, for example, pyridine, as in lazabemide^9^ or malabemide,^10,11^ pyrazine in bortezomib,^10^ benzofurane (befuraline),^12,13^ or isoxazole (leflunomide).^12^

One option to address the challenge of amide formation may be the use of enzymes.^1,2,14−18^ Amides have been recently prepared using biocatalysis in aqueous buffer, for example, starting from the carboxylic acids and using ATP as activation agent with enzymes such as ligases, amide bond synthetases, and carboxylic acid reductases (Scheme 1b).^19−24^ Since these enzymes require ATP that needs to be recycled on preparative scales, additional recycling enzymes or using living organisms increase the complexity of the reaction.

When using hydrolases such as lipases for amide synthesis, the reactions are generally performed in an organic solvent to circumvent hydrolysis of the ester substrate or amide product,^18^ or in an organic solvent in the presence of small amounts of water to hydrate the cells/enzyme.^25^ Interestingly, most lipases/esterases display a limited substrate scope in the sense that they do not transform aromatic/bulky acyl donors but prefer small acyl groups.^16,26^ Nevertheless, a few hydrolases have been reported to deal with more bulky esters in hydrolysis.^27−29^ Amide formations in buffer by hydrolytic enzymes are exceptions and have only been reported for certain substrates like (+)-6-aminopenicillanic acid,^16^ capuramycin analogues^30^ or glucosamine^31^ sometimes showing low conversion (e.g., max 20%)^29^ or being restricted to the transfer of the small acetyl group as noted for the acyltransferase from *Pseudomonas protegens*(32) and *Mycobacterium smegmatis* and its variants (Scheme 1c).^33,34^ The reaction in water would be highly desired as reactions in an aqueous environment may reduce the carbon footprint, minimize waste, and increase safety. Furthermore, biocatalytic reactions in buffer are easier to combine with subsequent reactions catalyzed by enzymes in one pot in a cascade fashion.^35^ Therefore, our aim was to find enzymes accepting aromatic esters as acyl donors and bulky amines for the formation in buffer with the challenge that the enzyme has to prefer the amine as a nucleophile over the water present at high concentration (Scheme 1d). An additional challenge might be that the amine is present in the buffer preferably as the corresponding protonated species at pH 7–9 and therefore would not readily react as a nucleophile.

## Results and Discussion

**Scheme 2:**
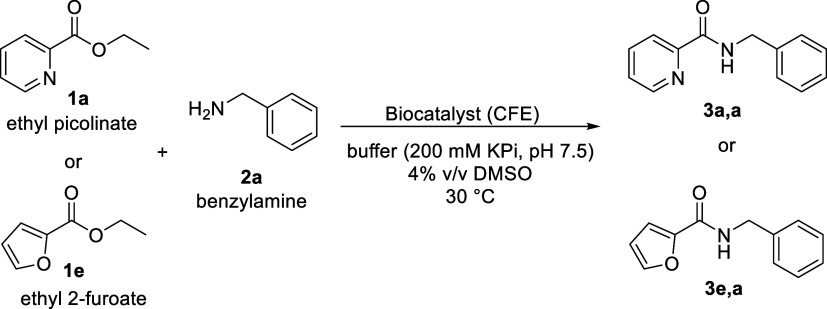
Amidation Reactions Used for Screening and Evaluation of the Ratio of Ester to Amine

In pursuit of amide formation activity in buffer using heteroaromatic esters as substrates, we investigated a library of 35 enzymes (wild type and variants) selected from literature either based on reports of transesterification in buffer or transformation of bulky acyl groups or due to their reactivity toward amides (Table S1). The library included the acyltransferase from *M. smegmatis* (MsAcT)^33^ and variants thereof^36^ as well as the variant F148 V from the acyltransferase from *P. protegens*,^37^ lipases from *Candida antarctica* (and variants thereof),^28^*Rhizomucor miehei* (RML),^38^*Sphingomonas* HXN-200 (SpL),^25^ and the lipase EstCE1 and variants thereof,^29^ the esterases from *Alcanivorax borkumensis* SK2,^27^*Pseudomonas aestusnigri* VGXO14,^27^*Pyrobaculum calidifontis* (PestE), and *Alyciclobacillus acidocaldarius* (Est2),^39^ the malonamidase (MEA2) from *Bradyrhizobium japonicum*, the amidase (AMI) from *Rhodococcus globerulus,* and the cyclic imide hydrolase (CIH) from *Pseudomonas putida*.^40^ The biocatalysts were overexpressed in *Escherichia coli* and tested using cell free-extracts (CFEs) as enzyme preparations with ethyl picolinate **1a** and ethyl 2-furoate **1e** (20 mM) and benzylamine **2a** (20 mM) in buffer at 30 °C (Scheme 2).

**Figure 1:**
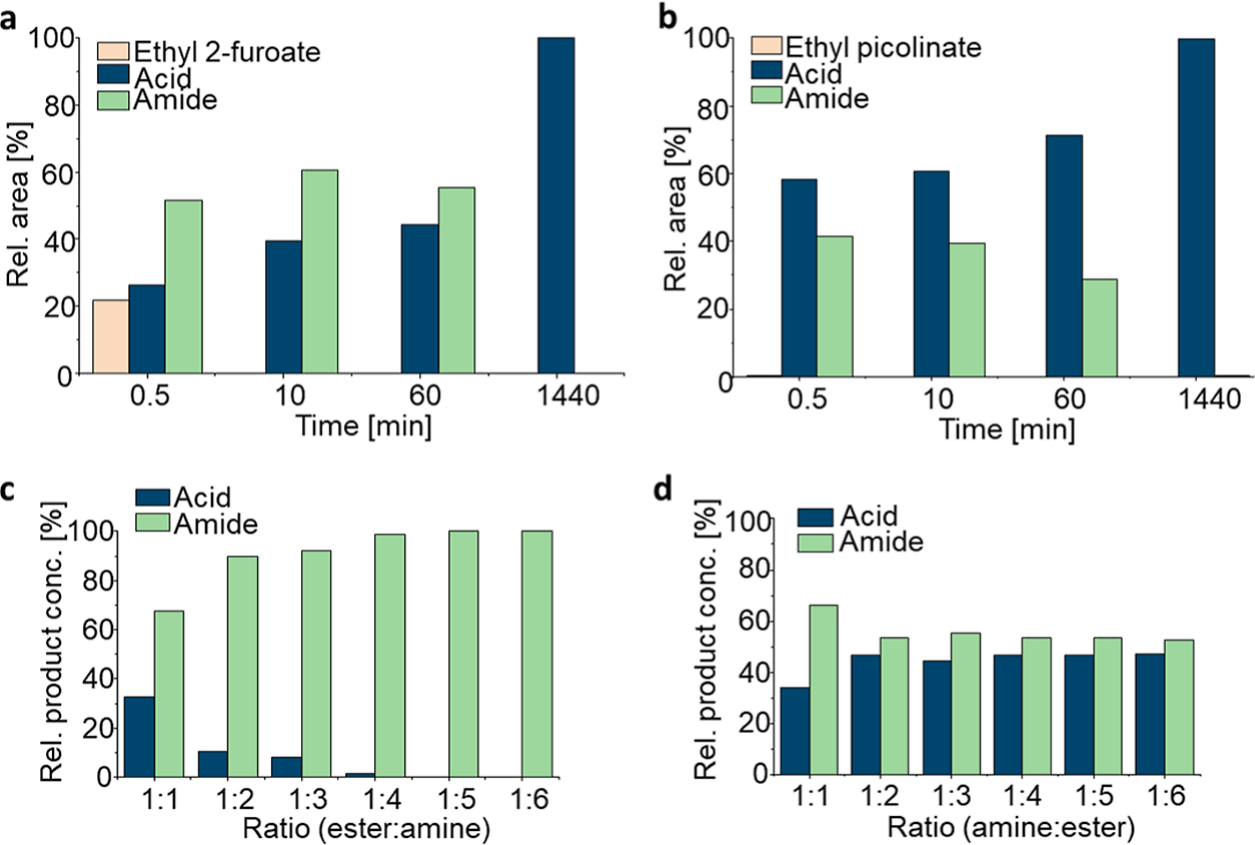
Amide formation after selected time points and influence of ester to amine ratio on amide formation catalyzed by SpL in buffer. (a, b) Amide and acid formation within 60 min and after 24 h for (a) ethyl 2-furoate **1e** and (b) ethyl picolinate **1a** with benzylamine **2a**. Reaction conditions: amine (20 mM), ester (20 mM), CFE (10 mg/mL), 4% DMSO in 200 mM K-phosphate buffer (pH 7.5) at 30 °C. HPLC-area at 280 nm. (c, d) Influence of ester/amine ratio on amide formation (c) varying benzylamine **2a** conc. and keeping ester conc. constant (10–60 mM, ester conc. at 10 mM ethyl picolinate) and (d) varying ester conc. at constant amine concentration (10–60 mM ethyl picolinate **1a**, benzylamine **2a** concentration at 10 mM). Further reaction conditions: CFE 0.01 mg/mL, total volume 250 μL, 200 mM KPi buffer, pH 7.5, 30 °C, 30 min.

From all catalysts tested, MsAcT, PestE, Est2, and SpL turned out to be interesting candidates as clear amide formation was observed in buffer after 1 h. All other enzymes did not show amide formation at all, very little, or only hydrolysis of the ester or no activity (Table S13). MsAcT and variants displayed activity with ethyl 2-furoate **1e** but did not accept ethyl picolinate **1a** as an ester substrate (data not shown). In a preliminary analytical comparison of the substrate scope of the lipases PestE, Est2, and SpL, SpL was superior in terms of substrate scope and estimated amide formation (Table S14). Consequently, we focused on SpL in further studies. The lipase from *Sphingomonas* sp. HXN-200 (SpL) has previously been described to produce amides starting from esters and acids in an organic solvent, namely, *n*-hexane containing water (up to 4% for the pure enzyme and 16% for the whole cells).^25^ Testing ethyl picolinate **1a** or ethyl 2-furoate **1e** as an ester substrate (20 mM) with benzylamine (20 mM) in phosphate buffer (pH 7.5 at 30 °C) resulted in amide formation (Figure 1a,b) for both substrates within minutes (60% amide formation for ethyl 2-furoate **1e** after 10 min, Figure 1a). Using an ester without a heteroatom as the substrate, like ethyl benzoate, amide formation was also observed, but was less pronounced, probably because the product amide did precipitate.

In the case of ethyl 2-furoate **1e** clearly, amide formation was preferred over hydrolysis of the ester. Although the amine concentration (20 mM) is much lower than the water concentration (55,000 mM), the catalyst seems to control which nucleophile, water or amine, is attacking preferentially. Running the reaction for 24 h led to the carboxylic acid as the main product for both substrates. This indicated that the amide can also act as a substrate for SpL leading to the thermodynamic products under these conditions. The ratio of ester to amine was varied to obtain highest yield of amide possible while using as little excess of amine as possible. Employing various ester/amine ratios (1:1, 1:2, 1:3, 1:4, 1:5, and 1:6 for ethyl picolinate/benzylamine), amide formation increased with increasing amine concentration (Figure 1c). An ester/amine ratio of 1:3 to 1:4 was minimally required for this substrate pair to minimize acid formation under these conditions. For comparison, when the amine concentration was kept at 10 mM and the ester concentration was varied (Figure 1d), it was observed that with increasing ester concentration, more amide as well as more acid was formed. The results indicate that it is more beneficial for the reactions to have the amine in excess than the ester. An amine concentration above 50 mM at 10 mM ester concentration led to slightly slower amide formation. Testing amide formation of ethyl picolinate **1a** and ethyl 2-furoate **1e** with benzylamine **2a** in the range of pH 4.0–11.0, it was found that for both esters, hydrolysis is dominant under more acidic conditions, while amide formation is preferred at more basic conditions reaching its maximum at pH 9.5 with CHES as the buffer (Figure S13). Nevertheless, pH 7.5 potassium phosphate buffer (KPi) was chosen for further experiments to minimize the molecules and buffer salts with amine functionalities in the reaction mixture.

The activity of the lyophilized CFE enzyme preparation was measured under improved reaction conditions for amide formation with the **1a**/benzylamine **2a** pair (1:4) to be 4.3 U mg^–1^. The same substrate pair was also used to measure the activity of the purified enzyme, which was 26.4 U mg^–1^. The comparison of amide formation versus hydrolysis showed that for **1a**/**2a** amide formation is 5.6 times faster than ester hydrolysis under the conditions employed (Figure S14). For general activity measurements of SpL, the hydrolysis of *para*-nitrophenyl butyrate (*p*NPB) was followed giving an activity of 708 U mg^–1^ for the pure enzyme and 84 U mg^–1^ for the cell-free extract.

With the improved conditions concerning pH, buffer, and substrate ratio, a library of heteroaromatic ethyl esters was investigated first on an analytical scale. In addition to benzylamine **2a**, aniline **2b**, *n*-hexylamine **2c**, allylamine **2d**, methylamine **2e**, and piperidine **2f** were also tested as an amine substrate. Methylamine **2e** and sterically demanding secondary amine piperidine **2f** were not transformed under the conditions employed. In contrast, benzylamine **2a**, aniline **2b**, and *n*-hexylamine **2c** were very well accepted, and amide formation was also observed for allylamine **2d** in selected cases.

**Figure 2:**
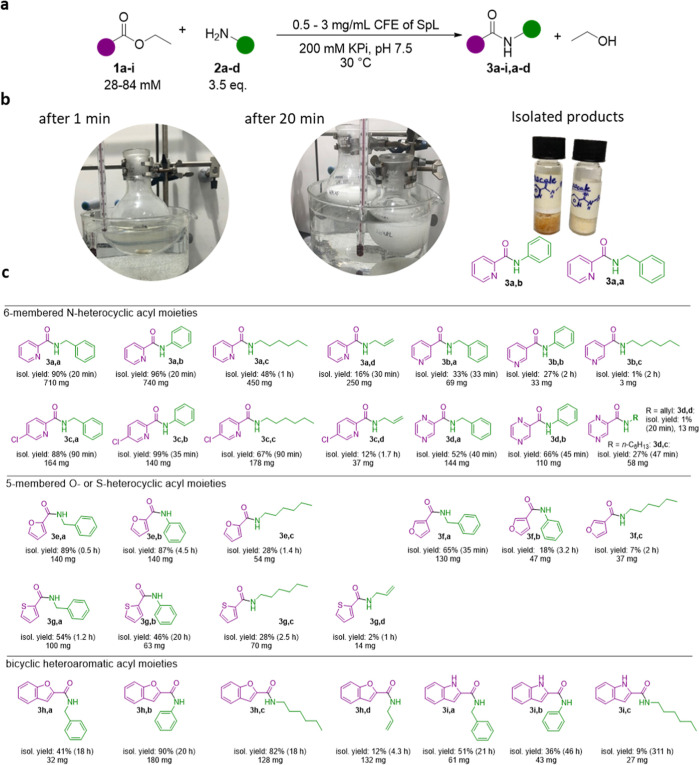
Preparative reaction conditions, precipitation of product amide and substrate scope. (a) Reaction scheme of preparative transformation coupling heteroaromatic ethyl esters **1** with amines **2** to yield amide **3**. (b) During product formation, here shown for *N*-phenylpicolinamide **3a,b** and *N*-benzylpicolinamide **3a,a**, precipitation was observed within the first minutes. For the examples shown, the reaction was finished within 20 min (65 or 62 mM ester concentration, respectively). (c) Substrate scope of biocatalytic amide formation starting from heteroaromatic esters and amines (benzylamine, aniline, *n*-hexylamine, and allylamine). Various types of heteroaromatic ethyl esters were employed, including six-membered N-heterocycles, five-membered heterocycles, as well as bicyclic ones. The isolated yields refer to preparative transformations after the time indicated in brackets.

Encouraged by the successful tests on an analytical scale, preparative transformations were performed (Figure 2) for the successful ester/amine pairs whereby the substrate concentration was increased, and depending on the reaction, the concentration of the catalyst was adapted or the reaction time was extended (see the Supporting Information). The ester concentration was varied between 28 and 84 mM employing 3.5 eq. of amine. While in the analytical experiments DMSO was used as the cosubstrate, DMSO was not used in any of the preparative transformations, all substrates were added directly to the mixture. For 18 out of 32, the product was isolated by simple extraction which was sufficient to obtain pure product for NMR. Column chromatography was only performed if there was unreacted ester remaining. For instance, ethyl picolinate **1a** at a concentration of 65 mM reacted with benzylamine **2a** (3.5 equiv) to give the corresponding amide **3a,a** on preparative scale (60 mL) within 20 min yielding 710 mg of the desired pure amide after a simple extraction which equals 90% isolated yield. This corresponds to an outstanding space time yield of 36 g of L^–1^ h^–1^ or 864 g of L^–1^ d^–1^. The precipitation of the formed amide in water turned the initially clear reaction solution turbid; product precipitation may very likely be the driving force of the reactions. For instance, amide formation with ethyl picolinate and benzylamine (and others) was already observed after the first minute as the solution turned turbid (Figure 2b, first picture) and subsequently a white precipitate was formed (second picture). Ethyl picolinate **1a** was successfully coupled also with aniline **2b**, *n*-hexylamine **2c**, and allylamine **2d** (Figure 2c).

Additionally, the *p*-substituted chloro-picolinate ethyl ester **1c** worked equally well. In the case of ethyl nicotinate **1b**, the conversions and yields were found to be reduced using the general reaction conditions employed. Nevertheless, when the heteroaromatic ring contained two nitrogen atoms, as in pyrazinoic acid ester **1d**, isolated yields of up to 66% were achieved. The approach worked also for five-membered heterocycles containing an O- or S-heteroatom as well as for bicyclic heteroaromatic systems with an O- or N-heteroatom reaching up to 90% isolated yield using standard conditions.

**Figure 3:**
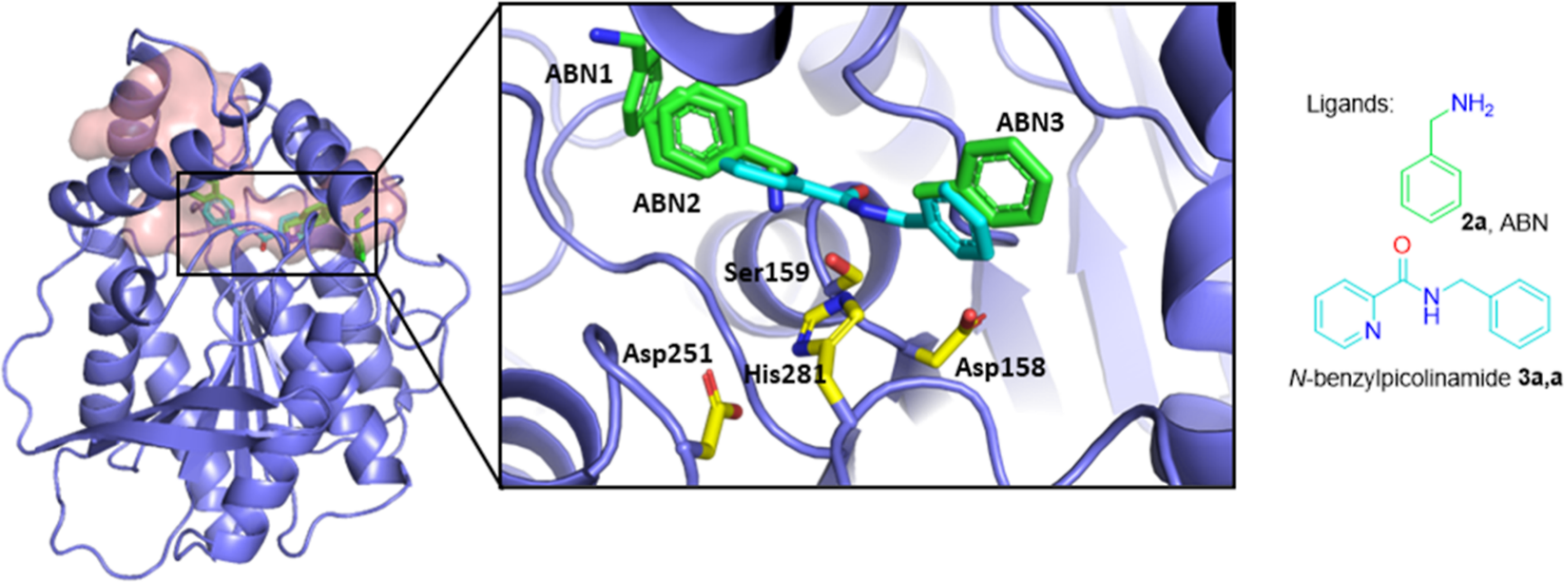
X-ray crystal structure of SpL with active site and channels soaked with benzylamine (green) and the amide product (cyan) overlaid, the active site enlarged, and the catalytic residues are shown. For polder omit maps of the bound ligands, see Figure S8.

To gain insights into the mechanism of amide formation catalyzed by lipase SpL, its structure was determined by X-ray crystallography (Figure 3). Diffraction data were collected under cryo-conditions from native crystals, as well as from crystals soaked with benzylamine **2a** or the amide *N*-benzyl-2-pyridinecarboxamide **3a,a** leading to data sets with resolutions of 2.0, 1.6, and 1.8 Å, respectively (Table S10). SpL is a hormone-sensitive lipase-like (HSL-like) protein adopting the alpha-beta hydrolase fold, with a helical lid domain over the active site cavity with the catalytic triad.^41^ The common Ser-His-Asp catalytic triad was located at positions Ser159, His281, and Asp251 (Figure 3). In the complex with the amide product, the carbonyl carbon atom of the ligand was found in close proximity of 2.1 Å to the Ser159 of the catalytic triad, while the carbonyl O is oriented toward the oxyanion hole (NH of Gly92, Gly93, and Ala160).

**Figure 4:**
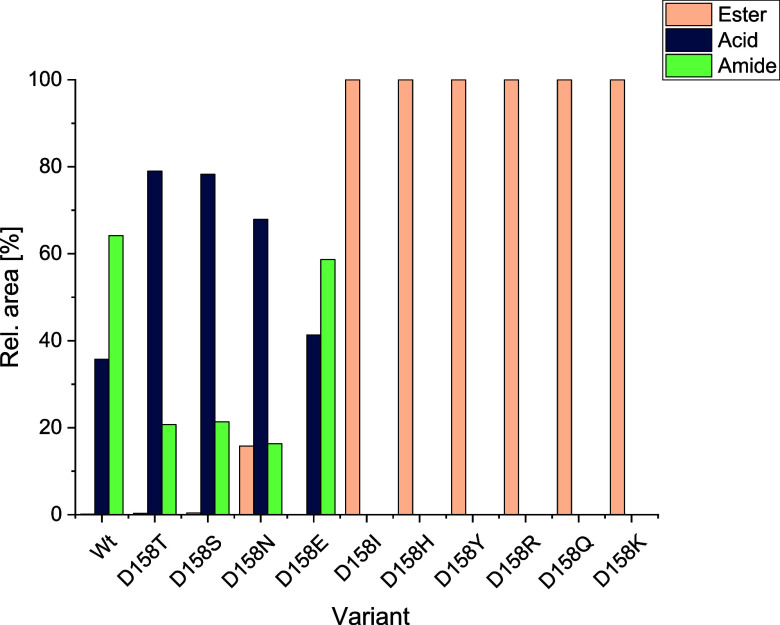
Comparison of the SpL-D158 variants regarding amide and acid formation. Reaction conditions: lyophilized CFE (2 mg/mL), ethyl picolinate (10 mM), and benzylamine (40 mM) in 200 mM KPi buffer (pH 7.5), 30 °C, 10 min. Analysis was by HPLC 280 nm.

Next to Ser159 of the catalytically active triad, an aspartate (Asp158) caught our attention as it is not present, e.g., in the lipase from *C. antarctica* B (CalB). Exchanging Asp158 to isoleucine (D158I) led to a variant that lost its ability to catalyze amide formation, although it was still expressed at a comparable level as the wild type (tested for ethyl picolinate with benzylamine at a ratio of 1:4,10 min) (Figure 4).

Interestingly, the D158I variant was still hydrolytically active, as ester **1a** was hydrolyzed to the corresponding acid with 84% conversion within 24 h as tested in a separate experiment in the absence of amine. This result indicated that the aspartate in position 158 may be essential for efficient amide formation. Subsequently, variants of D158 (SpL-D158 K/H/S/N/E/Q/T/R/Y) were generated and analyzed; the variants were expressible well in soluble form (Figure S2). The amide formation achieved with the Asp158Glu (D158E) variant was comparable to the wild type, although acid formation was more pronounced in this case under the conditions used. Reduced amide formation, but dominant acid formation was observed for asparagine (D158N) and amino acids with an aliphatic alcohol moiety (D158T and D158S). For all other variants, neither hydrolysis nor amide formation was observed within 10 min. Thus, a carboxylic acid moiety next to the serine at the binding site of the amine in the structure seems highly beneficial for amide formation.

**Scheme 3:**
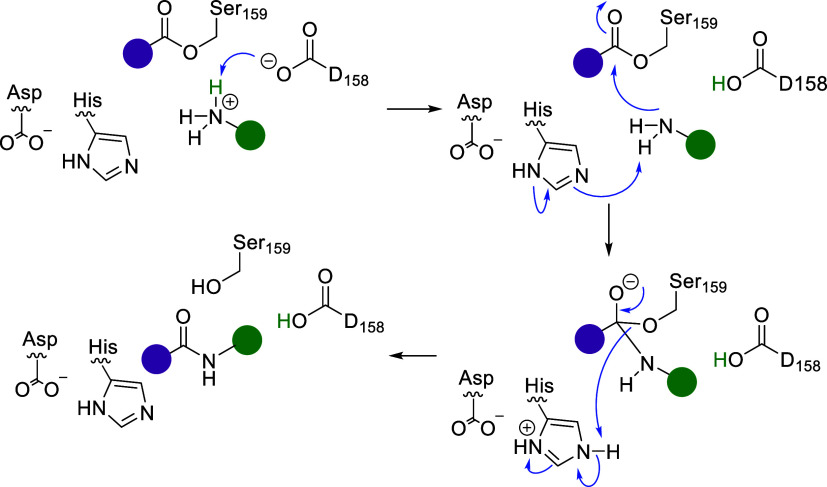
Proposal for a Mechanism Explaining the Role of Residue Asp158 for Amide Formation to Deprotonate the Aminium Species of the Substrate to Provide the Amine as a Nucleophile

Some other hydrolases (e.g., PestE, Est2, CE07, and CE03), which have been investigated in the initial aminolysis study in buffer, also possess an Asp next to the catalytically active Ser. To investigate the impact of the Asp also in the hydrolases which were of interest for amide formation (PestE and Est2), the aspartic acid next of the catalytic active site in PestE and Est2 was exchanged to isoleucine (Est2-D155I and PestE-D156I) (Figure S15). Also, for these enzymes, the exchange of the aspartate led to almost complete loss of activity, confirming that the aspartic acid residue next to the canonical serine plays a crucial role, enabling amide formation also for these hydrolases. As Asp158 in SpL seemed to be of high relevance for efficient amide formation, and putatively the negative charge of Asp158 is important to set the microscopic electrostatic environment at this position in favor of amide formation, we hypothesize that its carboxylate moiety may support deprotonation of the positively charged protonated amine substrate in buffer, which cannot act as a nucleophile (Scheme 3).

**Figure 5:**
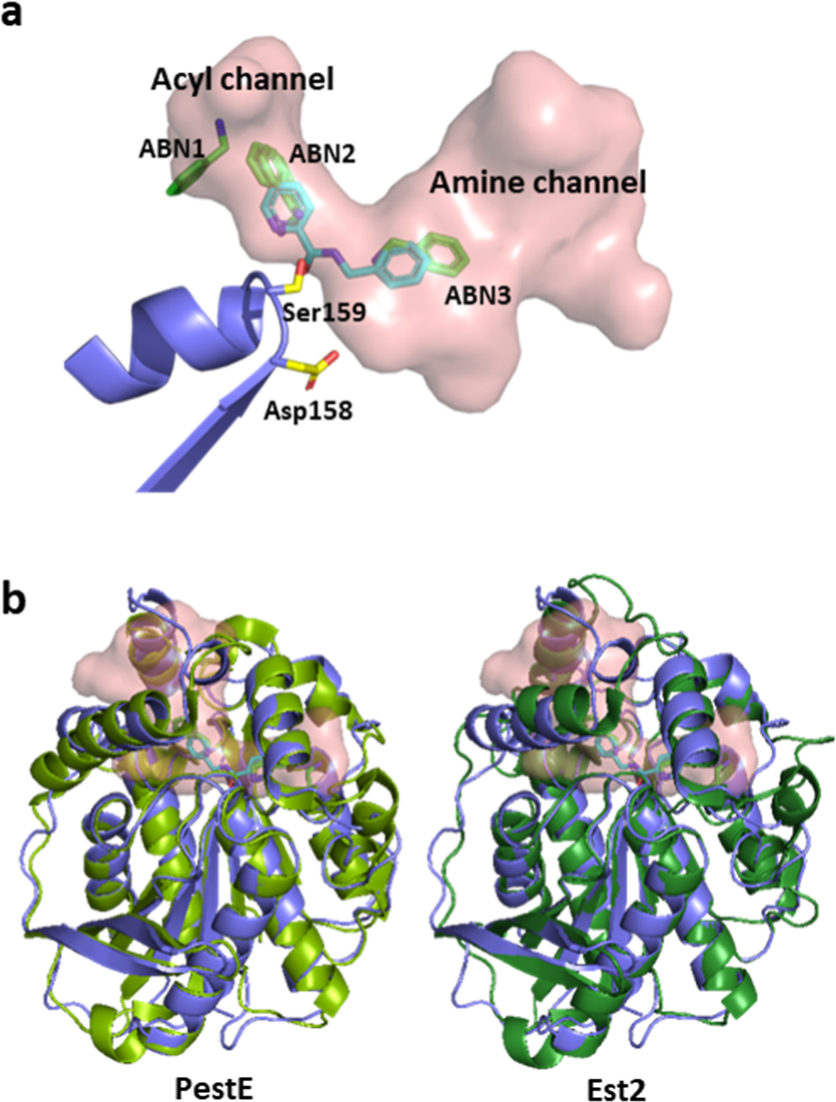
Channels in SpL, PestE, and Est2. (a) SpL has two funnel-shaped channels (acyl channel and amine channel) which allow to couple sterically demanding substrates since the ester enters from one side and the amine nucleophile from the other side. The positions of three benzylamine molecules are labeled with ABN1–3 and the molecules are colored in green. For an interactive 3D representation of the model, follow: https://mokosdaniel.github.io/SPL_modelviewer/. (b) Overlay of the structures of PestE (PDB: 2YH2) and Est2 (PDB: 1EVQ) with SPL (color slate), the ligands and the cavity. PestE (splitpea) and Est2 (forest) adopt a very similar fold to SPL, with the lid domain covering the active site cavity. The RMSD of their alignments to SPL are PestE: 0.610; Est2: 0.761.

By ensuring deprotonation of the aminium species, the amine becomes available as a nucleophile, which can then attack the acyl-enzyme intermediate following the classical lipase mechanism. The aspartate next to the catalytically active serine may represent an extension of the classical triad Ser-His-Asp/Glu^42−44^ in the lipase mechanism to a tetrad. Furthermore, two funnel-shaped channels leading from the surface to the catalytic triad or actually the tetrad were identified (Figures 3 and 5a). The two channels may be referred to as the acyl channel and the amine channel, based on the orientation of the catalytic residues and ligands, as well as the proposed mechanism. The acyl moiety of the amide product is located in the acyl channel, while the amine part is harbored in the amine channel.

In the complex with benzylamine, three benzylamine molecules (ABN1–3) could be placed into the electron density in the active site (Figure 5a). ABN3 was located in the nucleophile channel and adopts a position from which a nucleophilic attack on an assumed enzyme-acyl intermediate can be facilitated and is therefore considered at the catalytically relevant nucleophile position. Additionally, the angle between the ABN-NH_2_, the carbonyl C and the carbonyl O of the product is 101°, which is in line with the Bürgi-Dunitz angle of 107°, representing the ideal angle for a nucleophilic attack.^45^ The two channels seem to be the structural reason that the enzyme is able to couple two sterically demanding substrates by enabling the ester to enter the active site via the acyl channel, while the amine nucleophile approaches from the other side without interfering or the possibility of disturbing each other.

Since PestE and Est2 also showed activity for amide formation with sterically demanding heteroaromatic esters, the structure of SpL was compared to previously published structures of PestE^46^ (PDB: 3ZWQ) and Est2^47^ (PDB: 1EVQ). The structures show high similarity to SpL in the fold they adopt (Figure 5b). The catalytic residues also have the same positions in the aligned structures (Figure S16). Furthermore, PestE and Est2 also have a dual-access cavity with two funnel-shaped tunnels leading to the active site, similarly to SpL (Figures 5b and S17). Therefore, the active lipases identified in the initial screening fit the picture, that two access channels to the active site and an additional aspartate enable amide formation coupling heteroaromatic esters with amines in an aqueous buffered environment.

## Conclusions

In summary, we report the biocatalytic amide formation from heteroaromatic ethyl esters and amines in a buffer using a lipase from *Sphingomonas* HXN-200 (SpL). The enzyme possesses a broad substrate scope, enabling the efficient preparation of amides within short time (minutes to few hours) as shown on gram scale, reaching space time yields of up to 36 g L^–1^ h^–1^. Mutational and structural studies revealed that an aspartate next to the catalytically active serine is highly beneficial for the observed activity, which has not been described before, showing that a catalytic tetrad is actually required (Asp-Ser-His-Asp) for SpL for efficient amide formation in aqueous buffer. The additional aspartate may have the function to facilitate deprotonation of the in general protonated amine species in buffer at ambient pH.

Furthermore, the crystal structure showed that the enzyme possesses two access tunnels to the active site, probably one for the ester and one for the amine, another feature not described before. This feature allows for bringing together two sterically demanding substrates, the ester and amine, without interfering with each other. The possibility to efficiently make amides in buffer from sterically demanding esters and amines in the presence of water as competing nucleophile expands the repertoire of serine hydrolases for more sustainable synthesis of sterically demanding amides needed for pharmaceuticals and agrochemicals.

## Supporting Information Available

Materials; general, kits and instruments; cloning and site-directed mutagenesis; expression of His_6_-tagged hydrolases; purification of His_6_-tagged SpL; purification of His_6_-tagged Est2-D155I and PestE-D156I; SDS-PAGE analysis; crystallization of SpL and data collection; HPLC analysis; kinetic studies; pH screening of SpL Wt; biotransformation screening; PestE and Est2 structural comparison with SpL; synthesis of the substrate; NMR spectra of products; buffer for protein purification; medium preparation; used variants; and sequences (PDF)

## Notes

The code used for the 3D visualization of the SpL model is available at https://github.com/MokosDaniel/SPL_modelviewer/blob/main/index.html.
