# Supplementary material for: Biocatalytic Heteroaromatic Amide Formation in Water Enabled by a Catalytic Tetrad and Two Access Tunnels
Source: ACS Catal. 2024 May 25;14(11):8913–21. doi: 10.1021/acscatal.4c01268 (PMC11165448; doi:10.1021/acscatal.4c01268)
Supplement: Supplementary file 1 — cs4c01268_si_001.pdf [file cs4c01268_si_001.pdf]

Supporting Information

Biocatalytic Heteroaromatic Amide Formation in Water Enabled by a Catalytic Tetrad and Two Access Tunnels

Erna Zukic[a], Daniel Mokos[b], Melanie Weber[c], Niklas Stix[c], Klaus Ditrich[d], Valerio Ferrario[d], Henrik Müller[d], Christian Willrodt[d], Karl Gruber[b,e,f], Bastian Daniel[b,f]*, Wolfgang Kroutil[b,e,f]*

[a] Austrian Centre of Industrial Biotechnology Acib GmbH c/o University of Graz , Heinrichstrasse 28, 8010 Graz, Austria.

[b] Institute of Molecular Biosciences, University of Graz, Humboldtstraße 50, 8010 Graz, Austria.

[c] Institute of Chemistry, University of Graz, NAWI Graz, Heinrichstraße 28, 8010 Graz, Austria.

[d] Group Research BASF SE, A030, Carl-Bosch-Strasse 38, 67056 Ludwigshafen am Rhein, Germany.

[e] Field of Excellence BioHealth, University of Graz, 8010 Graz, Austria.

[f] BioTechMed Graz, 8010 Graz, Austria.

Email: Bastian.Daniel@uni-graz.at; Wolfgang.Kroutil@uni-graz.at.

Table of contents

Materials 3
General, Kits, and instruments 4
Cloning and site directed mutagenesis 4
Expression of His_6-tagged hydrolases 7
Purification of His_6-tagged SpL 8
Purification of His_6-tagged Est2-D155I & PestE-D156I 8
SDS-PAGE Analysis 9
Crystallization of SpL and Data Collection 11
HPLC analysis 14
Kinetic studies 14
Inhibition by benzylamine 17
pH screening of SpL Wt 18
Biotransformation screening 20
Biotransformations – testing PestE-D156I & Est2-D155I variants 23
PestE & Est2 – Structural comparison with SpL 23
Synthesis of substrate 24
Biotransformations – Preparative syntheses of amides 25
NMR of products 41
Buffer for protein purification 73
Medium preparation 73
Used variants 74
Sequences 74

Materials

SDS-PAGE loading buffer, SDS-PAGE gels and SDS-PAGE ladders were supplied by BioRad or Thermo Fisher. Solvents and compounds were obtained from Sigma-Aldrich, Fisher Scientific, ACROS, Ambeed, Roth or TCI. For plasmid preps, the Monarch Plasmid Miniprep Kit was used.

Synthetic genes were codon optimized for expression in *E. coli* by BioCat (Heidelberg, Germany). For CalB, the data base sequence was used (Table S1). The correct gene sequence for SpL was obtained after sequencing the plasmid obtained from Zhi Li (National University of Singapore, Singapore)[1] and the DNA sequence was ordered and the enzyme expressed using a pRSFDuet-1 vector. All plasmids encoded a C-terminal His_6 tag, except SpL, MAE2, CIH, AMI, MsAcT, 2R11, and CE13 which plasmid encoded a N-terminal His_6 tag. All used vectors to express enzymes are listed in the following table. The lipase from *Sphingomonas* HX- 200 (SpL) was ordered from biocat in a pRstDuet vector (N-terminal His_6-Tag). The gene sequence was codon optimized for *E. coli* from the amino acid sequence which was published by Li and co-workers. Due to wrongly published amino acid sequence, Li and co-workers kindly supplied us their plasmid (same vector as we ordered). Plasmid of Li and co-workers was sequenced and the enzyme's analyzed sequence was placed in "Sequences". The lipase from *Candida antarctica* (CalB) was ordered from biocat in a pET22b(+) vector (C-terminal His_6-Tag and pelB signal sequence). The gene sequence was taken from NCBI (Z30645.1) without any gene optimization for *E. coli*. The wt and the variant H143D of the lipase from *Rhizomucor miehei* (RML) was ordered from biocat in a pET-30a(+) vector (C-terminal His_6-Tag, codon optimized sequence from Hong-wei Yu and co-worker[2]). RML sequence contains a propeptide. The primers used in this work were obtained from Integrated DNA Technologies (IDT).

Table S1: Source organisms of enzymes investigated in initial screening.

Source organism	Short name	Enzyme type	UniProt accession no.	Reference or source
Mycobacterium smegmatis	MsAcT wt & variants[a]	Acyltransferase	A0R5U7	3-5
Pseudomonas protegens	PpATase-F148V	Acyltransferase	Q9TP23	6
Pseudozyma antarctica (C. antarctica)	CalB wt & variants[b]	Lipase	P41365	7
Sphingomonas sp. HXN-200	SpL wt	Lipase	-	1
Uncultured bacterium pCosCE1	EstCE1 wt & variants[c]	Lipase	Q1I192	8
Rhizomucor miehei	RML wt	Lipase	P19515	2
Alcanivorax borkumensis SK2	CE07 wt	Esterase	Q0VLP6	9
Alcanivorax borkumensis SK2	CE03 wt	Esterase	Q0VT77	9
Pseudomonas aestusnigri VGXO14	CE13 wt	Esterase	A0A218VKW9	9
Bacillus subtilis	2R11	Esterase	P96688	PDB: 2R11
Pyrobaculum calidifontis	PestE wt	Esterase	A3MVR4	10
Alyciclobacillus acidocaldarius	Est2 wt	Esterase	Q7SIG1	10
Bradyrhizobium japonicum	MAE2 wt	Malonamidase	Q9ZIV5	11
Pseudomonas putida	CIH wt	Cyclic imidase	Q4JG22	11
Rhodococcus globerulus	AMI wt	Amidase	Q76EV1	11

[a] S11C, D10A, D10I, L12A/F154, F154V/F174V, F174V, F154A/F174A, F150V/F154V, F174A, F154V, F154L, F154A, F150V/F174V, F150A/F154A, F174A/F150A, T93A/F154A
[b] QW10 (W104V/A281Y/A282Y/V149G), QW4 (W104V/S105C/A281Y/A282Y/V149G)
[c] W339Y, W339F

General, Kits, and instruments

DNA analysis or purification was performed with agarose gels. Agarose gels were prepared with agarose (1%, Invitrogen) in TAE buffer (Invitrogen) stained with SYBR Safe (Invitrogen). Samples were stained with 6x DNA Loading Dye (Thermo Fischer Scientific) and gel was run with Gene Ruler DNA Ladder Mix (Thermo Scientific) at 100 V.

The Eppendorf BioPhotometer plus was used for OD measurements, DNA concentration and protein concentration determination. The Eppendorf Mastercycler Nexus by Thermo Fisher was used to perform PCR reactions. Cell disruption was carried out with the BRANSON 250 sonifier.

In order to express the desired proteins, transformations were performed with BL21 (DE3) or Rosetta (DE3) chemically competent *E. coli* cells. The digested PCR products (Q5® Site-Directed Mutagenesis Kit from Neb) was transformed into *E. coli* NEB 5-alpha ultracompetent cells by Neb.

Plasmid preparation was performed from overnight cultures (ONC), using a Monarch Miniprep Kit by Neb following their protocol.

Transformation into *E. coli* NEB 5-alpha cells was performed for digested PCR products (two step site-directed mutagenesis) according to the High Efficiency Transformation Protocol of *E. coli* NEB 5-alpha cells. These cells were used for amplification of the plasmids prior to sequencing.

All samples that showed the correct sequence were transformation into the expression host *E. coli* BL21 (DE3). For the transformation, 1 µL DNA solution was added to 100 µL cell solution and incubated on ice for 30 min. The cells were heat shocked for 15 s at 42 °C in a heat block. Afterwards, the mixture was icubated on ice for 5 min. 900 µL LB was added and the mixture was incubated at 37 °C for 1 h, 120 rpm. 100 µL were spread onto a warm agar plate containing the appropriate antibiotic (Kan = 50 µg/mL, Amp = 100 µg/mL, or Amp = 50 µg/mL/Cam = 34 µg/mL) and cultivated overnight at 37 °C.

The protein concentration was determined by Bradford protein assay. Bradford reagent was diluted 1:4 with water. The sample solution (10 µL) was pipetted into the reagent solution (490 µL) and incubated at room temperature for 5 min before measurement at a wavelength of 595 nm with a photometer.

ExpressPlus PAGE Gels (GenScript) were used for protein analysis in MOPS buffer. Protein solutions were mixed with 2x Laemmli Sample Buffer and denatured at 95 °C for 5 min. PageRuler prestained Protein Ladder (Thermo Scientific) was used as reference. Gels were run at 120 V and stained with Coomassie Brilliant Blue solution.

Cloning and site directed mutagenesis

The site-directed mutagenesis was done with the Neb Q5 Site-Directed Mutagenesis Kit by Neb. The mutagenesis of MsAcT_S11C and _T93A/F154A were done by QuikChange Agilent. The primer design was done with the website 'https://nebasechanger.neb.com/' or with the Agilent QuikChange Primer Design Tool. The details of the mutagenesis are summarized in Tables S2-S7. The digestion of nonmutated, parental DNA and ligation was done by the KLD mix which was incubated in general for 5 min. For the four mutants, the incubation time was extended for 1 h. 5 µL were analyzed on a 1% agarose gel. Variant PestE-D156I was only feasible with an addition of 1.5 M betaine. After Step II, the mixture was incubated at room

temperature for 15 min and afterwards transformed into NEB 5-alpha competent cells and plated on agar plate with the appropriate antibiotic.

Table S2: Annealing and Elongation data for PCR reactions.

Variant	Stock conc. [ng/μL]	Annealing T [°C]	Elongation t [s]
SpL_D251V	50	70	180
SpL_S159I	50	72	150
SpL_D158I	50	72	180
SpL_H281A	50	67	150
SpL_D158E	80	72	200
SpL_ D158S	80	72	200
SpL_ D158H	80	72	200
SpL_ D158Y	80	72	200
SpL_ D158N	80	72	200
SpL_ D158R	80	72	200
SpL_ D158K	80	72	200
SpL_ D158Q	80	72	200
SpL_ D158T	80	69	200
SpL_H102N	74	70	200
SpL_H102S	74	70	200
SpL_H102Y	50	70	200
SpL_H102V	50	71.4	200
MsAcT_T12A/F154A	66	59	220
MsAcT _T93A/F154A	55	60	360
MsAcT _S11C	55	60	360
PestE_D156I	5	72	420
Est2_D155I	5	70.5	200

Table S3: Neb 5-alpha mutagenesis procedure.

**Step I – Exponential amplification**		
**Component**	**Stock concentration**	**Quantity added [μL]**
Template	See table S2	0.5
Forward primer	10 μmol	0.63
Reverse primer	10 μmol	0.63
Q5 Hot Start High-Fidelity Master Mix	2X	6.3
Autoclaved, deionized water	-	4.5
**Step II – KLD reaction**		
**Component**	**Stock concentration**	**Quantity added [μL]**
PCR product	-	0.5
KLD reaction buffer	2X	2.5
KLD enzyme mix	10X	0.5
Autoclaved water	-	1.5

Table S4: PCR conditions for Neb 5-alpha mutagenesis procedure.

Segment	Cycles	Temperature [°C]	Time [s]
Initialization	1	98	30
Denaturation		98	10
Annealing	30	See table S2	30
Elongation		72	See Table S2
Final Elongation	1	72	See Table S2
Hold		10	∞

Table S5: QuickChange Agilent kit mutagenesis procedure.

**Exponential amplification**		
**Component**	**Stock concentration**	**Quantity added [μL]**
Template	See table	0.5
Forward primer	20 μmol	1.25
Reverse primer	20 μmol	1.25
dNTPs	2 mM (each nucleotide)	1
Reaction buffer	10X	5
Quick solution reagent	-	3
PfuUltra HF DNA polymerase	2.5 U/μL	1
Autoclaved, deionized water	-	38

Table S6: PCR conditions for QuickChange Agilent kit mutagenesis procedure.

Segment	Cycles	Temperature [°C]	Time [s]
Initialization	1	95	120
Denaturation		95	50
Annealing	20	See Table S2	50
Elongation		68	360
Final Elongation	1	68	420
Cooling	1	4	120
Hold		10	∞

Table S7: Primers for mutagenesis. The position of the mutation is highlighted in blue.

Entry	Name	Template	Sequence (5' to 3')
1	SpL_D251V	Wt SpL	CGCCAGCCTCGTTCCGCTGCGCG
2	SpL_D251V	Wt SpL	GTGATCAGCAGCGTCGGC
3	SpL_S159I	Wt SpL	GTCGGGCGACATCGCGGGCGGCA
4	SpL_S159I	Wt SpL	AGGACCAGACCCGTGCAGGCAATATTGTCG
5	SpL_D158I	Wt SpL	CCTGTCGGGCATCAGCGCGGGCG
6	SpL_D158I	Wt SpL	ACCAGACCCGTGCAGGCAATATTG
7	SpL_H281A	Wt SpL	AGGTACGATCGCCGGCTATATCTGCCTCG
8	SpL_H281A	Wt SpL	TTCGCCTCGCGGTAGGTC
9	SpL_D158E	Wt SpL	CCTGTCGGGCGAAAGCGCGGGCG
10	SpL_D158E	Wt SpL	ACCAGACCCGTGCAGGCAATATTGTCGGC
11	SpL_D158S	Wt SpL	CCTGTCGGGCTCCAGCGCGGGCG
12	SpL_D158S	Wt SpL	ACCAGACCCGTGCAGGCAATATTG
13	SpL_D158H	Wt SpL	CCTGTCGGGCCATAGCGCGGGCG
14	SpL_D158H	Wt SpL	ACCAGACCCGTGCAGGCAATATTG
15	SpL_D158Y	Wt SpL	CCTGTCGGGCTATAGCGCGGGCG
16	SpL_D158Y	Wt SpL	ACCAGACCCGTGCAGGCAATATTG
17	SpL_D158N	Wt SpL	CCTGTCGGGCAATAGCGCGGGCG
18	SpL_D158N	Wt SpL	ACCAGACCCGTGCAGGCAATATTG
19	SpL_D158R	Wt SpL	CCTGTCGGGCCGCAGCGCGGGCG
20	SpL_D158R	Wt SpL	ACCAGACCCGTGCAGGCAATATTG
21	SpL_D158K	Wt SpL	CCTGTCGGGCAAAAGCGCGGGCG
22	SpL_D158K	Wt SpL	ACCAGACCCGTGCAGGCAATATTG
23	SpL_D158Q	Wt SpL	CCTGTCGGGCCAGAGCGCGGGCG
24	SpL_D158Q	Wt SpL	ACCAGACCCGTGCAGGCAATATTG
25	SpL_D158T	Wt SpL	CCTGTCGGGCACAAGCGCGGGCGG
26	SpL_D158T	Wt SpL	ACCAGACCCGTGCAGGCA
27	SpL_H102N	Wt SpL	CCTCGAAACGAACGATCCCTATTGC
28	SpL_H102N	Wt SpL	TCGCCGATCACCCAGCCG
29	SpL_H102S	Wt SpL	CCTCGAAACGTCTGATCCCTATTGCGCCGAAGC
30	SpL_H102S	Wt SpL	TCGCCGATCACCCAGCCG
31	SpL_H102Y	Wt SpL	CCTCGAAACGTATGATCCCTATTGCGC
32	SpL_H102Y	Wt SpL	TCGCCGATCACCCAGCCG
33	SpL_H102V	Wt SpL	CCTCGAAACGGTCGATCCCTATTGCGCCG
34	SpL_H102V	Wt SpL	TCGCCGATCACCCAGCCG
35	MsAcT_S11C	Wt MsAcT	CGTATTCTGTGTTTTGGTGATTGCCTGACCTGGG
36	MsAcT_S11C	Wt MsAcT	CCCAGGTCAGGCAATCACCAAAACACAGAATACG
37	MsAcT_L12A/F154A	MsAcT_F154A	TGGTGATAGCGCGACCTGGGGTTG
38	MsAcT_L12A/F154A	MsAcT_F154A	AAACACAGAATACGTTTTGC
39	MsAcT_T93A/F154A	MsAcT_F154A	GTGATTATTATGCTGGGCGCCAATGATACCAAAGCC
40	MsAcT_T93A/F154A	MsAcT_F154A	AGGCTTTGGTATCATTGGCGCCCAGCATAATAATCAC
41	Est2_D155I	Wt Est2	AGTTGGTGGTATCAGCGCCGGTGG
42	Est2_D155I	Wt Est2	GCAATGCGGGCCGGATCC
43	PestE_D156I	Wt PestE	ATATCGGCGGGGGGCAACTTA
44	PestE_D156I	Wt PestE	CCCAGCCACGGCGATTTTC

Expression of His_6-tagged hydrolases

Chemically competent *E. coli* cells were transformed with expression vectors containing the gene of interest. According to the used vector, transformants were plated on LB agar plates containing 50 or 100 µg/mL kanamycin or ampicillin, respectively.

The pre-cultures (10 mL LB containing appropriate antibiotic) were inoculated with single colonies and incubated overnight (37 °C, 120 rpm). The cultivation of bacteria was carried out by inoculation of cultivation media (TB, LB, or AutoinductionMedium (section medium)) with 1% or 0.2% (Est2 and PestE) (v/v) pre-culture supplied with the appropriate antibiotic. The cultivation was continued at 30 or 37 °C until a specific OD_{600} was reached. The expression of the heterologous genes was started by the addition of the appropriate inducer (isopropyl-β-D-thiogalactoside (IPTG) or lactose) to a specific concentration, followed by incubation at a specific temperature (120 rpm) for 16 h. Deviations from this standard protocol and specific requirements are listed in the following table.

Table S8: Expression protocols of used enzymes and vector used for heterologous expression.

Enzyme	Expression protocol	Antibiotic resistance	Host	Vector	pEG[a]
**SpL + variants**	TB, 37 °C, 120 rpm; OD_{600} = 0.6-0.9 +0.25 mM IPTG, 20 °C, 120 rpm O/N	kanamycin	BL21(DE3)	pRSFDuet-1	626[b]
**MsAcT + variants**	LB, 37°C, 120 rpm; OD_{600} = 0.6-0.9 +0.5 mM IPTG, 20 °C, 120 rpm O/N	kanamycin	BL21(DE3)	pET28a(+)	357[a]
**CE07**	AutoMedium, 37 °C, 120 rpm; first 30 °C for 4 h, then 20 °C, 120 rpm O/N	kanamycin	BL21(DE3)	pET-26b(+)	775
**CE03**	AutoMedium, 37 °C, 120 rpm; first 30 °C for 4 h, then 20 °C, 120 rpm O/N	kanamycin	BL21(DE3)	pET-26b(+)	776
**CE13**	LB, 37 °C, 120 rpm; OD_{600} = 0.6-0.9 +0.5 mM IPTG, 20 °C, 120 rpm O/N	kanamycin	BL21(DE3)	pRSFDuet-1	779
**CalB + variant**	TB, 37 °C, 120 rpm; OD_{600} = 0.6-0.9 + 0.1 mM IPTG, 16 °C, 120 rpm O/N	ampicillin	Rosetta™ (DE3)	pET22b(+)	-
**EstCE1+ variants**	LB, 37 °C, 120 rpm; OD_{600} = 0.6-0.8 +0.4 mM IPTG, 20 °C, 120 rpm O/N	kanamycin	BL21(DE3)	pET-24c(+)	778[a]
**RML+ variants**	LB, 37 °C, 120 rpm; OD_{600} = 0.6-0.8 +0.1 mM IPTG, 16 °C, 120 rpm O/N	kanamycin	BL21(DE3)	pET30a(+)	-
**MAE2**	LB, 37 °C, 120 rpm; OD_{600} = 0.4 + 1 mM IPTG, 37 °C, 120 rpm 3 h	kanamycin	BL21(DE3)	pET-28a(+)	214
**CIH**	LB, 37 °C, 120 rpm; OD_{600} =0.6-0.8 +0.05 mM IPTG, 20 °C, 120 rpm O/N	kanamycin	BL21(DE3)	pET-28a(+)	215
**AMI**	LB, 37 °C, 120 rpm; OD_{600} = 0.6-0.8 +0.05 mM IPTG, 20 °C, 120 rpm O/N	kanamycin	BL21(DE3)	pET-28a(+)	212
**PpATase-F148V**	LB, 37 °C, 120 rpm; OD_{600} = 0.7 + 200 μg/L AHTC, 30 °C, 120 rpm O/N	ampicillin	BL21(DE3)	pASK-IBA3Plus	507
**2R11**	LB, 30 °C, 120 rpm; OD_{600} = 0.6-0.8 + 0.3 mM IPTG, 20 °C, 120 rpm O/N	kanamycin	Shuffle T7 (DE3)	pSPeedET	325
**PestE+ variants**	TB, 37 °C, 120 rpm; OD_{600} = 0.6-0.8 + 0.1 mM IPTG, 20 °C, 120 rpm O/N	ampicillin	BL21(DE3)	pET-21a(+)	773[a]
**Est2+ variants**	TB, 37 °C, 120 rpm; OD_{600} = 0.6-0.8 + 0.1 mM IPTG, 20 °C, 120 rpm O/N	kanamycin	BL21(DE3)	pET-28a(+)	774[a]

[a] pEG stand for ‘plasmid of the ElkGroup’ and is an internal number. [b] = Wt

Cells were harvested by centrifugation (at 8,000 x g at 4 °C for 15 min) and the cell pellet was washed with 20 mL 50 mM KP*i* buffer (pH 7.5). Washed cell pellets were stored at -20°C for later use. Otherwise, the resuspension was lysed by sonification using a Branson Ultrasonics™ Sonifier™ (Table S9). The cell suspension was sonicated according to the protocol for the expressed enzyme. Insoluble cell residues were removed by centrifugation at 18,000 x g at 4 °C for 20 min. The supernatant was lyophilized with a freeze dryer (CHRIST Alpha 1-4 LSCbasic) overnight.

Table S9: Sonication procedure for the used enzymes.

Enzyme	Sonication procedure	Repetitions
SpL+ variants	5 min, 1 s on/ 2 s off, 40% amplitude	1x
MsAcT + variants	4 min, 1 s on/ 2 s off, 20% amplitude	1x
CE07	4 min, 1 s on/ 2 s off, 20% amplitude	1x
CE03	4 min, 1 s on/ 2 s off, 20% amplitude	1x
CE13	4 min, 1 s on/ 2 s off, 30% amplitude	1x
CalB + variant	4 min, 1 s on/ 2 s off, 30% amplitude	1x
EstCE1 + variants	4 min, 1 s on/ 2 s off, 20% amplitude	1x
RML + variants	3 min, 1 s on/ 2 s off, 30% amplitude	1x
MAE2	5 min, 1 s on/ 4 s off, 40% amplitude	1x
CIH	5 min, 1 s on/ 4 s off, 40% amplitude	1x
AMI	5 min, 1 s on/ 4 s off, 40% amplitude	1x
PpATase-F148V	8 min, 1 s on/ 4 s off, 40% amplitude	1x
2R11	4 min 2 s on/ 2 s off, 10% amplitude	1x
Est2 + variant	4 min, 5 s on/ 5 s off, 40% amplitude	1x
PestE + variant	4 min, 5 s on/ 5 s off, 40% amplitude	1x

Purification of His_6-tagged SpL

The His_6-tagged SpL was purified by immobilised metal-affinity chromatography using 5 ml of HisTrap^{TM} FF (Cytiva, Massachusetts, United states). The Ni-resin was washed with deionized water and equilibrated with “Lysis” buffer (300 mM NaCl, 50 mM KH_2PO_4 at pH 7.5, 20 mM imidazole) via an ÄKTA. The enzyme solution was applied via a pump attached to the ÄKTA and the flow-through was discarded. Non-specifically bound proteins were removed by washing the column with the “Lysis” buffer. The SpL was eluted with a gradient using the buffer “Lysis” and “Elution” (300 mM NaCl, 50 mM KH_2PO_4 at pH 7.5, 250 mM imidazole). Afterwards, the eluted enzyme was concentrated with a Vivaspin 10K until a volume of 2.5 mL was reached. A PD-10 desalting columns (GE Healthcare, UK) was used to exchange the buffer to 50 mM KPi buffer at pH 7.5.

For crystallization experiments, the IMAC purified enzyme was concentrated with a Vivaspin 50K until a volume of 2 mL was reached to be further purified by gel filtration on a Superdex 200 16/60 column (GE Healthcare, Freiburg, Germany) with “Protein” buffer (150 mM NaCl, 10 mM Tris*HCl at pH 7.5). Protein concentrations were determined by measuring absorbance at 280 nm using a NanoDropTM (Thermo Fisher, Germany) device. Theoretical extinction coefficients and molecular weights were calculated from the protein sequences using ExPASy ProtParam.

Purification of His_6-tagged Est2-D155I & PestE-D156I

The His_6-tagged SpL was purified by immobilised metal-affinity chromatography using 5 ml of HisTrap^{TM} FF (Cytiva, Massachusetts, United states). The Ni-resin was washed with deionized water and equilibrated with “Lysis” buffer (300 mM NaCl, 50 mM KH_2PO_4 at pH 8.0) via an ÄKTA. The enzyme solution was applied via a pump attached to the ÄKTA and the flow-

through was discarded. Non-specifically bound proteins were removed by washing the column with the "Washing" buffer (300 mM NaCl, 20 mM imidazole, 50 mM KH_2PO_4 at pH 8.0). The variants were eluted with a gradient using the buffers "Washing" and "Elution" (300 mM NaCl, 300 mM imidazole, 50 mM KH_2PO_4 at pH 8.0). Afterwards, the eluted enzyme was concentrated with a Vivaspin 10K until a volume of 2.5 mL was reached. A PD-10 desalting columns (GE Healthcare, UK) was used to exchange the buffer to 50 mM KPi buffer at pH 8 for Est2-D155I. Same elution buffer was used for PestE-D156I, but afterwards the pH was adjusted to pH 11.7 due to enzyme precipitation.

SDS-PAGE Analysis

SDS-PAGE was used to confirm the solubility of the expressed enzyme and to analyze the purity. Samples were mixed with SDS-PAGE 2x Laemmli-buffer (commercial) and denatured by heating at 95 °C for 5 min followed by centrifugation (max rpm, 5 s). Commercial SDS-PAGE gels from BioRad were used. Depending on the needed number of wells, 12 or 10% gels were used. To run the gel a constant voltage of 120 V was used. The gels were stained using Coomassie Brilliant Blue G-250 and destained with deionized water.

SDS-PAGE SpL variants and wt

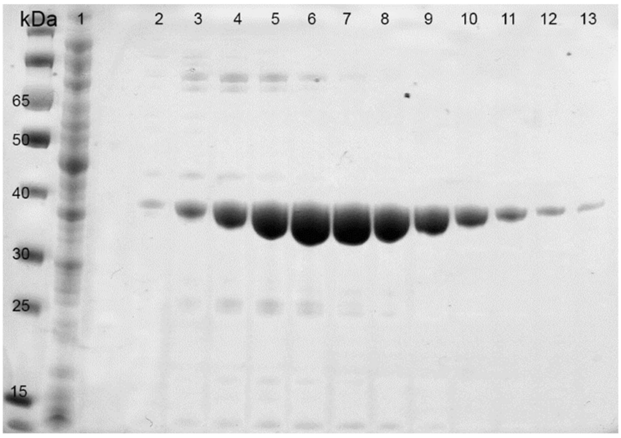

Figure S1: SDS-PAGE of selected SpL Wt fractions from His_6tag-purification. First lane: ladder; lane (1): Flow through; lanes (2-13): eluted fractions.

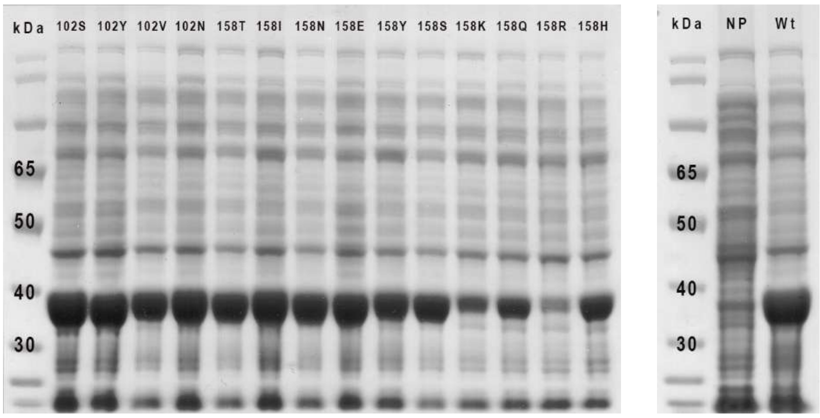

Figure S2: Expressed SpL variants. Number tells position in the amino acid chain and letter indicates the variant. NP (no plasmid) shows the content of cultivated empty BL21(DE3) and Wt shows the content of SpL Wt. Each well contains 10 µL of 5 mg/mL CFE solution.

SDS-PAGEs other enzymes

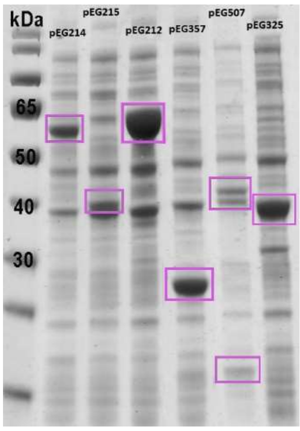

Figure S3: SDS-PAGE of selected expressed enzymes. The first line shows the ladder, followed by MAE2 (pEG214), CIH (pEG215), AMI (pEG212), MsAcT WT (pEG357), PpATaseF148V (pEG507) and 2R11 (pEG325). The corresponding protein band is highlighted in a purple box. E. coli *cell suspension after expression was normalized to OD_{600} = 7 and mixed in a 1:1 ratio with SDS-PAGE 2x loading buffer (commercial). 10 µL of that mix was loaded on this SDS-PAGE gel.*

For the SDS-PAGE of the MsAcT variants please refer to the SI of a published paper,[1] as the preparation analyzed there was used. It can be stated that the expression level was comparable to the wt as shown here.

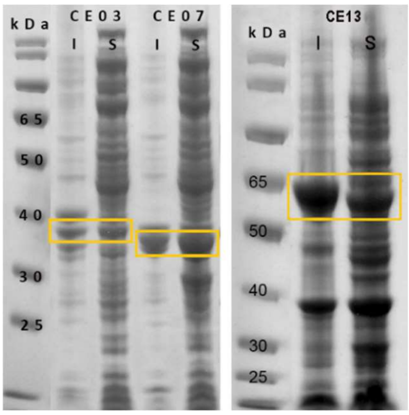

Figure S4: SDS-PAGE of lysed cells containing CE03, 07, and 13. First line = ladder, I = insoluble, and S = soluble. After enzyme expression and sonication, a certain volume of supernatant was used and mixed in a 1:1 ratio with SDS-PAGE 2x loading buffer (commercial). 10 µL of that mix was loaded on this SDS-PAGE gel. The corresponding protein band is highlighted in an orange box.

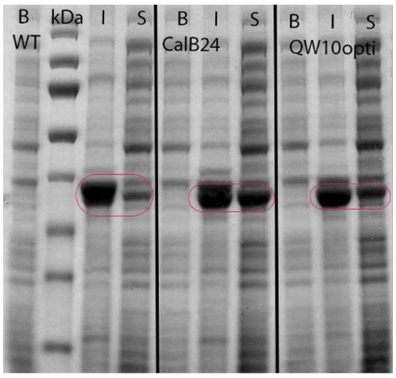

Figure S5: SDS-PAGE of lysed cells containing CalB wt and variants. B = before induction, I = insoluble, and S = soluble.

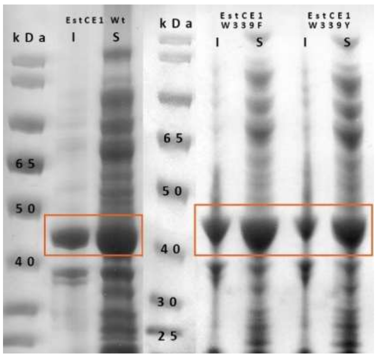

Figure S6: SDS-PAGE of lysed cells containing EstCE1 and its variants EctCE1-W339F and W339Y. After enzyme expression and sonication, a certain volume of supernatant was used and mixed in a 1:1 ratio with SDS-PAGE 2x loading buffer (commercial). 10 µL of that mix was loaded on this SDS-PAGE gel.

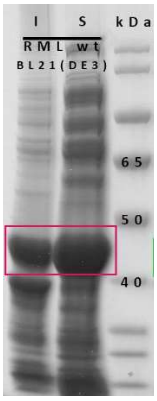

Figure S7: SDS-PAGE of the insoluble (I) and soluble fraction (S) of RML wt. After enzyme expression and sonication, a certain volume of supernatant was used and mixed in a 1:1 ratio with SDS-PAGE 2x loading buffer (commercial). 10 µL of that mix was loaded on this SDS-PAGE gel.

Crystallization of SpL and Data Collection

Protein crystallization was conducted by vapor diffusion technique, with the sitting drop method using a Swissci triple well plate (Swissci AG, Neuheim, Switzerland) with a reservoir volume of 30 µL, and an Oryx8 robot (Douglas Instruments Berkshire, UK) employing the Index screen (Hampton Research, Viejo, USA). The protein was prepared in “Protein” buffer, as described above for crystallization. Crystals were grown in several conditions. The first crystal was collected by mixing 0.5 µL Ammonium acetate (0.2 M), BIS-TRIS (0.1 M, pH 5.5) and 25% PEG 3350 with 0.5 µL SPL (11.70 mg/mL). Crystals appeared within 1-3 days and were frozen in liquid nitrogen without cryo-protection for diffraction experiments. A data set with a resolution of 2 Å was collected at the P11 beamline of the DESY in Hamburg (Germany) at 100 °K. Data processing was performed with the XDS program package. Unit cell parameters and assigned space groups as well as data statistics are shown in Table S10.

Further crystals were obtained by the method described above, by mixing 0.5 µL Magnesium chloride hexahydrate (0.02 M), HEPES (0.1 M, pH 7.5) and 22% Poly(acrylic acid sodium salt) with 0.5 µL SpL (11.70 mg/mL) as well as by mixing 0.5 µL BIS-TRIS (0.1 M, pH 6.5) and 25% PEG 3350 with 0.5 µL SPL (11.70 mg/mL). These crystals were harvested after soaking for 5 minutes in saturated solutions of the amide product and benzylamine respectively, prepared in the reservoir solution of their crystallization condition. Data collection and processing

happened as described above, and a data set with a resolution of 1.8 Å and 1.5 Å was collected from the crystals respectively.

Structure Solution and Refinement

Data sets were analyzed with Aimless[12, 13] in CCP4 Cloud.[14] The solvent content was estimated based on the calculated Matthews coefficient.[15] Phasing was performed using chain A of 4UU3 from the PDB as template for phaser MR.[16] Refinement was conducted by repetitive rounds of REFMAC[17] in the CCP4 cloud program package and manual model building in COOT.[18] Refinement cycles have also been performed with phenix.refine to improve the model quality. Models have been deposited with the PDB codes found in Table S10.

Table S10: Crystallographic statistics of the three structures deposited in the PDB: native SPL (8OIM), SPL in complex with benzylamine (8P7E) and SPL in complex with N-benzyl-picolinamide (8P8F).

Structure:	SPL	SPL-Benzylamine	SPL-Amide
**PDB code:**	**8OIM**	**8P7E**	**8P8F**
Resolution range (Å)	42.05 - 1.99 (2.06 - 1.99)	35.75 - 1.5 (1.55 - 1.5)	46.62 - 1.8 (1.86 - 1.8)
Space group	P 1 21 1	P 1 21 1	P 43
Unit cell (Å,°)	60.8 89.0 65.6 90 96.4 90	61.0 88.6 65.8 90 96.2 90	60.8 60.8 217.7 90 90 90
Total reflections	87043 (8242)	218507 (21574)	145515 (14564)
Unique reflections	44835 (4291)	109840 (10934)	72765 (7282)
Multiplicity	1.9 (1.9)	2.0 (2.0)	2.0 (2.0)
Completeness (%)	94.54 (91.08)	99.01 (98.77)	99.97 (100.00)
Mean I/sigma(I)	9.64 (3.79)	11.65 (4.60)	11.34 (2.38)
Wilson B-factor	26.87	12.92	24.57
R-merge	0.05 (0.19)	0.04 (0.15)	0.03 (0.19)
R-meas	0.07 (0.27)	0.05 (0.22)	0.04 (0.26)
R-pim	0.05 (0.19)	0.04 (0.15)	0.03 (0.19)
CC1/2	0.99 (0.93)	0.99 (0.93)	1 (0.88)
CC*	1 (0.98)	1 (0.98)	1 (0.97)
Reflections used in refinement	44791 (4288)	109831 (10929)	72762 (7282)
Reflections used for R-free	2309 (198)	5442 (568)	3612 (325)
R-work	0.16 (0.21)	0.14 (0.18)	0.19 (0.26)
R-free	0.20 (0.26)	0.16 (0.20)	0.22 (0.27)
CC(work)	0.95 (0.93)	0.98 (0.94)	0.96 (0.81)
CC(free)	0.93 (0.84)	0.96 (0.93)	0.94 (0.75)
Number of non-hydrogen atoms	5034	5753	5256
Macromolecules	4674	4742	4674
Ligands	0	238	56
Solvent	360	899	550
Protein residues	618	618	618
RMS(bonds) (Å)	0.016	0.012	0.014
RMS(angles) (°)	2.03	1.75	1.89
Ramachandran favored (%)	97.39	97.88	96.91
Ramachandran allowed (%)	2.61	2.12	3.09
Ramachandran outliers (%)	0.00	0.00	0.00
Rotamer outliers (%)	1.69	0.41	1.27
Clashscore	3.68	3.62	2.37
Average B-factor ($Å^2$)	33.36	18.88	27.86
- macromolecules ($Å^2$)	32.88	16.17	26.55
- solvent ($Å^2$)	39.65	31.89	38.15
- ligands ($Å^2$)	N.A.	29.11	41.98

Ligand occupancy, omit maps

The occupancy of the soaked ligands in both structures was confirmed by generating a polder electron density map, omitting the ligands from the model, and masking the bulk solvent around their position (Figure S8). In the case of the amide product, the catalytic serine was also omitted, as it formed a contiguous electron density with the ligand. The placement of the amide was unequivocally possible because of the clear density of the carbonyl oxygen pointing at the oxyanion whole and its relation to the histidine of the catalytic triad. The occupancy of this ligand was refined to a value of 0.7 in both chains. The omit maps for the three benzylamine molecules in each chain were also well-defined (with the exception of the phenyl ring of ABN3) and clearly supported the placement of these ligands.

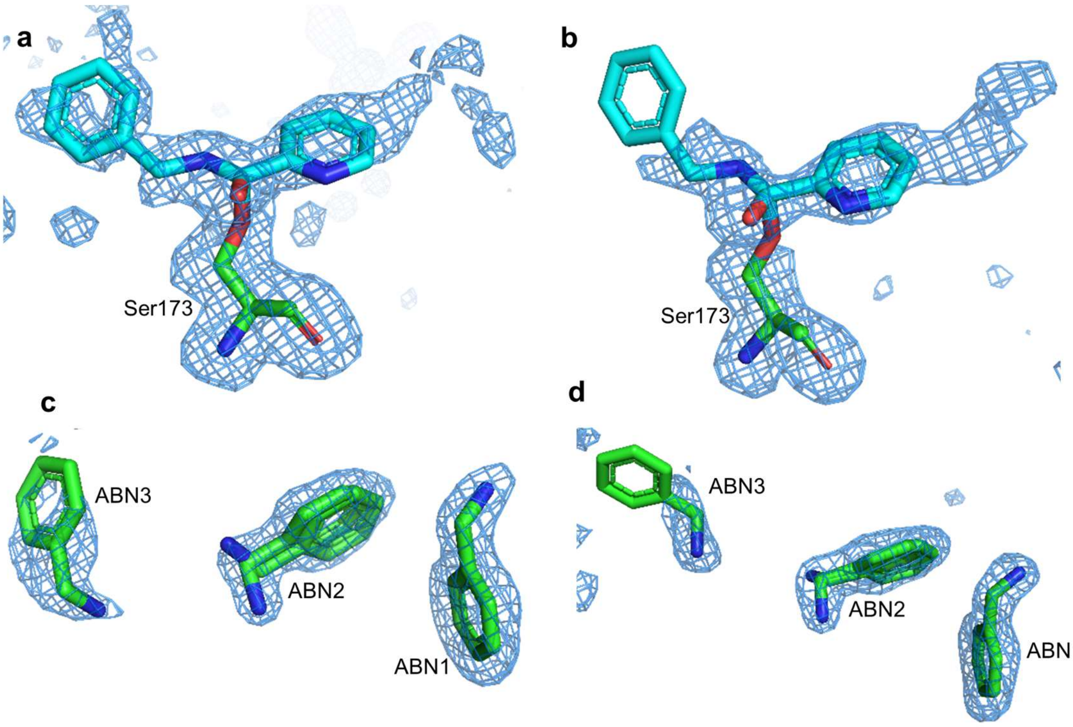

Figure S8: Polder omit maps contoured at 4σ around the soaked amide (panel **a** for chain A and panel **b** for chain B) and benzylamine (*panel* **c** for chain A and panel **d** for chain B). The figure was prepared using the program PyMOL (https://pymol.org).

HPLC analysis

Conversions were determined by high pressure liquid chromatography (HPLC), equipped with a reverse stationary phase column (Luna Omega 1.6 *µ*m C18 (A012), 100 Å, LC column 50 x 2.1 mm). Conversions of the pH screening were determined by ultra high pressure liquid chromatography (UHPLC), equipped with a Shimadzu instrument and a Luna Omega 1.6 µm C18 100 Å LC column with dimensions of 50*2.1 mm. Both used UV detection.

Following methods were used for HPLC:

For reactions with ethyl picolinate: 0.6 mL/min, 32 min at 30 °C, starting with 10 % MeCN until 20% (2 min), then 20% isocratic for 5 min and a gradient from 20% to 100 % (18 min). Water and acetonitrile contained 0.1% TFA.

For reactions with ethyl benzoate or ethyl furoate: 1 mL/min, 32 min at 30 °C, 10% to 100 % (from 2 min until 28 min). Water and acetonitrile contained 0.1% TFA.

Following methods were used for UHPLC:

Compounds containing 2-furane moiety: 0.5 mL/min, 20 min at 30 °C, starting with 10% MeCN to 100% water. Water and acetonitrile contained 0.1% TFA.
Compounds containing picoline moiety: 0.5 mL/min, 9 min at 30 °C, starting with 5% MeCN to 100% water. Water and acetonitrile contained 0.1% TFA.

Kinetic studies

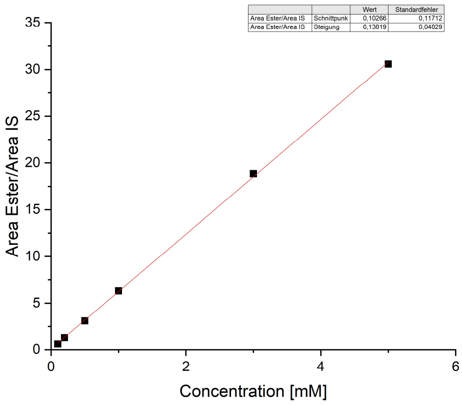

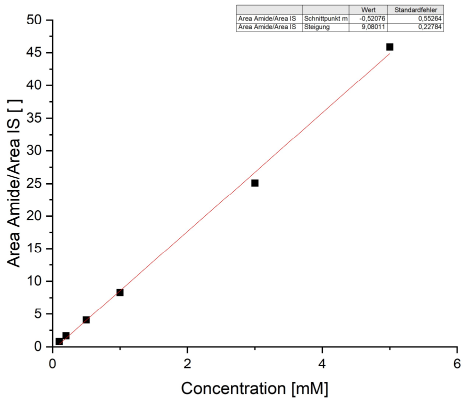

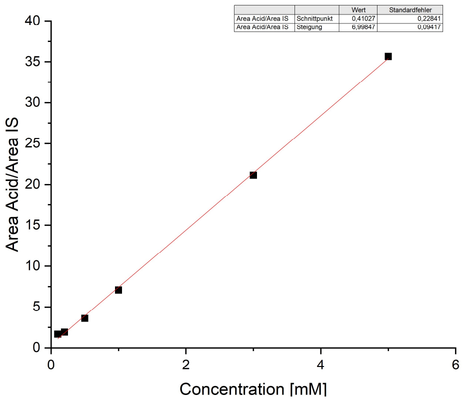

Figure S9: Calibration of ethyl picolinate, picolinic acid and N-benzyl-2-pyridinecarboxamide.

Determination of the activity of CFE and purified SpL for the hydrolysis of *p*-NPB

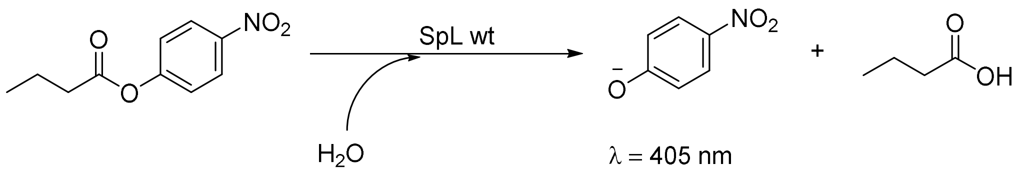

Scheme S1: Reaction scheme of kinetic assay with pNPB.

KPi-buffer (200 mM, pH 7.5) and substrate *p*NPB (0.1 mM, added from 10 mM stock solution prepared in acetonitrile) were added to a cuvette at rt. The reaction (1 mL total volume) was started by the addition of purified SpL (0.09 μg) or CFE (1.3 μg). The reaction was followed over 1 minute. An increase of absorption was recorded due to the formation of pNP (λ = 405 nm). All reactions were performed in duplicates. The protein concentration was measured via Bradford. Background reaction mixture consisted of the same mixture, but without enzyme. Except for the CFE reactions, there CFE from empty BL21(DE3) was used for the background reaction.

$$\varepsilon = 15383.33 \frac{L}{mol} * cm$$

$$c = \frac{x}{\varepsilon}$$

$$c = \frac{0.9946 \frac{A}{min}}{15383.33 \frac{L}{mol} * cm}$$

$$c = 6.465 * 10^{-8} molmL^{-1} * min$$

$$c = 0.06465 \mu molmin^{-1} perx \mu genzyme$$

$$SA = \frac{U}{m}$$

$$SA = \frac{0.06465 \mu molmin^{-1} perx \mu genzyme}{0.000091 mg}$$

$$SA = 710.5 \mu molmin^{-1} mg^{-1} enzyme$$

$$SA = 710.5 Umg^{-1}$$

Measurements were done twice. The mean value is 708 U mg^{-1}. Same was done with CFE. The mean activity for CFE was 84 U mg^{-1}.

Determination of the activity of CFE and purified SpL Wt for amide formation

For the time study, a 1:4 ration of ester to amine was chosen. For the reaction mixtures, 42 µL 2 M HCl (final 42 mM) was mixed with 838 µL deion. water. Afterwards, 1000 µL of 400 mM KPi buffer (pH 7.5) was added. 80 µL of 1 M benzylamine DMSO stock (40 mM) and 20 µL of 1 M ethyl picolinate DMSO stock (10 mM) were added. Final DMSO concentration was 5%. At last, 20 µL CFE stock solution (1 mg/mL CFE in water, final 0.02 mg/mL) was added to start the reaction. These mixtures were then incubated at 30 °C and 800 rpm. Samples were taken after 0, 5, 10, 20, 40, 60, 180, 300, 420, and 1560 min (Figure S10). After a specific time, 100 µL sample was taken and quenched with 100 µL 2 mM benzoic acid in MeOH. Preparation was centrifuged for 10 min at max speed before being measured on HPLC. To calculate the specific activity (SA), the first 4 points of the amide formation were used for linear regression.

$$y = 0.0428x + 0.1016, R^2 = 0{,}9969$$

$$U = \frac{0.0428mMmin^{-1} * 2mL}{1000mL} * 1000$$

$$U = 0.0000856mmolmin^{-1}$$

$$SA = \frac{0.0858\mu molmin^{-1}}{0.02mg}$$

$$SA = 4.29Umg^{-1}$$

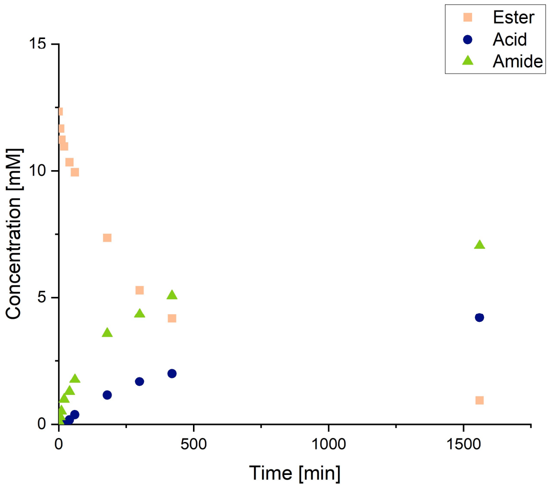

Figure S10. Time course of the reaction ethyl picolinate and benzylamine over 26 h.

The reaction mixture for the activity calculations of lyophilized, purified SpL had the same conditions as described above, with the exception that the final volume was 1 mL and an enzyme concentration of 0.05 mg mL^{-1} was used (calculated from Bradford). The first 4 points of the amide formation were used for linear regression. Calculations were done as follows:

$$y = 0.0138x + 0.0091, R^2 = 0{,}9939$$

$$U = \frac{0.0138mMmin^{-1} * 1mL}{1000mL} * 1000$$

$$U = 0.0138\mu molmin^{-1}$$

$$SA = \frac{0.0138\mu molmin^{-1}}{0.0005mg}$$

$$SA = 26.2Umg^{-1}$$

Inhibition by benzylamine

Specific amounts of 2 M HCl had to be added to maintain a pH of 7.5. The amount of 2 M HCl was experimentally figured out. Following table shows the composition of the reaction mixtures that were used for the amine inhibition study. Purified enzyme was used as a stock of 0.05 mg/mL. The reaction was started by adding the enzyme solution. Final DMSO concentration was read from left to right 2, 3, 4, 5, 6, and 7%. These mixtures were then incubated at 30 °C and 800 rpm for 30 min. Afterwards, the samples were quenched with 250 μL MeOH and centrifuged for 10 min at max speed before being measured on HPLC.

Table S11: Reaction mixture for kinetic study keeping ester concentration at 10 mM and varying the amine concentration.

	Amine concentration [mM]						
	**50**	**40**	**30**	**20**	**15**	**10**	**Final c [mM]**
400 mM KPi, pH 7.5 [µL]	500	500	500	500	500	500	200
dH_2O [µL]	390	409.8	430	450	457	465	-
2 M HCl [µL]	40	30.2	20	10	8	5	see note
1 M amine [µL]	50	40	30	20	15	10	40
1 M ester [µL]	10	10	10	10	10	10	-
0.05 mg/mL purified SpL [µL]	10	10	10	10	10	10	0.5 µg/mL
Finale V [µL]	1000	1000	1000	1000	1000	1000	-

Note: HCl final concentration from left to right: 80, 60.4, 40, 20, 16, and 10 mM

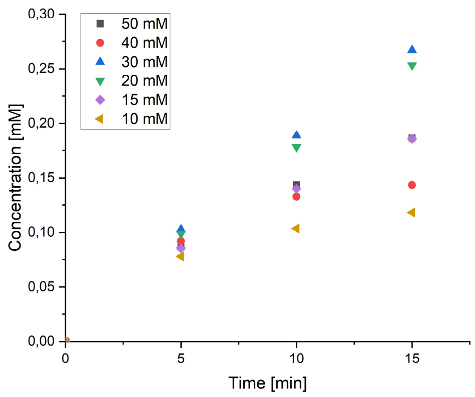

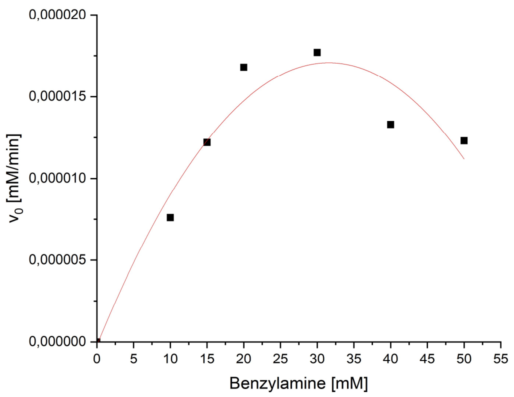

Figure S11: Testing different amine ratios in the reaction ethyl picolinate and benzylamine.

pH screening of SpL Wt

For the reaction mixtures, 25 mg of CFE were dissolved in 5 mL of 200 mM buffer (Table S12). The reaction mixture was created with 870 µL of buffer for a) or 930 µL for b). To this, 10 µL of 1 M ethyl 2-furoate or ethyl picolinate DMSO stock (10 mM) were added. Then 20 µL of 1 M benzylamine DMSO stock (20 mM) were added. Final DMSO concentration was 3%. These mixtures were then incubated for 5 minutes at 30 °C and 700 rpm without the enzyme. Afterwards, the enzyme solution was added (100 µL for reaction a)
(0.5 mg/mL CFE) and 40 µL for reaction b) (0.2 mg/ml CFE). The reaction mixture was incubated for 10 minutes at 30 °C and 700 rpm. The reactions were quenched with 50 µL of 2 M HCl.

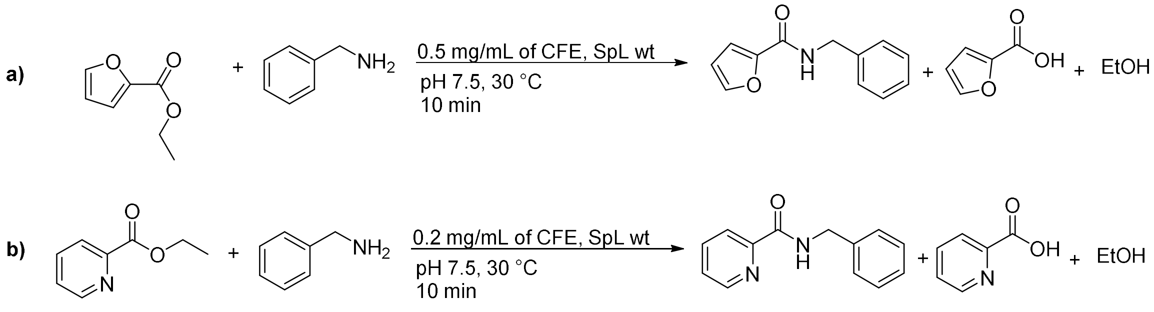

Scheme S2: Reactions for pH screening.

For the blank reaction mixtures, 25 mg of cell free extract from *E. coli* without SpL plasmid were dissolved in 5 mL of 200 mM buffer. The reaction mixture was created with 890 µl of buffer for the ethyl 2-furoate and 950 µl for the ethyl picolinate. To this, 10 µL of a 1 M DMSO stock solution (10 mM) were added. This mixture without the CFE was incubated for 5 minutes at 30 °C and 700 rpm before adding the *E. coli* CFE solution, 100 µL (0.5 mg/mL) for the mixture containing 1a and 40 µL (= 0.2 mg/ml CFE) for the mixture containing 1b. The reaction mixture was incubated for 10 minutes at 30 °C and 700 rpm. The reactions were quenched with 50 µL of 2 M HCl.

The preparation for the UHPLC measurement was done by combining 300 µL methanol with 300 µL of the reaction mixture. This was then centrifuged at 15,000 rpm for 15 minutes. 200 µL of the supernatant was used for the UHPLC measurement.

Table S12: Buffers and their pH values used for the pH screening.

pH value	Buffer
4	Sodium citrate
4.5	
5	
5.5	
6.0	
6.0	Potassium phosphate (KP*i*)
6.5	
7	
7.5	
8	
8	Bicine
8.5	
9	
9	CHES
9.5	
10	
10	CAPS
10.5	
11	

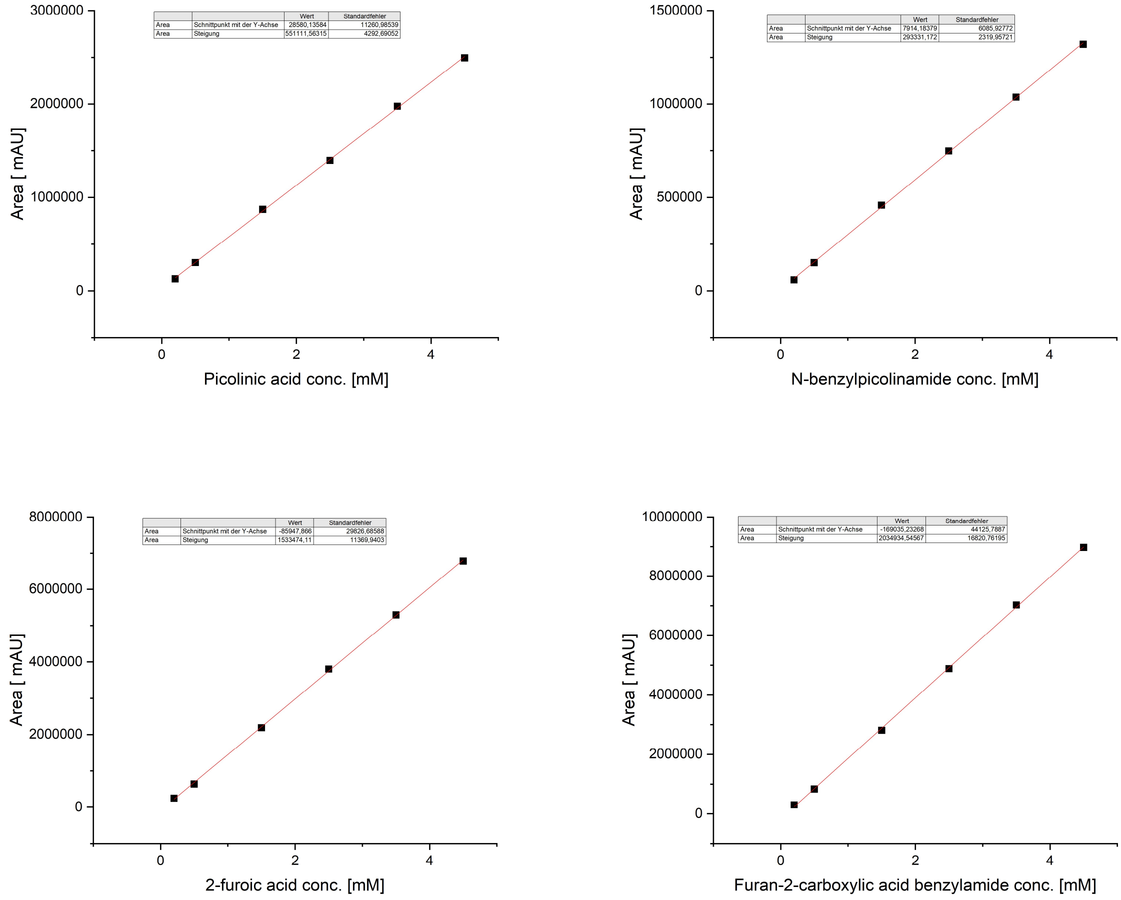

Figure S12: Calibration of acid and amide for the pH screening.

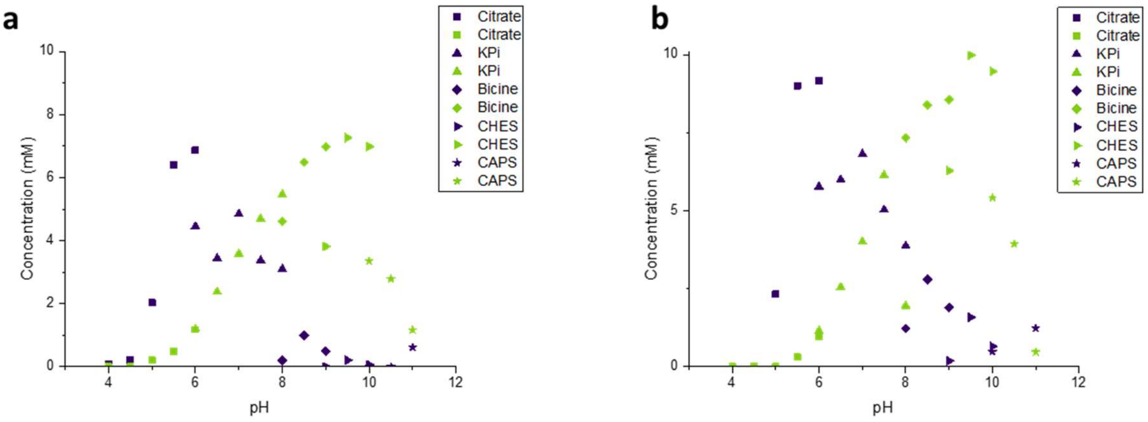

Figure S13: Influence of pH on the biocatalytic amide formation of the esters ethyl picolinate (a), ethyl 2-furoate (b) with benzylamine. Reaction conditions: 10 mM ethyl picolinate/ethyl 2-furoate, 20 mM benzylamine, 3% DMSO, 200 mM KPi buffer (pH 7.5), 0.2 mg mL^{-1} CFE for ethyl picolinate and 0.5 mg mL^{-1} CFE for ethyl 2-furoate, at 30 °C, 800 rpm, 10 min. **Blue color** indicates acid formation and **green color** indicates amide formation.

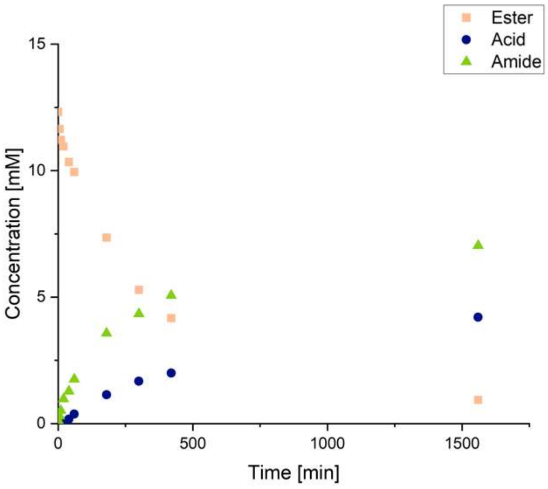

Figure S14: Time course of the amide formation starting from ethyl picolinate and benzylamine over 26 h. Reaction conditions: 10 mM ethyl picolinate, 40 mM benzylamine, 5% DMSO, 200 mM KPi buffer (pH 7.5), 0.01 mg mL-1 CFE, at 30 °C, 800 rpm.

Biotransformation screening

In vitro biotransformation reactions were conducted with CFE (preparation see 5.8 and 5.10) in closed 1.5 mL microcentrifuge tubes while shaking at 30 °C and 600 rpm in a table thermo-shaker. Total reaction volume was 1 mL consisting of KPi buffer (200mM) at pH 7.5 and containing lyophilized CFE (10 mg) and substrates (10 mM ester & 20 mM amine) which were added from a 1 M DMSO stock solution. The reaction mixtures contained 3% (30 mM in total) DMSO. For the reaction work up for TLC analysis, reaction solution (100 µL) was mixed with 2 M HCl (20 µL) and was then extracted once with ethyl acetate (100 µL) for further analysis. For HPLC analysis, 100 µL sample was mixed with 200 µL MeOH (for HPLC) and centrifuged for 15 min at maximum speed. Afterwards, 200 µL were taken for the HPLC analysis.

In the initial screening, no products were observed with piperidine and methylamine. Some esters did also not react with allylamine, these experiments were then not performed on preparative scale.

Table S13: Initial screening of biocatalysts for amide formation and ester hydrolysis in buffer using ethyl 2-furoate and benzylamine as substrate.

Enzyme	Catalytic triad	Acid formation	Amide formation
MsAcT wt	S/H/D	Yes	++
MsAcT S11C	C/H/D	No	-
MsAcT D10A	S/H/D	Yes	+
MsAcT D10I	S/H/D	Yes	+
MsAcT L12A/F154A	S/H/D	Yes	++
MsAcT F154V/F174V	S/H/D	Yes	++
MsAcT F174V	S/H/D	Yes	++
MsAcT F154A/F174A	S/H/D	Yes	+
MsAcT F150V/F154V	S/H/D	Yes	++
MsAcT F174A	S/H/D	Yes	++
MsAcT F154V	S/H/D	Yes	++
MsAcT F154L	S/H/D	Yes	++
MsAcT F154A	S/H/D	Yes	+
MsAcT F150V/F174V	S/H/D	Yes	++
MsAcT F150A/F154A	S/H/D	Yes	+
MsAcT F174A/F150A	S/H/D	Yes	+
MsAcT T93A/F154A	S/H/D	Yes	+
SpL wt	S/H/D	Yes	+++
CE13 wt	S/H/D	Yes	-
CE03 wt	S/H/D	Yes	+
CE07 wt	S/H/D	Yes	+
MAE2 wt	S/S/K	No	-
CIH wt	not described	No	-
AMI wt	not described	No	-
PpATase F148V	not described	No	-
2R11	S/H/E	No	-
CalB wt	S/H/D	Yes	-
CalB QW10	C/H/D	Yes	+
CalB QW4	S/H/D	Yes	-
Est2 wt	S/H/D	Yes	++
PestE wt	S/H/D	Yes	++
EstCE1 wt	S/H/D	Yes	+
EstCE1 W339Y	S/H/D	Yes	+
EstCE1 W339F	S/H/D	Yes	+
RML	S/H/D	No	-

Legend: low (+ = 0-14%), medium (++ = 15-70%), very good (+++ = >70%)

Table S14: SpL, PestE and Est2 tested for possible amide formation in initial screening.

Entry	Amide	PestE[a]	Est2[a]	SpL
1	**3a,a**	++	++	+++
2	**3a,b**	++	++	+++
3	**3a,c**	+	+	++
4	**3a,d**	-	-	++
5	**3a,e**	-	-	-
6	**3b,a**	-	-	++
7	**3b,b**	-	-	++
8	**3b,c**	-	-	+
9	**3b,d**	-	-	-
10	**3b,e**	-	-	-
11	**3c,a**	+	++	+++
12	**3c,b**	++	++	+++
13	**3c,c**	-	+	++
14	**3c,d**	-	-	++
15	**3c,e**	-	-	-
16	**3d,a**	+	++	++
17	**3d,b**	+	++	++
18	**3d,c**	-	-	++
19	**3d,d**	-	-	+
20	**3d,e**	-	-	-
26	**3e,a**	-	+	+++
27	**3e,b**	-	+	+++
28	**3e,c**	-	+	++
29	**3e,d**	-	-	-
30	**3e,e**	-	-	-
51	**3f,a**	-	-	++
52	**3f,b**	-	-	++
53	**3f,c**	-	-	+
54	**3f,d**	-	-	-
55	**3f,e**	-	-	-
21	**3g,a**	-	+	++
22	**3g,b**	-	+	++
23	**3g,c**	-	-	++
24	**3g,d**	-	-	+
25	**3g,e**	-	-	-
41	**3h,a**	-	-	++
42	**3h,b**	+	+	+++
43	**3h,c**	+	+	+++
44	**3h,d**	+	+	++
45	**3h,e**	-	-	-
46	**3i,a**	-	-	++
47	**3i,b**	-	-	++
48	**3i,c**	-	-	+
49	**3i,d**	-	-	-
50	**3i,e**	-	-	-
36	**3j,a**	-	-	+[a]
37	**3j,b**	-	-	+[a]
38	**3j,c**	-	-	+[a]
39	**3j,d**	-	-	-
40	**3j,e**	-	-	-

Summary of 55 reactions with heteroaromatic and aromatic esters performed with PestE, Est2, and SpL. Reaction conditions: ester (10 mM), amine (20 mM), 200 mM KPi buffer (pH 7.5), and 2 mg/mL CFE (PestE) or 10 mg/mL CFE (Est2), 30 °C, 1 h. Legend for amide formation:
- = not detected, = <10% (low), ++ = 10%-80% (middle), and +++ = >80% (high). 2e = piperidine, 1j = ethyl isonicotinate
[a] = amounts from rel. area (HPLC) [%]

Biotransformations – testing PestE-D156I & Est2-D155I variants

For the reaction mixtures, 0.4 mg/mL of purified enzyme (stock in 50 mM KPi, pH 8 or 11.7 for PestE D156I) was mixed with a specific amount of 50 mM KPi buffer (pH 8) to have a final volume of 1 mL. In this 1 mL, 20 µL of 1 M benzylamine DMSO stock (20 mM) and 12 µL 2 M HCl were included, too. To start the reaction, 10 µL of 1 M ester (ethyl picolinate or ethyl furoate) DMSO stock (10 mM) were added. Final DMSO concentration was 3%. The reaction mixtures were then incubated at 30 °C and 700 rpm for 15 min. Samples were measured on aq. HPLC.

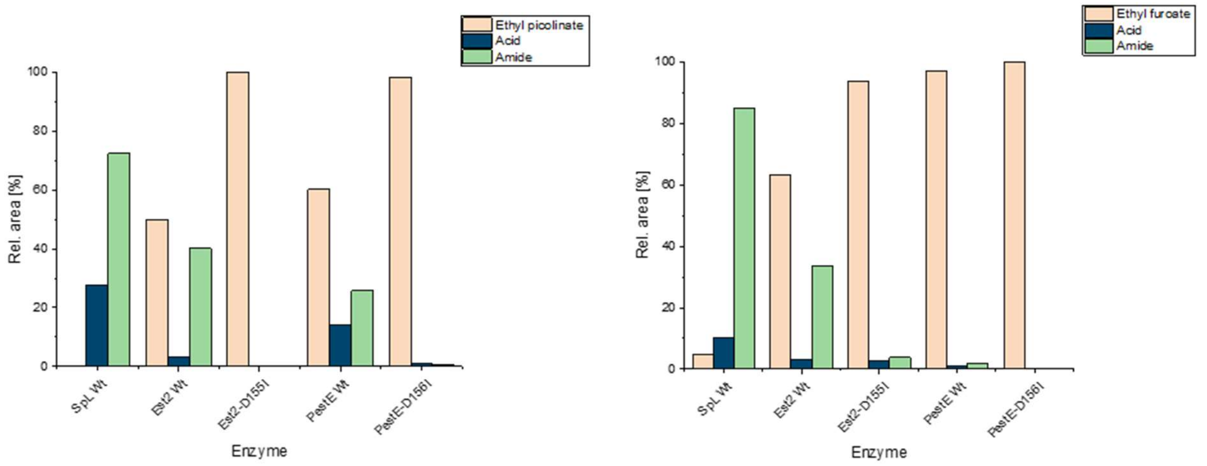

Figure S15: Influence of the exchange of the aspartate neighboring the canonical catalytically active serine in Est2 and PestE to isoleucine on amide formation. a. ethyl picolinate served as substrate ester. b. ethyl furoate was used as substrate. Reaction condition: 0.4 mg/mL his$_6$tag purified enzyme, ethyl ester (10 mM), benzylamine (20 mM), 3% DMSO, 200 mM KPi (pH 7.5), 30 °C, 800 rpm, 15 min. For comparison also the results for SpL are given. The composition was measured by HPLC at 280 nm.

PestE & Est2 – Structural comparison with SpL

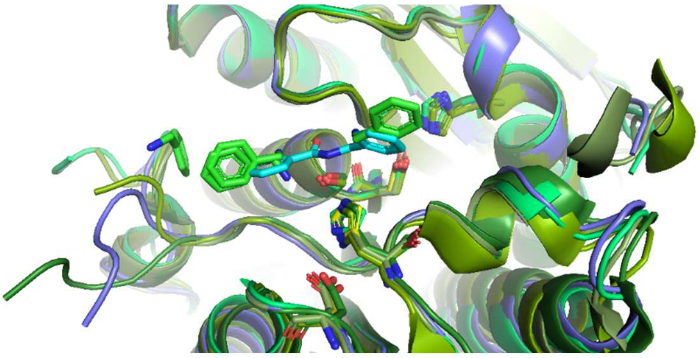

Figure S16: Overlay of the catalytic residues in the active site of Spl; Est2 and PestE with the position of the ligands shown as before. The catalytically important residues are located in the same positions in all enzymes with very small variations in side-chain rotations. SpL is depicted in slate, PestE and Est2 are colored in splitpea and forest, respectively, compare Figure 5 and Figure S17.

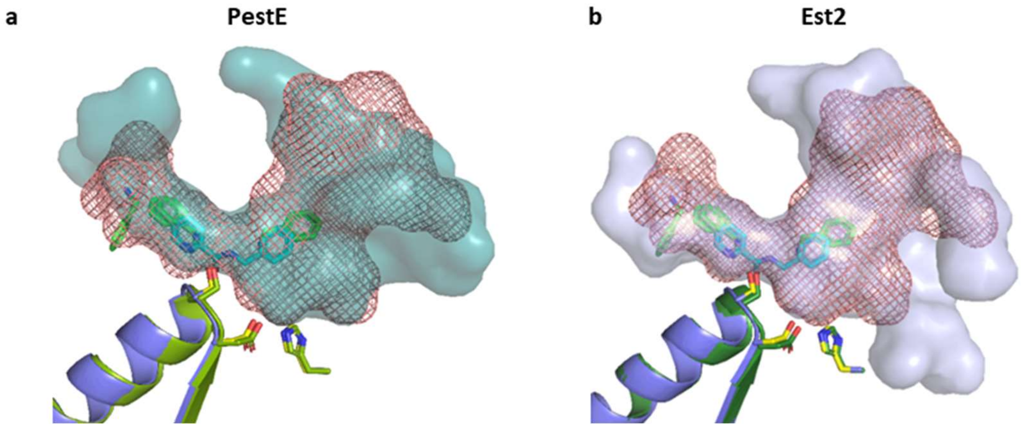

Figure S17: Overlay of the active site cavities of PestE and Est2 with SpL. a. PestE. b. Est2. The cavity of SpL is represented as a mesh. The active site has a similar shape in the aligned enzymes.

Synthesis of substrate

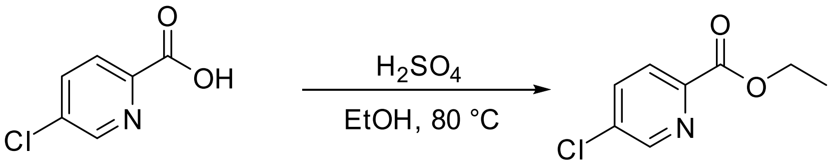

In a 50 mL round bottomed flask, commercially available 5-chloro-2-pyridinecarboxylic acid (2 g, 12.7 mmol) was mixed with EtOH (20 mL). After the addition of H_2SO_4 (0.2 mL, 1.1 mmol) and heating up to 80 °C, the suspension dissolved and a clear, yellow liquid was obtained. The solution was mixed for 9 h. A TLC control still showed the acid spot, but the reaction was stopped. The reaction solution was basified with $NaHCO_{3(sat.)}$ and extracted three times with EtOAc. The combined organic phases was dried over Na_2SO_4 and the solvent removed with reduced pressure to give the corresponding ester in 80.2% yield (10.2 mmol, 1.89 g). Spectral data in in agreement with literature. Lit.: DOI: 10.1021/acs.jmedchem.7b01795

Biotransformations – Preparative syntheses of amides

All syntheses were done in 200 mM KPi buffer at 30 °C. The amine was added first to the buffer, then the pH was adjusted to pH 7.5 using conc. aqueous HCl or 85% H_3PO_4. CFE was added for 5 min. Finally, the ester was added to start the reaction. Depending on the reaction vessel, a water bath was used and the solution was stirred with a magnetic stirrer or incubated in an incubator at 120 rpm.

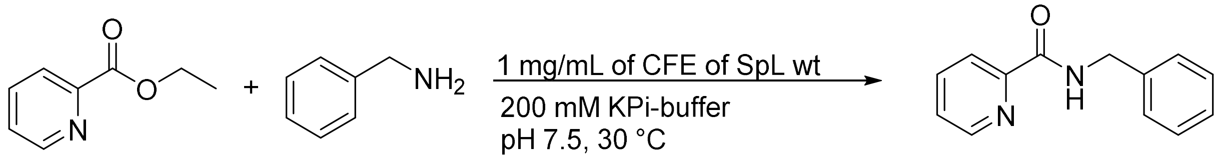

N-benzylpicolinamide

Synthesis of *N*-benzylpicolinamide 3a,a:

In a 100 mL round bottomed flask, benzylamine (1.36 g, 12.72 mmol) was dissolved in KPi buffer (200 mM, 60 mL at pH 7.5) and the pH was readjusted to 7.5 with the addition of 85% H_3PO_4. CFE of SpL was added (60 mg) and the mixture was stirred till a clear solution was observed. The water bath was adjusted to 30 °C and then ethyl-2-picolinate (0.56 g, 3.69 mmol) was added and stirred. After 20 min, no ester was observed by TLC and the reaction was quenched with conc. HCl to reach pH 4. The aqueous phase was extracted with ethyl acetate (3 x 50 mL). The combined organic phases were washed with 2 M KOH (2 x 50 mL) and washed with brine (1 x 50 mL). The organic phase was dried over Na_2SO_4. The Na_2SO_4 was filtered off and the organic phase evaporated under vacuum to give 710 mg (90%) product.
^{1}H NMR (300 MHz, Chloroform-*d*) δ 8.53 (d, *J* = 4.9 Hz, 1H), 8.38 (s, 1H), 8.24 (d, *J* = 7.8 Hz, 1H), 7.86 (td, *J* = 7.7 ,7.6 Hz, 1H), 7.47 – 7.22 (m, 6H), 4.68 (d, *J* = 6.1 Hz, 2H).
^{13}C NMR (75 MHz, Chloroform-*d*) δ 164.4, 150, 148.2, 138.3, 137.5, 128.8, 128, 127.6, 126.3, 122.5, 43.6.

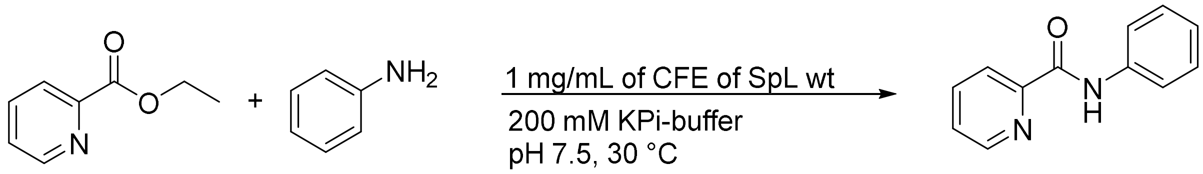

Synthesis of *N*-phenylpicolinamide 3a,b:

In a 100 mL round bottomed flask, aniline (1.29 g, 13.84 mmol) and KPi buffer (200 mM, 60 mL at pH 7.5) were mixed together. Afterwards, the pH was measured and adjusted to 7.5 with the addition of 85% H_3PO_4. At the pH 7.5, CFE of SpL was added (60 mg) and the mixture was stirred till a clear solution was observed. The water bath was adjusted to 30 °C and then ethyl-2-picolinate (0.59 g, 3.91 mmol) was added and stirred. After 20 min, no ester was observed on the TLC and the reaction was quenched with conc. HCl to reach pH 4. The aqueous phase was extracted with ethyl acetate (3 x 50 mL). The combined organic phases were washed with 2 M KOH (2 x 50 mL) and with brine (1 x 50 mL). The organic phase was dried over Na_2SO_4. The Na_2SO_4 was filtered off and the organic phase evaporated under vacuum to give 740 mg (96%) product.
^{1}H NMR (300 MHz, Chloroform-*d*) δ 10.03 (s, 1H), 8.71 – 8.57 (m, 1H), 8.37 – 8.23 (m, 1H), 7.91 (td, *J* = 1.4 Hz, 1H), 7.79 (d, *J* = 7.6 Hz, 2H), 7.52 – 7.45 (m, 1H), 7.39 (t, *J* = 8.0 Hz, 2H), 7.16 (t, J = 7.4, 1H).

^{13}C NMR (75 MHz, Chloroform-*d*) δ 162.1, 150, 148.1, 137.8, 129.2, 126.6, 124.5, 122.6, 119.8.

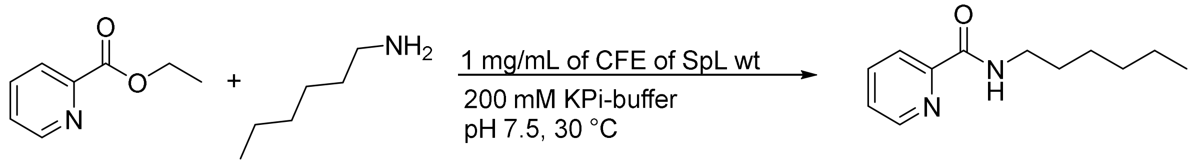

Synthesis of *N*-hexylpicolinamide 3a,c:

In a 100 mL round bottomed flask, *n*-hexylamine (1.6 g, 15.77 mmol) and KPi buffer (200 mM, 75 mL at pH 7.5) were mixed together. Afterwards, the pH was measured and adjusted to 7.5 with the addition of 85% H_3PO_4. At the pH 7.5, CFE of SpL was added (80 mg) and the mixture was stirred till a clear solution was observed. The water bath was adjusted to 30 °C and then ethyl-2-picolinate (0.56 g, 3.69 mmol) was added and stirred. After 60 min, no ester was observed on the TLC and the reaction was quenched with conc. HCl to reach pH 4. The aqueous phase was extracted with ethyl acetate (3 x 50 mL). The combined organic phases were washed with 2 M KOH (2 x 50 mL) and with brine (1 x 50 mL). The organic phase was dried over Na_2SO_4. The Na_2SO_4 was filtered off and the organic phase evaporated under vacuum to give 450 mg (48%) product.
^{1}H NMR (300 MHz, Chloroform-*d*) δ 8.54 (d, 1H), 8.20 (d, *J* = 7.8 Hz, 1H), 8.05 (s, 1H), 7.84 (td, *J* = 7.7, 7.6 Hz, 1H), 7.43 – 7.39 (m, 1H), 3.46 (q, *J* = 6.8 Hz, 2H), 1.72 – 1.53 (m, 2H), 1.47 – 1.21 (m, 6H), 0.89 (t, *J* = 6.3 Hz, 3H).
^{13}C NMR (75 MHz, Chloroform-*d*) δ 164.3, 150.2, 148.1, 137.5, 126.1, 122.3, 39.6, 31.7, 29.8, 26.8, 22.7, 14.2.

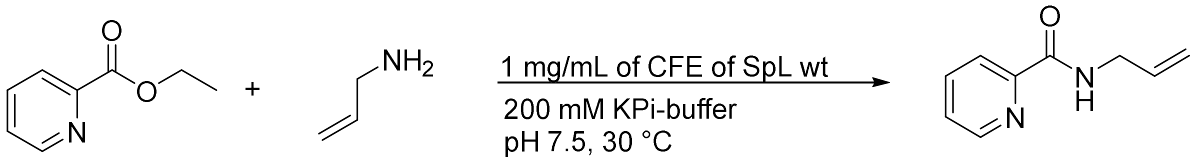

Synthesis of *N*-allylpicolinamide 3a,d:

In a 250 mL round bottomed flask, allylamine (1.94 g, 33.91 mmol) and KPi buffer (200 mM, 160 mL at pH 7.5) were mixed together. Afterwards, the pH was measured and adjusted to 7.5 with the addition of 85% H_3PO_4. At the pH 7.5, CFE of SpL was added (60 mg) and the mixture was stirred till a clear solution was observed. The water bath was adjusted to 30 °C and then ethyl-2-picolinate (1.46 g, 9.69 mmol) was added and stirred. Although the ester was still not completely converted after 30 min, the reaction was quenched with conc. HCl to reach pH 4 due to a significant amount of produced acid on the TLC. The aqueous phase was extracted with ethyl acetate (3 x 80 mL). The combined organic phases were washed with 2 M KOH (2 x 60 mL) and with brine (1 x 70 mL). The organic phase was dried over Na_2SO_4. The Na_2SO_4 was filtered off and the organic phase evaporated under vacuum to give 250 mg (16%) product.
^{1}H NMR (300 MHz, Chloroform-*d*) δ 8.55 (d, *J* = 4.1 Hz, 1H), 8.20 (dt, *J* = 1, 1.1 Hz, 1H), 8.15 (s, 1H), 7.85 (td, *J* = 1.7, 1.6, 1.5 Hz, 1H), 7.48 – 7.36 (m, 1H), 6.04 – 5.85 (m, 1H), 5.34 – 5.12 (m, 2H), 4.16 – 4.05 (m, 2H).
^{13}C NMR (75 MHz, Chloroform-*d*) δ 164.3, 150, 148.2, 137.5, 134.2, 126.3, 122.4, 116.6, 41.9.

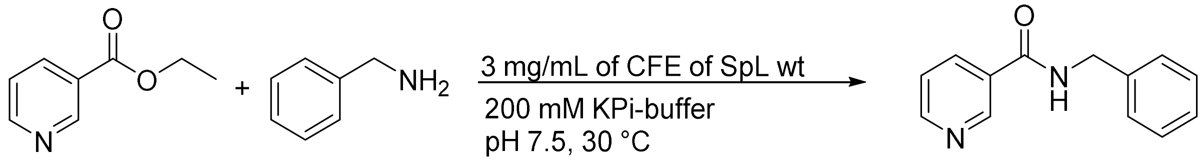

Synthesis of *N*-benzylnicotinamide 3b,a:

In a 25 mL round bottomed flask, benzylamine (0.15 g, 5.1 mmol) and KPi buffer (200 mM, 16 mL at pH 7.5) were mixed together. Afterwards, the pH was measured and adjusted to 7.5 with the addition of con. HCl. At the pH 7.5, CFE of SpL was added (80 mg) and the mixture was stirred until the CFE was dissolved. The water bath was adjusted to 30 °C and then ethyl nicotinate (0.55 g, 0.97 mmol) was added and stirred. After 33 min, the reaction was stopped by extraction with ethyl acetate (3 x 8 mL). The combined organic phases were washed with 2 M KOH (1 x 5 mL) and with brine (1 x 8 mL). The organic phase was dried over Na_2SO_4. The Na_2SO_4 was filtered off and the organic phase was evaporated under vacuum. The crude was purified via a column pasteur pipette using first the eluent mix 50% cyclohexane and 50% ethyl acetate to remove the impurities. The product was eluted with ethyl acetate. The organic phase was evaporated under vacuum to give 68 mg (33%) product.

^{1}H NMR (300 MHz, Chloroform-*d*) δ 8.95 (d, *J* = 2.3 Hz, 1H), 8.68 (dd, *J* = 1.4, 1.6 Hz, 1H), 8.12 (dt, *J* = 1.7, 1.8 Hz, 1H), 7.39 – 7.27 (m, 6H), 6.74 (s, 1H), 4.64 (d, *J* = 5.6 Hz, 2H).

^{13}C NMR (75 MHz, Chloroform-*d*) δ 165.6, 152.4, 148, 137.9, 135.3, 130.2, 129, 128.1, 127.9, 123.7, 44.3.

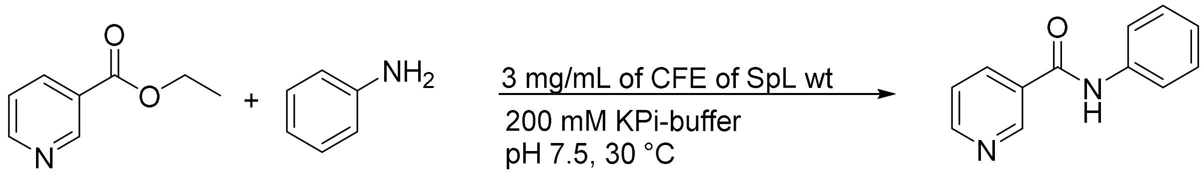

Synthesis of *N*-phenylnicotinamide 3b,b:

In a 14 mL conical plastic tube, aniline (0.195 g, 2.09 mmol) and KPi buffer (200 mM, 10 mL at pH 7.5) were mixed together. Afterwards, the pH was measured and adjusted to 7.5 with the addition of conc. HCl. At the pH 7.5, CFE of SpL was added (30 mg) and the mixture was mixed until the CFE was dissolved. Then ethyl nicotinate (0.09 g, 0.6 mmol) was added and incubated at 30 °C, 120 rpm. After 26 h, the reaction was stopped by extraction with ethyl acetate (3 x 5 mL). The combined organic phases were washed with 2 M KOH (1 x 5 mL) and with brine (1 x 5 mL). The organic phase was dried over Na_2SO_4. The Na_2SO_4 was filtered off and the organic phase evaporated under vacuum. The crude was purified by column chromatography with a biotage using a gradient with the eluents cyclohexane and ethyl acetate (stationary phase: silica, flow: 5 mL/min, 4-15% 2 CV, 15-100% 8 CV, 100% 9 CV). The product was in eluted with ethyl acetate. The organic phase was evaporated under vacuum to give 33 mg (27%) product.

^{1}H NMR (300 MHz, Chloroform-*d*) δ 9.07 (d, *J* = 2.1 Hz, 1H), 8.73 (dd, *J* = 4.8 Hz, 1.7 Hz, 1H), 8.23 (s, 1H), 8.26 – 8.14 (m, 2H), 7.68 – 7.59 (m, 2H), 7.46 – 7.30 (m, 3H), 7.23 – 7.11 (m, 1H).

^{13}C NMR (75 MHz, Chloroform-*d*) δ 164.1, 152.6, 148, 137.6, 135.5, 131, 129.3, 125.2, 123.8, 120.7.

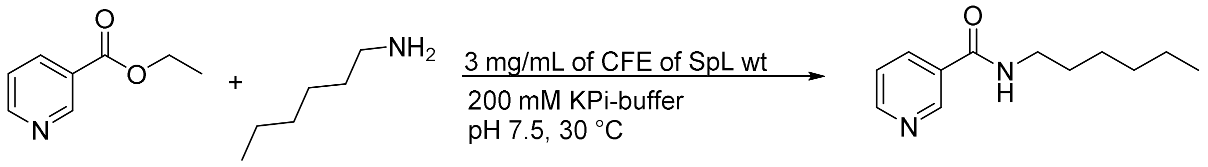

Synthesis of *N*-hexylnicotinamide 3b,c:

In a 50 mL round bottomed flask, *n*-hexylamine (0.35 g, 3.43 mmol) and KPi buffer (200 mM, 25 mL at pH 7.5) were mixed together. Afterwards, the pH was measured and adjusted to 7.5 with the addition of con. HCl. At the pH 7.5, CFE of SpL was added (75 mg) and the mixture was stirred for 3 min. The water bath was adjusted to 30 °C and then ethyl-2-picolinate (0.15 g, 0.98 mmol) was added and stirred. After 45 min, the reaction was stopped by extraction with ethyl acetate (3 x 15 mL). The combined organic phases were washed with 2 M KOH (1 x 15 mL) and with brine (1 x 15 mL). The organic phase was dried over Na_2SO_4. The Na_2SO_4 was filtered off and the organic phase evaporated under vacuum. The crude was purified via a column chromatography using first the eluent mixture 50% cyclohexane and 50% ethyl acetate to remove the impurities. The product was finally eluted with ethyl acetate. The organic phase was evaporated under vacuum to give 3 mg (1%) product.
^{1}H NMR (300 MHz, Chloroform-*d*) δ 8.95 (d, *J* = 2.2 Hz, 1H), 8.72 (dd, *J* = 4.9, 1.7 Hz, 1H), 8.11 (dt, *J* = 1.7, 1.8 Hz, 1H), 7.39 (dd, *J* = 4.9, 4.8 Hz, 1H), 6.14 (s, 1H), 3.47 (q, *J* = 7.1 Hz, 2H), 1.64 (d, *J* = 8.6 Hz, 2H), 1.40 – 1.27 (m, 6H), 0.90 (t, *J* = 6.7 Hz, 3H).
^{13}C NMR (75 MHz, Chloroform-*d*) δ 165.7, 152.1, 147.8, 135.3, 123.7, 40.4, 31.6, 29.7, 26.8 22.7, 14.1.

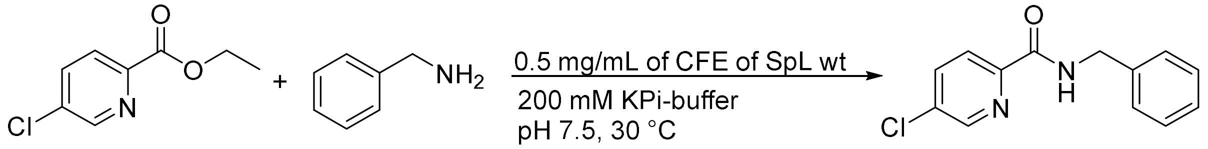

Synthesis of 5-chloro-*N*-benzylamide 3c,a:

In a 25 mL round bottomed flask, benzylamine (0.28 g, 2.66 mmol) and KPi buffer (200 mM, 20 mL at pH 7.5) were mixed together. Afterwards, the pH was measured and adjusted to 7.5 with the addition of 85% H_3PO_4 and 1 M NaOH. At the pH 7.5, CFE of SpL was added (10 mg) and the mixture was stirred till a clear solution was observed. The water bath was adjusted to 30 °C and then ethyl-5-chloropicolinate (0.14 g, 0.76 mmol) was added and stirred. After 90 min, the reaction was quenched with conc. HCl to reach pH 4. The aqueous phase was extracted with ethyl acetate (3 x 5 mL). The combined organic phases were washed with 2 M KOH (3 x 5 mL) and with brine (2 x 5 mL). The organic phase was dried over Na_2SO_4. The Na_2SO_4 was filtered off and the organic phase evaporated under vacuum to give 164 mg (88%) product.
^{1}H NMR (300 MHz, Chloroform-*d*) δ 8.47 (d, *J* = 2.4 Hz, 1H), 8.22 (s, 1H), 8.19 (d, *J* = 8.3 Hz, 1H), 7.83 (dd, *J* = 2.3, 2.4 Hz, 1H), 7.41 – 7.27 (m, 5H), 4.66 (d, *J* = 6.1 Hz, 2H).
^{13}C NMR (75 MHz, Chloroform-*d*) δ 163.5, 148.1, 147.2, 138.1, 137.2, 135.2, 128.9, 128, 127.7, 123.5, 43.7.

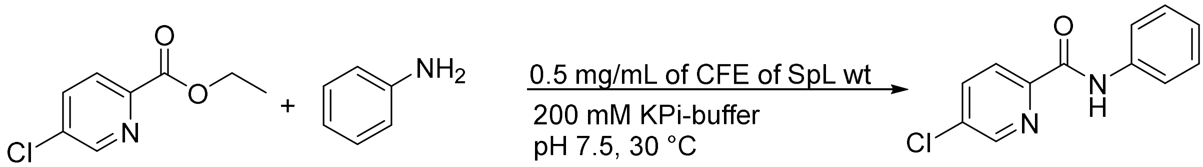

Synthesis of 5-chloro-*N*-phenylpicolinamide 3c,b:

In a 25 mL round bottomed flask, aniline (0.20 g, 2.14 mmol) and KPi buffer (200 mM, 20 mL at pH 7.5) were mixed together. Afterwards, the pH was measured and adjusted to 7.5 with the addition of 85% H_3PO_4. At the pH 7.5, CFE of SpL was added (10 mg) and the mixture was stirred till a clear solution was observed. The water bath was adjusted to 30 °C and then ethyl-5-chloropicolinate (0.11 g, 0.59 mmol) was added and stirred. After 35 min, only a faint ester spot was observed on the TLC and due to progressive increase of the acid spot, the reaction was quenched with conc. HCl to reach pH 4. 5 mL brine was added to the solution. The aqueous phase was extracted ethyl acetate (3 x 5 mL). The combined organic phases were washed with 2 M KOH (3 x 10 mL) and with brine (1 x 10 mL). The organic phase was dried over Na_2SO_4. The Na_2SO_4 was filtered off and the organic phase evaporated under vacuum to give 140 mg (102%) product.
^{1}H NMR (300 MHz, Chloroform-*d*) δ 9.85 (s, 1H), 8.59 (d, *J* = 1.8 Hz, 1H), 8.28 (d, *J* = 8.4 Hz, 1H), 7.90 (dd, *J* = 2.4, 2.4 Hz, 1H), 7.78 (d, *J* = 7.4 Hz, 2H), 7.41 (t, *J* = 7.8 Hz, 2H), 7.18 (t, *J* = 7.4 Hz, 2H).
^{13}C NMR (75 MHz, Chloroform-*d*) δ 164.4, 150, 148.2, 138.4, 137.6, 128.9, 128, 127.6, 126.4, 122.5, 43.6.

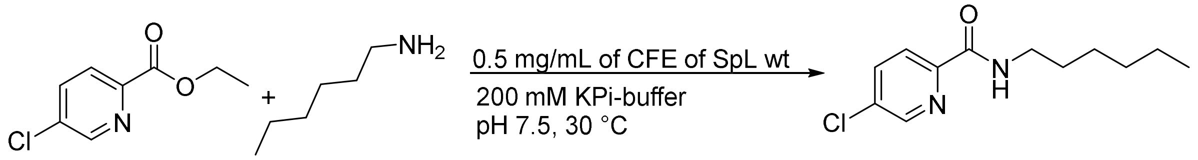

Synthesis of 5-chloro-*N*-hexylamide 3c,c:

In a 25 mL round bottomed flask, *n*-hexylamine (0.39 g, 3.85 mmol) and KPi buffer (200 mM, 20 mL at pH 7.5) were mixed together. Afterwards, the pH was measured and adjusted to 7.5 with the addition of 85% H_3PO_4 and 1 M NaOH. At the pH 7.5, CFE of SpL was added (10 mg) and the mixture was stirred till a clear solution was observed. The water bath was adjusted to 30 °C and then ethyl-5-chloropicolinate (0.21 g, 1.11 mmol) was added and stirred. After 90 min, the reaction was quenched with conc. HCl to reach pH 4. The aqueous phase was extracted with ethyl acetate (3 x 5 mL). The combined organic phases were washed with 2 M KOH (3 x 5 mL) and with brine (2 x 5 mL). The organic phase was dried over Na_2SO_4. The Na_2SO_4 was filtered off and the organic phase evaporated under vacuum to give 178 mg (67%) product.
^{1}H NMR (300 MHz, Chloroform-*d*) δ 8.48 (d, *J* = 2.4 Hz, 1H), 8.15 (d, *J* = 8.3 Hz, 1H), 7.90 (s, 1H), 7.80 (dd, *J* = 2.3, 2.4 Hz, 1H), 3.45 (q, *J* = 6.8 Hz, 2H), 1.67 – 1.56 (m, 2H), 1.40 – 1.24 (m, 6H), 0.88 (t, *J* = 6.7 Hz, 4H).
^{13}C NMR (75 MHz, Chloroform-*d*) δ 163.5, 148.4, 147.1, 137.2, 134.9, 123.3, 39.7, 31.6, 29.7, 26.8, 22.7, 14.2.

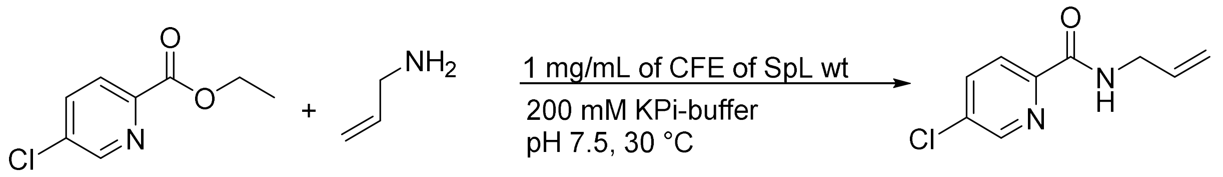

Synthesis of 5-chloro-*N*-allylamide 3c,d:

In a 50 mL round bottomed flask, allylamine (0.30 g, 5.31 mmol) and KPi buffer (200 mM, 20 mL at pH 7.5) were mixed together. Afterwards, the pH was measured and adjusted to 7.5 with the addition of conc. HCl. At the pH 7.5, CFE of SpL was added (20 mg) and the mixture was stirred for 5 min. The water bath was adjusted to 30 °C and then ethyl-5-chloropicolinate (0.28 g, 1.52 mmol) was added and stirred. After 100 min, the reaction was quenched with conc. HCl to reach pH 4. The aqueous phase was extracted ethyl acetate (3 x 10 mL). The combined organic phases were washed with 2 M KOH (2 x 5 mL) and with brine (1 x 5 mL). The organic phase was dried over Na_2SO_4. The Na_2SO_4 was filtered off and the organic phase evaporated under vacuum. The crude was purified by column chromatography with a biotage using a gradient with the eluents cyclohexane and ethyl acetate (stationary phase: silica, flow: 5 mL/min, 4-15% 2 CV, 15-100% 8 CV, 100% 9 CV). The organic phase was evaporated under vacuum to give 37 mg (12%) product.

^{1}H NMR (300 MHz, Chloroform-*d*) δ 8.49 (d, *J* = 1.7 Hz, 1H), 8.16 (d, *J* = 8.4 Hz, 1H), 7.99 (s, 1H), 7.82 (dd, *J* = 2.4, 2.4 Hz, 1H), 6.03 – 5.83 (m, 1H), 5.32 – 5.13 (m, 2H), 4.10 (tt, *J* = 1.5, 1.3 Hz, 2H).

^{13}C NMR (75 MHz, Chloroform-*d*) δ 163.4, 148.1, 147.3, 137.2, 135.1, 134, 123.4z, 116.7, 42.

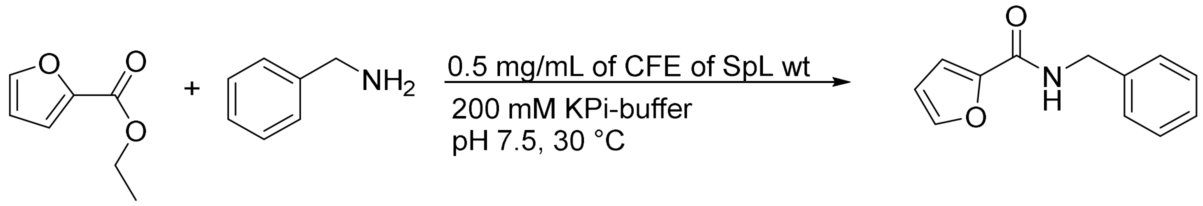

Synthesis of *N*-benzylfuran-2-carboxamide 3e,a:

In a 25 mL round bottomed flask, benzylamine (0.29 g, 2.75 mmol) and KPi buffer (200 mM, 20 mL at pH 7.5) were mixed together. Afterwards, the pH was measured and adjusted to 7.5 with the addition of 85% H_3PO_4. At the pH 7.5, CFE of SpL was added (10 mg) and the mixture was stirred till a clear solution was observed. The water bath was adjusted to 30 °C and then ethyl-2-furoate (0.11 g, 0.75 mmol) was added and stirred. After 30 min, only a faint ester spot was observed on the TLC and due to progressive increase of the acid spot, the reaction was quenched with conc. HCl to reach pH 4. 5 mL brine was added to the solution. The aqueous phase was extracted ethyl acetate (3 x 5 mL). The combined organic phases were washed with 2 M KOH (3 x 10 mL) and with brine (1 x 10 mL). The organic phase was dried over Na_2SO_4. The Na_2SO_4 was filtered off and the organic phase evaporated under vacuum to give 140 mg (89%) product.

^{1}H NMR (300 MHz, Chloroform-*d*) δ 7.58 – 7.25 (m, 7H), 6.77 (s, 1H), 6.62 (s, 1H), 4.73 (d, *J* = 5.2 Hz, 2H).

^{13}C NMR (75 MHz, Chloroform-*d*) δ 158.4, 148, 144, 138.1, 128.9, 128, 127.8, 114.5, 112.3, 43.3.

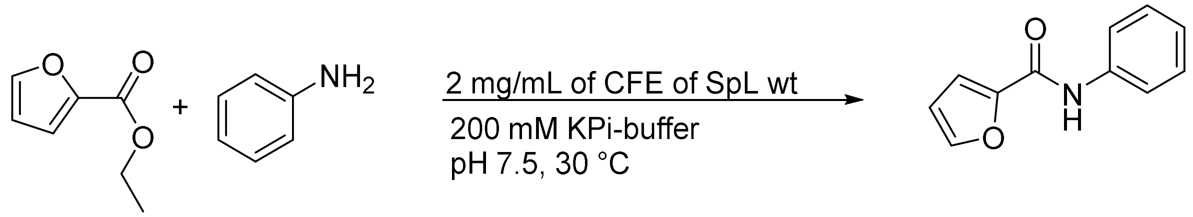

Synthesis of furan-2-carboxylic acid phenylamide 3e,b:

In a 14 mL conical plastic tube, aniline (0.28 g, 3.02 mmol) and KPi buffer (200 mM, 14 mL at pH 7.5) were mixed together. Afterwards, the pH was measured and adjusted to 7.5 with the addition of conc. HCl. At the pH 7.5, CFE of SpL was added (28 mg) and mixed until the CFE was dissolved. Then ethyl-2-furoate (0.12 g, 0.86 mmol) was added and everything was incubated at 30 °C, 120 rpm. After 4.5 h, the reaction was quenched with conc. HCl to reach pH 4. The aqueous phase was extracted ethyl acetate (3 x 8 mL). The combined organic phases were washed with 2 M KOH (1 x 5 mL). The organic phase was dried over Na_2SO_4. The Na_2SO_4 was filtered off and the organic phase evaporated under vacuum to give 140 mg (87%) product.
^{1}H NMR (300 MHz, Chloroform-*d*) δ 8.08 (s, 1H), 7.65 (d, *J* = 7.6 Hz, 2H), 7.55 – 7.48 (m, 1H), 7.37 (t, *J* = 7.9 Hz, 2H), 7.24 (d, *J* = 3.6 Hz, 1H), 7.15 (t, *J* = 7.4 Hz, 1H), 6.56 (dd, *J* = 1.8, 1.7 Hz, 1H).
^{13}C NMR (75 MHz, Chloroform-*d*) δ 156.2, 148, 144.3, 137.5, 129.3, 124.7, 120, 115.4, 112.8.

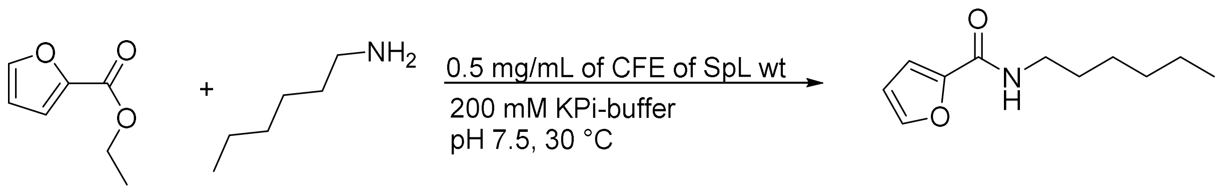

Synthesis of furan-2-carboxylic acid hexylamide 3e,c:

In a 25 mL round bottomed flask, *n*-hexylamine (0.36 g, 3.53 mmol) and KPi buffer (200 mM, 20 mL at pH 7.5) were mixed together. Afterwards, the pH was measured and adjusted to 7.5 with the addition of 85% H_3PO_4 and 1 M NaOH. At the pH 7.5, CFE of SpL was added (10 mg) and the mixture was stirred till a clear solution was observed. The water bath was adjusted to 30 °C and then ethyl-2-furoate (0.14 g, 1.01 mmol) was added and stirred. After 85 min, the reaction was quenched with conc. HCl to reach pH 4. The aqueous phase was extracted with ethyl acetate (3 x 10 mL). The combined organic phases were washed with 2 M KOH (3 x 10 mL) and with brine (2 x 10 mL). The organic phase was dried over Na_2SO_4. The Na_2SO_4 was filtered off and the organic phase evaporated under vacuum. The crude was purified by column chromatography with a biotage using a gradient with the eluents cyclohexane and ethyl acetate (stationary phase: silica, flow: 5 mL/min, 4-15% 2 CV, 15-100% 8 CV, 100% 9 CV). The organic phase evaporated under vacuum to give 54 mg (28%) product.
^{1}H NMR (300 MHz, Chloroform-*d*) δ 7.45 – 7.37 (m, 1H), 7.09 (dd, *J* = 0.7, 0.8 Hz, 1H), 6.51 – 6.45 (m, 1H), 6.36 (s, 1H), 3.41 (m, 2H), 1.65 – 1.53 (m, 2H), 1.42 – 1.23 (m, 6H), 0.88 (t, *J* = 6.6 Hz, 3H).
^{13}C NMR (75 MHz, Chloroform-*d*) δ 158.5, 148.4, 143.8, 114, 112.2, 39.3, 31.6, 29.8, 26.7, 22.7, 14.1.

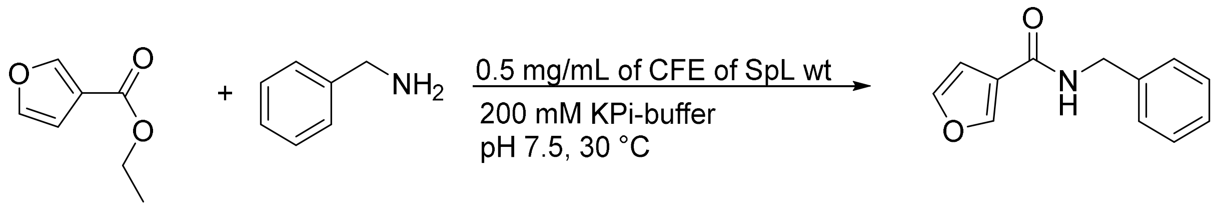

Synthesis of *N*-benzylfuran-3-carboxamide 3f,a:

In a 25 mL round bottomed flask, benzylamine (0.30 g, 2.75 mmol) and KPi buffer (200 mM, 20 mL at pH 7.5) were mixed together. Afterwards, the pH was measured and adjusted to 7.5 with the addition of 85% H_3PO_4. At the pH 7.5, CFE of SpL was added (10 mg) and the mixture was stirred till a clear solution was observed. The water bath was adjusted to 30 °C and then ethyl-3-furoate (0.14 g, 1.03 mmol) was added and stirred. After 35 min, no ester spot was observed on the TLC and the reaction was quenched with conc. HCl to reach pH 4. The aqueous phase was extracted ethyl acetate (3 x 5 mL). The combined organic phases were washed with 2 M KOH (3 x 10 mL) and with brine (1 x 10 mL). The organic phase was dried over Na_2SO_4. The Na_2SO_4 was filtered off and the organic phase evaporated under vacuum to give 130 mg (65%) product.
^{1}H NMR (300 MHz, Chloroform-*d*) δ 7.94 (s, 1H), 7.45 – 7.26 (m, 7H), 6.61 (s, 1H), 4.58 (d, *J* = 5.8 Hz, 2H).
^{13}C NMR (75 MHz, Chloroform-*d*) δ 162.6, 145, 143.9, 138.2, 128.9, 128.1, 127.8, 122.5, 108.3, 43.7.

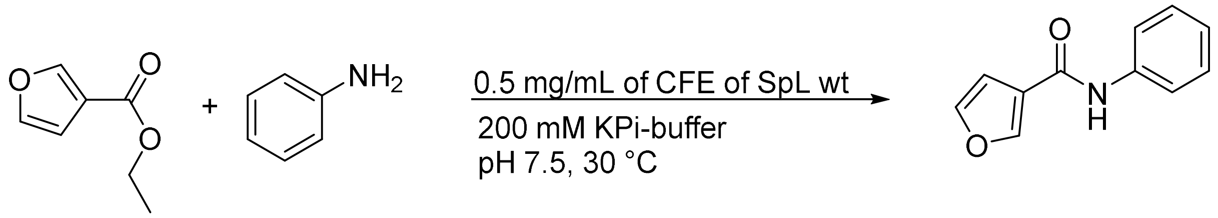

Synthesis of *N*-phenyl-3-furancarboxamide 3f,b:

In a 25 mL conical flask, aniline (0.45 g, 4.81 mmol) and KPi buffer (200 mM, 20 mL at pH 7.5) were mixed together. Afterwards, the pH was measured and adjusted to 7.5 with the addition of 85% H_3PO_4 and 1 M NaOH. At the pH 7.5, CFE of SpL was added (150 mg) and the mixture was stirred until the CFE was dissolved. The water bath was adjusted to 30 °C and then the ethyl-3-furoate (0.20 g, 1.39 mmol) was added and stirred. After 190 min, the reaction was quenched with conc. HCl to reach pH 4. The aqueous phase was extracted ethyl acetate (3 x 10 mL). The combined organic phases were washed with 2 M KOH (3 x 10 mL) and with brine (2 x 20 mL). The organic phase was dried over Na_2SO_4. The Na_2SO_4 was filtered off and the organic phase evaporated under vacuum. The crude was purified by column chromatography with a biotage using a gradient with the eluents cyclohexane and ethyl acetate (stationary phase: silica, flow: 5 mL/min, 4-15% 2 CV, 15-100% 8 CV, 100% 9 CV). The organic phase evaporated under vacuum to give 47 mg (18%) product.
^{1}H NMR (300 MHz, Chloroform-*d*) δ 8.03 (t, *J* = 1.2 Hz, 1H), 7.68 – 7.55 (m, 3H), 7.47 (t, *J* = 1.8 Hz, 1H), 7.39 – 7.29 (m, 2H), 7.18 – 7.09 (m, 1H), 6.73 (dd, *J* = 0.8, 0.8 Hz, 1H).
^{13}C NMR (75 MHz, Chloroform-*d*) δ 160.9, 145.3, 144.2, 137.7, 129.2, 124.8, 123.2, 120.5, 108.5.

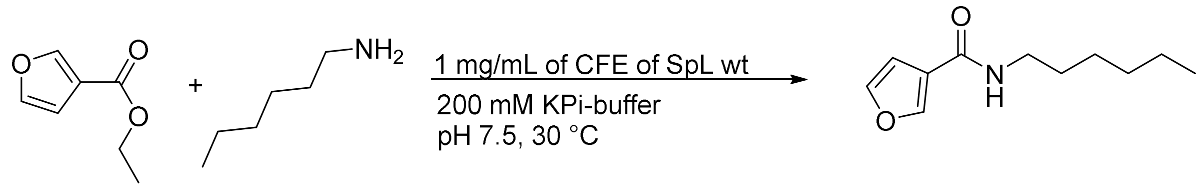

Synthesis of *N*-hexyl-3-furancarboxamide 3f,c:

In a 25 mL round bottomed flask, *n*-hexylamine (0.90 g, 8.86 mmol) and KPi buffer (200 mM, 35 mL at pH 7.5) were mixed together. Afterwards, the pH was measured and adjusted to 7.5 with the addition of conc. HCl. At the pH 7.5, CFE of SpL was added (10 mg) and the mixture was stirred until the CFE was dissolved. The water bath was adjusted to 30 °C and then ethyl-3-furoate (0.35 g, 2.53 mmol) was added and stirred. After 2 h, the reaction was quenched with conc. HCl to reach pH 4. The aqueous phase was extracted with ethyl acetate (3 x 20 mL). The combined organic phases were washed with 2 M KOH (1 x 15 mL) and with brine (1 x 20 mL). The organic phase was dried over Na_2SO_4. The Na_2SO_4 was filtered off and the organic phase evaporated under vacuum. The crude was purified by column chromatography with a biotage using a gradient with the eluents cyclohexane and ethyl acetate (stationary phase: silica, flow: 5 mL/min, 4-15% 2 CV, 15-100% 8 CV, 100% 9 CV).
^{1}H NMR (300 MHz, Chloroform-*d*) δ 7.94 – 7.89 (m, 1H), 7.42 (t, *J* = 1.8 Hz, 1H), 6.60 (dd, *J* = 0.8, 0.8 Hz, 1H), 5.84 (s, 1H), 3.38 (m, 2H), 1.56 (m, 2H), 1.32 (m, 6H), 0.89 (t, *J* = 6.6, 3H).
^{13}C NMR (75 MHz, Chloroform-*d*) δ 162.7, 144.6, 143.8, 122.9, 108.4, 39.7, 31.6, 29.8, 26.8, 22.7, 14.1.

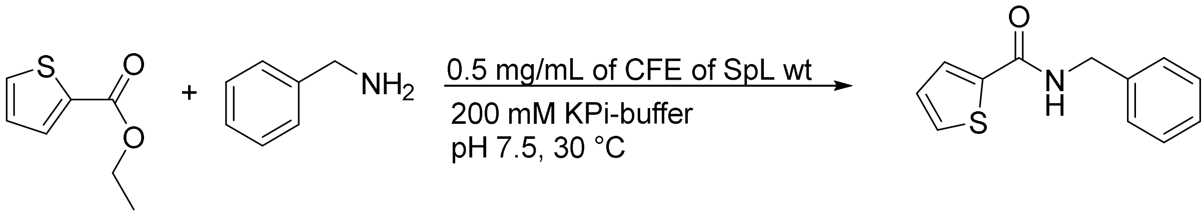

Synthesis of *N*-benzylthiophene-2-carboxamide 3g,a:

In a 25 mL round bottomed flask, benzylamine (0.33 g, 3.07 mmol) and KPi buffer (200 mM, 20 mL at pH 7.5) were mixed together. Afterwards, the pH was measured and adjusted to 7.5 with the addition of 85% H3PO4. At the pH 7.5, CFE of SpL was added (10 mg) and the mixture was stirred till a clear solution was observed. The water bath was adjusted to 30 °C and then ethyl thiophene-2-carboxylate (0.14 g, 0.87 mmol) was added and stirred. After 70 min, only a faint ester spot was observed on the TLC and due to increasing acid spot, the reaction was quenched with conc. HCl to reach pH 4. 5 mL brine was added to the solution. The aqueous phase was extracted ethyl acetate (3 x 5 mL). The combined organic phases were washed with 2 M KOH (3 x 10 mL) and with brine (1 x 10 mL). The organic phase was dried over Na_2SO_4. The Na_2SO_4 was filtered off and the organic phase was evaporated under reduced pressure to give 100 mg (54%) product.
^{1}H NMR (300 MHz, Chloroform-*d*) δ 7.53 – 7.46 (m, 2H), 7.39 – 7.27 (m, 5H), 7.07 (dd, *J* = 3.8, 3.8 Hz, 1H), 6.31 (s, 1H), 4.62 (d, *J* = 5.7 Hz, 2H).
^{13}C NMR (75 MHz, Chloroform-*d*) δ 161.9, 138.9, 138.1, 130.2, 128.9, 128.3, 128.1, 127.8, 127.8, 44.2.

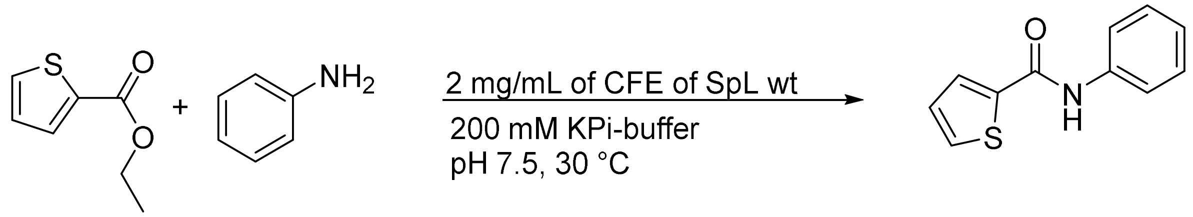

Synthesis of *N*-phenylthiophene-2-carboxamide 3g,b:

In a 25 mL round bottomed flask, aniline (0.22 g, 2.37 mmol) and KPi buffer (200 mM, 10 mL at pH 7.5) were mixed together. Afterwards, the pH was measured and adjusted to 7.5 with the addition of conc. HCl. At the pH 7.5, CFE of SpL was added (20 mg) and the mixture was stirred until the CFE was dissolved. Then, ethyl thiophene-2-carboxylate (0.11 g, 0.68 mmol) was added and stirred. The water bath was adjusted to 30 °C. After 20 h, no ester spot was observed on the TLC and the reaction was quenched with conc. HCl to reach pH 4. The aqueous phase was extracted ethyl acetate (3 x 5 mL). The combined organic phases were washed with 2 M KOH (1 x 5 mL). The organic phase was dried over Na_2SO_4. The Na_2SO_4 was filtered off and the organic phase evaporated under vacuum. The crude was purified by column chromatography with a biotage using a gradient with the eluents cyclohexane and ethyl acetate (stationary phase: silica, flow: 5 mL/min, 4-15% 2 CV, 15-100% 8 CV, 100% 9 CV). The organic phase evaporated under vacuum to give 63 mg (46%) product.
^{1}H NMR (300 MHz, Chloroform-*d*) δ 7.79 (s, 1H), 7.66 – 7.59 (m, 3H), 7.54 (dd, *J* = 1.1, 1.1 Hz, 1H), 7.41 – 7.31 (m, 2H), 7.19 – 7.09 (m, 2H).
^{13}C NMR (75 MHz, Chloroform-*d*) δ 160, 139.4, 137.7, 130.9, 129.2, 128.6, 128, 124.8, 120.4

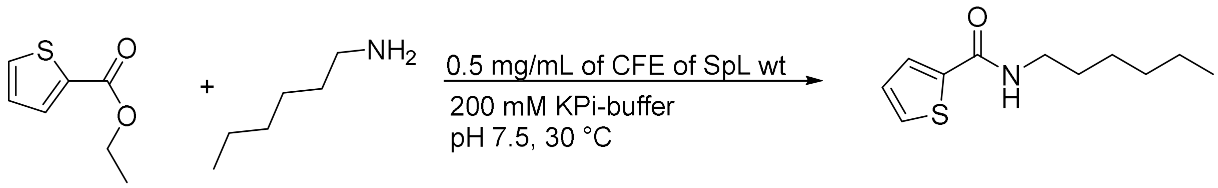

Synthesis of *N*-hexylthiophene-2-carboxamide 3g,c:

In a 25 mL round bottomed flask, *n*-hexylamine (0.40 g, 3,97 mmol) and KPi buffer (200 mM, 20 mL at pH 7.5) were mixed together. Afterwards, the pH was measured and adjusted to 7.5 with the addition of 85% H3PO4. At the pH 7.5, CFE of SpL was added (10 mg) and the mixture was stirred till a clear solution was observed. The water bath was adjusted to 30 °C and then ethyl thiophene-2-carboxylate (0.18 g, 1.13 mmol) was added and stirred. After 150 min, no ester spot was observed on the TLC and the reaction was quenched with conc. HCl to reach pH 4. 5 mL brine was added to the solution. The aqueous phase was extracted ethyl acetate (3 x 5 mL). The combined organic phases were washed with 2 M KOH (3 x 10 mL) and with brine (1 x 10 mL). The organic phase was dried over Na_2SO_4. The Na_2SO_4 was filtered off and the organic phase evaporated under vacuum to give 70 mg (28%) product.
^{1}H NMR (300 MHz, Chloroform-*d*) δ 7.47 (dd, *J* = 3.4, 4.8 Hz, 2H), 7.06 (t, *J* = 4.2, 1H), 6.01 (s, 1H), 3.42 (q, *J* = 7.1 Hz, 2H), 1.67 – 1.53 (m, 2H), 1.38 – 1.25 (m, 7H), 0.88 (d, *J* = 6.6 Hz, 2H).
^{13}C NMR (75 MHz, Chloroform-*d*) δ 162, 139.3, 129.7, 127.9, 127.7, 40.2, 31.6, 29.8, 26.8, 22.7, 14.2.

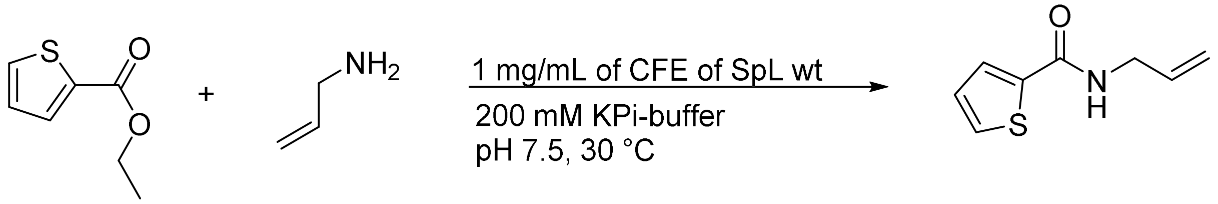

Synthesis of *N*-allylthiophene-2-carboxamide 3g,d:

In a 25 mL round bottomed flask, allylamine (1.01 g, 17.71 mmol) and KPi buffer (200 mM, 60 mL at pH 7.5) were mixed together. Afterwards, the pH was measured and adjusted to 7.5 with the addition of conc. HCl. At the pH 7.5, CFE of SpL was added (60 mg) and the mixture was stirred until the CFE was dissolved. The water bath was adjusted to 30 ℃ and then ethyl-2 thiophene carboxylate (0.22 g, 5.06 mmol) was added and stirred. After 1 h, the reaction was quenched with conc. HCl to reach pH 4. The aqueous phase was extracted ethyl acetate (3 x 30 mL). The combined organic phases were washed with 2 M KOH (2 x 25 mL) and with brine (1 x 40 mL). The organic phase was dried over Na_2SO_4. The Na_2SO_4 was filtered off and the organic phase evaporated under vacuum. Crude was purified via a column chromatography using the eluent mix 40% cyclohexane and 60% ethyl acetate. The organic phase was evaporated under vacuum to give 14 mg (2%) product.
^{1}H NMR (300 MHz, Chloroform-*d*) δ 7.53 (dd, *J* = 1.2, 1.2 Hz, 1H), 7.47 (dd, *J* = 1.2, 1.1 Hz, 1H), 7.07 (dd, *J* = 3.7, 3.7 Hz, 1H), 6.18 (s, 1H), 6.02 – 5.84 (m, 1H), 5.31 – 5.08 (m, 2H), 4.06 (tt, *J* = 1.5, 1.6 Hz, 2H).
^{13}C NMR (75 MHz, Chloroform-*d*) δ 161.9, 138.9, 134.2, 130, 128.2, 127.7, 116.9, 42.5.

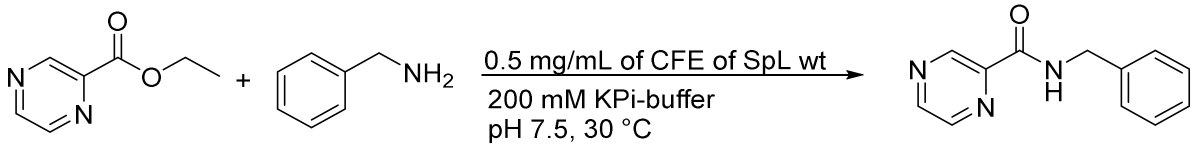

Synthesis of *N*-benzylpyrazin-2-carboxamide 3d,a:

In a 25 mL round bottomed flask, benzylamine (0.49 g, 4.55 mmol) and KPi buffer (200 mM, 20 mL at pH 7.5) were mixed together. Afterwards, the pH was measured and adjusted to 7.5 with the addition of 85% H3PO4 and 1 M NaOH. At the pH 7.5, CFE of SpL was added (10 mg) and the mixture was stirred till a clear solution was observed. The water bath was adjusted to 30 °C and then ethyl pyrazine-2-carboxylate (0.20 g, 1.3 mmol) was added and stirred. After 40 min, the reaction was quenched with conc. HCl to reach pH 4. The aqueous phase was extracted with ethyl acetate (3 x 10 mL). The combined organic phases were washed with 2 M KOH (3 x 10 mL) and with brine (2 x 10 mL). The organic phase was dried over Na_2SO_4. The Na_2SO_4 was filtered off and the organic phase was evaporated under vacuum to give 144 mg (52%) product.
^{1}H NMR (300 MHz, Chloroform-*d*) δ 9.45 (d, *J* = 1.5 Hz, 1H), 8.74 (d, *J* = 2.5 Hz, 1H), 8.50 (dd, *J* = 1.3, 1.4 Hz, 1H), 8.13 (s, 1H), 7.39 – 7.31 (m, 5H), 4.68 (d, *J* = 6.0 Hz, 2H).
^{13}C NMR (75 MHz, Chloroform-*d*) δ 163, 147.5, 144.7, 144.5, 142.7, 137.9, 128.9, 128, 127.8, 43.6.

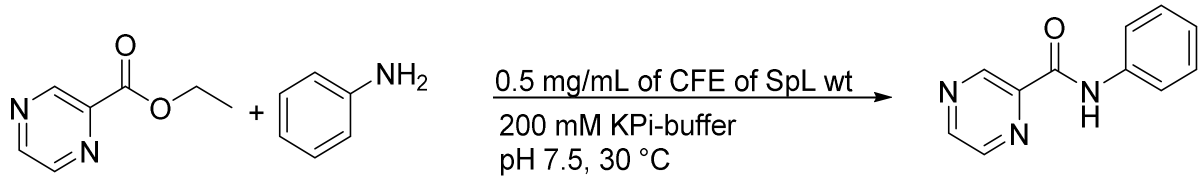

Synthesis of *N*-phenylpyrazine-2-carboxamide 3d,b:

In a 25 mL round bottomed flask, aniline (0.24 g, 2.57 mmol) and KPi buffer (200 mM, 20 mL at pH 7.5) were mixed together. Afterwards, the pH was measured and adjusted to 7.5 with the addition of 85% H3PO4. At the pH 7.5, CFE of SpL was added (10 mg) and the mixture was stirred till a clear solution was observed. The water bath was adjusted to 30 °C and then ethyl pyrazine-2-carboxylate (0.13 g, 0.85 mmol) was added and stirred. After 45 min, only a faint ester spot was observed on the TLC and the reaction was quenched with conc. HCl to reach pH 4. 5 mL brine was added to the solution. The aqueous phase was extracted ethyl acetate (3 x 5 mL). The combined organic phases were washed with 2 M KOH (3 x 10 mL) and with brine (1 x 10 mL). The organic phase was dried over Na_2SO_4. The Na_2SO_4 was filtered off and the organic phase evaporated under vacuum to give 110 mg (66%) product.
^{1}H NMR (300 MHz, Chloroform-*d*) δ 9.67 (s, 1H), 9.52 (d, *J* = 1.5 Hz, 1H), 8.81 (d, *J* = 2.5 Hz, 1H), 8.60 (m, 1H), 7.77 (d, *J* = 7.8, 2H), 7.41 (t, *J* = 8.0 Hz, 2H), 7.18 (t, *J* = 7.4 Hz, 1H).
^{13}C NMR (75 MHz, Chloroform-*d*) δ 160.77, 147.68, 144.84, 144.54, 142.49, 137.35, 129.34, 124.99, 119.94.

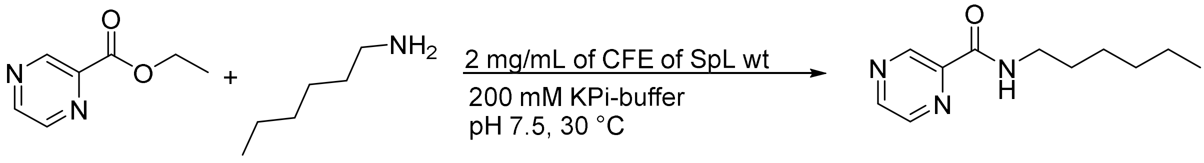

Synthesis of *N*-hexylpyrazin-2-carboxamide 3d,c:

In a 25 mL round bottomed flask, *n*-hexylamine (0.36 g, 3.6 mmol) and KPi buffer (200 mM, 14 mL at pH 7.5) were mixed together. Afterwards, the pH was measured and adjusted to 7.5 with conc. HCl. At the pH 7.5, CFE of SpL was added (14 mg) and the mixture was stirred until the CFE was dissolved. The water bath was adjusted to 30 °C and then ethyl pyrazine-2-carboxylate (0.30 g, 1.03 mmol) was added and stirred. After 47 min, no ester spot was observed on the TLC and the reaction was quenched with conc. HCl to reach pH 4. The aqueous phase was extracted ethyl acetate (3 x 10 mL). The combined organic phases were washed with 2 M KOH (1 x 5 mL). The organic phase was dried over Na_2SO_4. The Na_2SO_4 was filtered off and the organic phase was evaporated under vacuum to give 58 mg (27%) product.
^{1}H NMR (300 MHz, Chloroform-*d*) δ 9.41 (d, *J* = 1.5 Hz, 1H), 8.74 (d, *J* = 2.5 Hz, 1H), 8.51 (t, *J* = 2.0 Hz, 1H), 7.80 (s, 1H), 3.48 (q, *J* = 6.8 Hz, 2H), 1.63 (m, 2H), 1.47 – 1.23 (m, 6H), 0.87 (t, *J* = 7, 3H).
^{13}C NMR (75 MHz, Chloroform-*d*) δ 163, 147.3, 144.8, 144.6, 142.6, 39.6, 31.6, 29.7, 26.8, 22.7, 14.2.

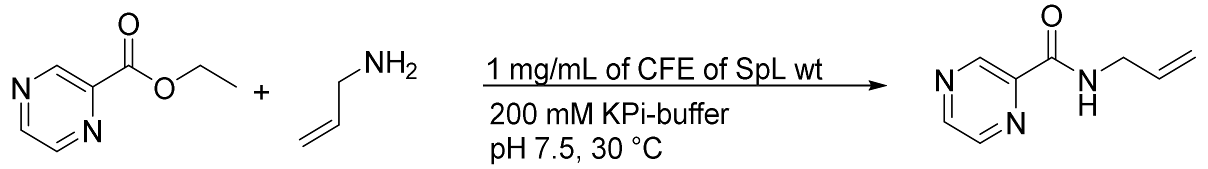

Synthesis of *N*-allylpyrazin-2-carboxamide 3d,d:

In a 25 mL round bottomed flask, benzylamine (1.2 g, 21.06 mmol) and KPi buffer (200 mM, 120 mL at pH 7.5) were mixed together. Afterwards, the pH was measured and adjusted to 7.5 with the addition of conc. HCl. At the pH 7.5, CFE of SpL was added (120 mg) and the mixture was stirred until the CFE was dissolved. The water bath was adjusted to 30 °C and then the ethyl pyrazine-2-carboxylate (0.14 g, 1.03 mmol) was added and stirred. After 20 min, the reaction was quenched with conc. HCl to reach pH 4. The aqueous phase was extracted ethyl acetate (3 x 50 mL). The combined organic phases were washed with 2 M KOH (2 x 30 mL) and with brine (1 x 30 mL). The organic phase was dried over Na_2SO_4. The Na_2SO_4 was filtered off and the organic phase evaporated under vacuum. The crude was purified via a column chromatography using first the eluent mixture 30% cyclohexane and 70% ethyl acetate and then to elute the product with ethyl acetate. The organic phase was evaporated to give 13 mg (1%) product.
^{1}H NMR (300 MHz, Chloroform-*d*) δ 9.41 (d, *J* = 1.5 Hz, 1H), 8.75 (d, *J* = 2.5 Hz, 1H), 8.52 (dd, *J* = 1.5 Hz, 1H), 7.89 (s, 1H), 5.93 (m, 1H), 5.37 – 5.14 (m, 2H), 4.12 (tt, *J* = 1.4, 1.5 Hz, 2H).
^{13}C NMR (75 MHz, Chloroform-*d*) δ 163, 147.4, 144.6, 144.6, 142.7, 133.8, 117, 41.9.

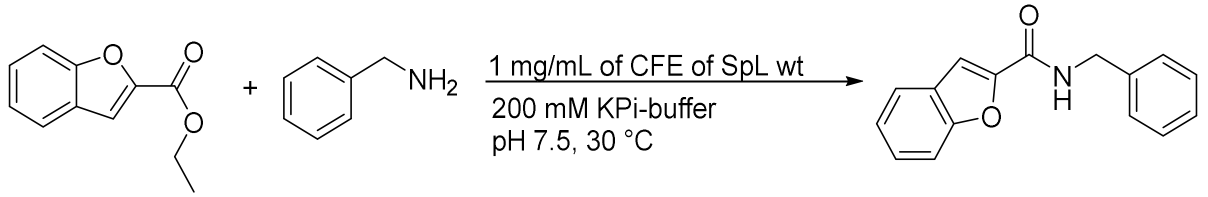

Synthesis of *N*-benzyl-1-benzofuran-2-carboxamide 3h,a:

In a 14 mL conical plastic tube, benzylamine (0.12 g, 1.10 mmol) and KPi buffer (200 mM, 5 mL at pH 7.5) were mixed together. Afterwards, the pH was measured and adjusted to 7.5 with the addition of 85% H3PO4. At the pH 7.5, CFE of SpL was added (5 mg) and the mixture was mixed until the CFE was dissolved. Then, ethyl-1-benzofuran-2-carboxylate (0.122 g, 0.64 mmol) was added and everything was incubated at 30 °C, 120 rpm. After 18 h, the reaction was quenched with conc. HCl to reach pH 4. The aqueous phase was extracted ethyl acetate (3 x 2 mL). The combined organic phases were washed with 2 M KOH (2 x 3 mL). The organic phase was dried over Na_2SO_4. The Na_2SO_4 was filtered off and the organic phase evaporated under vacuum to give 32 mg (41%) product.
^{1}H NMR (300 MHz, Chloroform-*d*) δ 7.68 (d, *J* = 7.9 Hz, 1H), 7.54 – 7.22 (m, 9H), 6.94 (s, 1H), 4.68 (d, *J* = 5.9 Hz, 2H).
^{13}C NMR (75 MHz, Chloroform-*d*) δ 158.9, 154.9, 137.9, 129, 128.2, 127.9, 127.8, 127.1, 123.9, 122.9, 111.9, 110.8, 43.6.

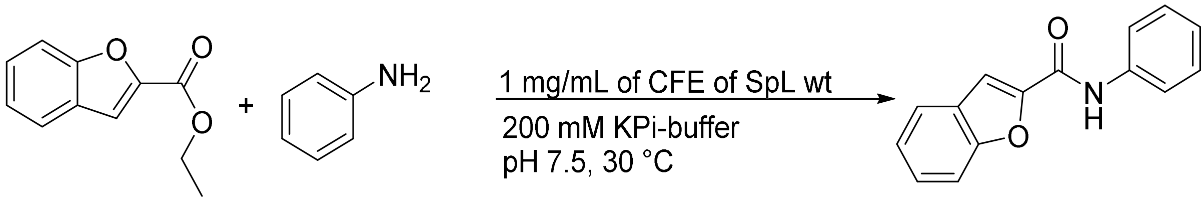

Synthesis of benzofuran-2-carboxylic acid *N*-phenylamide 3h,b:

In a 14 mL conical plastic tube, aniline (0.27 g, 2.94 mmol) and KPi buffer (200 mM, 10 mL at pH 7.5) were mixed together. Afterwards, the pH was measured and adjusted to 7.5 with the addition of 85% H3PO4. At the pH 7.5, CFE of SpL was added (10 mg) and the mixture was mixed until the CFE was dissolved. Then, ethyl-1-benzofuran-2-carboxylate (0.16 g, 0.84 mmol) was added and everything was incubated at 30 °C, 120 rpm. After 20 h, the reaction was quenched with conc. HCl to reach pH 4. The aqueous phase was extracted ethyl acetate (3 x 5 mL). The combined organic phases were washed with 2 M KOH (2 x 5 mL) and with brine (1 x 5 mL). The organic phase was dried over Na_2SO_4. The Na_2SO_4 was filtered off and the organic phase evaporated under vacuum to give 180 mg (90%) product.
^{1}H NMR (300 MHz, Chloroform-*d*) δ 8.36 (s, 1H), 7.75 – 7.69 (m, 3H), 7.61 – 7.54 (m, 2H), 7.48 – 7.31 (m, 4H), 7.18 (t, *J* = 7.4 Hz, 1H).
^{13}C NMR (75 MHz, Chloroform-*d*) δ 156.7, 154.9, 148.6, 137.4, 129.3, 127.8, 127.4, 124.9, 124.1, 123, 120.1, 112, 111.6.

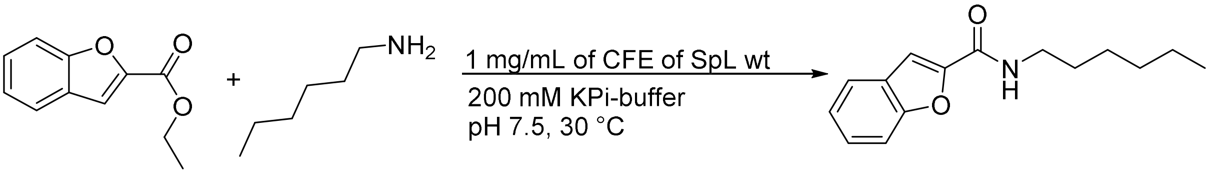

Synthesis of *N*-hexyl-1-benzofuran-2-carboxamide 3h,c:

In a 14 mL conical plastic tube, *n*-hexylamine (0.23 g, 2.23 mmol) and KPi buffer (200 mM, 10 mL at pH 7.5) were mixed together. Afterwards, the pH was measured and adjusted to 7.5 with the addition of 85% H3PO4. At the pH 7.5, CFE of SpL was added (10 mg) and the mixture was mixed until the CFE was dissolved. Then, ethyl-1-benzofuran-2-carboxylate (0.122 g, 0.64 mmol) was added and everything was incubated at 30 °C, 120 rpm. After 18 h, the reaction was quenched with conc. HCl to reach pH 4. The aqueous phase was extracted ethyl acetate (3 x 5 mL). The combined organic phases were washed with 2 M KOH (2 x 3 mL). The organic phase was dried over Na_2SO_4. The Na_2SO_4 was filtered off and the organic phase evaporated under vacuum to give 128 mg (82%) product.
^{1}H NMR (300 MHz, Chloroform-*d*) δ 7.67 (d, *J* = 7.8 Hz, 1H), 7.52 – 7.36 (m, 3H), 7.32 – 7.27 (m, 1H), 6.64 (s, 1H), 3.48 (q, *J* = 6.9 Hz, 2H), 1.72 – 1.57 (m, 2H), 1.44 – 1.24 (m, 6H), 0.89 (t, *J* = 6.7, 3H).
^{13}C NMR (75 MHz, Chloroform-*d*) δ 159, 154.8, 149.1, 127.8, 126.9, 123.8, 122.9, 111.8, 110.3, 39.5, 31.6, 29.8, 26.8, 22.7 14.2.

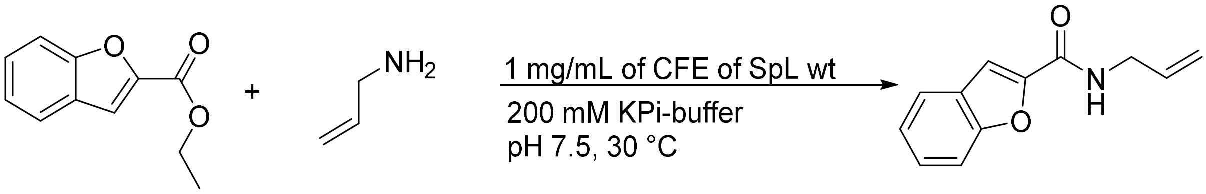

Synthesis of *N*-prop-2-enyl-1-benzofuran-2-carboxamide 3h,d:

In a 250 mL round bottomed flask, allylamine (1.75 g, 30.67 mmol) and KPi buffer (200 mM, 146 mL at pH 7.5) were mixed together. Afterwards, the pH was measured and adjusted to 7.5

with the addition of conc. HCl. At the pH 7.5, CFE of SpL was added (150 mg) and the mixture was stirred until the CFE was dissolved. The water bath was adjusted to 30 °C and then ethyl-1-benzofuran-2-carboxylate (1 g, 5.26 mmol) was added and stirred. After 4.3 h, no ester spot was observed on the TLC and the reaction was quenched with conc. HCl to reach pH 4. The aqueous phase was extracted ethyl acetate (3 x 20 mL). The combined organic phases were washed with 2 M KOH (3 x 20 mL) and with brine (1 x 30 mL). The organic phase was dried over Na_2SO_4. The Na_2SO_4 was filtered off and the organic phase evaporated under vacuum. The crude was purified by column chromatography with a biotage using the eluent mixture 60% cyclohexane and 40% ethyl acetate to remove impurities, stationary phase: silica. The product was finally eluted with ethyl acetate. The organic phase was evaporated to give 132 mg (12%) product.
^{1}H NMR (300 MHz, Chloroform-*d*) δ 7.67 (d, *J* = 7.7 Hz, 1H), 7.54 – 7.36 (m, 3H), 7.33 – 7.26 (m, 1H), 6.73 (s, 1H), 6.04 – 5.85 (m, 1H), 5.38 – 5.17 (m, 2H), 4.12 (tt, *J* = 1.5 Hz, 2H).
^{13}C NMR (75 MHz, Chloroform-*d*) δ 158.8, 154.9 148.8, 133.8, 127.8, 127, 123.9, 122.9, 117.2 111.9, 110.7, 41.8.

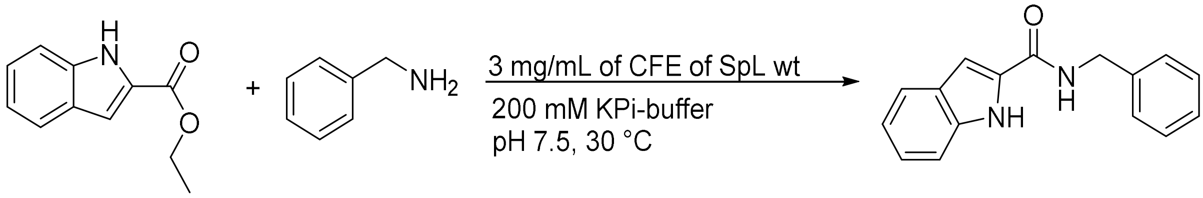

Synthesis of *N*-benzyl-1H-indole-2-carboxamide 3i,a:

In a 50 mL conical plastic tube, benzylamine (0.09 g, 1.67 mmol) and KPi buffer (200 mM, 15 mL at pH 7.5) were mixed together. Afterwards, the pH was measured and adjusted to 7.5 with the addition of conc. HCl. At the pH 7.5, 0.858 mL 17.5% triton X-100 was added followed by CFE of SpL (45 mg). Mixture was mixed, then ethyl-indole-2-carboxylate (0.09 g, 0.48 mmol) was added and the reaction vessel was incubated at 30 °C, 120 rpm. After 46 h the reaction was quenched with conc. HCl to reach pH 4. The aqueous phase was extracted ethyl acetate (3 x 6 mL). The combined organic phases were washed with 2 M KOH (2 x 6 mL). The organic phase was dried over Na_2SO_4. The Na_2SO_4 was filtered off and the organic phase evaporated under vacuum. The crude was purified by column chromatography with a biotage using a gradient with the eluents cyclohexane and ethyl acetate (stationary phase: silica, flow: 5 mL/min, 4-15% 2 CV, 15-100% 8 CV, 100% 9 CV). The organic phase was evaporated to give 61 mg (51%) product.
^{1}H NMR (300 MHz, DMSO-*d*$_6$) δ 11.61 (s, 1H), 9.04 (t, *J* = 6.1 Hz, 1H), 7.61 (d, *J* = 8.1 Hz, 1H), 7.43 (d, *J* = 7.8 Hz, 1H), 7.34 (d, *J* = 4.4 Hz, 4H), 7.30 – 7.21 (m, 1H), 7.21 – 7.14 (m, 2H), 7.07 – 7.00 (m, 1H), 4.52 (d, *J* = 6.0 Hz, 2H).
^{13}C NMR (75 MHz, DMSO-*d*$_6$) δ 161.1, 139.6, 136.5, 131.6, 128.3, 127.2, 127.1, 126.8, 123.3, 121.5, 119.7, 112.3, 102.6, 42.2.

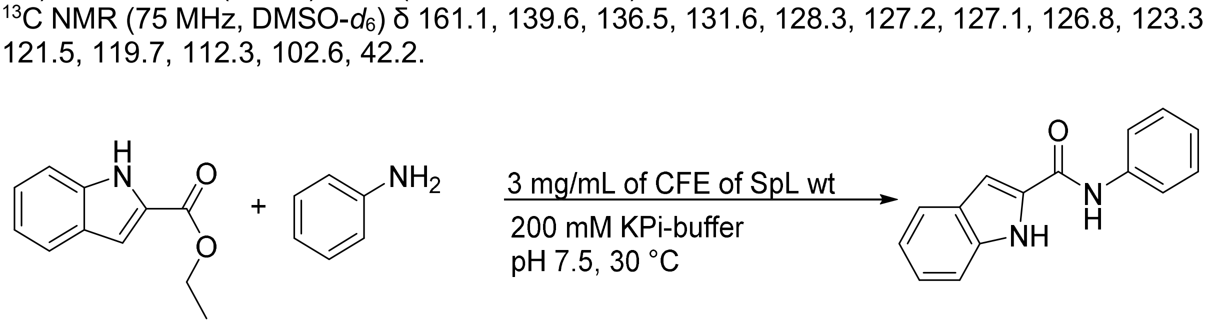

Synthesis of *N*-phenyl-1H-indole-2-carboxamide 3i,b:

In a 50 mL conical plastic tube, aniline (0.16 g, 1.76 mmol) and KPi buffer (200 mM, 15 mL at pH 7.5) were mixed together. Afterwards, the pH was measured and adjusted to 7.5 with the addition of conc. HCl. At the pH 7.5, 0.858 mL 17.5% triton X-100 was added followed by CFE of SpL (45 mg). Mixture was mixed until the CFE was dissolved, then ethylindole-2-carboxylate

(0.10 g, 0.51 mmol) was added and the reaction vessel was incubated at 30 °C, 120 rpm. After 46 h the reaction was quenched with conc. HCl to reach pH 4. The aqueous phase was extracted ethyl acetate (3 x 5 mL). The combined organic phases were washed with 2 M KOH (2 x 5 mL). The organic phase was dried over Na_2SO_4. The Na_2SO_4 was filtered off and the organic phase evaporated under vacuum. The crude was purified by column chromatography with a biotage using a gradient with the eluents cyclohexane and ethyl acetate (stationary phase: silica, flow: 5 mL/min, 4-15% 2 CV, 15-100% 8 CV, 100% 9 CV). The organic phase was evaporated to give 43 mg (36%) product.
^{1}H NMR (300 MHz, DMSO-d_6) δ 11.76 (s, 1H), 10.21 (s, 1H), 7.82 (d, J = 7.3 Hz, 2H), 7.68 (d, J = 7.9 Hz, 1H), 7.50 – 7.33 (m, 4H), 7.27 – 7.18 (m, 1H), 7.14 – 7.04 (m, 2H).
^{13}C NMR (75 MHz, DMSO-d_6) δ 159.7, 139, 136.8, 131.5, 128.7, 127, 123.8, 123.5, 121.7, 120.1, 119.9, 112.4, 103.9.

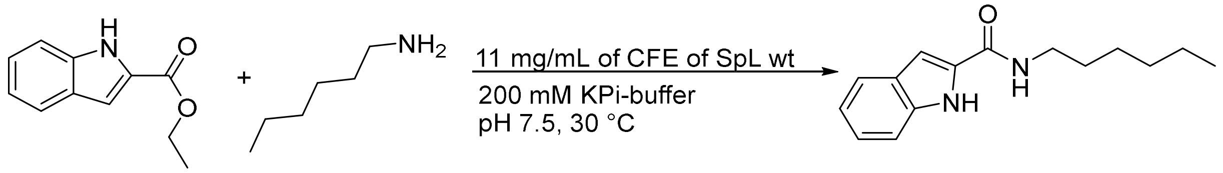

Synthesis of *N*-hexyl-indole-2-carboxamide 3i,c:

In a 50 mL conical plastic tube, *n*-hexylamine (0.44 g, 4.32 mmol) and KPi buffer (200 mM, 15 mL at pH 7.5) were mixed together. Afterwards, the pH was measured and adjusted to 7.5 with the addition of conc. HCl. At the pH 7.5, 0.858 mL 17.5% triton X-100 was added followed by CFE of SpL (165 mg). Mixture was mixed, then ethyl-indole-2-carboxylate (0.23 g, 1.24 mmol) was added and the reaction vessel was incubated at 30 ℃, 120 rpm. After 311 h (13 d), the reaction was quenched with conc. HCl to reach pH 4. The aqueous phase was extracted ethyl acetate (3 x 5 mL). The combined organic phases were washed with 2 M KOH (2 x 5 mL) and with brine (1x10 mL). The organic phase was dried over Na_2SO_4. The Na_2SO_4 was filtered off and the organic phase evaporated under vacuum. The crude was purified by column chromatography with a biotage using a gradient with the eluents cyclohexane and ethyl acetate (stationary phase: silica, flow: 5 mL/min, 4-15% 2 CV, 15-100% 8 CV, 100% 9 CV). The organic phase was evaporated to give 27 mg (9%) product.
^{1}H NMR (300 MHz, Chloroform-d) δ 9.74 (s, 1H), 7.65 (d, J = 8.0 Hz, 1H), 7.46 (dd, J = 0.7, 0.8 Hz, 1H), 7.32 – 7.24 (m, 1H), 7.17 – 7.09 (m, 1H), 6.83 (d, J = 1.3 Hz, 1H), 6.23 (t, J = 6.0 Hz, 1H), 3.51 (q, J = 7.2 , 2H), 1.73 – 1.59 (m, 2H), 1.47 – 1.28 (m, 6H), 0.89 (t, J = 7, 3H).
^{13}C NMR (75 MHz, Chloroform-d) δ 161.8, 136.5, 131.1, 127.8, 124.5, 121.95, 120.7, 112.2, 101.7, 39.9, 31.7, 29.9, 26.8, 22.7, 14.2.

NMR of products

NMR of all substances were determined with a 300 MHz Bruker Avance II system.

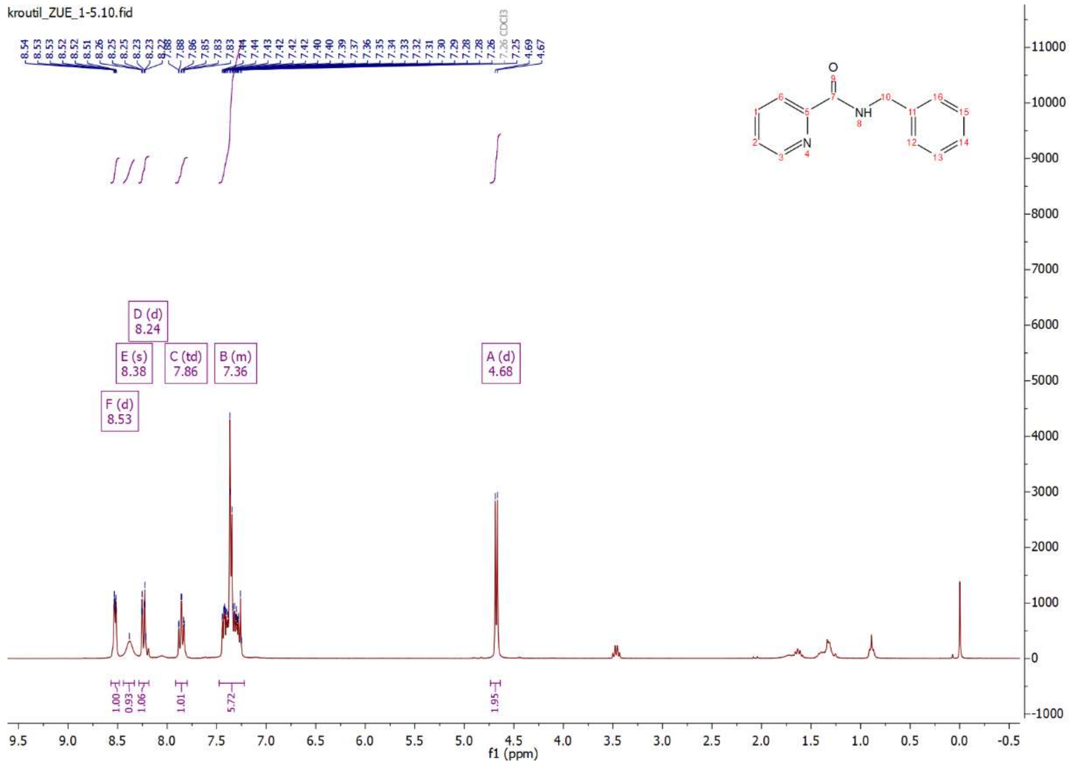

*Figure S18: ^{1}H-NMR of **3a,a** in $CDCl_3$.*

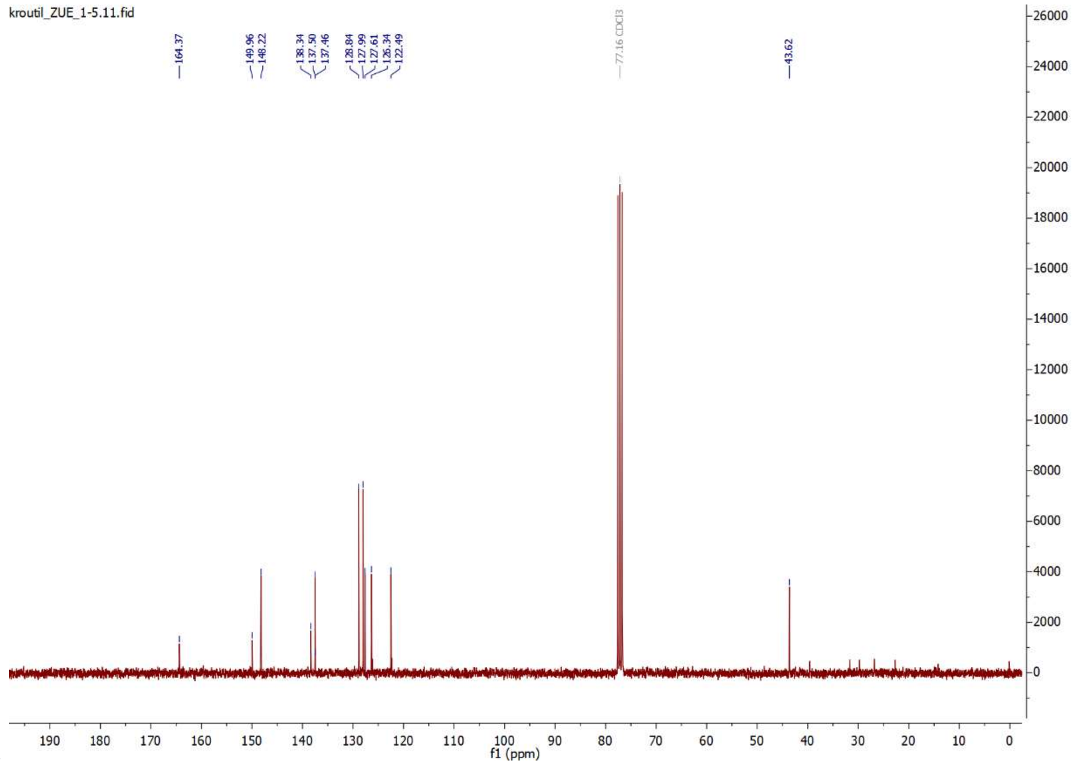

*Figure S19: ^{13}C-NMR of **3a,a** in $CDCl_3$.*

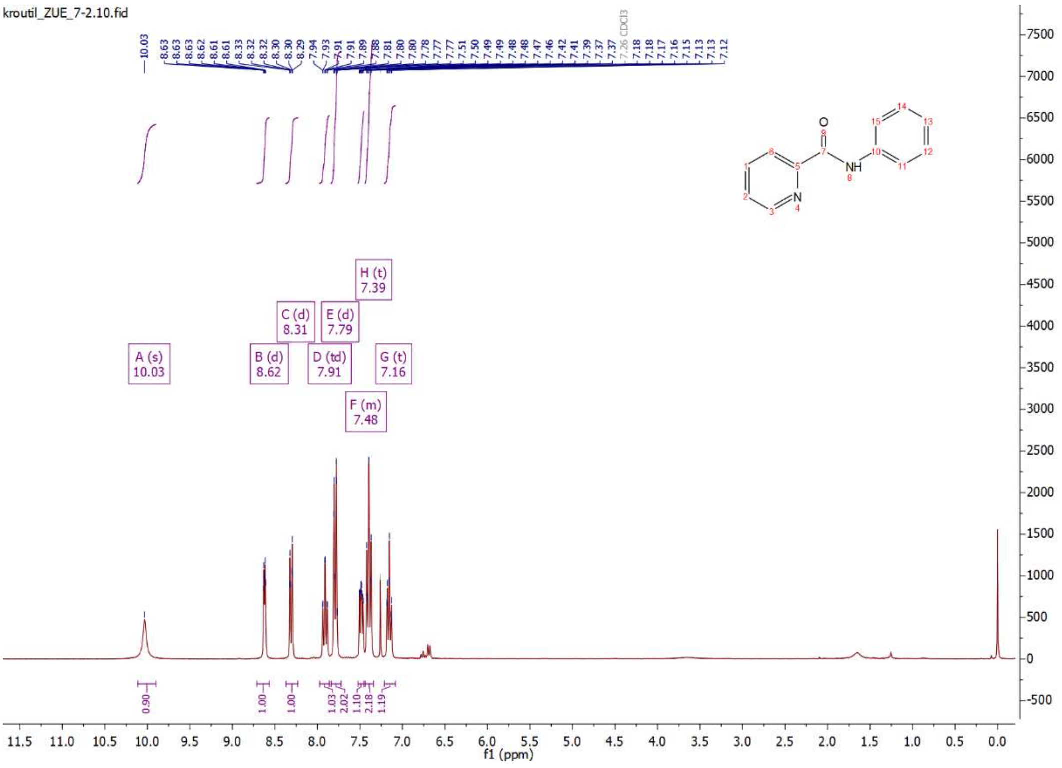

*Figure S20: ^{1}H-NMR of **3a,b** in $CDCl_3$.*

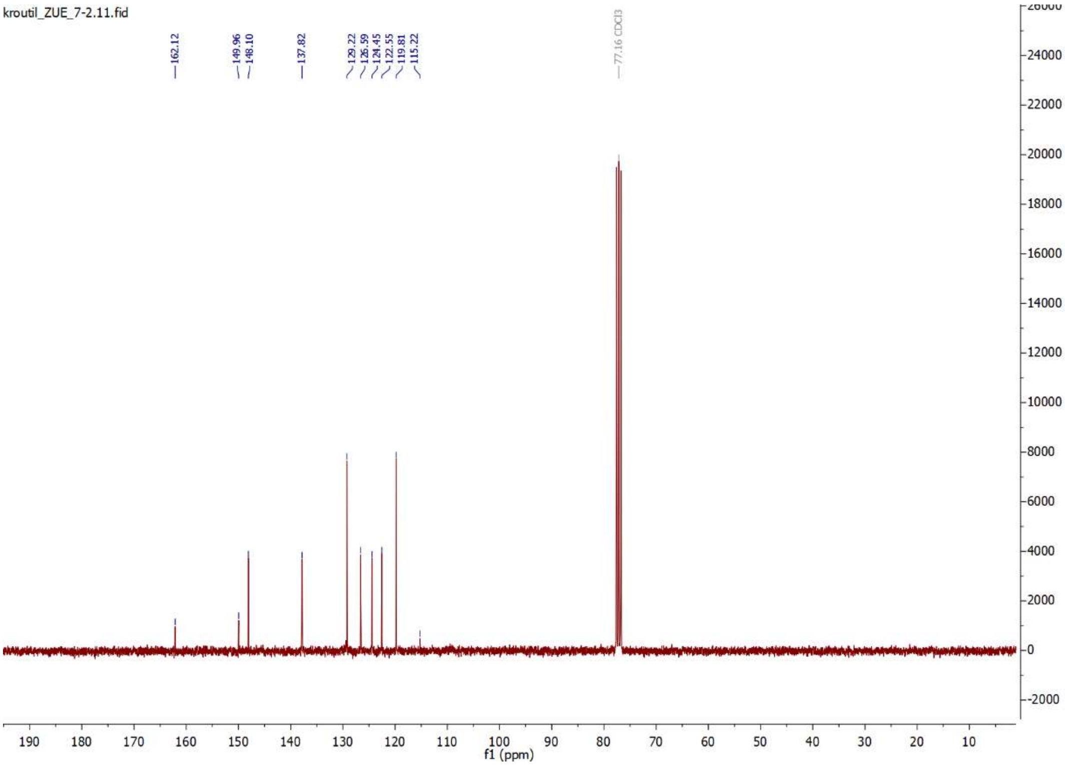

*Figure S21: ^{13}C-NMR of **3a,b** in $CDCl_3$.*

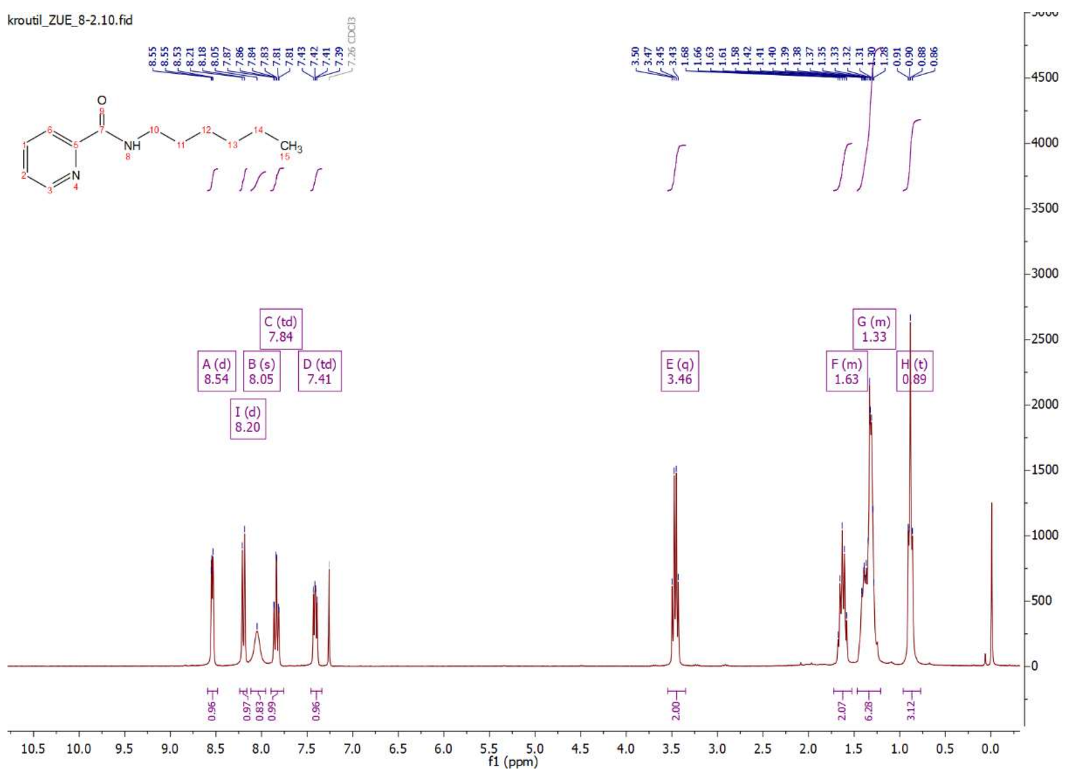

*Figure S22: ^{1}H-NMR of **3a,c** in $CDCl_3$.*

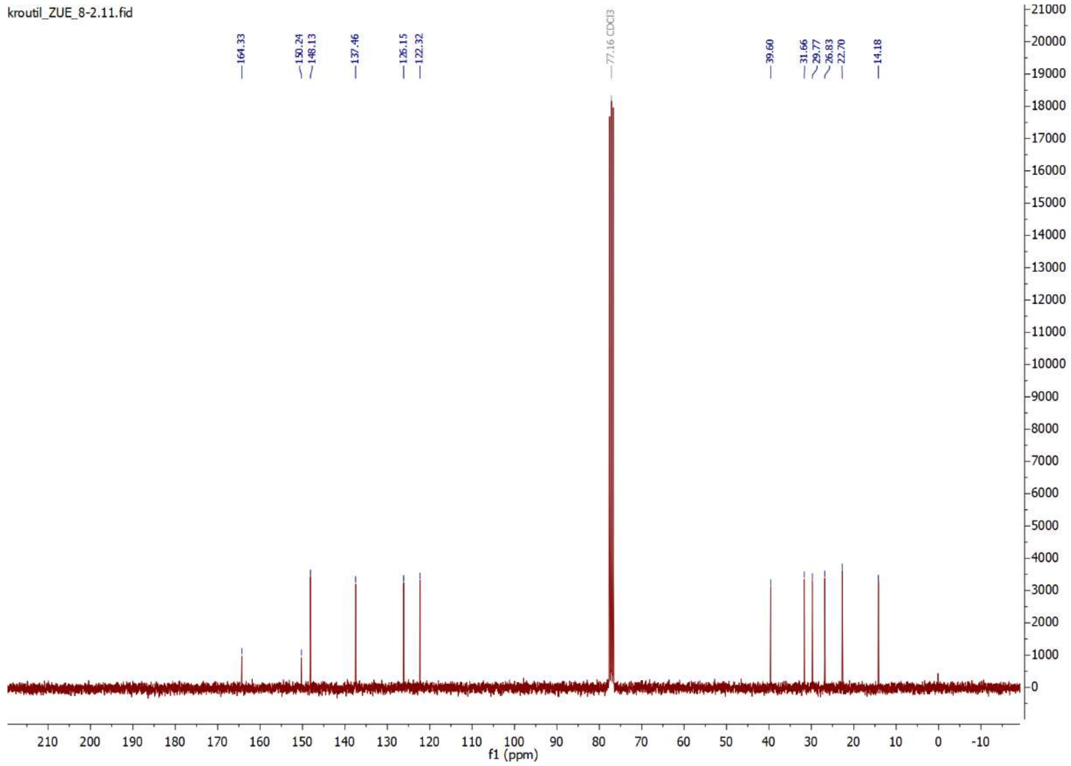

*Figure S23: ^{13}C-NMR of **3a,c** in $CDCl_3$.*

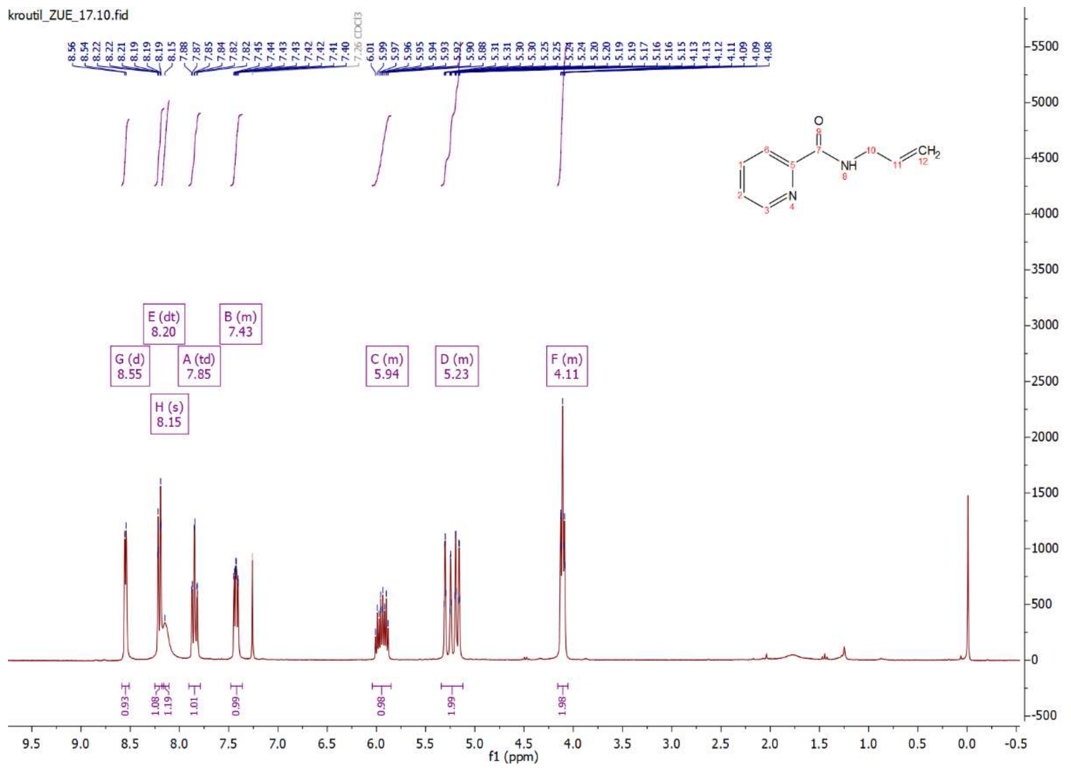

*Figure S24: ^{1}H-NMR of **3a,d** in CDCl$_3$.*

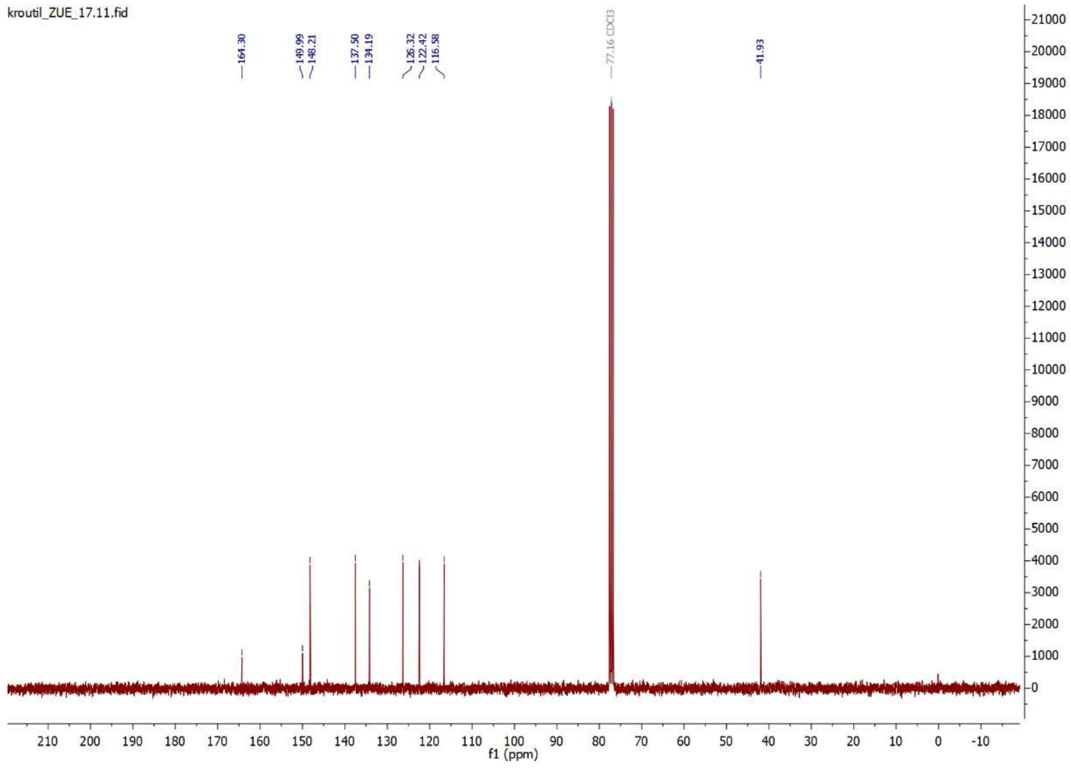

*Figure S25: ^{13}C-NMR of **3a,d** in CDCl$_3$.*

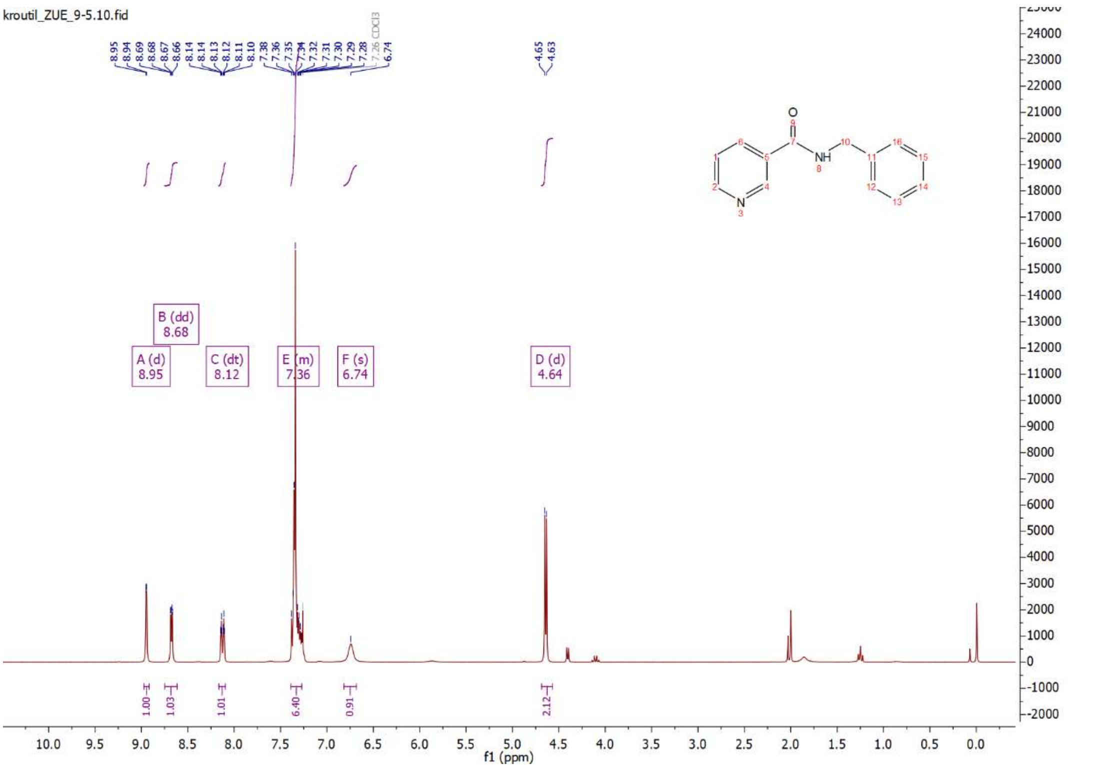

Figure S26: ^{1}H-NMR of **3b,a** in $CDCl_3$.

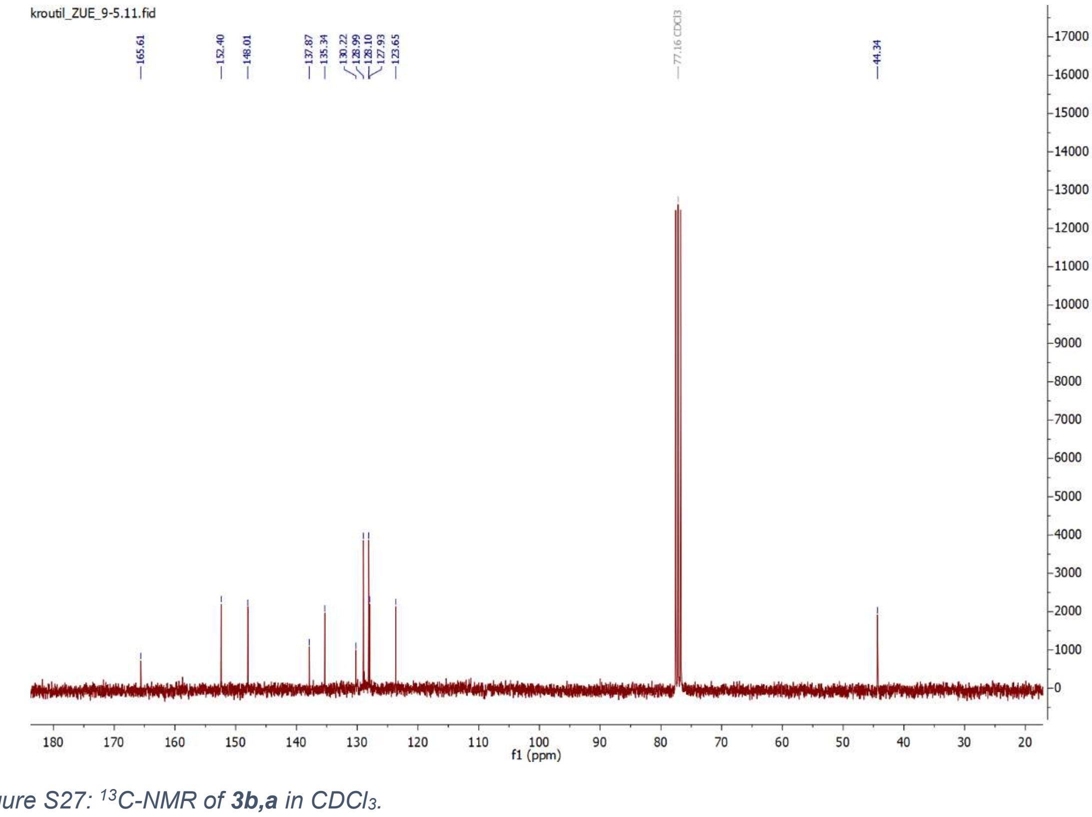

Figure S27: ^{13}C-NMR of **3b,a** in $CDCl_3$.

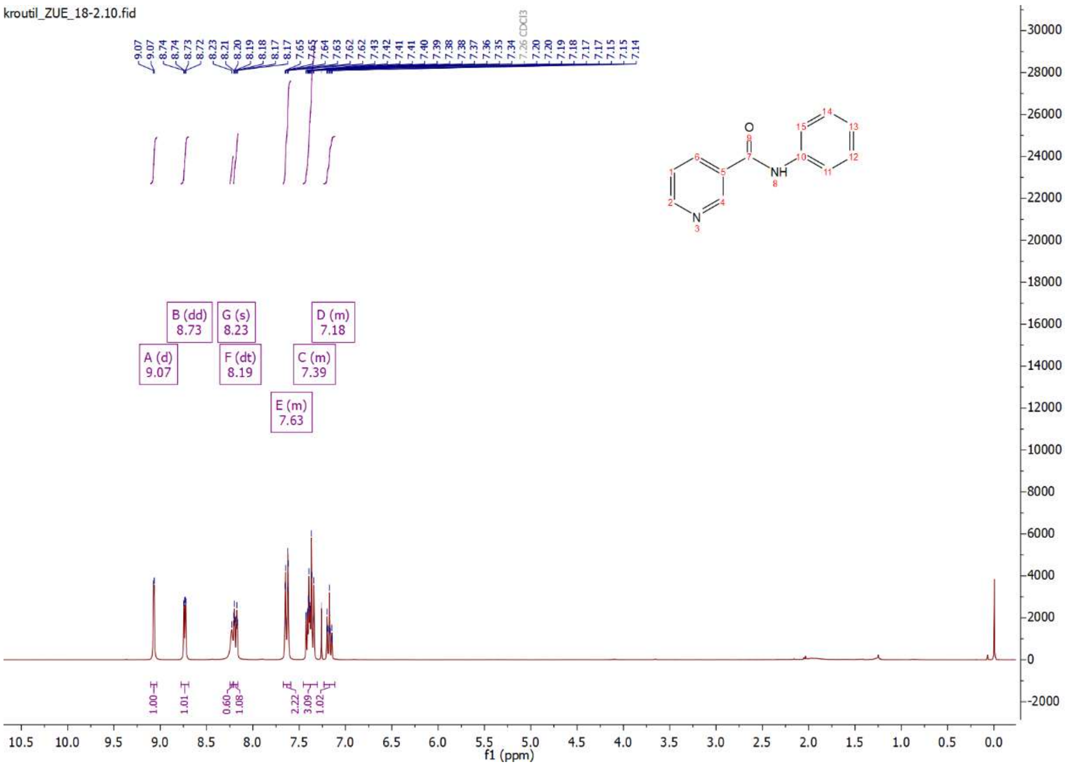

*Figure S28: ^{1}H-NMR of **3b,b** in $CDCl_3$.*

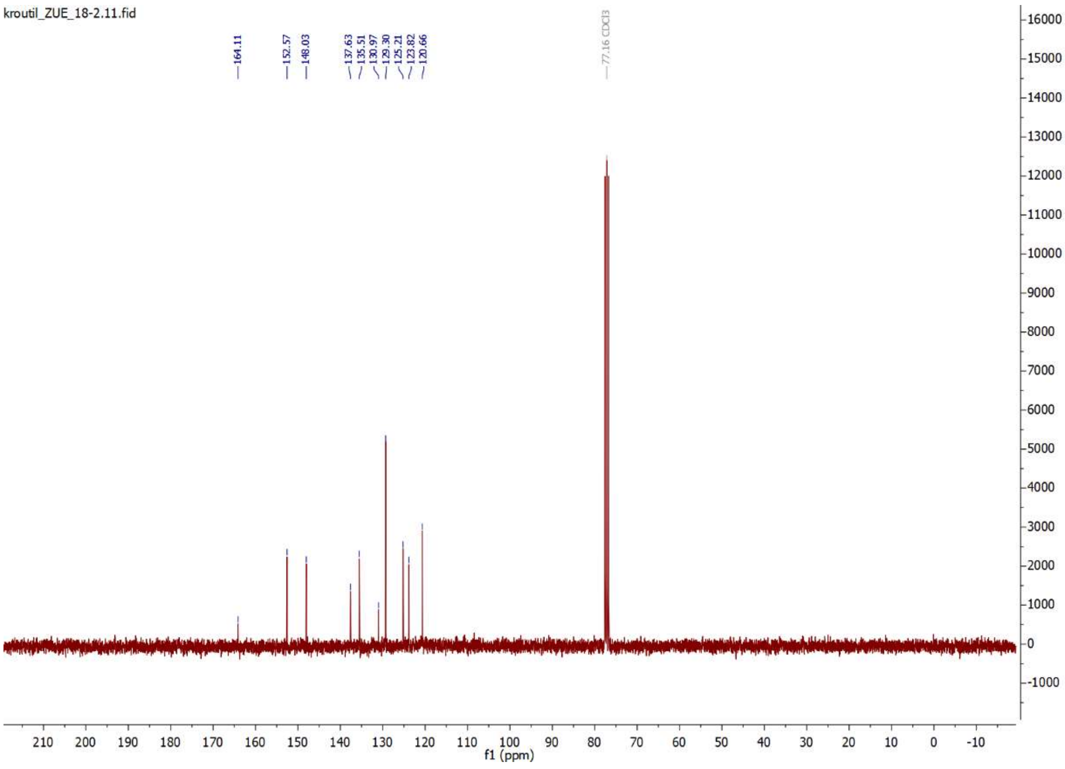

*Figure S29: ^{13}C-NMR of **3b,b** in $CDCl_3$.*

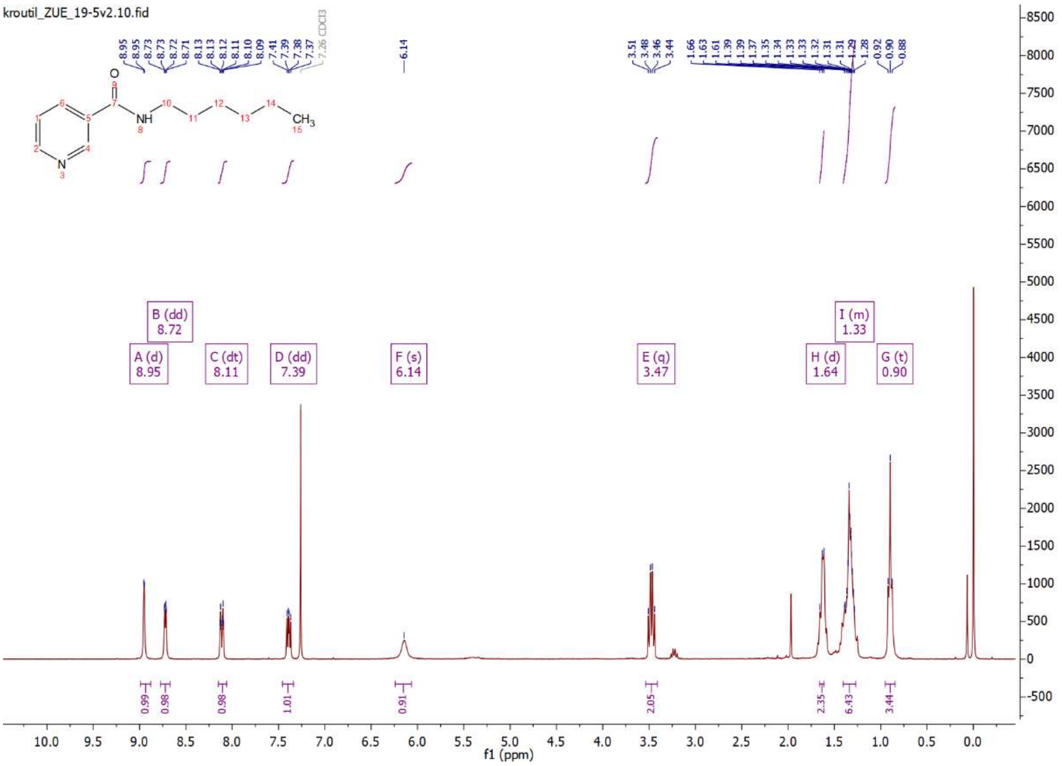

*Figure S30: ^{1}H-NMR of **3b,c** in $CDCl_3$.*

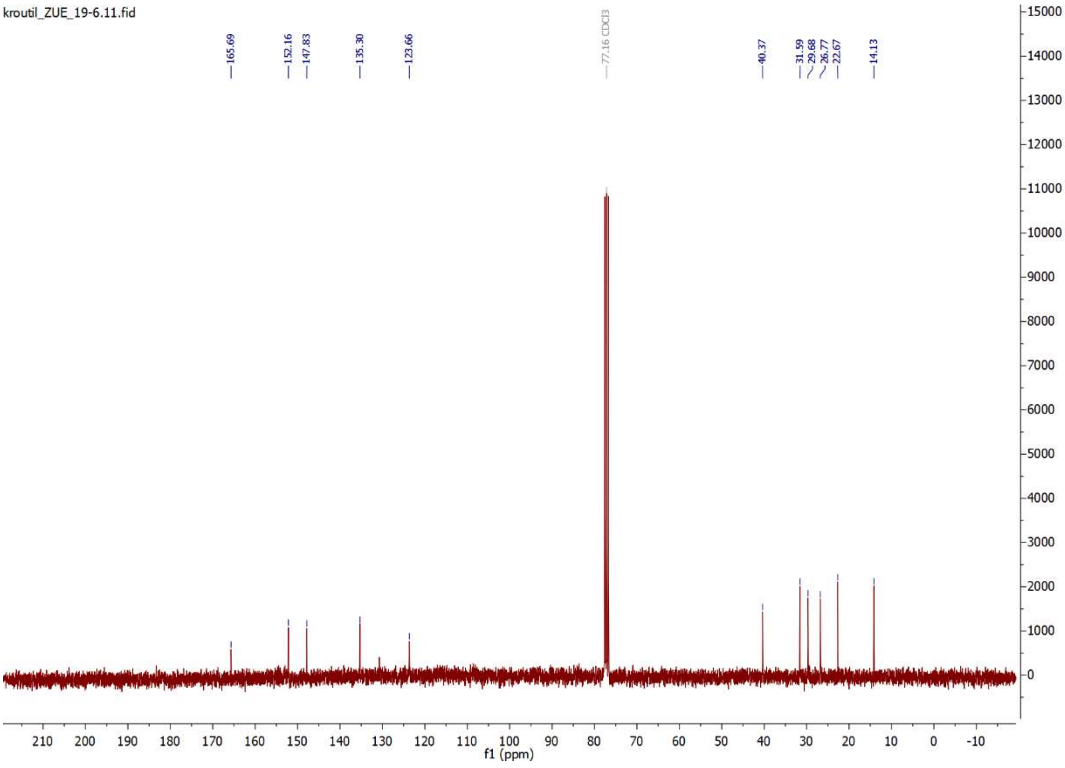

*Figure S31: ^{13}C-NMR of **3b,c** in $CDCl_3$.*

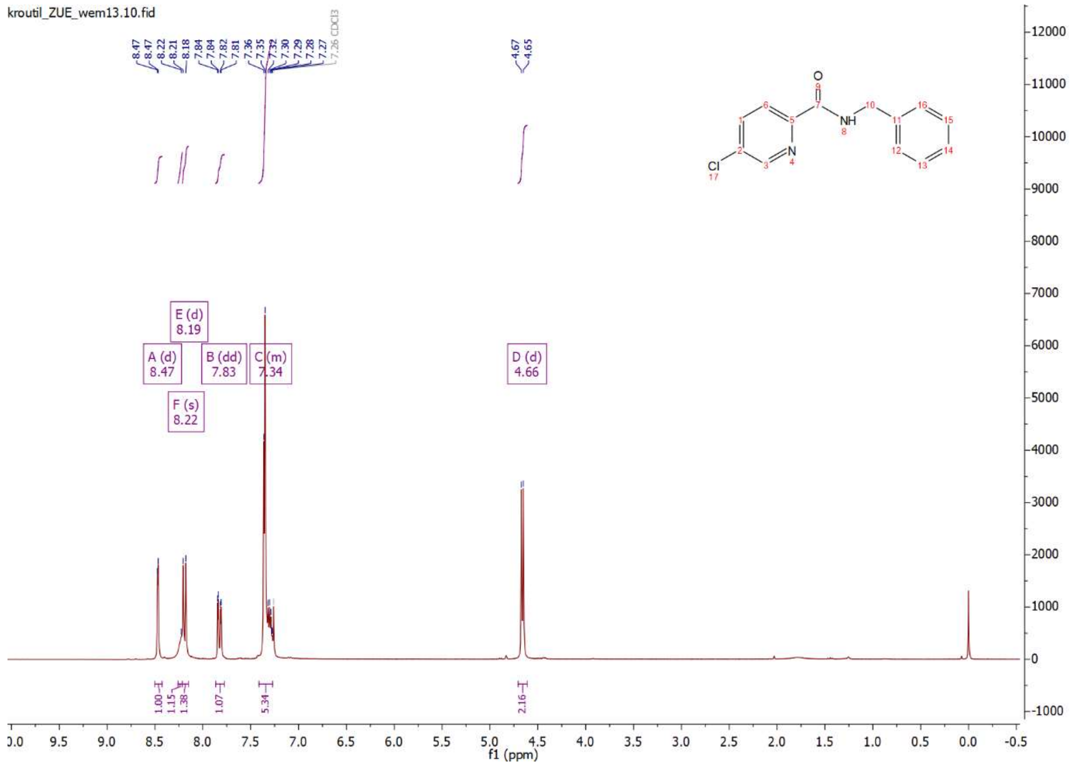

*Figure S32: ^{1}H-NMR of **3c,a** in $CDCl_3$.*

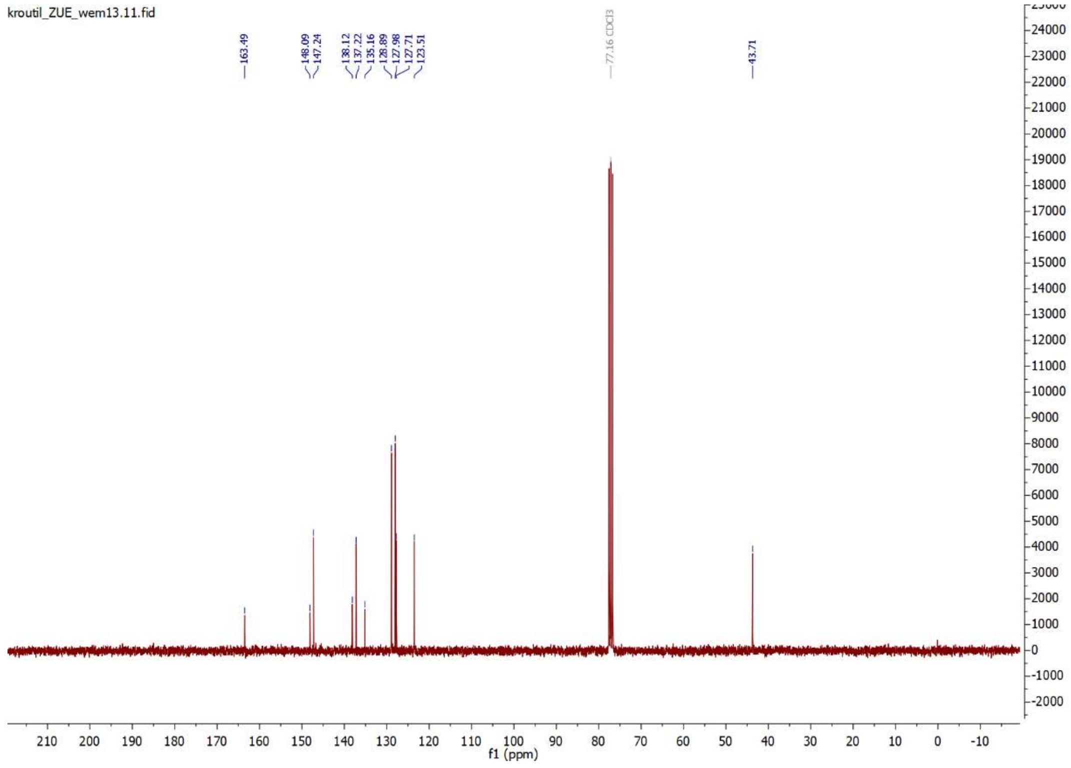

*Figure S33: ^{13}C-NMR of **3c,a** in $CDCl_3$.*

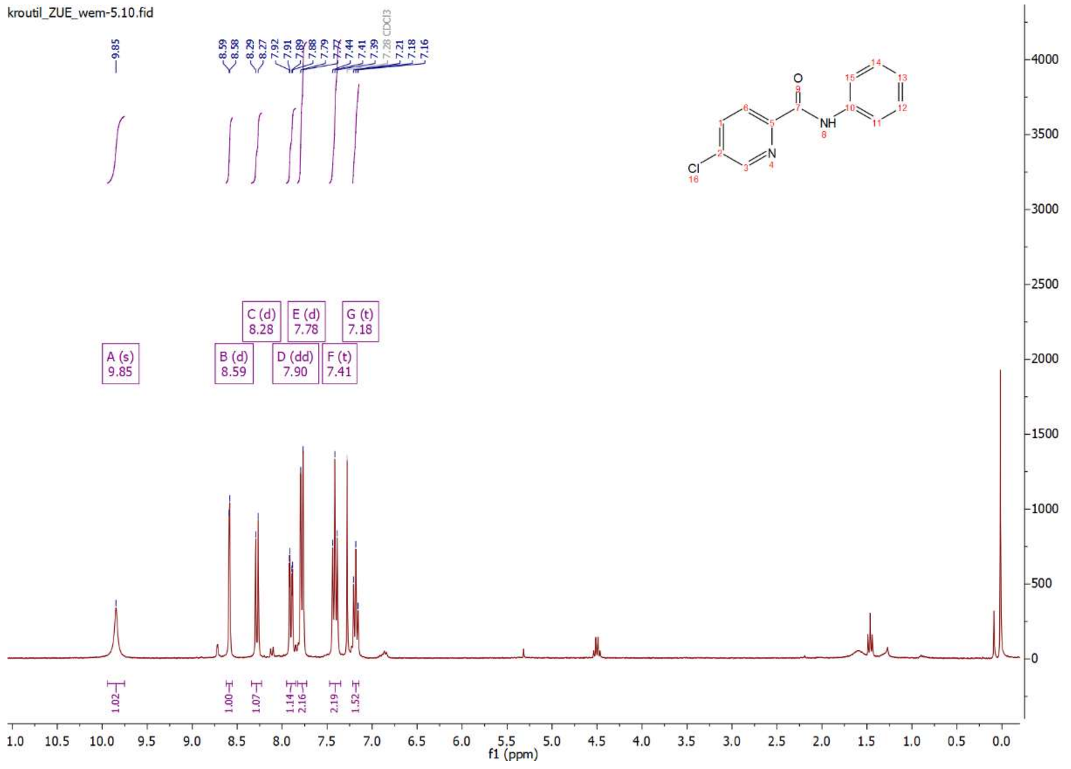

*Figure S34: ^{1}H-NMR of **3c,b** in $CDCl_3$.*

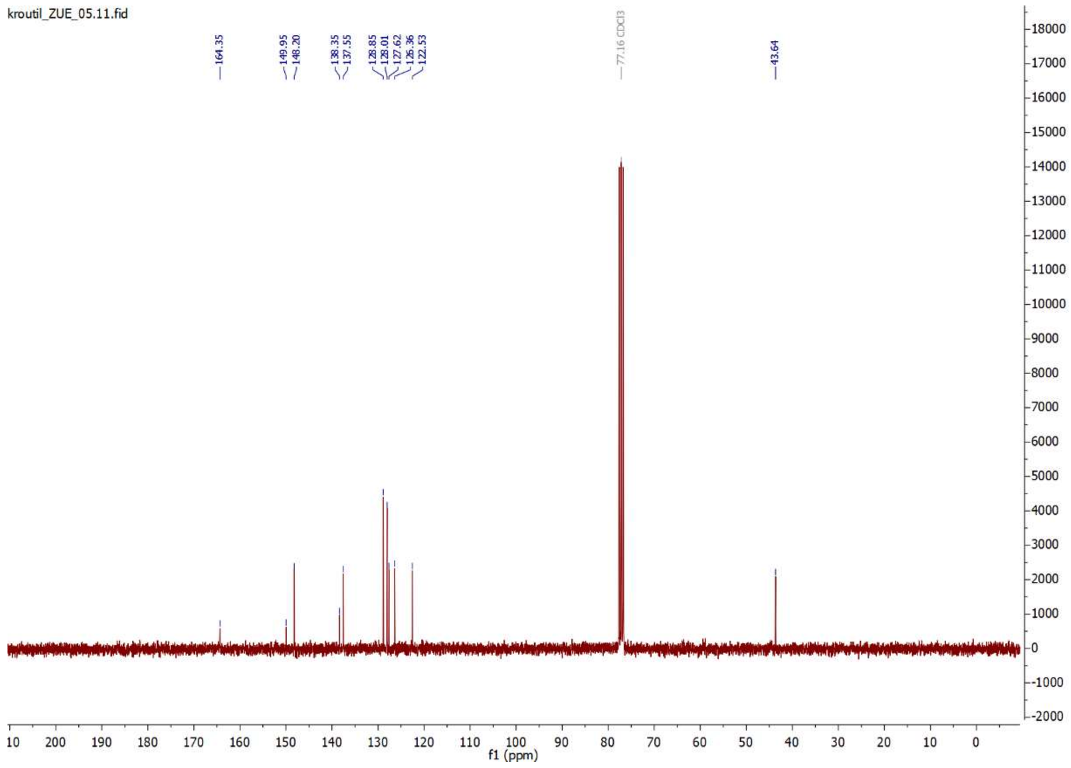

*Figure S35: ^{13}C-NMR of **3c,b** in $CDCl_3$.*

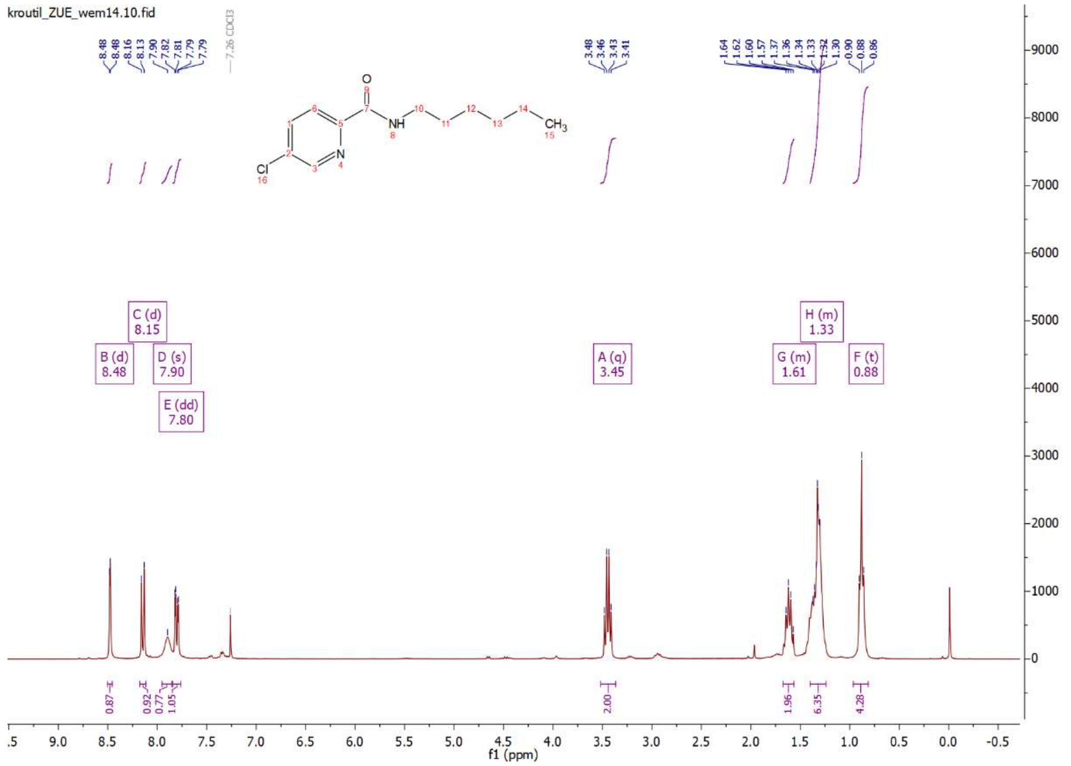

*Figure S36: ^{1}H-NMR of **3c,c** in $CDCl_3$.*

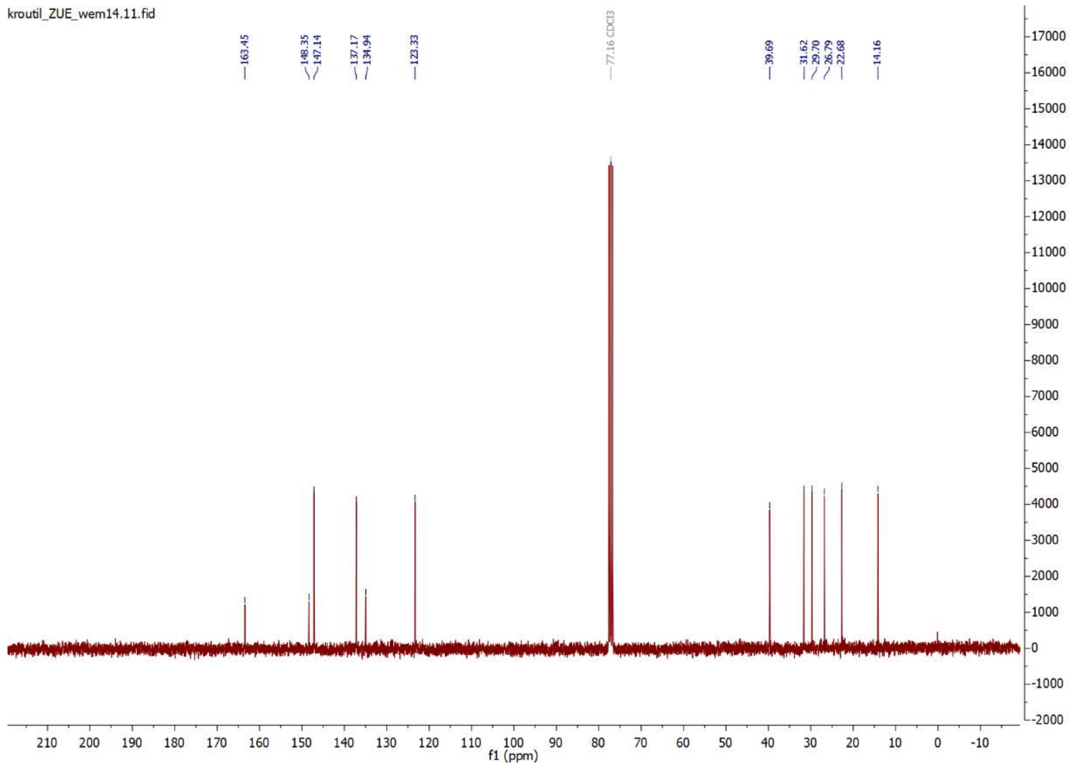

*Figure S37: ^{13}C-NMR of **3c,c** in $CDCl_3$.*

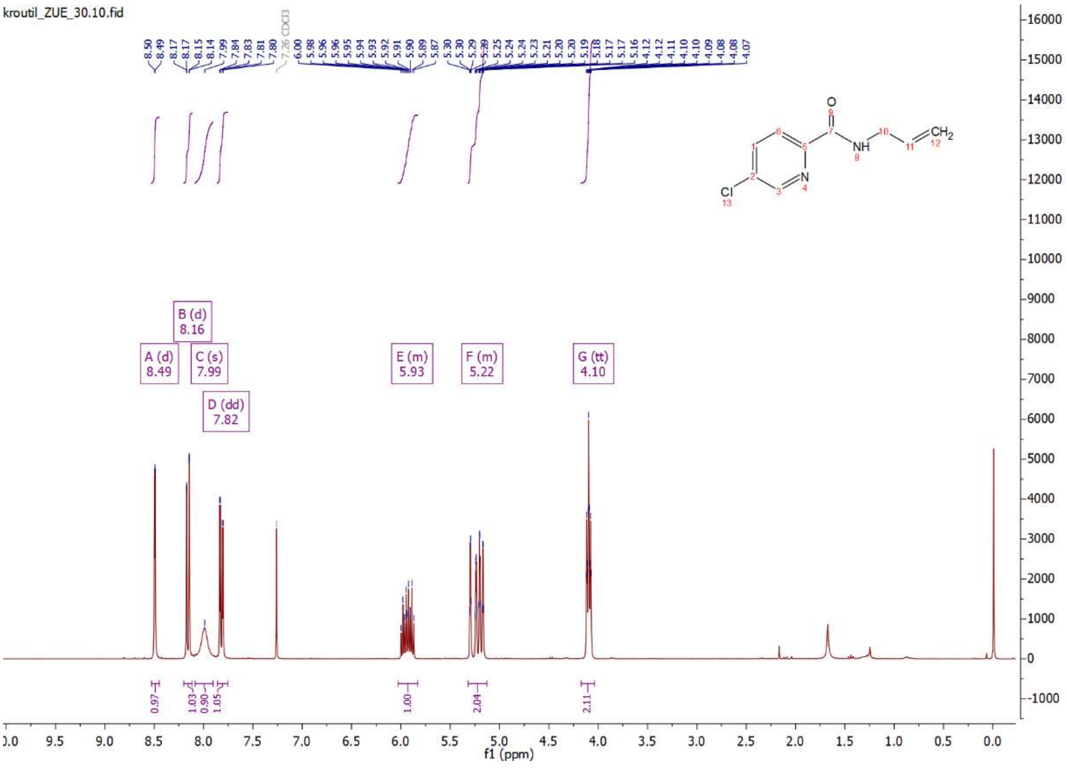

*Figure S38: ^{1}H-NMR of **3c,d** in $CDCl_3$.*

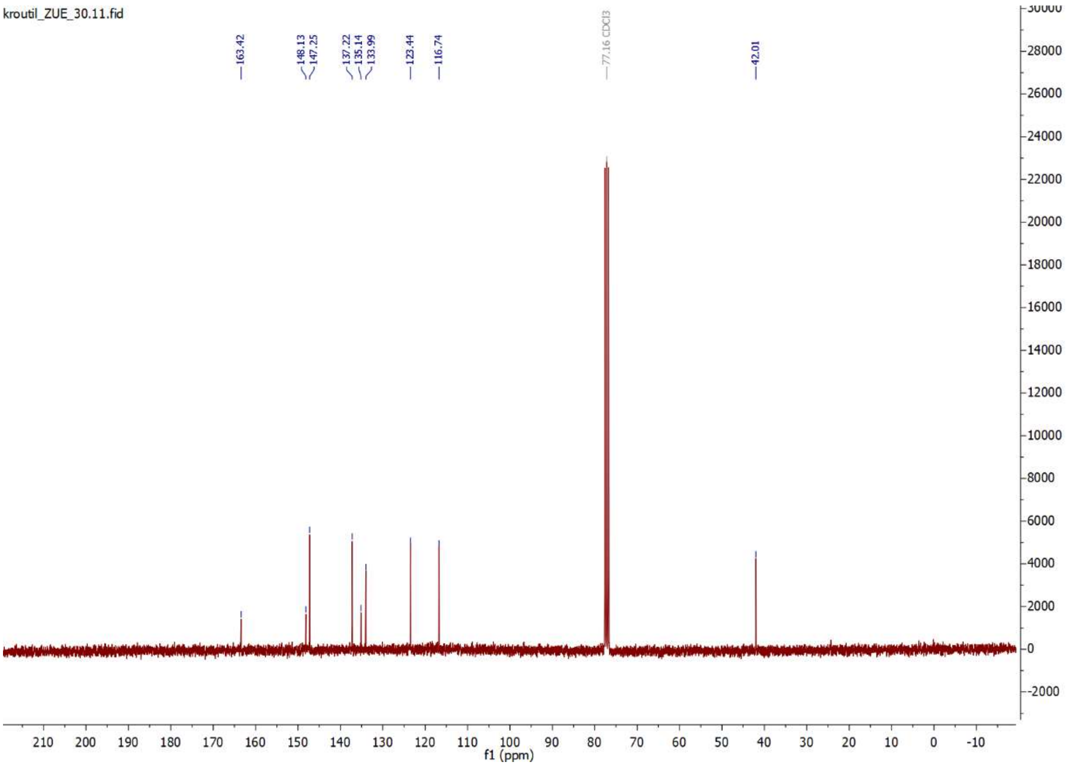

*Figure S39: ^{13}C-NMR of **3c,d** in $CDCl_3$.*

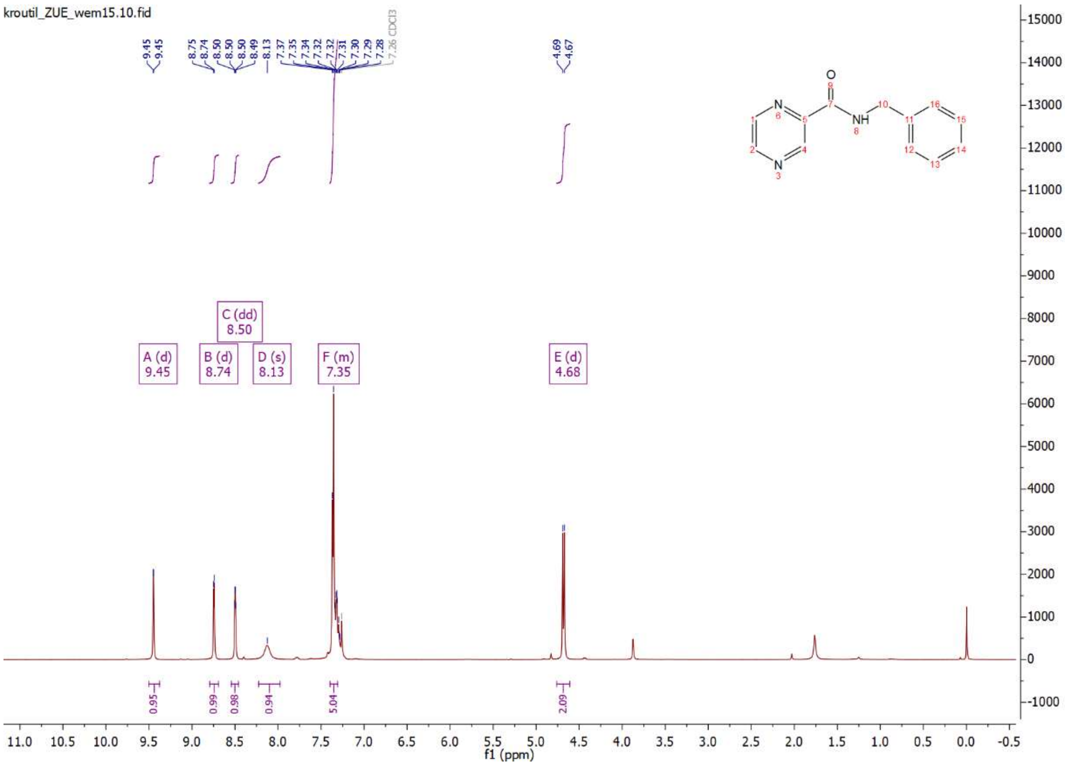

*Figure S40: ^{1}H-NMR of **3d,a** in $CDCl_3$.*

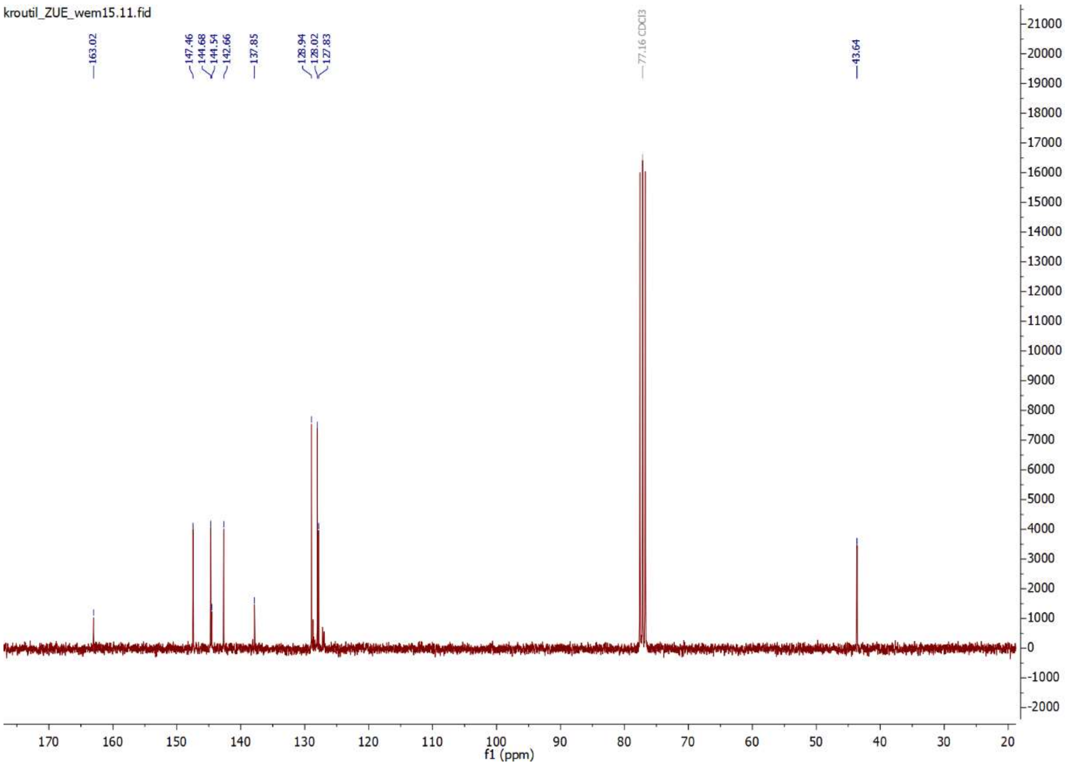

*Figure S41: ^{13}C-NMR of **3d,a** in $CDCl_3$.*

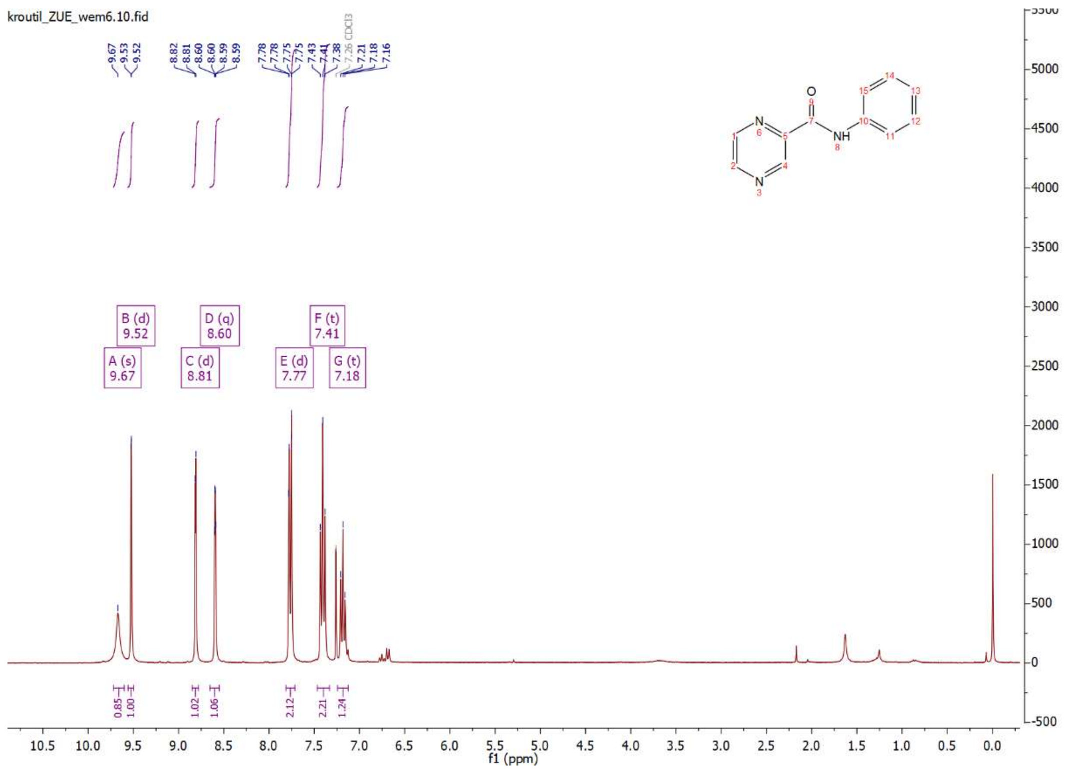

*Figure S42: ^{1}H-NMR of **3d,b** in $CDCl_3$.*

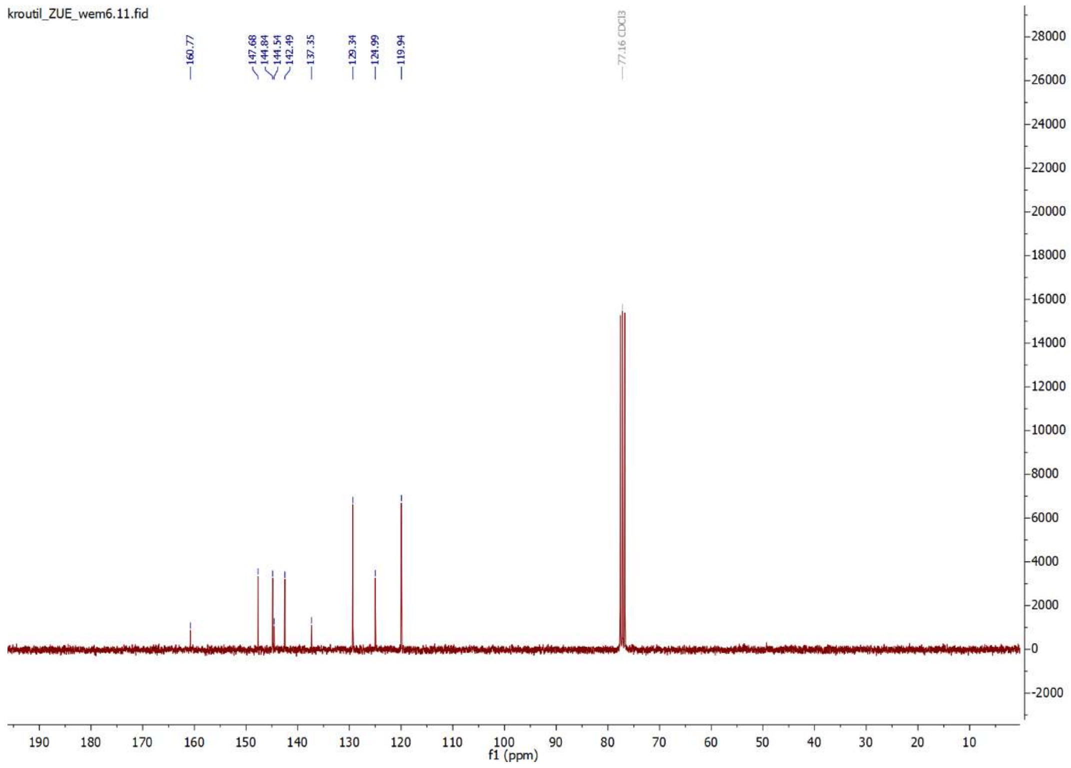

*Figure S43: ^{13}C-NMR of **3d,b** in $CDCl_3$.*

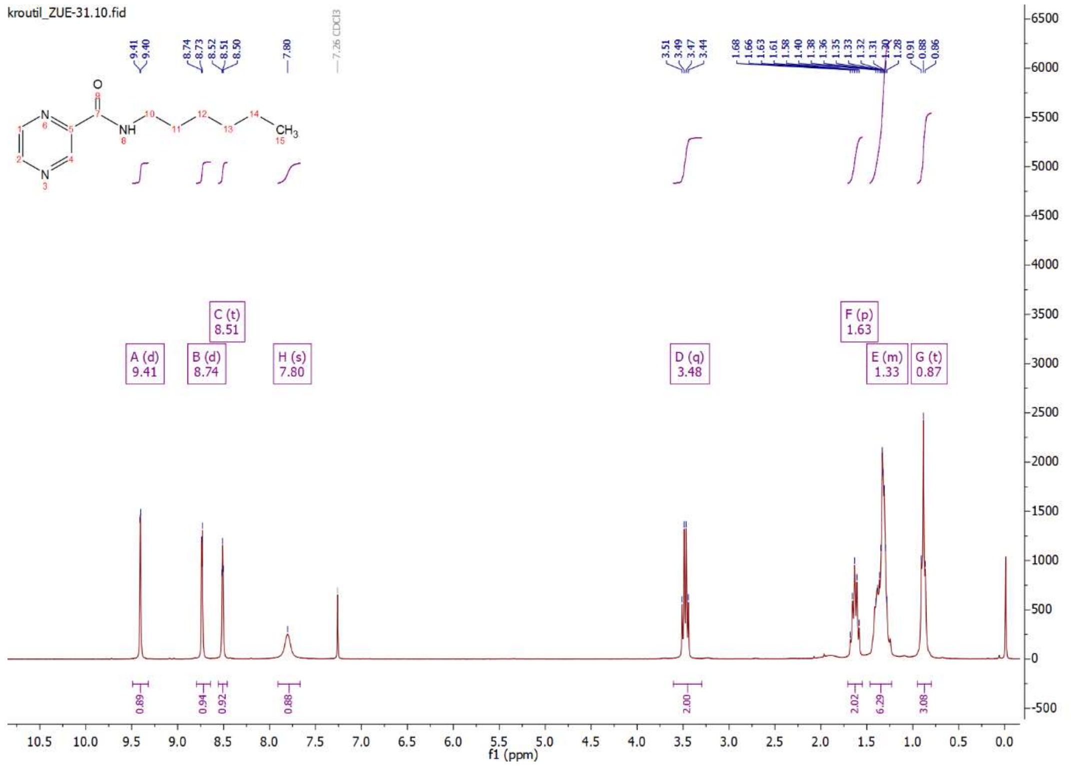

*Figure S44: ^{1}H-NMR of **3d,c** in $CDCl_3$.*

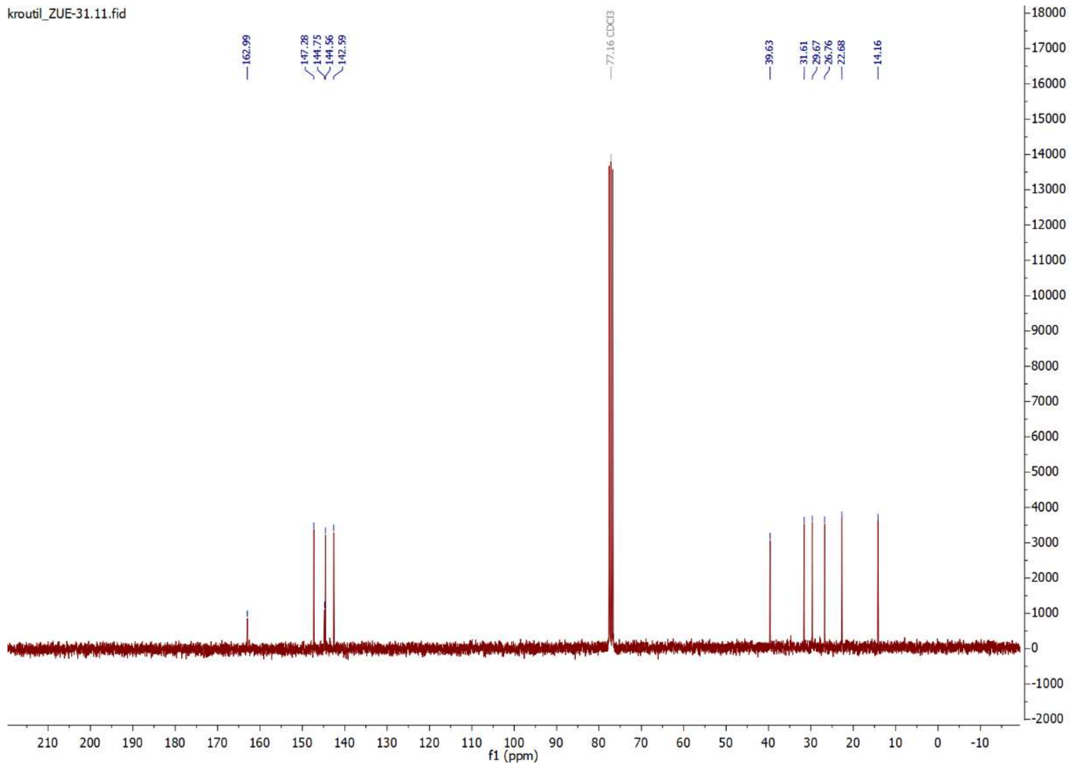

*Figure S45: ^{13}C-NMR of **3d,c** in $CDCl_3$.*

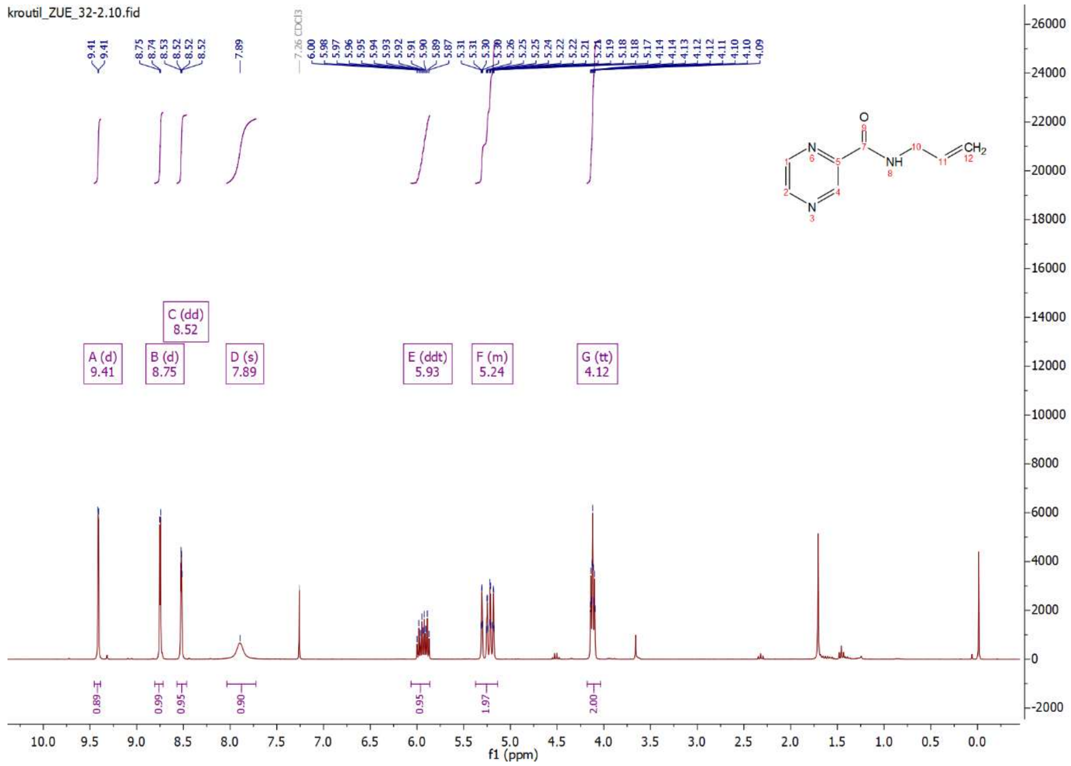

*Figure S46: ^{1}H-NMR of **3d,d** in CDCl₃.*

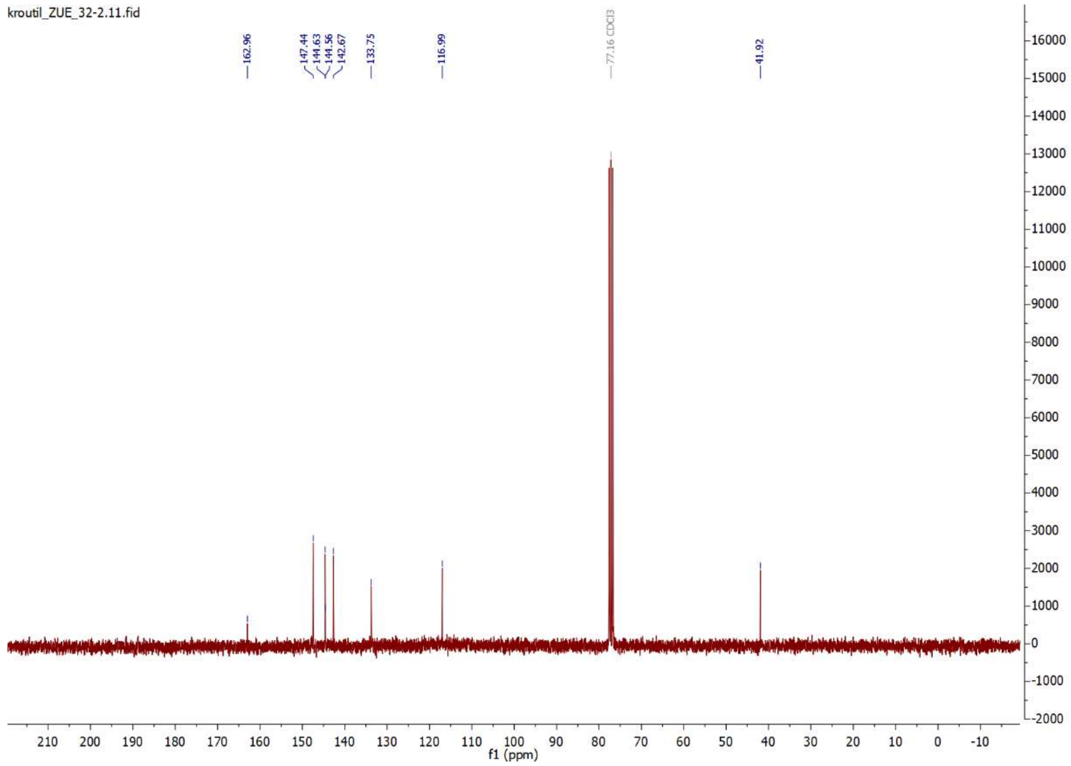

*Figure S47: ^{13}C-NMR of **3d,d** in CDCl₃.*

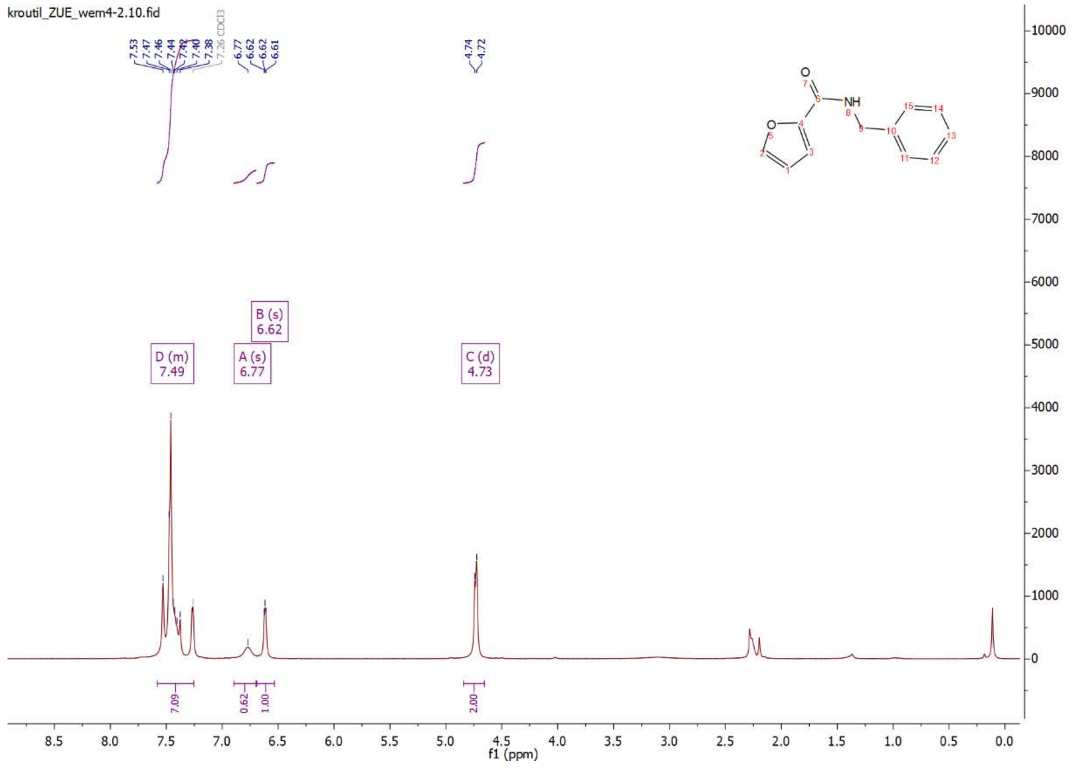

*Figure S48: ^{1}H-NMR of **3e,a** in $CDCl_3$.*

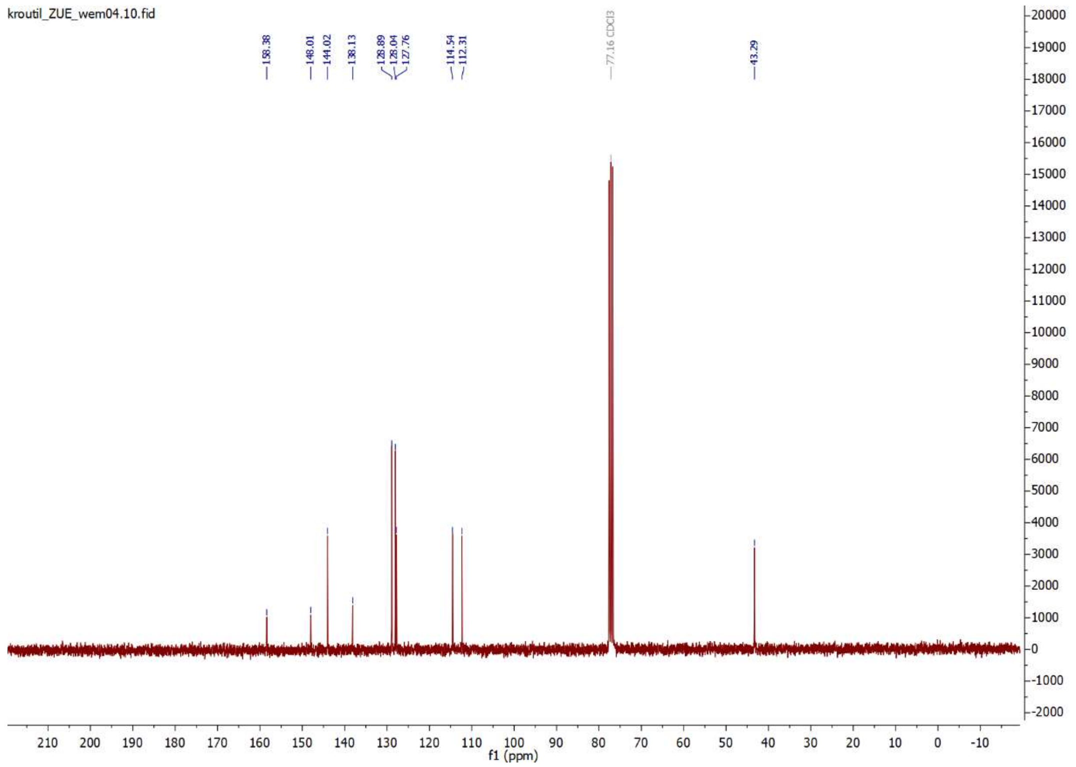

*Figure S49: ^{13}C-NMR of **3e,a** in $CDCl_3$.*

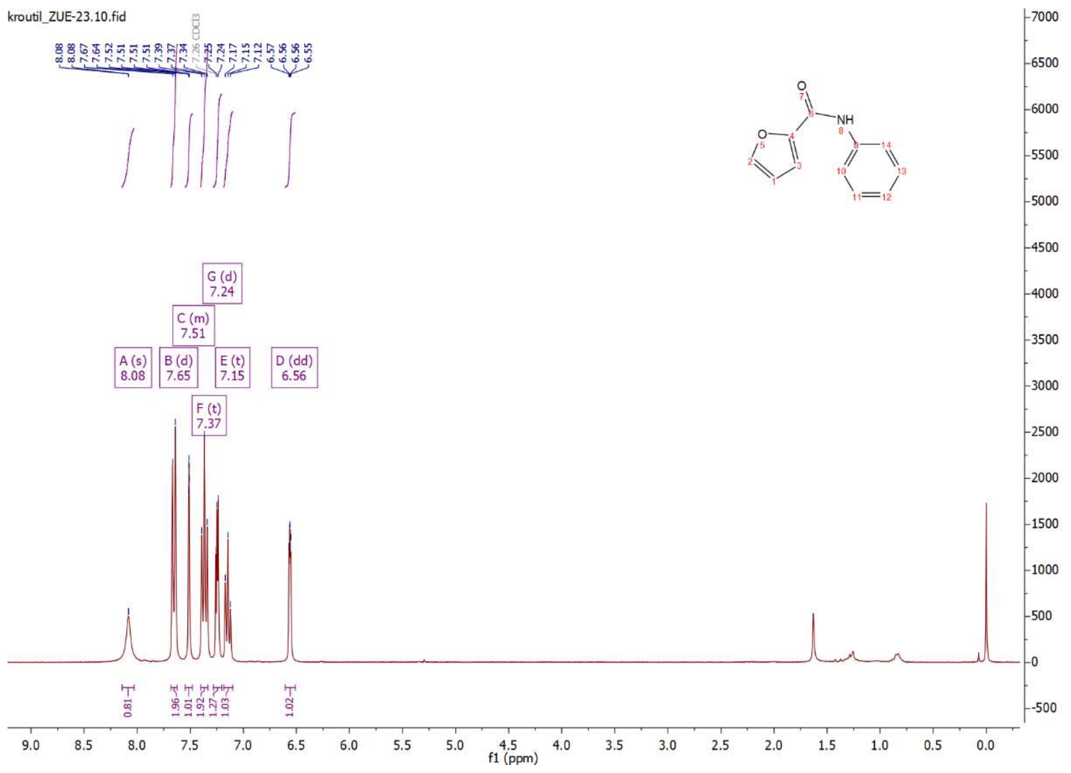

*Figure S50: ¹H-NMR of **3e,b** in CDCl₃.*

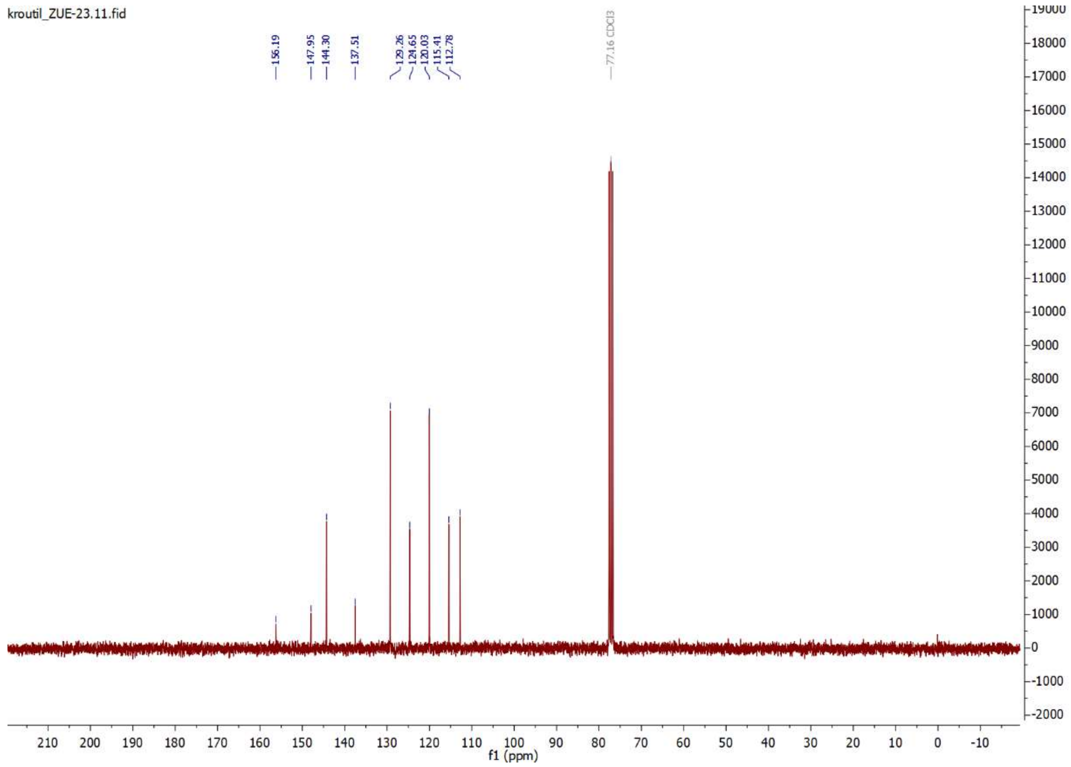

*Figure S51: ¹³C-NMR of **3e,b** in CDCl₃.*

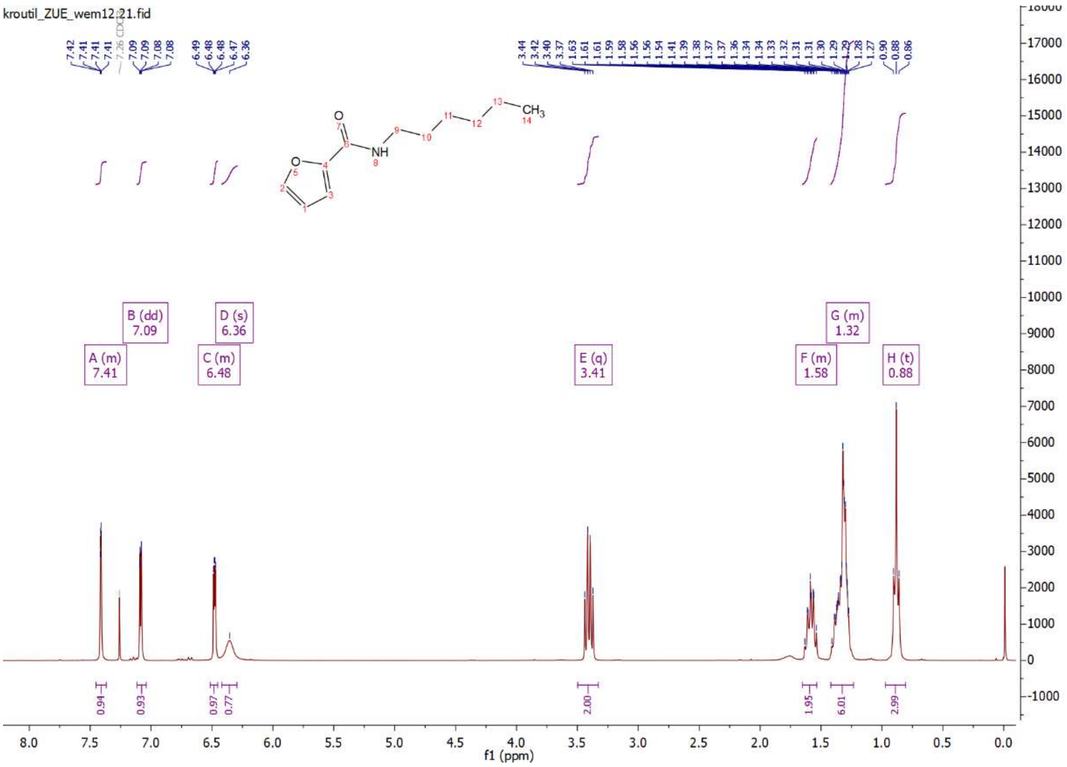

*Figure S52: ^{1}H-NMR of **3e,c** in $CDCl_3$.*

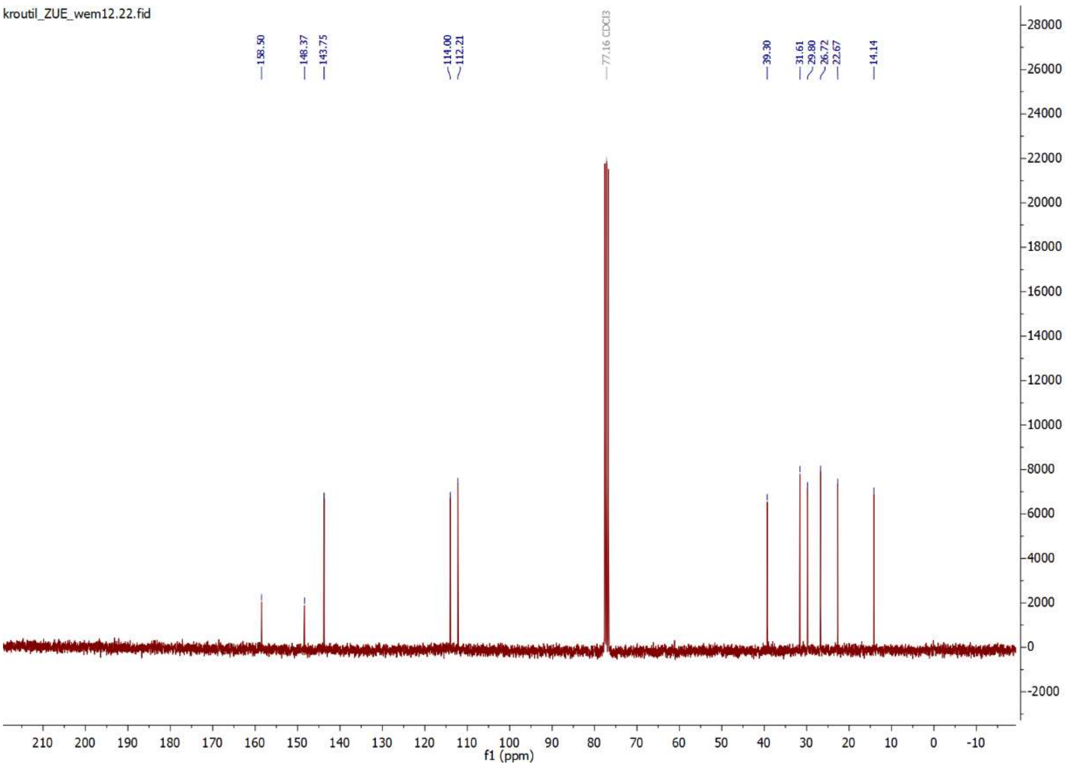

*Figure S53: ^{13}C-NMR of **3e,c** in $CDCl_3$.*

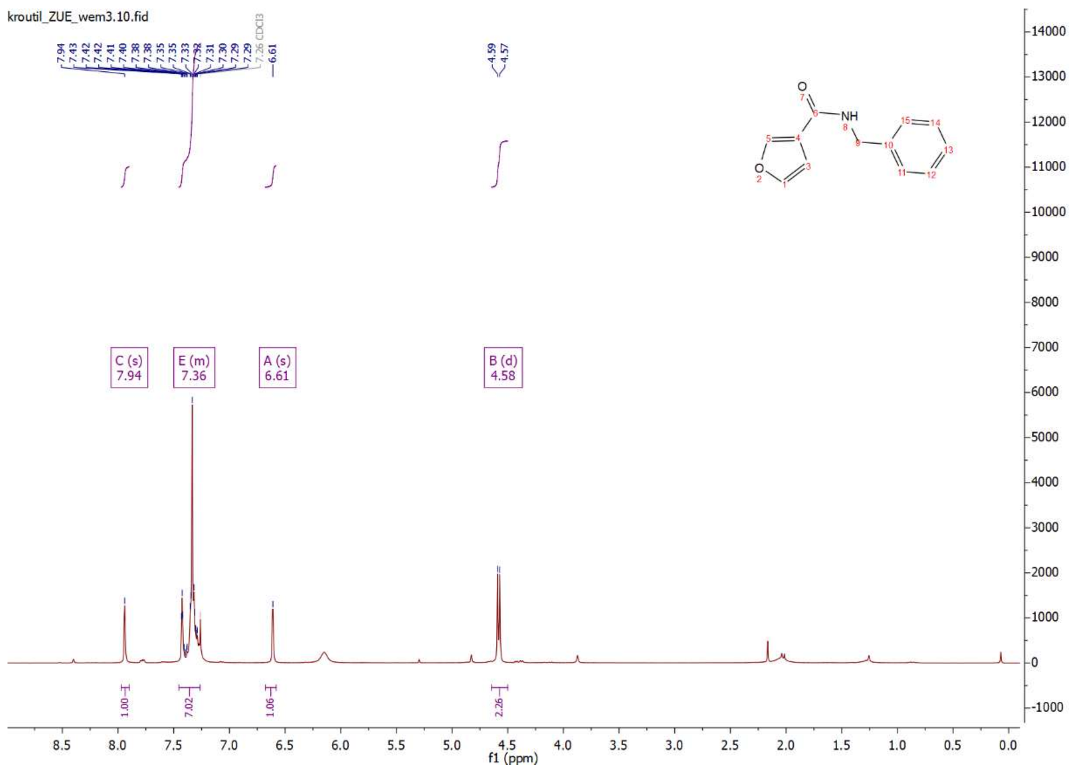

*Figure S54: ^{1}H-NMR of **3f,a** in CDCl$_3$.*

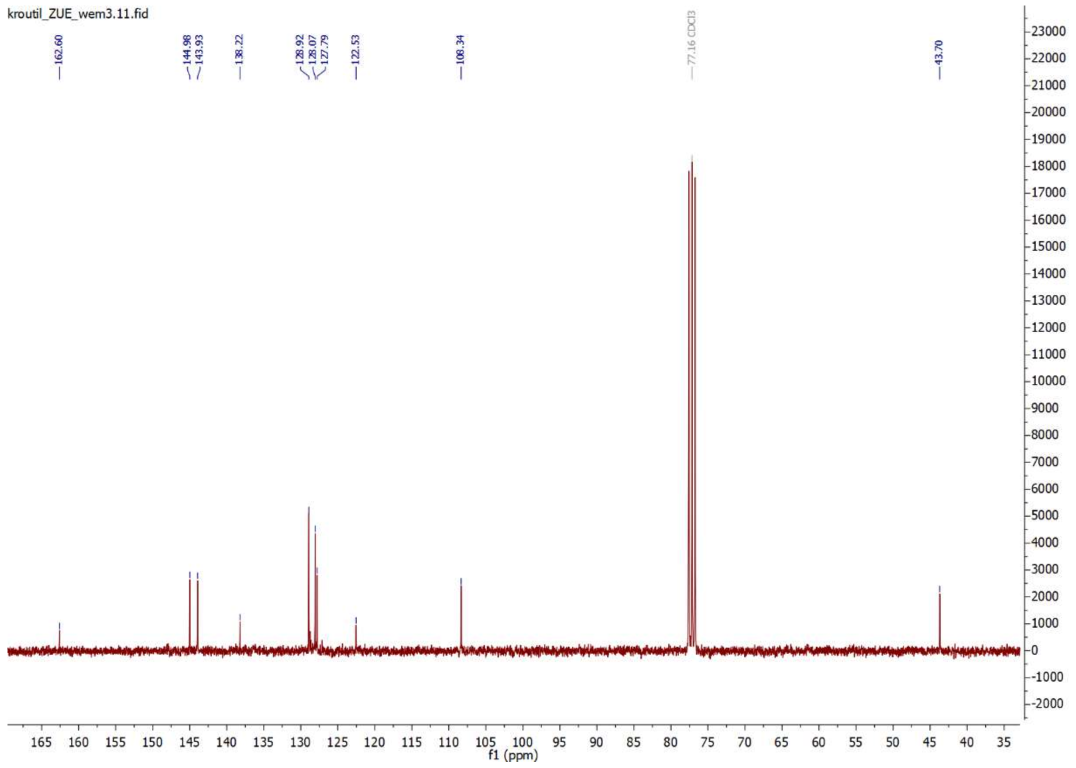

*Figure S55: ^{13}C-NMR of **3f,a** in CDCl$_3$.*

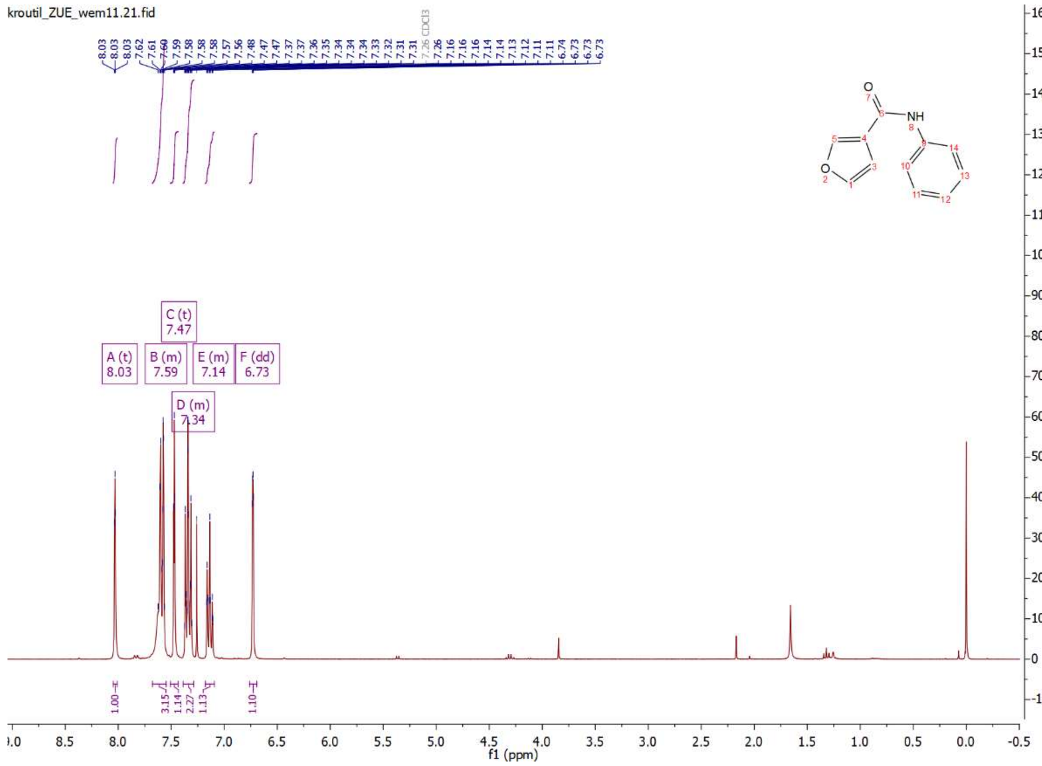

*Figure S56: ^{1}H-NMR of **3f,b** in $CDCl_3$.*

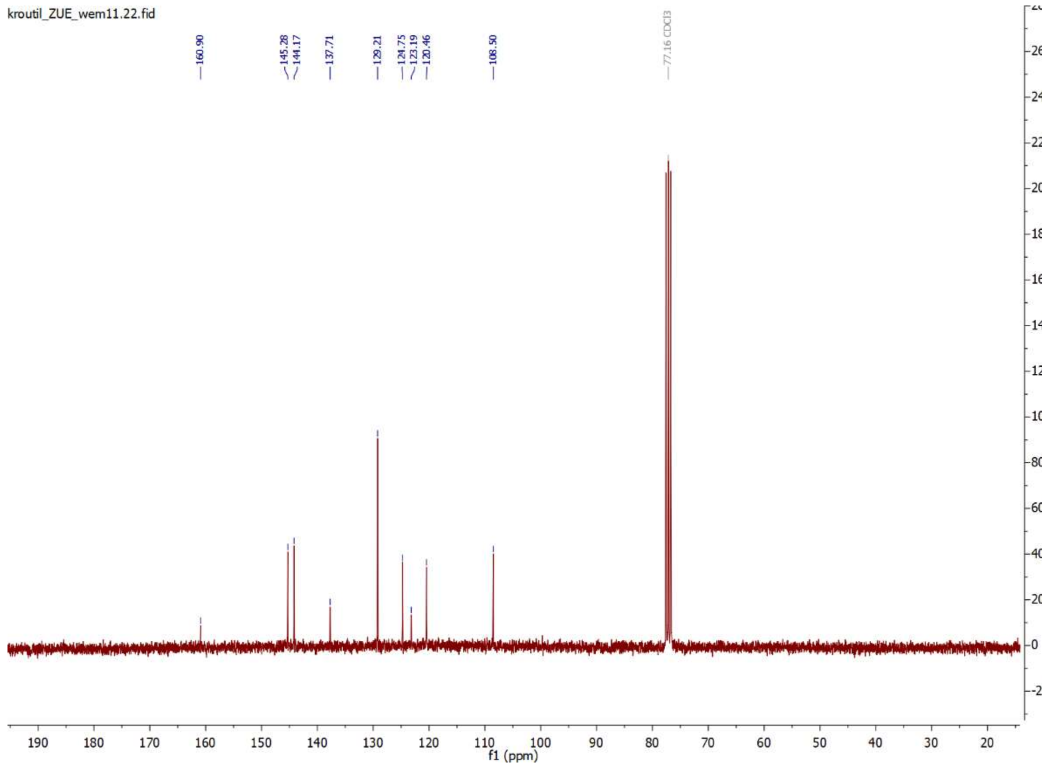

*Figure S57: ^{13}C-NMR of **3f,b** in $CDCl_3$.*

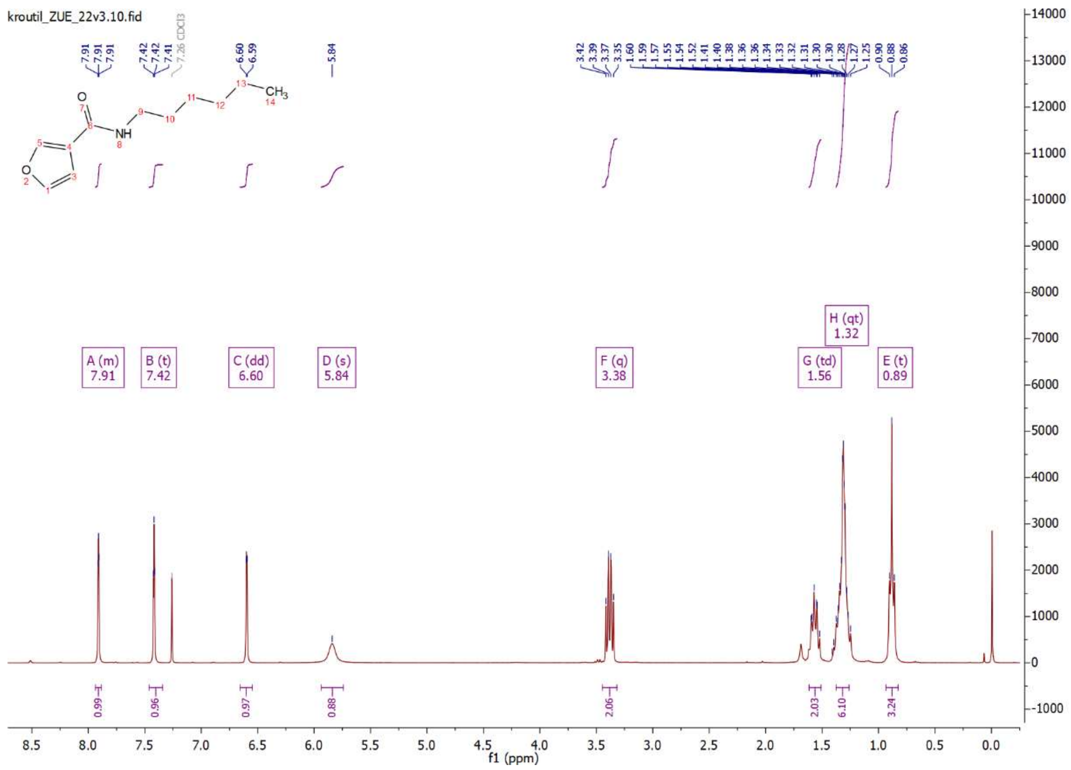

*Figure S58: ^{1}H-NMR of **3f,c** in $CDCl_3$.*

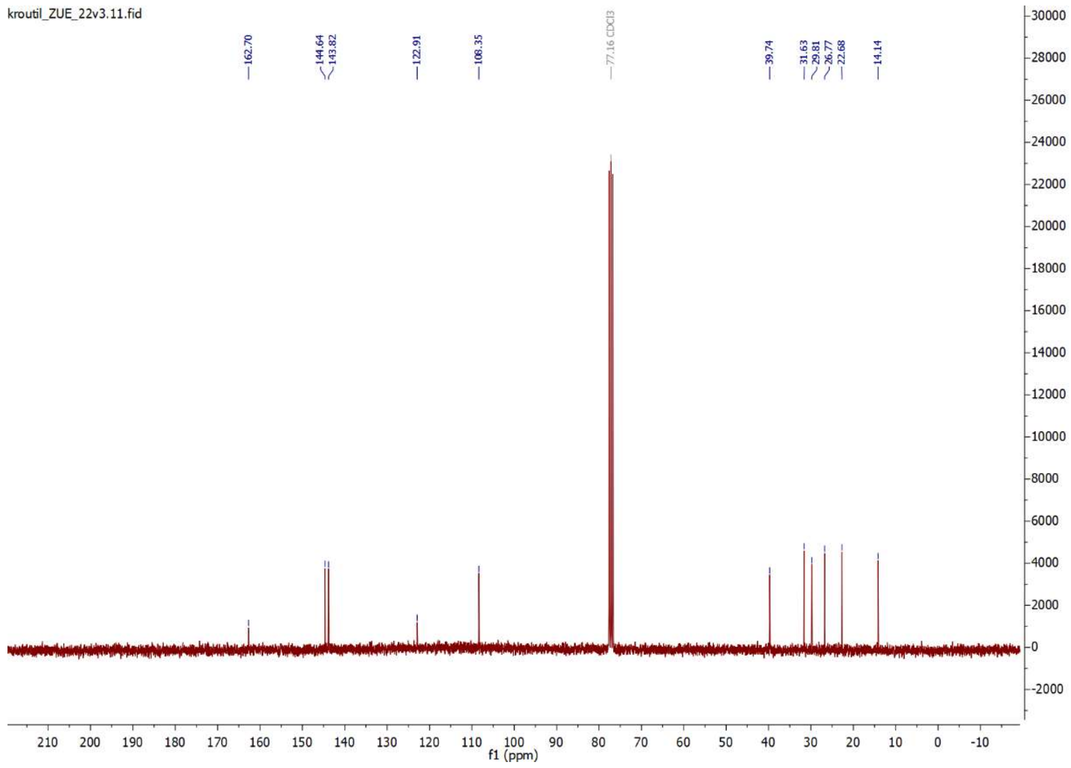

*Figure S59: ^{13}C-NMR of **3f,c** in $CDCl_3$.*

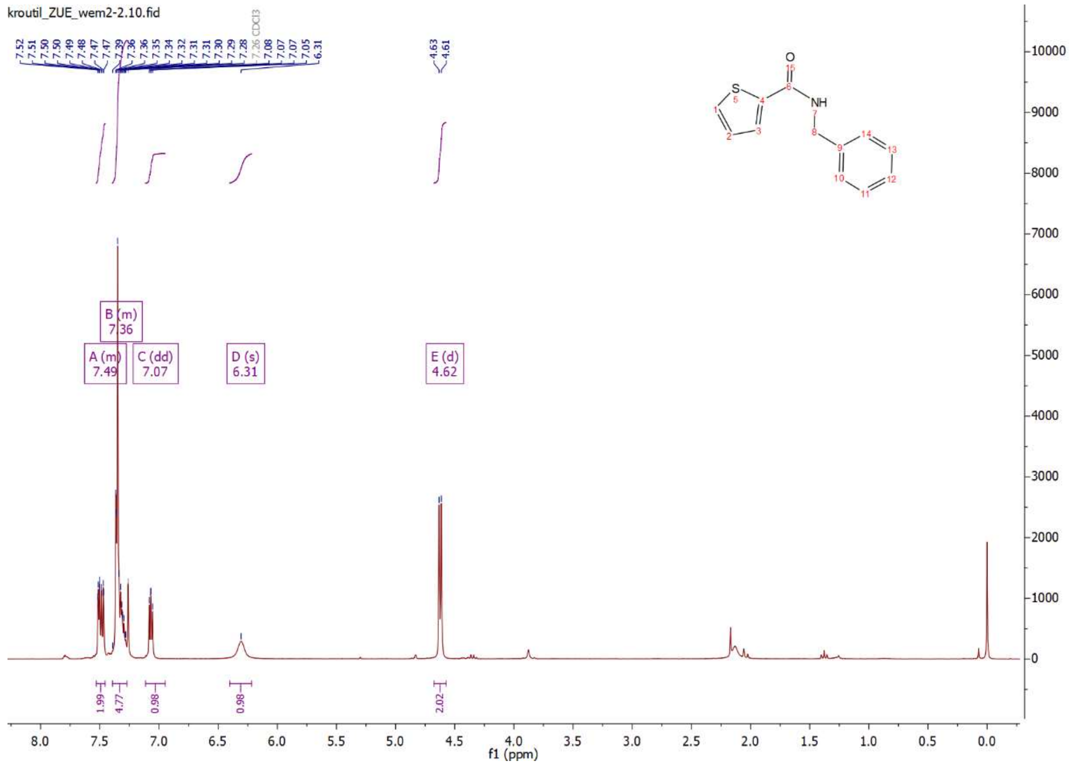

*Figure S60: ^{1}H-NMR of **3g,a** in $CDCl_3$.*

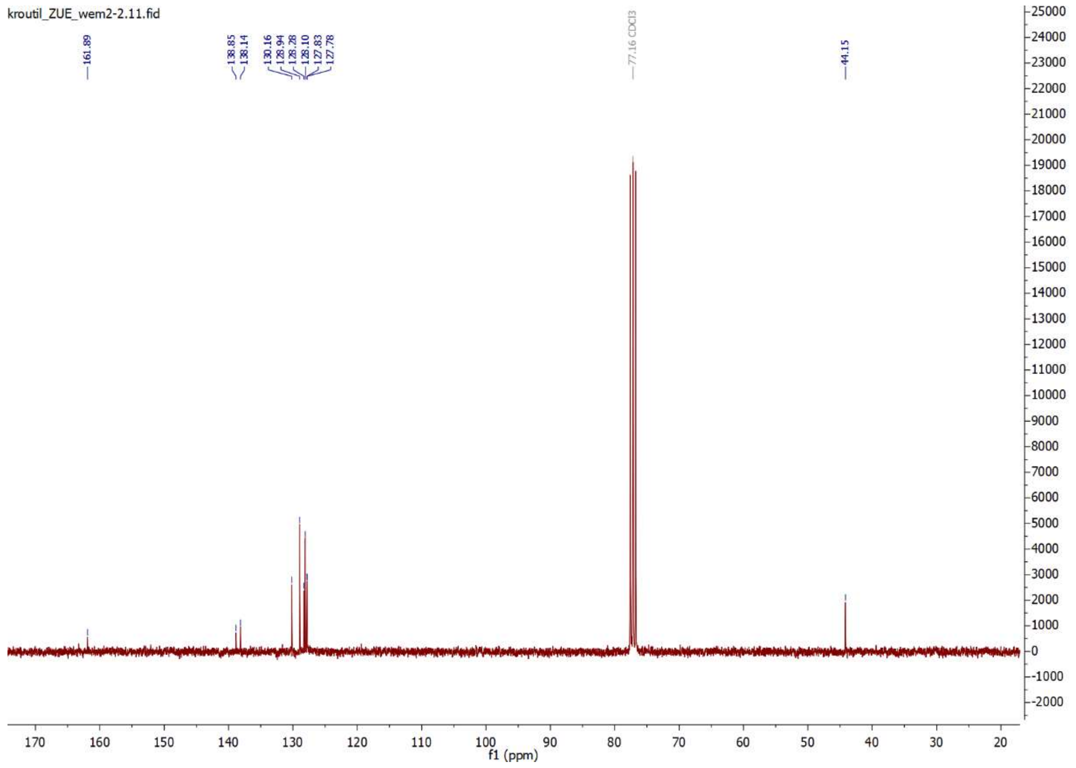

*Figure S61: ^{13}C-NMR of **3g,a** in $CDCl_3$.*

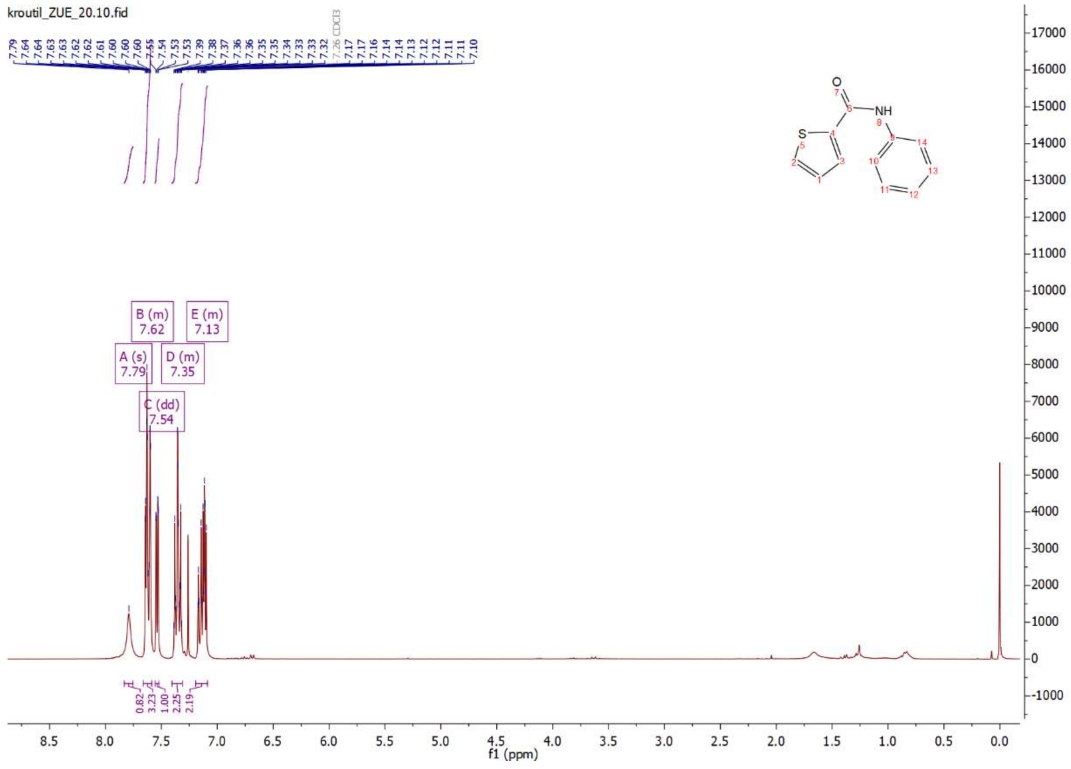

*Figure S62: ^{1}H-NMR of **3g,b** in CDCl$_3$.*

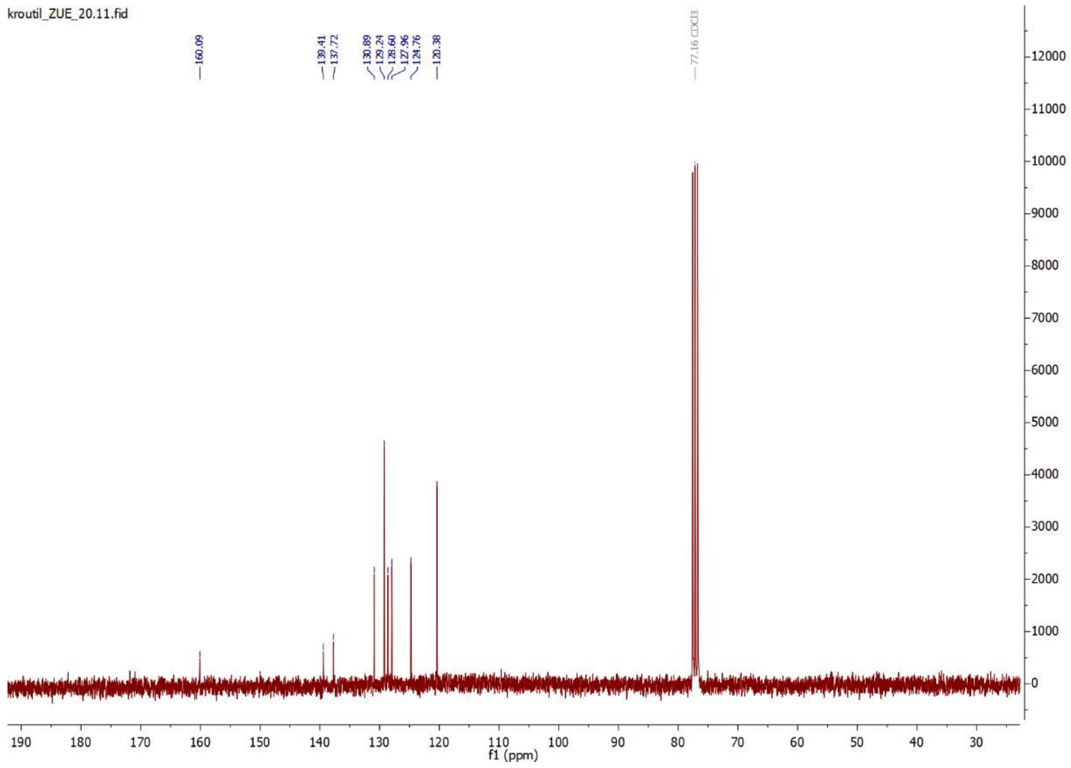

*Figure S63: ^{13}C-NMR of **3g,b** in CDCl$_3$.*

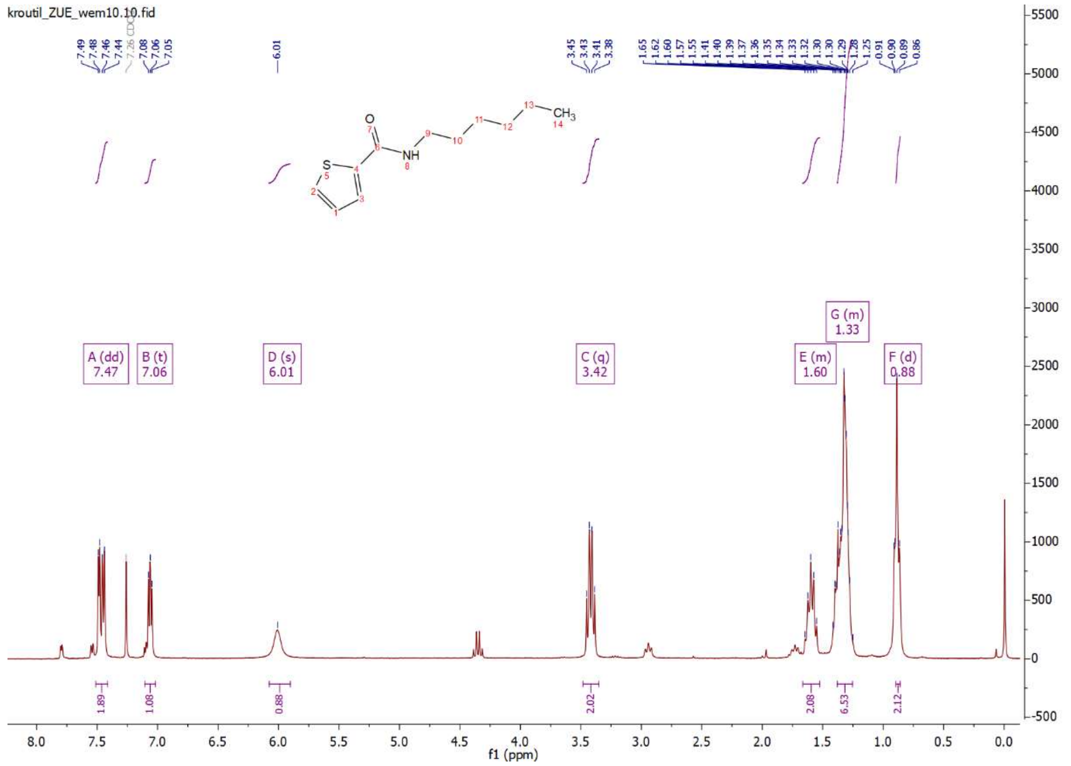

*Figure S64: ^{1}H-NMR of **3g,c** in $CDCl_3$.*

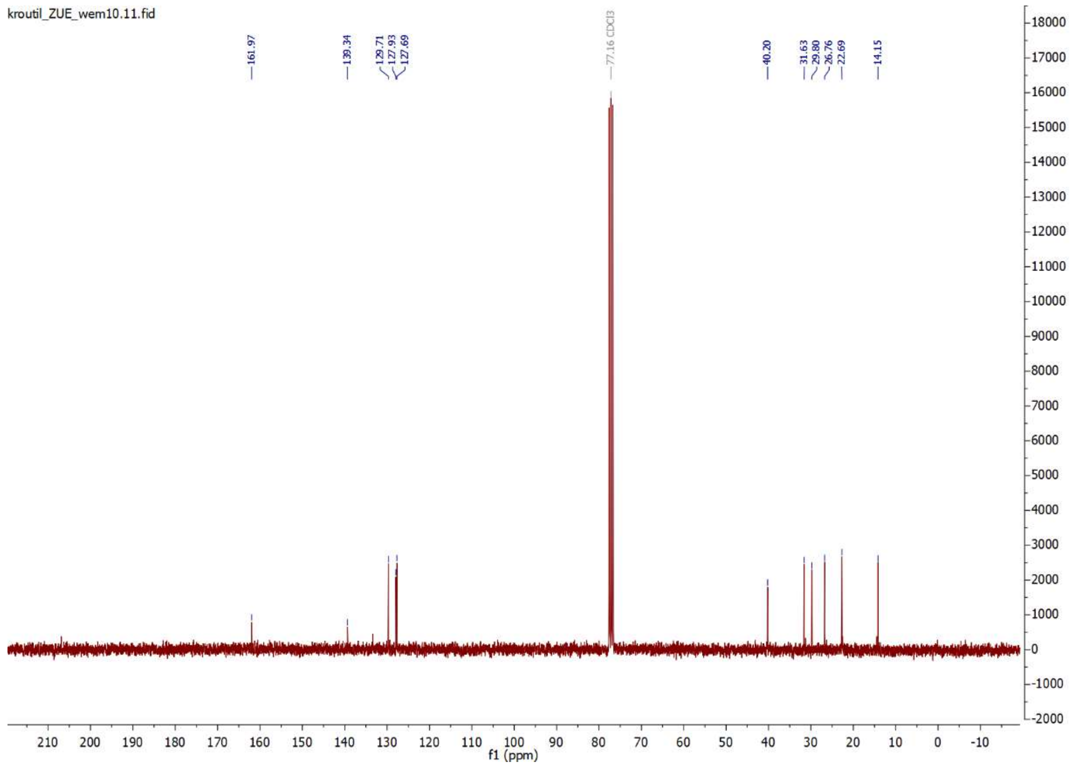

*Figure S65: ^{13}C-NMR of **3g,c** in $CDCl_3$.*

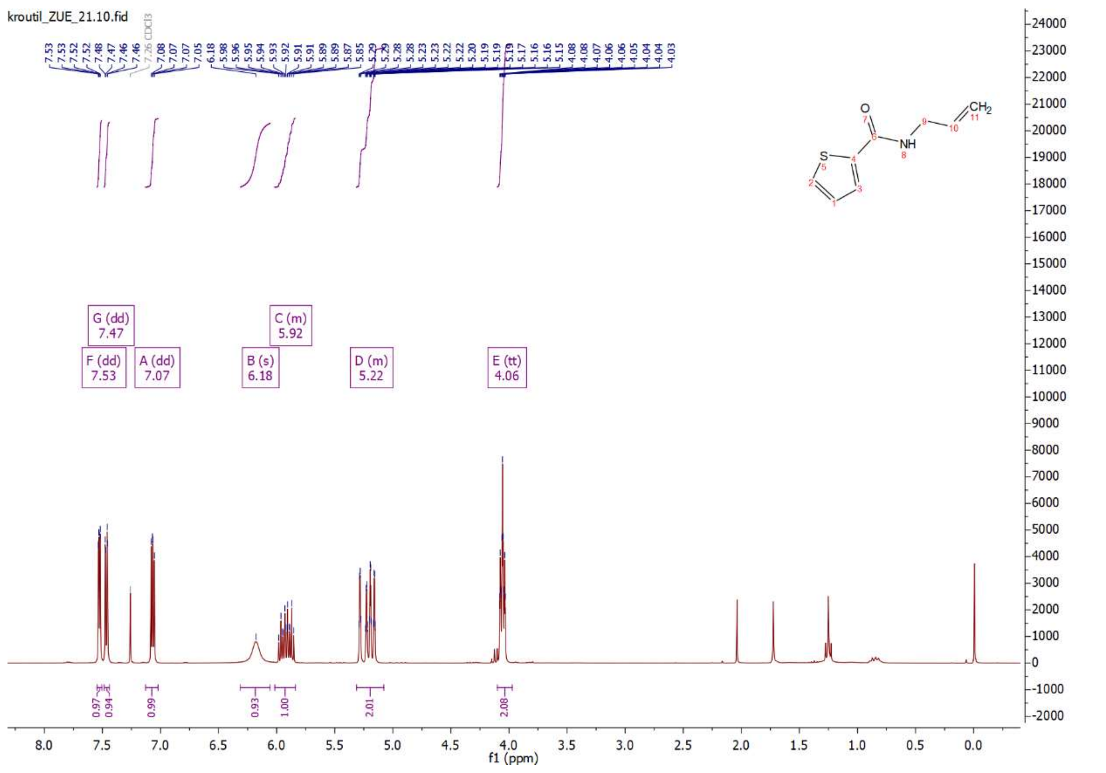

*Figure S66: ^{1}H-NMR of **3g,d** in $CDCl_3$.*

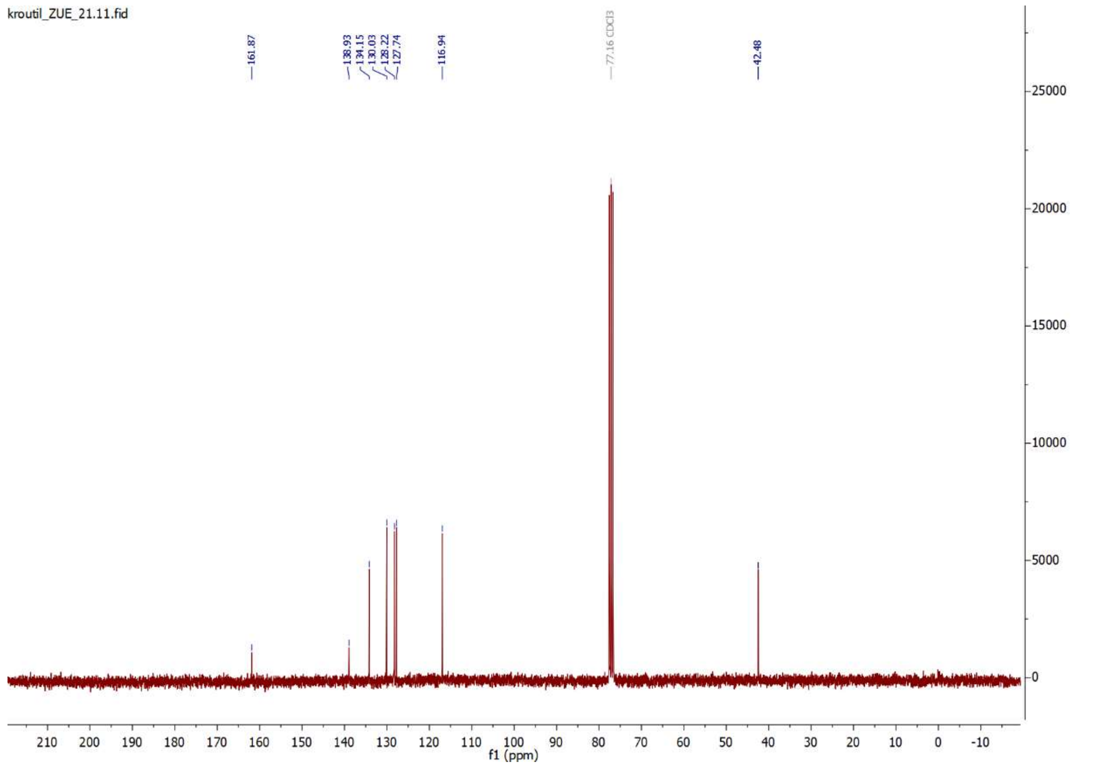

*Figure S67: ^{13}C-NMR of **3g,d** in $CDCl_3$.*

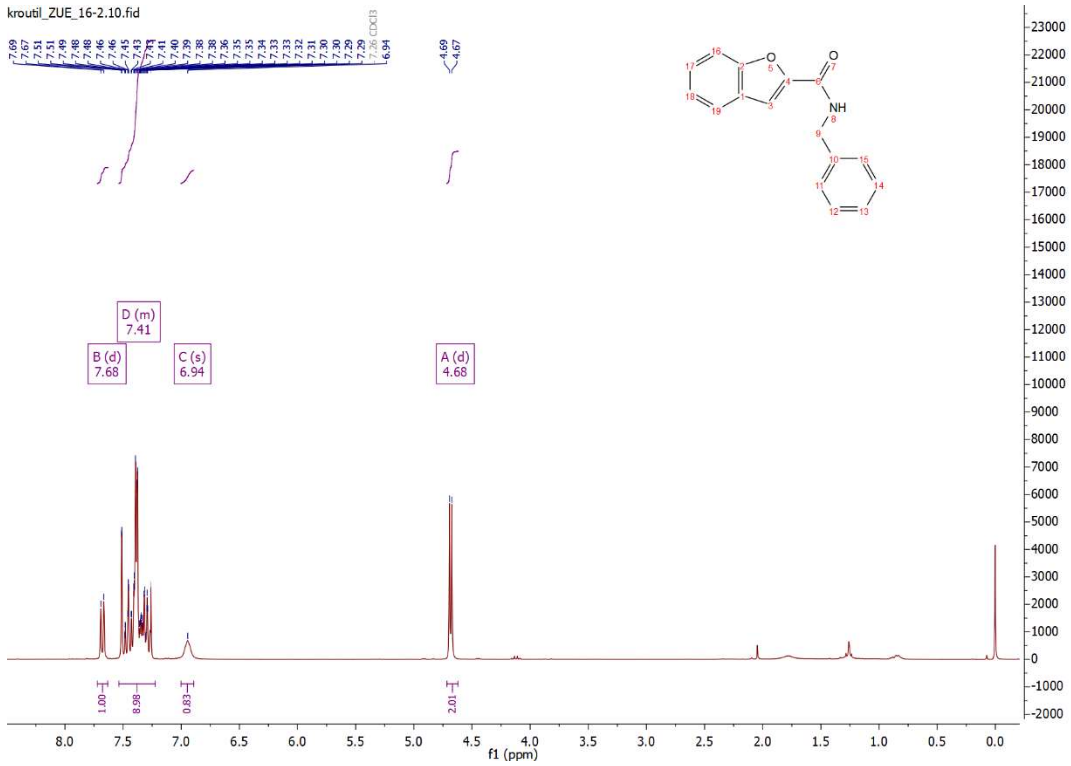

*Figure S68: ¹H-NMR of **3h,a** in $CDCl_3$.*

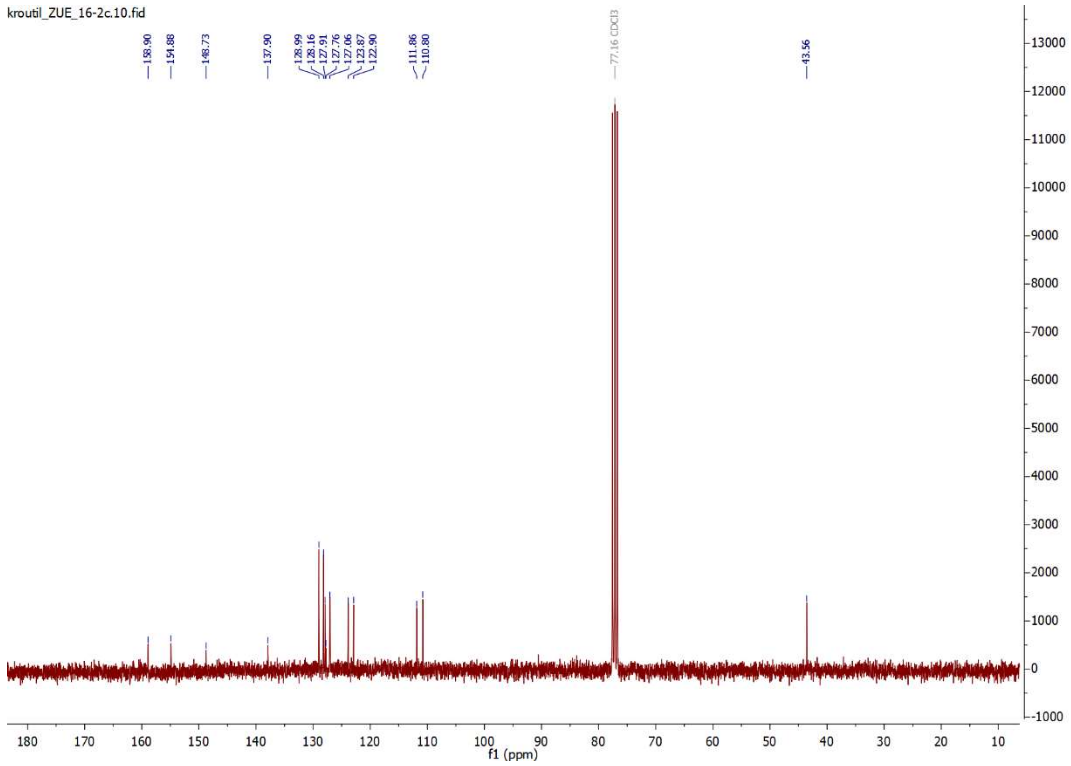

*Figure S69: ¹³C-NMR of **3h,a** in $CDCl_3$.*

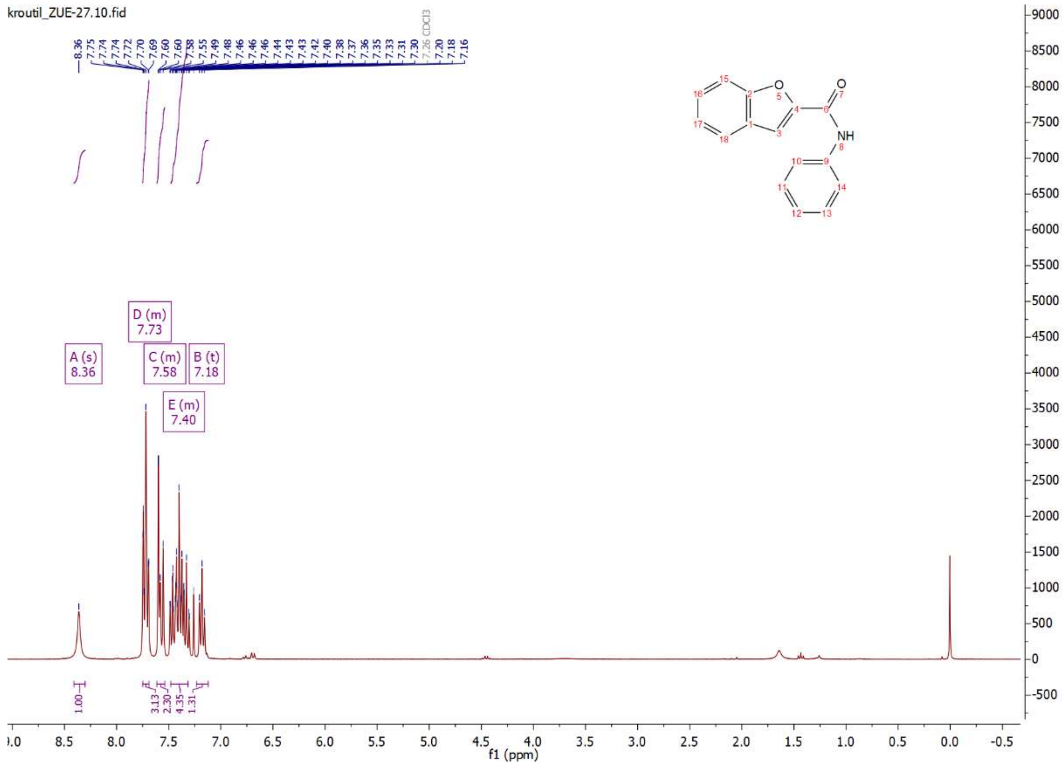

*Figure S70: ^{1}H-NMR of **3h,b** in $CDCl_3$.*

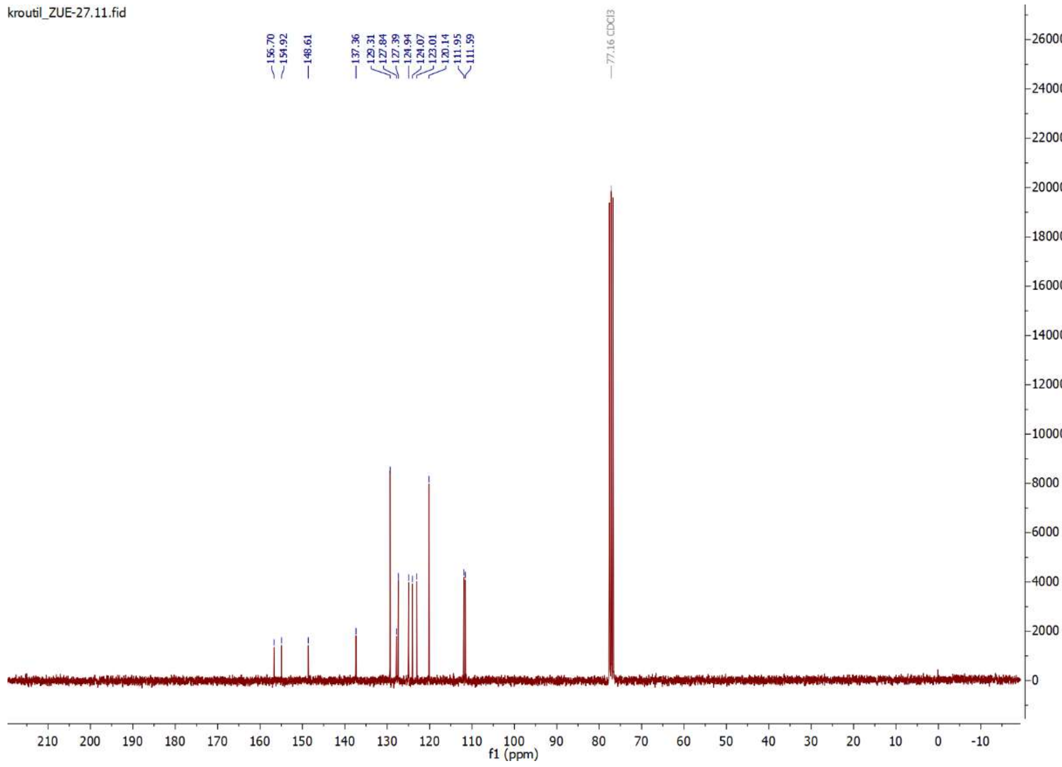

*Figure S71: ^{13}C-NMR of **3h,b** in $CDCl_3$.*

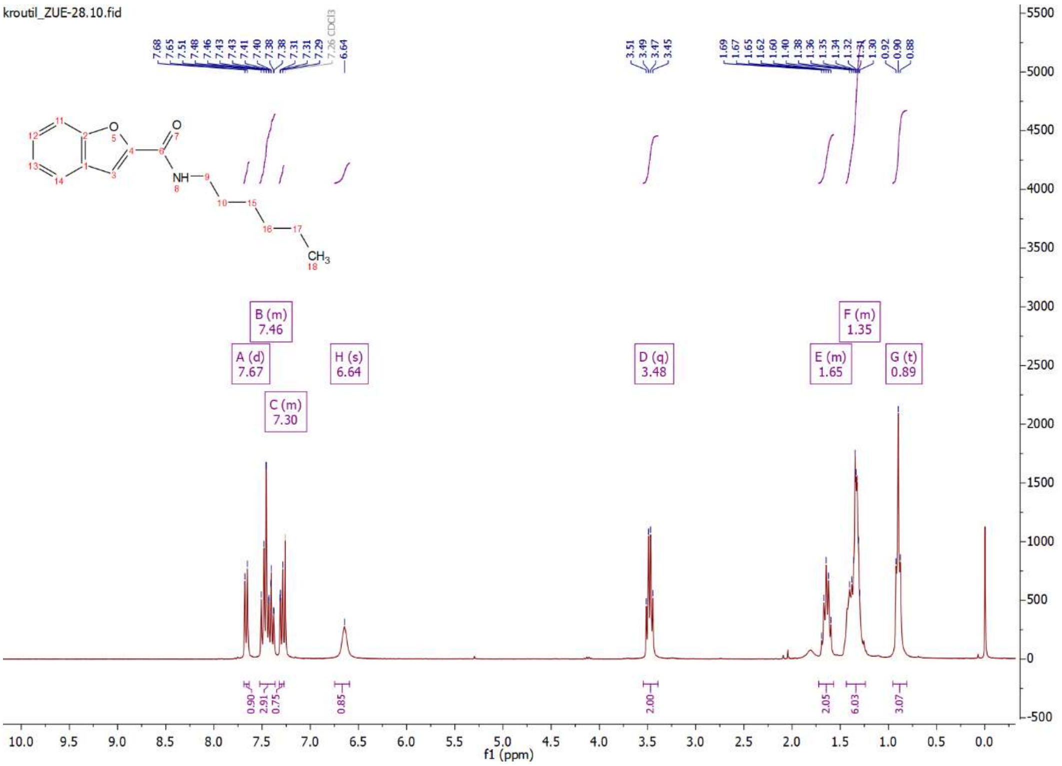

*Figure S72: ^{1}H-NMR of **3h,c** in $CDCl_3$.*

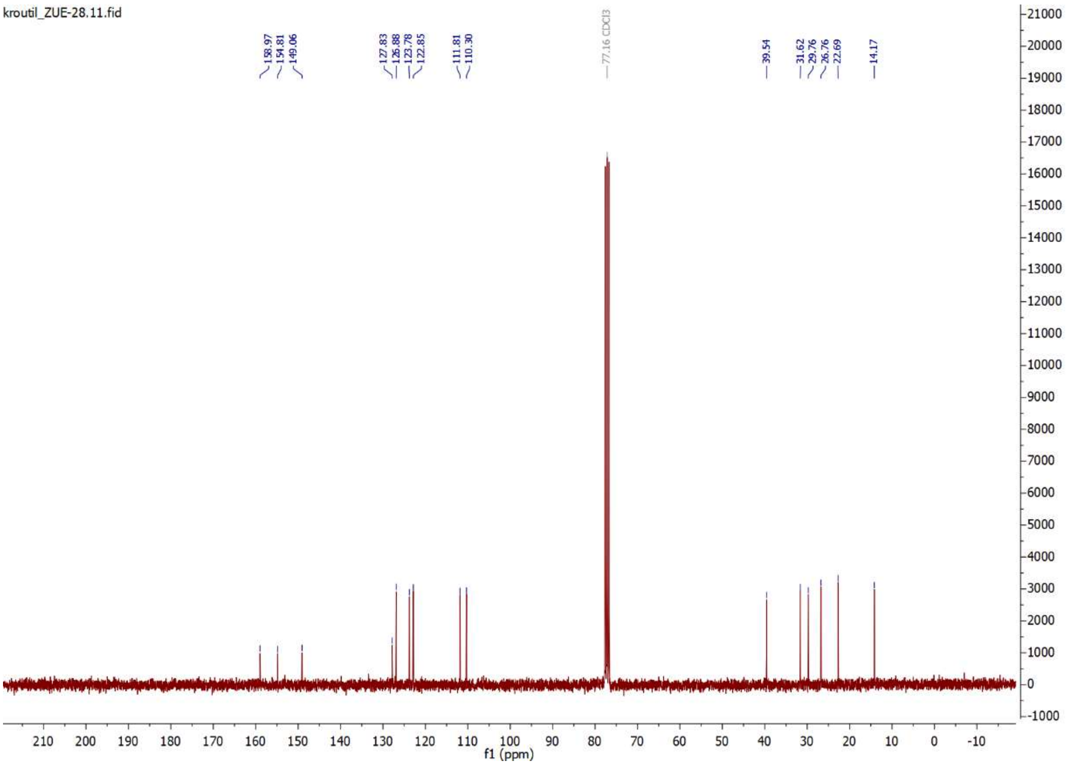

*Figure S73: ^{13}C-NMR of **3h,c** in $CDCl_3$.*

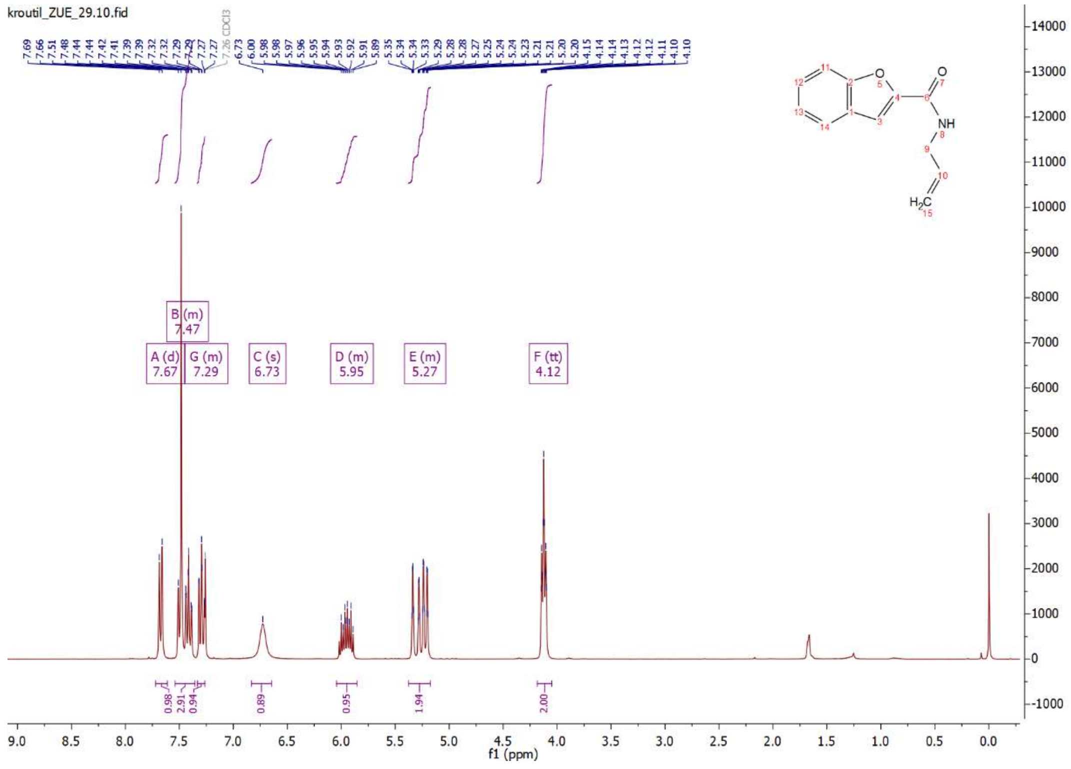

*Figure S74: ^{1}H-NMR of **3h,d** in CDCl₃.*

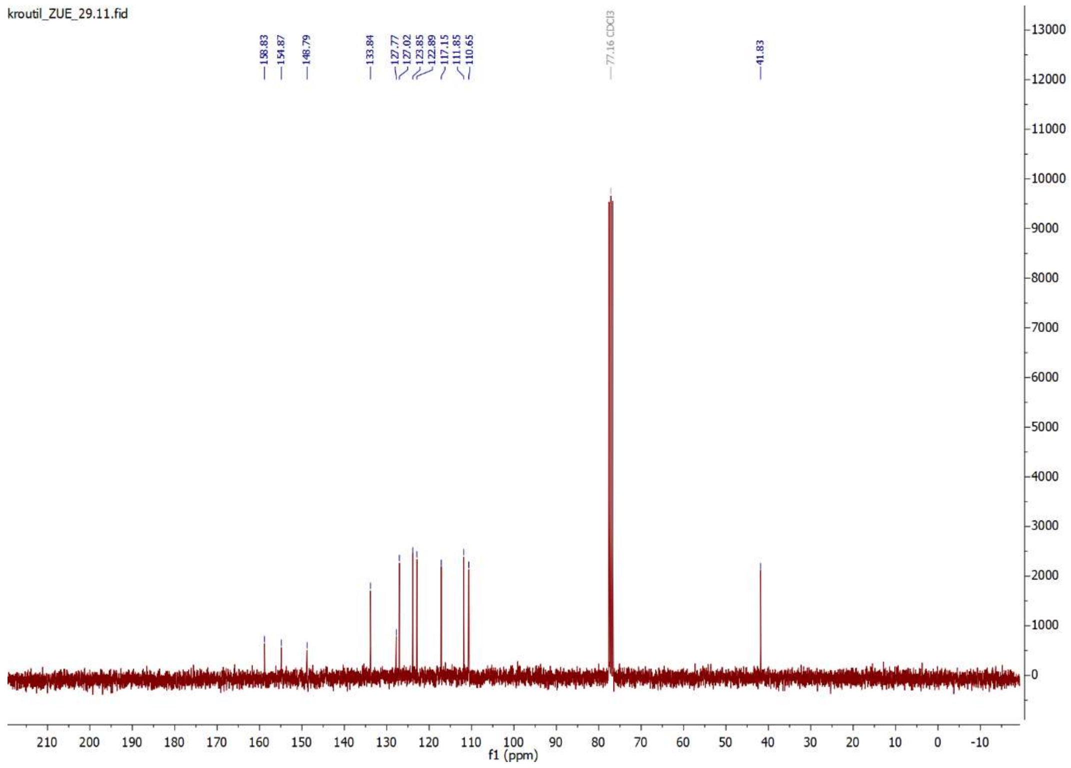

*Figure S75: ^{13}C-NMR of **3h,d** in CDCl₃.*

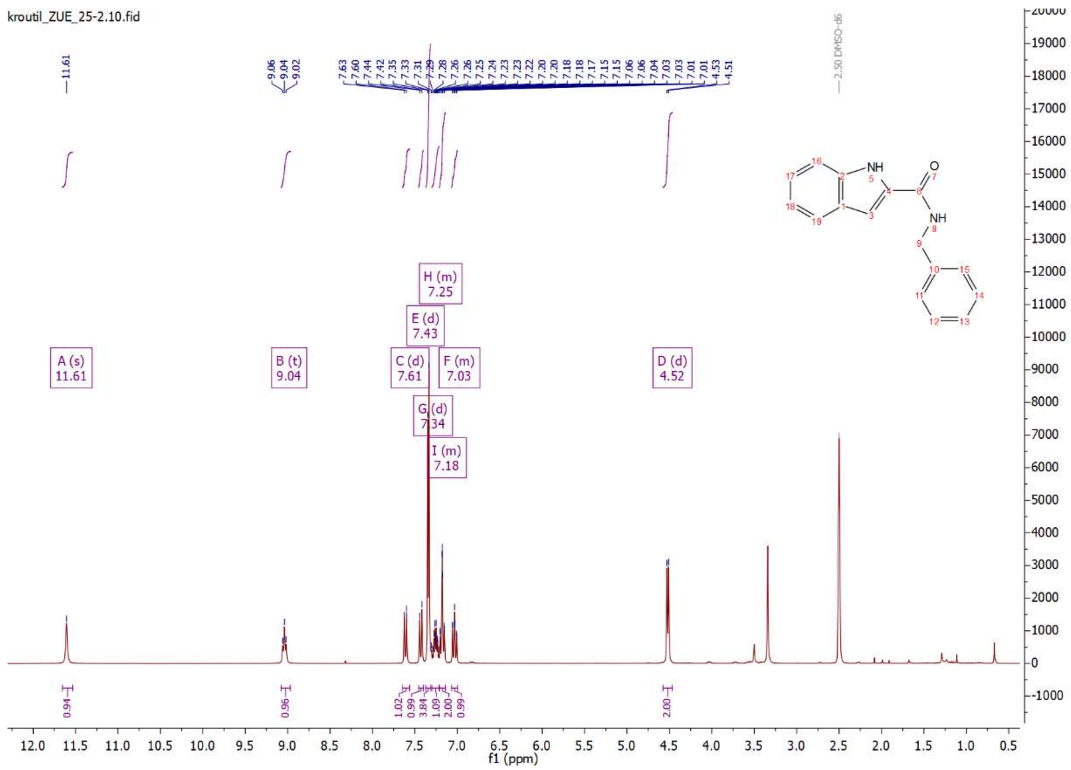

*Figure S76: ^{1}H-NMR of **3i,a** in CDCl₃.*

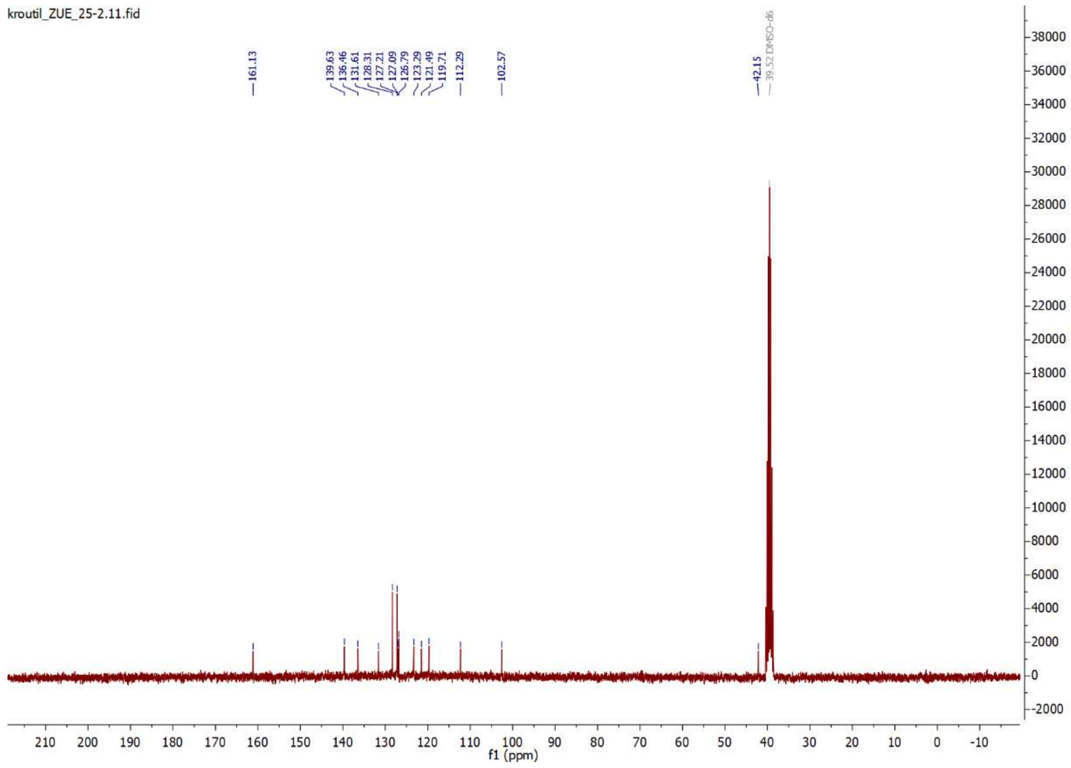

*Figure S77: ^{13}C-NMR of **3i,a** in CDCl₃.*

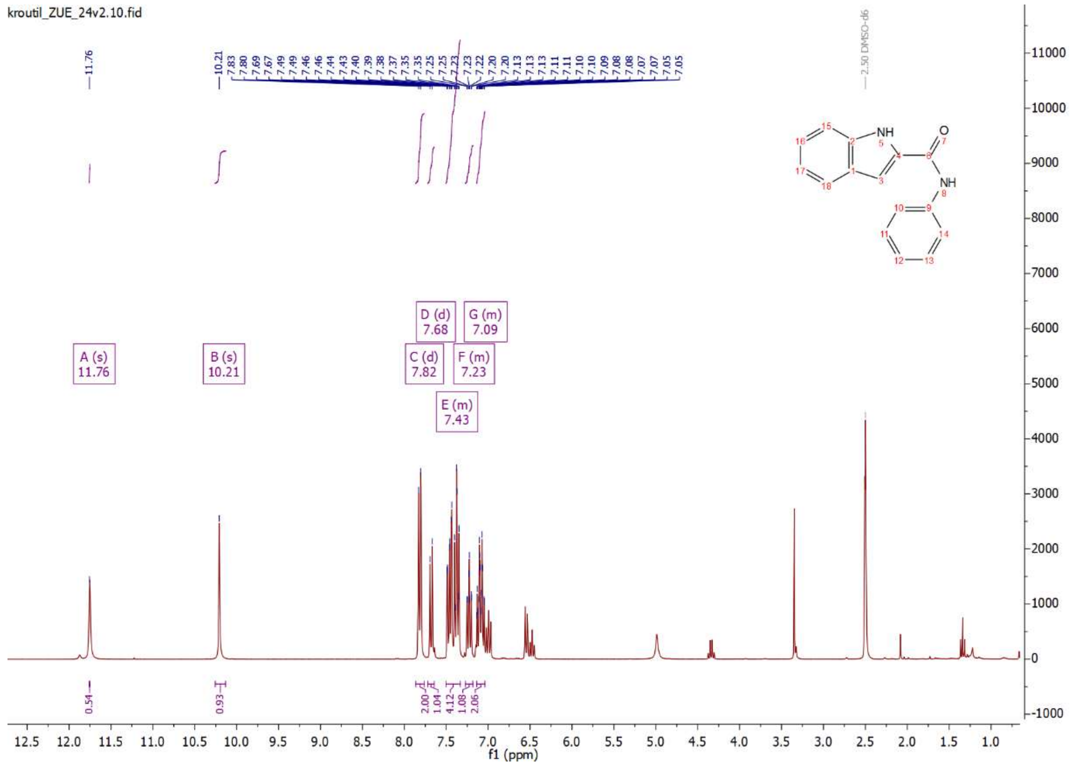

*Figure S78: ^{1}H-NMR of **3i,b** in CDCl3.*

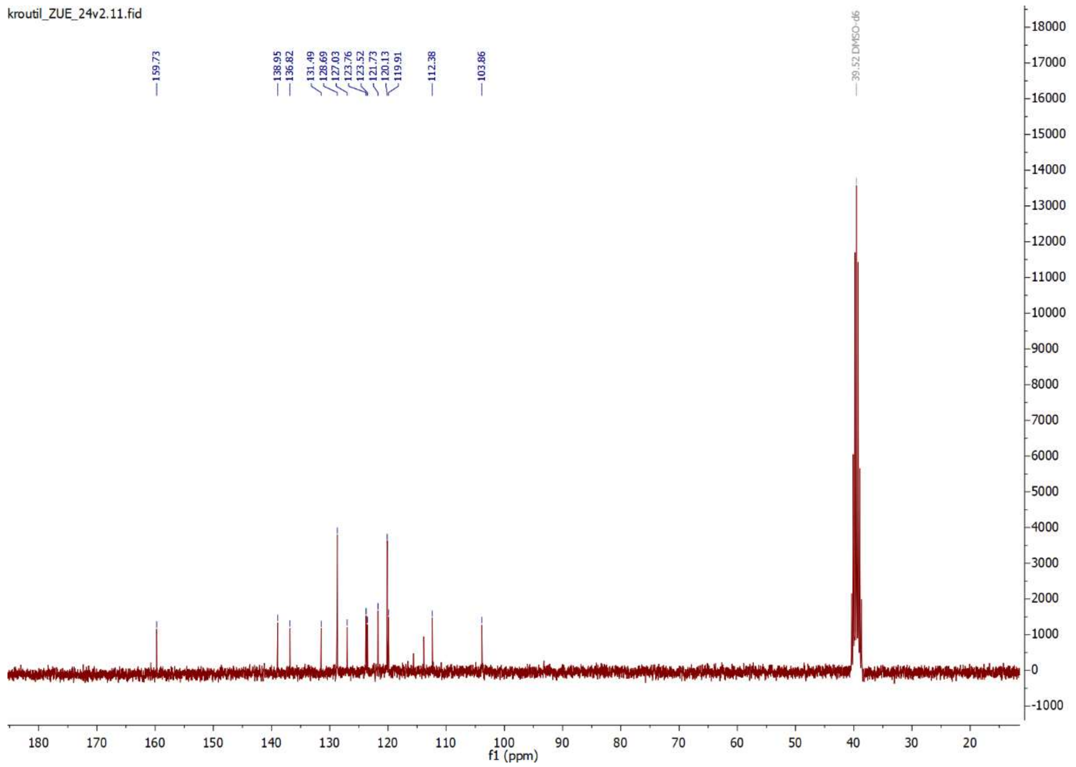

*Figure S79: ^{13}C-NMR of **3i,b** in CDCl3.*

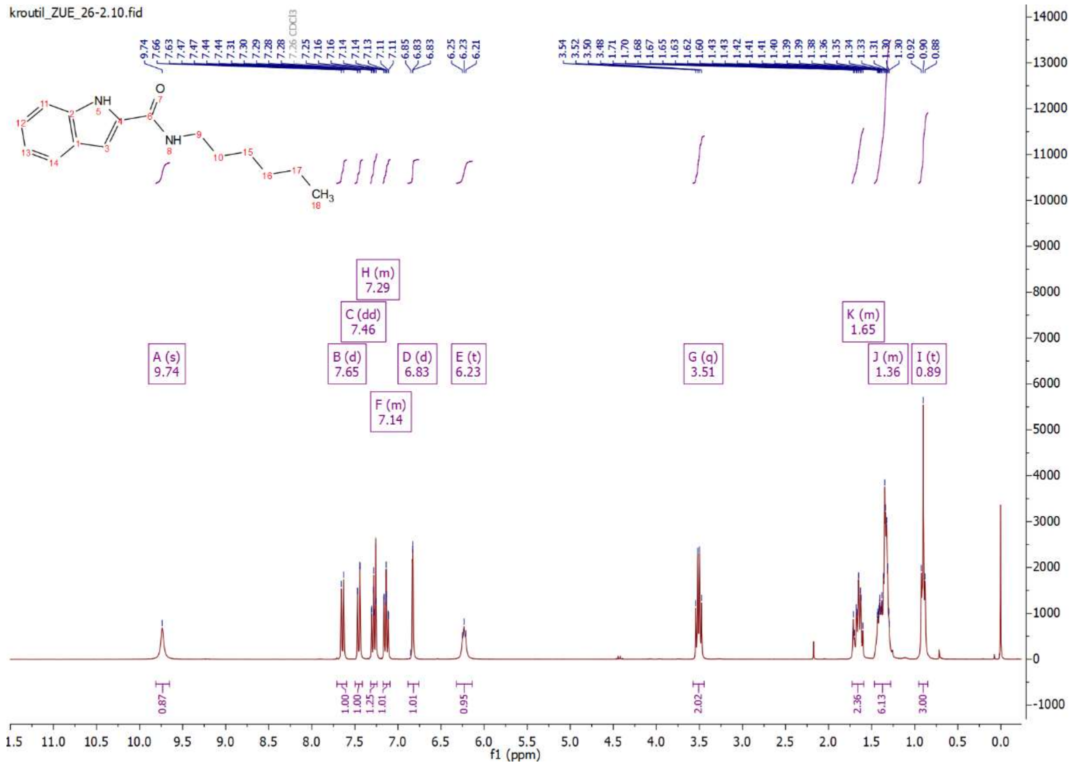

*Figure S80: ^{1}H-NMR of **3i,c** in $CDCl_3$.*

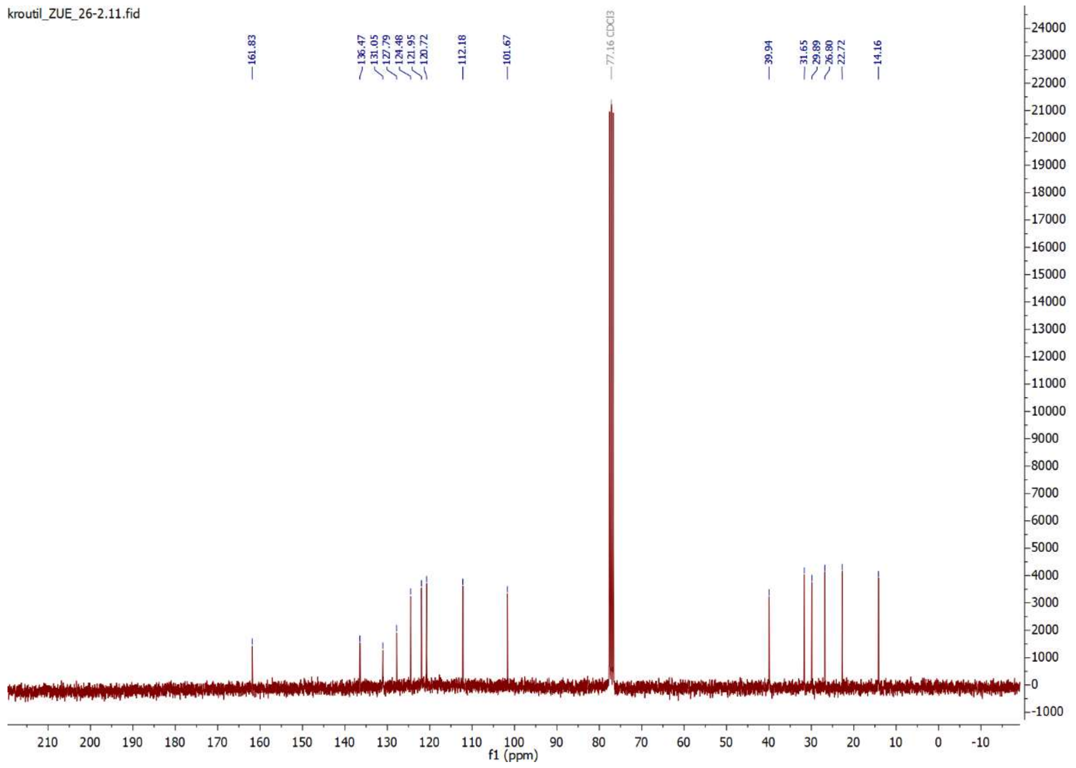

*Figure S81: ^{13}C-NMR of **3i,c** in $CDCl_3$.*

Buffer for protein purification

Table S15: Buffer „Lysis“ at pH 7.5

Component	Quantity added
300 mM NaCl	17.53 g
50 mM KH2PO4	6.80 g
20 mM imidazole	1.36 g
**dH_2O**	Up to 1 L

Table S16: Buffer „Elution“ at pH 7.5

Component	Quantity added
300 mM NaCl	17.53 g
50 mM KH2PO4	6.80 g
250 mM imidazole	17.02 g
**dH_2O**	Up to 1 L

Table S17: Buffer „Protein“ at pH 7.5

Component	Quantity added
150 mM NaCl	8.77 g
10 mM Tris*HCl	1.58 g
**dH_2O**	Up to 1 L

Medium preparation

Table S18: TB medium for 1 L.

Component	Quantity [g]
Tryptone	12
Yeast extract	24
Glycerol	5
**K_2HPO_4**	12.5
**KH_2PO_4**	2.3

Table S19: Autoinduction medium for 1 L.

Component	Quantity [g]
Tryptone	20
Yeast extract	5
Glycerol	6
**Na_2HPO_4**	6
**KH_2PO_4**	3
NaCl	5
Lactose	2
Glucose	0.5

Table S20: LB medium for 1 L.

Component	Quantity [g]
Tryptone	10
Yeast extract	5
NaCl	5

Used variants

Table S21: Used variants in this work.

Source organism	Name of the variant	Vector	Reference or source
Pseudozyma antarctica (C. antarctica)	CalB QW4	pET-22b(+)	[7]
Pseudozyma antarctica (C. antarctica)	CalB QW10	pET-22b(+)	[7]
Pseudozyma antarctica (C. antarctica)	CalB-24	pET-22b(+)	[19]
Pseudozyma antarctica (C. antarctica)	CalB-24 _T245S	pET-22b(+)	[19]
uncultured bacterium pCosCE1	EstCE1-W339Y	pET-24c(+)	[8]
uncultured bacterium pCosCE1	EstCE1-W339F	pET-24c(+)	[8]
Mycobacterium smegmatis	MsAcT-L12A/F154A	pET-28a(+)	[5]
Mycobacterium smegmatis	MsAcT-T93A/F154A	pET-28a(+)	[5]
Mycobacterium smegmatis	MsAcT-S11C	pET-28a(+)	[3]
Mycobacterium smegmatis	MsAcT-F150V/F174V	pET-28a(+)	[4]
Mycobacterium smegmatis	MsAcT- F174A/F154A	pET-28a(+)	[4]
Mycobacterium smegmatis	MsAcT-F154V/F174V	pET-28a(+)	[4]
Mycobacterium smegmatis	MsAcT-F150A/F154A	pET-28a(+)	[4]
Mycobacterium smegmatis	MsAcT-F150V/F154V	pET-28a(+)	[4]
Mycobacterium smegmatis	MsAcT-F174A/F150A	pET-28a(+)	[4]
Mycobacterium smegmatis	MsAcT-F174A	pET-28a(+)	[4]
Mycobacterium smegmatis	MsAcT-F154L	pET-28a(+)	[4]
Mycobacterium smegmatis	MsAcT-F154V	pET-28a(+)	[4]
Mycobacterium smegmatis	MsAcT-F154A	pET-28a(+)	[4]
Mycobacterium smegmatis	MsAcT-F174V	pET-28a(+)	[4]
Sphingomonas sp. HXN-200	SpLH102V	pRSFDuet-1	This work
Sphingomonas sp. HXN-200	SpLH102Y	pRSFDuet-1	This work
Sphingomonas sp. HXN-200	SpLH102N	pRSFDuet-1	This work
Sphingomonas sp. HXN-200	SpLH102S	pRSFDuet-1	This work
Sphingomonas sp. HXN-200	SpLD158E	pRSFDuet-1	This work
Sphingomonas sp. HXN-200	SpLD158I	pRSFDuet-1	This work
Sphingomonas sp. HXN-200	SpLD158Q	pRSFDuet-1	This work
Sphingomonas sp. HXN-200	SpLD158K	pRSFDuet-1	This work
Sphingomonas sp. HXN-200	SpLD158H	pRSFDuet-1	This work
Sphingomonas sp. HXN-200	SpLD158T	pRSFDuet-1	This work
Sphingomonas sp. HXN-200	SpLD158S	pRSFDuet-1	This work
Sphingomonas sp. HXN-200	SpLD158R	pRSFDuet-1	This work
Sphingomonas sp. HXN-200	SpLD158N	pRSFDuet-1	This work
Sphingomonas sp. HXN-200	SpLD158Y	pRSFDuet-1	This work

QW4 - W104V/S105C/A281Y/A282Y/V149G
QW10 - W104V/A281Y/A282Y/V149G
CalB24 - T57A/A89T/G226R/R168K

Sequences

DNA Sequence SpL wt

ATGACCGACAGCACGACCCATTACACTCGCCCCGACGTCGCGGCATTTCTGGCCTTCCTGAATGCGCAGGAGGGG
CCGAAGATGGAGGAAATGCCTCCTGCGGGCGCGCGCGAGATGATGCGTGTGATGGGCCAGCTCGCCGACGTACCG
CGCGGCGAGATCGCGAAGGTCGAGGACCGCATGATCCCGGGCCCGGATGGTGACATTCCCATCCGCCTCTACGAC
AATCGCCCCGATCGCGAAGCGGGTCCGGTGATGGTCTTCTATCACGGCGGCGGCTGGGTGATCGGCGACCTCGAA
ACGCACGATCCCTATTGCGCCGAAGCCGCGCGCATCCTCGACATGCCGGTCATCGCGATCGATTATCGCCTCGCC
CCCGAACATCCCTTCCCCGCCGCCCCGATCGACTGCGAGGCCGCAACGCGCTGGGTCGCCGACAATATTGCCTGC
ACGGGTCTGGTCCTGTCGGGCGACAGCGCGGGCGGCAATTTGACCATCGTGACGGCGTTGGCGCTGCGCGACGAA
CCGGCAGCGAAGCCGGTGATCGCGATCCATCCCATCTATCCCGCCGTCACGACGCACAACGACTGGCAAAGCTAT
CGCGATTTCGGCGAGGGTCATTTGCTGACCGAAGGCAGCATGACCTGGTTCGGCAATCATTATGCGGCCGACCCC
GCCGACCGCCGCGCCGCGCCGATCGACTTTCCCGCCGACGGCCTGCCGCCGACGCTGCTGATCACCGCCAGCCTC
GACCCGCTGCGCGATCAGGGCCGCGCCTATGCCGCCAAGCTGATCGAAGCGGGTGTACCGACGACCTACCGCGAG
GCGAAAGGTACGATCCACGGCTATATCTGCCTCGCGCAGGGCATCCCCAGCGCCAAGGACGACATTCGCGGCGCA
TTGACGGTCTTGAAGGCGATCGTCGCCGAGGCTACCGGAGCGGCA

Amino Acid Sequence SpL wt

MTDSTTHYTRPDVAAFLAFLNAQEGPKMEEMPPAGAREMMRVMGQLADVPRGEIAKVEDRMIPGPDGDIPIRLYD
NRPDREAGPVMVFYHGGGWVIGDLETHDPYCAEAARILDMPVIAIDYRLAPEHPFPAAPIDCEAATRWVADNIAC
TGLVLSGDSAGGNLTIVTALALRDEPAAKPVIAIHPIYPAVTTHNDWQSYRDFGEGHLLTEGSMTWFGNHYAADP

ADRRAAPIDFPADGLPPTLLITASLDPLRDQGRAYAAKLIEAGVPTTYREAKGTIHGYICLAQGIPSAKDDIRGA
LTVLKAIVAEATGAA

DNA Sequence CalB wt

ATGAAATACCTGCTGCCGACCGCTGCTGCTGGTCTGCTGCTCCTCGCTGCCCAGCCGGCGATGGCCCTACCTTCC
GGTTCGGACCCTGCCTTTTCGCAGCCCAAGTCGGTGCTCGATGCGGGTCTGACCTGCCAGGGTGCTTCGCCATCC
TCGGTCTCCAAACCCATCCTTCTCGTCCCCGGAACCGGCACCACAGGTCCACAGTCGTTCGACTCGAACTGGATC
CCCCTCTCAACGCAGTTGGGTTACACACCCTGCTGGATCTCACCCCCGCCGTTCATGCTCAACGACACCCAGGTC
AACACGGAGTACATGGTCAACGCCATCACCGCGCTCTACGCTGGTTCGGGCAACAACAAGCTTCCCGTGCTTACC
TGGTCCCAGGGTGGTCTGGTTGCACAGTGGGGTCTGACCTTCTTCCCCAGTATCAGGTCCAAGGTCGATCGACTT
ATGGCCTTTGCGCCCGACTACAAGGGCACCGTCCTCGCCGGCCCTCTCGATGCACTCGCGGTTAGTGCACCCTCC
GTATGGCAGCAAACCACCGGTTCGGCACTCACCACCGCACTCCGAAACGCAGGTGGTCTGACCCAGATCGTGCCC
ACCACCAACCTCTACTCGGCGACCGACGAGATCGTTCAGCCTCAGGTGTCCAACTCGCCACTCGACTCATCCTAC
CTCTTCAACGGAAAGAACGTCCAGGCACAGGCCGTGTGTGGGCCGCTGTTCGTCATCGACCATGCAGGCTCGCTC
ACCTCGCAGTTCTCCTACGTCGTCGGTCGATCCGCCCTGCGCTCCACCACGGGCCAGGCTCGTAGTGCAGACTAT
GGCATTACGGACTGCAACCCTCTTCCCGCCAATGATCTGACTCCCGAGCAAAAGGTCGCCGCGGCTGCGCTCCTG
GCGCCGGCAGCTGCAGCCATCGTGGCGGGTCCAAAGCAGAACTGCGAGCCCGACCTCATGCCCTACGCCCGCCCC
TTTGCAGTAGGCAAAAGGACCTGCTCCGGCATCGTCACCCCC

Amino Acid Sequence CalB wt

MKYLLPTAAAGLLLLAAQPAMALPSGSDPAFSQPKSVLDAGLTCQGASPSSVSKPILLVPGTGTTGPQ
SFDSNWIPLSTQLGYTPCWISPPPFMLNDTQVNTEYMVNAITALYAGSGNNKLPVLTWSQGGLVAQWG
LTFFPSIRSKVDRLMAFAPDYKGTVLAGPLDALAVSAPSVWQQTTGSALTTALRNAGGLTQIVPTTNL
YSATDEIVQPQVSNSPLDSSYLFNGKNVQAQAVCGPLFVIDHAGSLTSQFSYVVGRSALRSTTGQARS
ADYGITDCNPLPANDLTPEQKVAAAALLAPAAAAIVAGPKQNCEPDLMPYARPFAVGKRTCSGIVTP

pelB signal sequence

DNA Sequence CE07 wt

ATGTCCTTGCAGGCCAGGCTGATAAAAGCAGTAACCAAACGAACCATCAAGCGGAGCGGGTTAAACCAGGACCAA
CTAGTTCGCCACCTTCGCAAAGTCTTTAACGAAACCCCCGTGCTCACTCTACTGCCGCGGGGCGTGAAGCTGTCG
CGCGTAGAGCATCCTGCGTTCACCGGTGATCGTATTAGCGTGCAGAGGCCTGAAATGGCCGTGCTGTACTTACAC
GGTGGGGCCTATATCGGAGGTATAACGAAAACCTATCATAATTTGGCCGGCCGTCTTGCAAAAAAATTAAACGCG
GAAGTGTTCTTACCTGTTTATCCATTTGCCCCCGAACACCCATATCCCGCAGCTGTGAATCGGGTGATGGAGGCA
TACGAGTATCTGCTGAGTCTAGGAAAGCAACCCCAGGACATCGTAATTGCGGGTGACTCTGCCGGAGGCGGGCTG
ACTTTAGCCACCCTGCTCCACATAAGGGATAAGGGCCTTGAACAACCTCGGTGTGCAGTAACCTTTAGCCCGGCT
AGTAACGCGTTCCCGGACGACAGTATTCTCGAAGCGTTAGACCCTAGCGATGCCATGTTAAGTGCAGATATCATC
AGAACTGCAATAGAAATTTACACTCCTAACCCTGAAGATCGCAGTCAGCCCTACGCATCGCCGTGCCTGGGCGAT
TACACCAACATTTGCCCTCTCCTGATAACGGCATCTACGGATGAACTGCTGTATGCCGATGGGAAACGAGTCAAA
CGGGTAGCAGAGAAAGCTGGCGTGAAGGTGACCTGGATTGAACGTCCAGGGGTATTCCACGTGTGGCCAGTTATG
GTGCCTTTTCTGCCGGAAGCAAATAAAGATCTCAAGAGAATTGTTGCGTTCATTAAAGAGGCA

Amino Acid Sequence CE07 wt

MSLQARLIKAVTKRTIKRSGLNQDQLVRHLRKVFNETPVLTLLPRGVKLSRVEHPAFTGDRISVQRPEMAVLYLH
GGAYIGGITKTYHNLAGRLAKKLNAEVFLPVYPFAPEHPYPAAVNRVMEAYEYLLSLGKQPQDIVIAGDSAGGGL
TLATLLHIRDKGLEQPRCAVTFSPASNAFPDDSILEALDPSDAMLSADIIRTAIEIYTPNPEDRSQPYASPCLGD
YTNICPLLITASTDELLYADGKRVKRVAEKAGVKVTWIERPGVFHVWPVMVPFLPEANKDLKRIVAFIKEA

DNA Sequence CE03 wt

ATGGCCTCAATCCCGGCACATCTTATGAAGCTGCTTCTGCGCGCTGGTGTGAAGCGTGACATACGGGACCCTGAT
AAATTAGTAAAACATCTACGGCGTGCCATGAATGCACCGCTTGCCCCGTCACCTCTGCCACGTGGAATTAGGTTA
CAGCGTGGCAAAGTGGCAGGTACGGCTGGTCACTGGCTGAGTCCCACAGATCCCCAAACAACGATCTTATATCTG
CACGGCGGTGCTTTTATTGGTGGCCGGCTTGCTACATATCATAACTTTTGCGGGCATCTGGCGAGGACCTTAAAT
GCACGTGTATTTTTACCAGACTACCGGTTGGCACCCGAGCACCCGTTCCCGGCTGCAACGGATGATGCCTTTAAT
GTGTACCGGGAACTGATGGCTGATCCTCGACCGATCGTCATTGCAGGCGATAGCGCCGGCGGTAATCTGACACTT
GTGACACTGCTCCGCGCGCGGGATCACAAATTACGGATGCCCGCGTGTGCCGTTGCGATTTCGCCTGCGAGCGAC

GCCAGAGGCAATCTTATGTCGCGTCAGGCGAACTCCGACTCCGATGCGATGCTGTCGCACTGTATGATCGAAGTT
GCTACTGATGTGTATCTCGCAGGCGCAGATCCTGCTCACCCGTATGCATCCCCGATAACACAGGATTTCACTGGT
CTGCCGCCATTGCTATTTACTGTCTCTAGTGAGGAATGCCTGCGAGACGATGCTTACGCAGCCGCGCACTGCGCG
CGCCAGGCTGGCGTTCCTGTGCAGCTGCTTGAACGCAAAGATATGCCTCACGTTTGGCCAGTGTTCACTTTCTTG
TTACCTGAAGCAAAACAGGATCTTCCGACGATTGTGCGGTTCCTGCGTAAATATCTGGCGACCACAGATGCCCAT
GAAGAAGCATTCCACACGGCTAATGAGTCTGACACCACTATCCCAGAAATTTCTAGC

Amino Acid Sequence CE03 wt

MASIPAHLMKLLLRAGVKRDIRDPDKLVKHLRRAMNAPLAPSPLPRGIRLQRGKVAGTAGHWLSPTDPQTTILYL
HGGAFIGGRLATYHNFCGHLARTLNARVFLPDYRLAPEHPFPAATDDAFNVYRELMADPRPIVIAGDSAGGNLTL
VTLLRARDHKLRMPACAVAISPASDARGNLMSRQANSDSDAMLSHCMIEVATDVYLAGADPAHPYASPITQDFTG
LPPLLFTVSSEECLRDDAYAAAHCARQAGVPVQLLERKDMPHVWPVFTFLLPEAKQDLPTIVRFLRKYLATTDAH
EEAFHTANESDTTIPEISS

DNA Sequence CE13 wt

ATGCCGCAATCTTTTAAACAGTATAGAACAACCATAATGTCAACAGCCCGTCTCACGCCTACTACAGCGTCCACC
CTGAAACTGCTTTCAGTTGCCGTCGCCGCACTGACTCTGTCAGCATGTCTGAGCGGCGGAGGTGGGGGCCGGGCC
GATGGACCGAATCCGCTGGCAATCGAAACATCAGAAGGCAAAGTTGTTGGCATATCCAACGATGGTATTCGCGTC
TTTCGCGGTATCCCCTATGCGGCTCCCCCGGTTGGAGATTTGCGGCTTGCGCCTCCACAACCTCCAGCCTCAAGA
TCAGAAACCCTGCGGCTTAGCGAAGAATTTGGGAATTCCTGTCCCCAGTCCGACCTTACCACTGGCCAGCAAGTC
GGTAATGAAGATTGTTTGTATCTTAATGTTTATGCCCCGGCAGAAGCCGAAGACCTGCCCGTAATGGTGTGGATC
CACGGCGGTGCGTTCGTCTTCGGCAACGGTGGCGGTGAATACGATCCGACGCGTCTGGTGGAACAGGATGTTATC
GTTGTCACCCTTAACTATCGATTAGGAAACCTTGGCTTTCTGGCTCATCCCGCCCTGGAATCCGATGCCGGTAAT
TTCGCGCTGATGGACCAACAACTCGCTTTGGCATGGGTAAAAGAGAATATCGCAGCATTTGGCGGCGATCCTGCA
AATGTAACTATTTTCGGGGAGAGTGCGGGTGGACATTCAGTTATGAGCCATATTGTTAGCCCTCGGGCAGAAGAA
GCCGACTTATTCCAGCGCGCCATTGTTCAGTCCGGCAGCTATGCGCCATTCCAGATGCCGAAAGCAACTGCGCAG
TTTTTAGGGACTTCGGTCGCCAATGGTCTGGGGTGTACAGACCCTGAGACGGCTGCATCTTGCCTGCGAAGCCTT
CCAGTTTCCGCTTTTCTAGCAGCCCAGGGCAGCCAGTCAATTCCGGTGGTTGATCCTGACGATGATCTGCTACCG
AAAAGTATCCAGCAGGCTTTGGCGGATGGTGATTTTAATTCCAGCCTGGATATCATGATCGGTAGCAACCAAAAC
GAAGGGACGCTGTTTGTCGCGCTAGACGAAGTAGGCGGTGATCCGATTGACGATGAGGCTGAATACCGGGAACGA
GTGGCTGAATTTTTTCAGCCATACCAAGCGTCAATTCCTTTCGATGATGATCAAATAGCTACTGATTACCTTGAT
TTTGTGGACGGTGCGGCAAAACCATTCGAGGCCGCGTTGAGTGGTATCTGGACCGATTTCATGTTTGCATGTAAT
GCATATTCCCAAGCATCCACATTCGCGGGAGCCAGTATGAACACGTTTCAATATTGGTTTCGTGACGAGGACGCG
CCGTGGACTTTAGTCCCTCCCTTTGCTGTTTCCTTTCCACTAGGGGCAACGCATGCCGGTGAGATTCCCTATGTA
CTTTATCCACAGGCTATTATGGAACAACGTTATACGGGCGATCCGGACGATTTAAATTCATTGGCCGGCGAGATG
GTTGACTATTGGACACAGTTTGCCAAAACTGGTGATCCTAATACTACCGACGGCGTTGCGGCCGCATGGCAACAG
GCCGCTACCGGTAATTTGTTGACGCTGGATGTTCCCAATGCGAGTAATGCGAATACGCTAGGTTTTTTGGGATAC
CACCATTGTTCATATTGGGCGGACCCGCCGCTGGTTTTACCA

Amino Acid Sequence CE13 wt

MPQSFKQYRTTIMSTARLTPTTASTLKLLSVAVAALTLSACLSGGGGGRADGPNPLAIETSEGKVVGISNDGIRV
FRGIPYAAPPVGDLRLAPPQPPASRSETLRLSEEFGNSCPQSDLTTGQQVGNEDCLYLNVYAPAEAEDLPVMVWI
HGGAFVFGNGGGEYDPTRLVEQDVIVVTLNYRLGNLGFLAHPALESDAGNFALMDQQLALAWVKENIAAFGGDPA
NVTIFGESAGGHSVMSHIVSPRAEEADLFQRAIVQSGSYAPFQMPKATAQFLGTSVANGLGCTDPETAASCLRSL
PVSAFLAAQGSQSIPVVDPDDDLLPKSIQQALADGDFNSSLDIMIGSNQNEGTLFVALDEVGGDPIDDEAEYRER
VAEFFQPYQASIPFDDDQIATDYLDFVDGAAKPFEAALSGIWTDFMFACNAYSQASTFAGASMNTFQYWFRDEDA
PWTLVPPFAVSFPLGATHAGEIPYVLYPQAIMEQRYTGDPDDLNSLAGEMVDYWTQFAKTGDPNTTDGVAAAWQQ
AATGNLLTLDVPNASNANTLGFLGYHHCSYWADPPLVLP

DNA Sequence MsAcT wt

ATGGCAAAACGTATTCTGTGTTTTGGTGATAGCCTGACCTGGGGTTGGGTTCCTGTTGAAGATGGTGCACCGACC
GAACGTTTTGCACCGGATGTTCGTTGGACCGGTGTTCTGGCACAGCAGCTGGGTGCAGATTTTGAAGTTATTGAA
GAAGGTCTGAGCGCACGTACCACCAATATTGATGATCCGACCGATCCGCGTCTGAATGGTGCAAGCTATCTGCCG
AGCTGTCTGGCAACCCATCTGCCGCTGGATCTGGTGATTATTATGCTGGGCACCAATGATACCAAAGCCTATTTT
CGTCGTACACCGCTGGATATTGCACTGGGTATGAGCGTTCTGGTTACCCAGGTTCTGACCAGTGCCGGTGGTGTT
GGCACCACCTATCCGGCACCGAAAGTTCTGGTTGTTAGTCCGCCTCCGCTGGCACCGATGCCGCATCCGTGGTTT

CAGCTGATTTTTGAAGGTGGTGAACAGAAAACCACCGAACTGGCACGTGTTTATAGCGCACTGGCAAGCTTTATG
AAAGTGCCGTTTTTTGATGCAGGTAGCGTTATTAGCACCGATGGTGTTGATGGTATTCACTTTACCGAAGCCAAT
AATCGTGATCTGGGTGTTGCACTGGCCGAACAGGTTCGTAGCCTGCTG

Amino Acid Sequence MsAcT wt

MAKRILCFGDSLTWGWVPVEDGAPTERFAPDVRWTGVLAQQLGADFEVIEEGLSARTTNIDDPTDPRLNGASYLP
SCLATHLPLDLVIIMLGTNDTKAYFRRTPLDIALGMSVLVTQVLTSAGGVGTTYPAPKVLVVSPPPLAPMPHPWF
QLIFEGGEQKTTELARVYSALASFMKVPFFDAGSVISTDGVDGIHFTEANNRDLGVALAEQVRSLL

DNA Sequence EstCE1 wt

ATGTCGATAGCGGATCAGTCATTAGCAAAAAGAGTGCAGGGCGTTAGCCAACAGGCGATTGATGAAGGGCGTATC
GTTGGCAGCGTGGTGCTGATCGCTCGGCACGGTCGCGTGATTTACGCCAATGCCAGCGGCTATGCCGATCGTGAA
CAGAAGAAACCTATGGTGCGTGAGACCCAATTTCGGCTGTCGTCGGTGTCCAAGCCTTATATTACGCTGGCGGCC
ATGCGTATGATCGAACAGCAGAAGCTGGGGCTGGATGATACCGTCAGCCGTTGGTTGCCGTGGTTTACCCCGGCG
CTGGCCGATGGGGTTCGCCCGCCAATTAAAATCCGTCACTTGTTGAGCCACACTGCCGGCCTGGATTATCGTTTG
AGCCAACCTGCGGAAGGACCGTATCATCGACTCGGTATTAAAGACGGTATGGAACTGTCGTCGTTAACGCTGGAA
CAGAATCTGCGCCTGTTGGCGCAGGCGGATCTGTTGGCCGAGCCGGGCAGCGAGTTTCGATATTCACTGGCAATC
GATGTGCTGGGGGCGGTGCTGGAACAGGTGGCGGGCGAGCCCTTGCCGCAGGTGTTCAACCATTGGGTTGCCCAA
CCTTTGGGGTTGCGTAATACCGGTTTTTACACCACCGATGTCGATAATCTGGCAACGGCGTATCACGACACCGCC
GCGGAGCCGGAACCTATACGAGATGGCATGTTGCTGACCCTGCCGGAAGGGTTCGGCTTCGAGATTGAACTGGCA
CCCTCGCGCGCACTGGACGCTCAGGCCTATCCTTCTGGCGGCGCTGGCATGGTCGGCGATGCAGACGATGTGTTG
CAGTTGGTGGAAACCTTGCGCACTGGCAAGGAAGGCATTTTACAGCCGGCCACCGCAGCGCTGATGCGTCAAGCG
CATGTCGGGTCGCACGCCGAGACTCAGGGGCCCGGCTGGGGGTTTGGTTTCGGCGGTGCGGTACTGGAAGATGCG
CAGTTGGCGGCGACGCCTCAGCACAATGGCACTCTGCAGTGGGGCGGTGTCTATGGCCACAGTTGGTTTTACGAT
CCGCAAGCGGCGATCAGCGTGGTAGCCTTGACCAATACGGCCTTTGAAGGCATGAGTGGACGTTATCCACTGCAA
ATCCGCGATGCTGTTTACGGGACAAACGAACCTACTCGC

Amino Acid Sequence EstCE1 wt

MSIADQSLAKRVQGVSQQAIDEGRIVGSVVLIARHGRVIYANASGYADREQKKPMVRETQFRLSSVSKPYITLAA
MRMIEQQKLGLDDTVSRWLPWFTPALADGVRPPIKIRHLLSHTAGLDYRLSQPAEGPYHRLGIKDGMELSSLTLE
QNLRLLAQADLLAEPGSEFRYSLAIDVLGAVLEQVAGEPLPQVFNHWVAQPLGLRNTGFYTTDVDNLATAYHDTA
AEPEPIRDGMLLTLPEGFGFEIELAPSRALDAQAYPSGGAGMVGDADDVLQLVETLRTGKEGILQPATAALMRQA
HVGSHAETQGPGWGFGFGGAVLEDAQLAATPQHNGTLQWGGVYGHSWFYDPQAAISVVALTNTAFEGMSGRYPLQ
IRDAVYGTNEPTR

DNA Sequence RML wt

GTGCCAATCAAGAGACAATCAAACAGCACGGTGGATAGTCTGCCACCCCTCATCCCCTCTCGAACCTCGGCACCT
TCATCATCACCAAGCACAACCGACCCTGAAGCTCCAGCCATGAGTCGCAATGGACCGCTGCCCTCGGATGTAGAG
ACTAAATATGGCATGGCTTTGAATGCTACTTCCTATCCGGATTCTGTGGTCCAAGCAATGAGCATTGATGGTGGT
ATCCGCGCTGCGACCTCGCAAGAAATCAATGAATTGACTTATTACACTACACTATCTGCCAACTCGTACTGCCGC
ACTGTCATTCCTGGAGCTACCTGGGACTGTATCCACTGTGATGCAACGGAGGATCTCAAGATTATCAAGACTTGG
AGCACGCTCATCTATGATACAAATGCAATGGTTGCACGTGGTGACAGCGAAAAAACTATCTATATCGTTTTCCGA
GGTTCGAGCTCTATCCGCAACTGGATTGCTGATCTCACCTTTGTGCCAGTTTCATATCCTCCGGTCAGTGGTACA
AAAGTACACAAGGGATTCCTGGACAGTTACGGGGAAGTTCAAAACGAGCTTGTTGCTACTGTTCTTGATCAATTC
AAGCAATATCCAAGCTACAAGGTTGCTGTTACAGGTCACTCACTCGGTGGTGCTACTGCGTTGCTTTGCGCCCTG
GGTCTCTATCAACGAGAAGAAGGACTCTCATCCAGCAACTTGTTCCTTTACACTCAAGGTCAACCACGGGTAGGC
GACCCTGCCTTTGCCAACTACGTTGTTAGCACCGGCATTCCTTACAGGCGCACGGTCAATGAACGAGATATCGTT
CCTCATCTTCCACCTGCTGCTTTTGGTTTTCTCCACGCTGGCGAGGAGTATTGGATTACTGACAATAGCCCAGAG
ACTGTTCAGGTCTGCACAAGCGATCTGGAAACCTCTGATTGCTCTAACAGCATTGTTCCCTTCACAAGTGTTCTT
GACCATCTCTCGTACTTTGGTATCAACACAGGCCTCTGTACT

Amino Acid Sequence RML wt

VPIKRQSNSTVDSLPPLIPSRTSAPSSSPSTTDPEAPAMSRNGPLPSDVETKYGMALNATSYPDSVVQAMSIDGG
IRAATSQEINELTYYTTLSANSYCRTVIPGATWDCIHCDATEDLKIIKTWSTLIYDTNAMVARGDSEKTIYIVFR
GSSSIRNWIADLTFVPVSYPPVSGTKVHKGFLDSYGEVQNELVATVLDQFKQYPSYKVAVTGHSLGGATALLCAL

GLYQREEGLSSSNLFLYTQGQPRVGDPAFANYVVSTGIPYRRTVNERDIVPHLPPAAFGFLHAGEEYWITDNSPE
TVQVCTSDLETSDCSNSIVPFTSVLDHLSYFGINTGLCT

Propeptide

DNA Sequence MAE2 wt

ATGATTAGCCTGGCCGATCTGCAGCGTCGTATTGAAACCGGTGAACTGAGCCCGAATGCAGCAATTGCACAGAGC
CATGCAGCCATTGAAGCACGTGAAAAAGAAGTTCACGCATTTGTTCGTCATGATAAAAGCGCACGTGCACAGGCA
AGCGGTCCGCTGCGTGGTATTGCAGTTGGTATTAAAGATATTATCGACACCGCAAATATGCCGACCGAAATGGGT
AGCGAAATTTATCGTGGTTGGCAGCCTCGTTCAGATGCACCGGTTGTTATGATGCTGAAACGTGCCGGTGCAACC
ATTATTGGTAAAACCACCACCACCGCATTTGCAAGCCGTGATCCGACCGCAACCCTGAATCCGCATAATACCGGT
CATAGTCCGGGTGGTAGCAGCAGCGGTAGCGCAGCAGCAGTTGGTGCAGGTATGATTCCGCTGGCACTGGGCACC
CAGACCGGTGGTAGCGTTATTCGTCCGGCAGCATATTGTGGCACCGCAGCAATTAAACCGAGCTTTCGCATGCTG
CCGACCGTTGGTGTTAAATGTTATAGCTGGGCACTGGATACCGTTGGTCTGTTTGGTGCCCGTGCCGAAGATCTG
GCACGTGGTCTGCTGGCAATGACCGGTCGTAGTGAATTTAGCGGTATTGTTCCGGCAAAAGCACCGCGTATTGGT
GTTGTGCGTCAAGAATTTGCGGGTGCAGTTGAACCGGCAGCAGAACAGGGTCTGCAGGCAGCGATTAAAGCAGCC
GAACGTGCGGGTGCCAGCGTTCAGGCAATTGATCTGCCGGAAGCAGTTCATGAAGCATGGCGTATTCATCCGATT
ATCCAGGATTTTGAAGCACATCGTGCACTGGCATGGGAATTTAGTGAACATCACGATGAAATTGCACCGATGCTG
CGTGCAAGCCTGGATGCAACCGTGGGTCTGACCCCGAAAGAATATGATGAAGCCCGTCGCATTGGTCGTCGTGGT
CGCCGTGAACTGGGTGAAGTTTTTGAAGGTGTTGATGTGCTGCTGACCTATAGCGCACCGGGTACAGCCCCTGCA
AAAGCCCTGGCAAGCACCGGTGATCCGCGTTATAATCGTCTGTGGACCCTGATGGGTAATCCGTGTGTTAATGTT
CCGGTTCTGAAAGTTGGTGGTCTGCCGATTGGTGTTCAGGTTATTGCACGTTTTGGTAATGATGCACATGCACTG
GCCACCGCATGGTTTCTGGAAGATGCGCTGGCAAAAAGCGGT

Amino Acid Sequence MAE2 wt

MISLADLQRRIETGELSPNAAIAQSHAAIEAREKEVHAFVRHDKSARAQASGPLRGIAVGIKDIIDTANMPTEMG
SEIYRGWQPRSDAPVVMMLKRAGATIIGKTTTTAFASRDPTATLNPHNTGHSPGGSSSGSAAAVGAGMIPLALGT
QTGGSVIRPAAYCGTAAIKPSFRMLPTVGVKCYSWALDTVGLFGARAEDLARGLLAMTGRSEFSGIVPAKAPRIG
VVRQEFAGAVEPAAEQGLQAAIKAAERAGASVQAIDLPEAVHEAWRIHPIIQDFEAHRALAWEFSEHHDEIAPML
RASLDATVGLTPKEYDEARRIGRRGRRELGEVFEGVDVLLTYSAPGTAPAKALASTGDPRYNRLWTLMGNPCVNV
PVLKVGGLPIGVQVIARFGNDAHALATAWFLEDALAKSG

DNA Sequence CIH wt

GCCAAAGAAATTCTGTGCAGCTTTGGTATTGATGTTGATGCAGTTGCAGGTTGGCTGGGTAGCTATGGTGGTGAA
GATAGTCCGGATGATATTTCACGTGGTCTGTTTGCCGGTGAAGTTGGTAGTCCGCGTCTGCTGAAACTGTTTGAA
CGTTTTGGTATTAAAACCACCTGGTTTATTCCGGGTCATAGCATTGAAACCTTTCCGGAACAAATGCAGGCAGTT
GCCGATGCAGGTCATGAAATTGGTATTCATGGTTATACGCATGAAAACCCGATTGCAATGACCCGTGAACAAGAA
ACCGCAGTTCTGGATAAATGTATTGATCTGGTTACCAAACTGAGCGGTAAACGTCCGACCGGTTATGTTGCACCG
TGGTGGGAATTTAGCAATGTTACCAATGAACTGCTGCTGGAACGTGGCATCAAATATGATCATTCACTGATGCAC
AATGACTTCACCCCGTATTATGTTCGTGTTGGTGATAAATGGACCAAAATCGATTACAGCAAAAAACCGAGCGAT
TGGATGGTTCCGCTGACCCGTGGTAAAGAAACGGATCTGATTGAAATTCCGGCAAGCTGGTATCTGGATGATCTG
CCTCCGATGATGTTTATCAAAAAAAGCCCGAATAGCCACGGTTTTGTTAACCCGCATGATATTGAGCAGATTTGG
CGTGATCAGTTTGATTGGGTTTATCGCGAAATGGATTATGCCGTTTTTCCGATTACCATTCATCCTGATGTTGCA
GGTCGTCCGCAGGTTCTGATGATGCTGGAACGCCTGTATGCACATATGATTAAACATCCGGGTGTGAAATTTGTG
ACCATGAATGAAATTGCCGATGATTTCGCCAAACGTTTCCCTCGTAAAAAA

Amino Acid Sequence CIH wt

AKEILCSFGIDVDAVAGWLGSYGGEDSPDDISRGLFAGEVGSPRLLKLFERFGIKTTWFIPGHSIETFPEQMQAV
ADAGHEIGIHGYTHENPIAMTREQETAVLDKCIDLVTKLSGKRPTGYVAPWWEFSNVTNELLLERGIKYDHSLMH
NDFTPYYVRVGDKWTKIDYSKKPSDWMVPLTRGKETDLIEIPASWYLDDLPPMMFIKKSPNSHGFVNPHDIEQIW
RDQFDWVYREMDYAVFPITIHPDVAGRPQVLMMLERLYAHMIKHPGVKFVTMNEIADDFAKRFPRKK

DNA Sequence AMI wt

GCAACCATTCGTCCGGATGATAATGCAATTGATACCGCAGCAAAACATTATGGCATTACCCTGGATCAGAGCGCA
CGTCTGGAATGGCCTGCACTGATTGATGGTGCACTGGGTAGCTATGATGTTGTTGATCAGCTGTATGCAGATGAA
GCAACCCCTCCGACCACCAGTCGTGAACATACCGTTCCGACCGCAAGCGAAAATCCGCTGAGCGCATGGTATGTT

ACCACCAGTATTCCGCCTACCAGTGATGGTGTTCTGACCGGTCGTCGTGTTGCAATTAAAGATAATGTTACCGTT
GCCGGTGTTCCGATGATGAATGGTAGCCGTACCGTTGAAGGTTTTACCCCGAGCCGTGATGCAACCGTTGTTACC
CGTCTGCTGGCAGCCGGTGCCACCGTTGCAGGTAAAGCAGTTTGTGAAGATCTGTGTTTTAGCGGTAGCAGCTTT
ACACCGGCATCAGGTCCGGTTCGTAATCCGTGGGATCCGCAGCGTGAAGCCGGTGGTAGCAGCGGTGGTAGTGCA
GCACTGGTTGCAAATGGTGATGTTGATTTTGCAATTGGTGGTGATCAGGGTGGTTCAATTCGTATTCCGGCAGCA
TTTTGTGGTGTTGTTGGTCATAAACCGACCTTTGGTCTGGTTCCGTATACCGGTGCATTTCCGATTGAACGTACA
ATTGATCATCTGGGTCCGATTACCCGTACCGTGCATGATGCAGCCCTGATGCTGAGCGTTATTGCAGGTCGTGAT
GGTAATGATCCGCGTCAGGCAGATAGCGTTGAAGCGGGTGATTATCTGAGCACCCTGGATAGTGATGTGGATGGT
CTGCGTATTGGTATTGTTCGCGAAGGTTTTGGTCATGCAGTTAGCCAGCCGGAAGTTGATGATGCAGTTCGTGCA
GCAGCACATAGCCTGGCAGAAATTGGTTGTACCGTGGAAGAAGTTAATATTCCTTGGCATCTGCATGCCTTTCAT
ATTTGGAATGTTATTGCAACCGATGGTGGTGCATATCAGATGCTGGATGGCAATGGTTATGGTATGAATGCCGAA
GGTCTGTATGATCCGGAACTGATGGCACATTTTGCAAGCCGTCGTCTGCAGCATGCCGATGCACTGAGCGAAACC
GTTAAACTGGTTGCCCTGACAGGTCATCATGGTATTACCACCCTGGGTGGCGCAAGCTATGGTAAAGCACGTAAT
CTGGTGCCGCTGGCACGTGCAGCATATGATACAGCACTGCGTCAGTTTGATGTTCTGGTTATGCCGACCCTGCCG
TATGTTGCAAGCGAACTGCCTGCAAACGATGTTGATCGTGCAACCTTTATTACCAAAGCCCTGGGTATGATTGCA
AATACCGCACCGTTTGATGTGACGGGTCATCCGAGCCTGAGCGTTCCGGCAGGTCTGGTTAATGGTCTGCCGGTT
GGTATGATGATTACCGGCAAAACCTTTGATGACGCAACCGTTCTGCGTGTTGGTCGTGCATTTGAAAAACTGCGT
GGTGCCTTTCCGACACCGGCAGATCATATTAGCGATAGCGCACCGCAGCTGAGCCTGACC

Amino Acid Sequence AMI wt

ATIRPDDNAIDTAAKHYGITLDQSARLEWPALIDGALGSYDVVDQLYADEATPPTTSREHTVPTASENPLSAWYV
TTSIPPTSDGVLTGRRVAIKDNVTVAGVPMMNGSRTVEGFTPSRDATVVTRLLAAGATVAGKAVCEDLCFSGSSF
TPASGPVRNPWDPQREAGGSSGGSAALVANGDVDFAIGGDQGGSIRIPAAFCGVVGHKPTFGLVPYTGAFPIERT
IDHLGPITRTVHDAALMLSVIAGRDGNDPRQADSVEAGDYLSTLDSDVDGLRIGIVREGFGHAVSQPEVDDAVRA
AAHSLAEIGCTVEEVNIPWHLHAFHIWNVIATDGGAYQMLDGNGYGMNAEGLYDPELMAHFASRRLQHADALSET
VKLVALTGHHGITTLGGASYGKARNLVPLARAAYDTALRQFDVLVMPTLPYVASELPANDVDRATFITKALGMIA
NTAPFDVTGHPSLSVPAGLVNGLPVGMMITGKTFDDATVLRVGRAFEKLRGAFPTPADHISDSAPQLSLT

DNA Sequence 2R11 wt

ATGTCAAACCATTCATCTAGTATTCCCGAATTAAGTGACAACGGTATCCGCTATTATCAAACTTATAATGAAAGC
CTTAGTCTTTGGCCGGTCCGTTGTAAATCATTCTATATATCTACTCGTTTTGGTCAAACACATGTGATTGCAAGC
GGCCCAGAGGATGCCCCGCCGCTTGTATTACTCCACGGAGCATTATTCAGCTCGACGATGTGGTATCCCAACATC
GCCGATTGGAGCAGTAAATACAGAACTTATGCAGTTGATATCATAGGTGATAAAAACAAGAGTATTCCTGAGAAT
GTAAGCGGTACAAGAACGGATTACGCCAATTGGCTTCTTGATGTGTTTGACAATCTGGGGATCGAAAAGTCCCAC
ATGATCGGACTTTCGCTTGGCGGTCTCCATACGATGAATTTCCTTTTACGTATGCCTGAGAGAGTAAAAAGCGCA
GCTATACTGAGTCCGGCAGAAACGTTTTTGCCATTTCATCACGATTTCTACAAATACGCTCTTGGCCTTACAGCG
TCAAATGGAGTTGAAACATTCTTAAATTGGATGATGAATGATCAGAATGTGCTGCACCCGATTTTTGTGAAGCAG
TTTAAGGCAGGGGTAATGTGGCAGGATGGATCAAGAAATCCAAATCCTAATGCCGACGGATTTCCGTATGTTTTT
ACCGATGAGGAATTACGTTCAGCAAGAGTTCCTATCCTATTATTACTTGGTGAACATGAAGTCATCTATGATCCC
CACTCAGCCCTGCACCGAGCCTCTTCATTCGTTCCAGATATTGAGGCGGAAGTCATTAAAAATGCCGGACATGTT
TTATCGATGGAACAACCCACTTACGTAAATGAACGTGTAATGCGTTTTTTCAATGCAAAAACAGGCATTTCACGG
TAA

Amino Acid Sequence 2R11 wt

MSNHSSSIPELSDNGIRYYQTYNESLSLWPVRCKSFYISTRFGQTHVIASGPEDAPPLVLLHGALFSSTMWYPNI
ADWSSKYRTYAVDIIGDKNKSIPENVSGTRTDYANWLLDVFDNLGIEKSHMIGLSLGGLHTMNFLLRMPERVKSA
AILSPAETFLPFHHDFYKYALGLTASNGVETFLNWMMNDQNVLHPIFVKQFKAGVMWQDGSRNPNPNADGFPYVF
TDEELRSARVPILLLLGEHEVIYDPHSALHRASSFVPDIEAEVIKNAGHVLSMEQPTYVNERVMRFFNAKTGISR

DNA Sequence PpATase-F148V

AAGGAGATATACATATGATGAATGTGAAGAAAATAGGTATCGTTAGCTACGGCGCCGGTATCCCGGTATGTCGCC
TGAAAGTTCAGGAAGTGATTAATGTTTGGAAGAATACCGATCTCAAACTGGTGGAGGAAAATCTCGGCGTTACGG
AAAGGGCGGTGCTGCAACCGGACGAAGACGTTATTACCCTCGGGGTGCTGGCCGCCCAACGGGCGCTGGACAAGG
TTCCAGGTCACCAGATTGAAGCGCTGTATCTGGGCACCTGCACCAACCCATACGATTCCCGGGCGTCGGCTTCGA
TTATCCTGGAAATGCTCGGCAGCGGCTATGATGCGTACTGCGCGGATGTGCAGTTTGCGGGCAAATCGGGAACTT
CAGCGCTGCAGATTTGCCAGGCCCTGGTGGCTTCGGGCATGACGGGCAGCGCGCTGGCGATTGGCGCGGATACCA

TTAATCGCAATACCGCGCCGGGCGATCTGACCGAATCTTACGCGGGGGCGGGAGCGGCTGCCCTGCTGATTGGCA
GCCAGGACGTTATTGCGGAATTTGATGCGAGCTTTTCTTGCGCGGCCGACGTTGCGGATAATATTCGCCCACAGG
GCGACCGCTATATCCGTTCGGGCATGGGCCTGGGCTCGGATAAAAATAGCATTGGCCTGGAAGATCAGACCCGCC
GTGCGGCCGAAGGCCTGATGGCGAAACTGCATACCAGCCCAGCGGATTACGATTACGTGGTTTTTCAGCAAAATC
TGGTGTCGACGCCATATTCTCTGGCGAAACATCTGGGCTTTAATCCAAAACAGGTGGAACCGGGCATTTACGCGG
GCAATGTAGGCGACGCGGGATCGGCGAGCCCGCTGCTCGGCCTGATTAATGTACTGGATCAGGCACGCCCGGGGC
AGAAGATCCTTTTGGTGTCTTATGGTTTTGGCGCGGGCAGCGATGCGATTGCGCTGACCGTTACCGATGCGATTG
AGCAGTATCAGAAACATAATAAACCTCTGCGCGAACTGCTGGAATCTAAAATTTATGTTGATTATGGCACGTCTA
TTAAATATGAGTTTAAATATCTGCGGGCTGATTATGCGCTGACCGCGTACCTCTAAGGTACCAAGGAGATATACA
TATGATGTGCGCACGTCGCGTAGCAATTGTATCGGCGGCCTACACGCCGAAGCCAGGAAGTTCACGAGTACGGCA
GACGTTTAAAGAAATGATTGTTGAGTCTGCGTATAAAGCACTCAAAGATGCGAAAATGCATCCACGGGAAATTCA
GGCGGTGGCGTACGGTTACCATGGTGAAGGCATCTCGGAATACGGCGGTCTGGGCCCGACCATCTCTGATGCGCT
GGGCATTAGCCCGGCCCCGACCTTTATGAGCACCGCGAATTGCACCAGCAGCTCGGTGTCGTTTCAGATGGGCCA
TCAGATGGTGGCCTCGGGGGAGTATGATATTGTTCTGTGCGGCGGTTTTGAGAAAATGACCGACCATTTTAATTA
TGCGGAATATATTGGCTCGAGCACTGAATGTGAATATGACTACTTTCTCGGCATCTCTCATACCGACGCGGTTGC
GCTGGCGACCGCGGAGTATTTTCAGAAATTTGGCTACGCGGGTCGCGAGGCGGATGTACTGGCGACCTTTGGCCG
GCAGATGCGCATTTATGCACAGAATACCCCAACCGCGACCCGTTACGGCCAGCCGATCCCATCGCTGGAAGTGTT
GAAAAATAGCGAAGCGTGCGGCTCGATGCTGGCGTGGGGCGAAGCGAGTGGCTGCGCCATTCTGGTGGCGGAACA
TCTGGCGCATAAATATACCGACAAGCCGGTGTTTGTACGCGGTTGCGCGTACACCGGGGTTTCTCATTACTTTGG
TACGCGCTTCCATAATCCGACCCTGCACCATCCGGGCCTGCCAAAAGACGTGGGCATGGCCGTCTCGGCGAATTC
TATTGCGTGTGCCGAGATTGCGTATAAAAAGGCGGGGATTACCGCGAAAGATATTGATGTGGCGCAGGTTTATGA
TCTGCTCGGCGCAGGGCTGATTCAGATGGAATCTATGGGCATTTGTGGCAAAGGCCAGGCGGGCGATTTTGTGCT
CGAAGGCGGTATCGCGCTGGACGGCCAGCTGCCGCTCAACACCGATGGCGGTAATATTGGCCGCGGCCACGCGTC
TGGCTGCGATGGCATTCTGCATATTACCGAGCTGTTTCGGCAGCTGAGAGGTGAATCGGATAATCAGGTTAAAGG
CGCGCGCATTGGCGTGTCGCAGAATCTTGGCGGTTATGCCGCGCATAATTCCGTGATCGTTCTCTCGAATGATTG
AATTTAAATCACATGCTCGGCTCAGTCGCCTGCGGATGCATGGTCTGGATGTGCCGAGGTCTGCGTCGGTGCAGA
CTCAGTCGACGAGATCTAAGGAGATATACATATGATGTCTATGTATCCTGAACAGATTCATAGAATGACCACCGC
GAGCATGCTTCGCGAATGGCGCGAGCACGGCGGTAAATATCGCCTCGAAGGCAGCCAGTGCGAAGAATGCAATGA
AATTTTTTTCCCACGGCGCACCGTTTGCGGCGCTTGCAATTCTCTGAGTGTGAAACCGTACCGCTGCGCCCGCAG
TGGCAAAATTGAGGTTATGGCGCCGGCGGAGAATCCGATTCTGGCGGCCATGGGCTACGGCGAAACCGTGCCGCG
CATTATGGCGATGGTGCGCCTGGATGATGGCCTGGTGATTGCTTCGGAAATTGTTGACGTGTGCGACCAGCAACA
GCTGAAGGTTGGTGCGCCGGTGCGCATGGTGATTCGCAAACACGTGCGCGAAAGCAATCTGGCGTGGCAATATGC
TTACAAATTTGTACTCGACATATGA

Amino Acid Sequence PpATase-F148V

PhlA

MNVKKIGIVSYGAGIPVCRLKVQEVINVWKNTDLKLVEENLGVTERAVLQPDEDVITLGVLAAQRALDKVPGHQI
EALYLGTCTNPYDSRASASIILEMLGSGYDAYCADVQFAGKSGTSALQICQALVASGMTGSALAIGADTINRNTA
PGDLTESYAGAGAAALLIGSQDVIAEFDASFSCAADVADNIRPQGDRYIRSGMGLGSDKNSIGLEDQTRRAAEGL
MAKLHTSPADYDYVVFQQNLVSTPYSLAKHLGFNPKQVEPGIYAGNVGDAGSASPLLGLINVLDQARPGQKILLV
SYGFGAGSDAIALTVTDAIEQYQKHNKPLRELLESKIYVDYGTSIKYEFKYLRADYALTAYL

PhlC

MCARRVAIVSAAYTPKPGSSRVRQTFKEMIVESAYKALKDAKMHPREIQAVAYGYHGEGISEYGGLGPTISDALG
ISPAPTFMSTANCTSSSVSFQMGHQMVASGEYDIVLCGGFEKMTDHFNYAEYIGSSTECEYDYFLGISHTDAVAL
ATAEYFQKFGYAGREADVLATFGRQMRIYAQNTPTATRYGQPIPSLEVLKNSEACGSMLAWGEASGCAILVAEHL
AHKYTDKPVFVRGCAYTGVSHYFGTRFHNPTLHHPGLPKDVGMAVSANSIACAEIAYKKAGITAKDIDVAQVYDL
LGAGLIQMESMGICGKGQAGDFVLEGGIALDGQLPLNTDGGNIGRGHASGCDGILHITELFRQLRGESDNQVKGA
RIGVSQNLGGYAAHNSVIVLSND

PhlB

MSMYPEQIHRMTTASMLREWREHGGKYRLEGSQCEECNEIFFPRRTVCGACNSLSVKPYRCARSGKIEVMAPAEN
PILAAMGYGETVPRIMAMVRLDDGLVIASEIVDVCDQQQLKVGAPVRMVIRKHVRESNLAWQYAYKFVLDI

DNA Sequence PestE wt

ATGCCTCTTAGCCCTATACTAAGGCAAATTCTCCAACAGTTGGCCGCGCAGTTGCAGTTTAGACCCGACATGGAC
GTCAAGACGGTGAGAGAGCAGTTTGAGAAGTCCTCCCTCATCCTCGTCAAAATGGCCAATGAGCCTATTCACCGT
GTGGAGGACATCACGATTCCGGGCAGGGGCGGGCCAATTAGGGCTAGGGTTTATAGGCCGCGGGATGGGGAGAGG
TTGCCCGCGGTGGTGTACTACCACGGCGGGGGCTTCGTCTTGGGGAGCGTGGAGACTCACGACCACGTGTGTAGG
CGGTTGGCCAACCTCTCCGGGGCAGTCGTCGTCTCTGTGGACTACCGCCTAGCCCCCGAGCACAAATTCCCCGCC
GCCGTGGAAGACGCATACGACGCCGCCAAGTGGGTCGCCGACAACTACGACAAGCTCGGCGTCGACAATGGGAAA
ATCGCCGTGGCTGGGGACTCGGCGGGGGGCAACTTAGCCGCGGTGACGGCCATCATGGCCAGGGACAGGGGGGAG
AGCTTTGTGAAATACCAAGTGTTAATCTACCCCGCGGTCAACCTCACTGGGTCTCCCACAGTGTCTAGAGTGGAG
TACAGCGGGCCCGAATACGTCATCCTCACCGCCGACTTAATGGCGTGGTTTGGGAGACAGTATTTCTCAAAGCCG
CAAGACGCCCTCAGTCCCTATGCCTCTCCCATATTTGCAGATTTGTCAAACCTCCCGCCCGCCCTGGTGATAACC
GCCGAGTACGACCCGCTACGCGACGAGGGAGAGCTCTACGCCCACTTGTTGAAGACTAGGGGAGTTAGGGCCGTG
GCGGTGAGGTACAACGGCGTCATCCACGGCTTCGTCAACTTCTACCCCATATTAGAAGAGGGGAGAGAGGCAGTT
TCGCAAATTGCGGCCTCAATAAAGTCGATGGCTGTGGCGTAA

Amino Acid Sequence PestE wt

MPLSPILRQILQQLAAQLQFRPDMDVKTVREQFEKSSLILVKMANEPIHRVEDITIPGRGGPIRARVYRPRDGER
LPAVVYYHGGGFVLGSVETHDHVCRRLANLSGAVVVSVDYRLAPEHKFPAAVEDAYDAAKWVADNYDKLGVDNGK
IAVAGDSAGGNLAAVTAIMARDRGESFVKYQVLIYPAVNLTGSPTVSRVEYSGPEYVILTADLMAWFGRQYFSKP
QDALSPYASPIFADLSNLPPALVITAEYDPLRDEGELYAHLLKTRGVRAVAVRYNGVIHGFVNFYPILEEGREAV
SQIAASIKSMAVA

DNA Sequence Est2 wt

ATGCCGCTGGATCCGGTTATTCAGCAGGTGCTGGATCAGCTGAATCGTATGCCGGCCCCGGATTATAAACATCTG
AGTGCACAGCAGTTTCGTAGTCAGCAGAGCCTGTTTCCGCCGGTGAAAAAAGAACCGGTGGCAGAAGTTCGTGAA
TTTGATATGGATCTGCCGGGTCGTACCCTGAAAGTTCGTATGTATCGTCCGGAAGGTGTGGAACCGCCGTATCCG
GCCCTGGTTTATTATCATGGCGGTGGTTGGGTTGTGGGCGATCTGGAAACCCATGATCCGGTGTGCCGCGTTCTG
GCCAAAGATGGTCGCGCCGTGGTGTTTAGCGTTGATTATCGTCTGGCACCGGAACATAAATTTCCGGCCGCCGTT
GAAGATGCATATGATGCACTGCAGTGGATTGCAGAACGTGCCGCAGATTTTCATCTGGATCCGGCCCGCATTGCA
GTTGGTGGTGACAGCGCCGGTGGTAATCTGGCAGCAGTGACCAGTATTCTGGCAAAAGAACGCGGCGGCCCGGCA
CTGGCATTTCAGCTGCTGATCTATCCGAGCACCGGTTATGATCCGGCACATCCGCCGGCAAGTATTGAAGAAAAT
GCCGAAGGTTATCTGCTGACCGGTGGTATGATGCTGTGGTTTCGCGATCAGTATCTGAATAGCCTGGAAGAACTG
ACCCATCCGTGGTTTAGTCCGGTTCTGTATCCGGATCTGAGCGGCCTGCCGCCGGCCTATATTGCCACCGCACAG
TATGATCCGCTGCGCGATGTGGGTAAACTGTATGCAGAAGCACTGAATAAGGCAGGTGTGAAAGTGGAAATTGAA
AATTTTGAAGACCTGATTCACGGTTTTGCCCAGTTTTATAGCCTGAGCCCGGGCGCCACCAAAGCACTGGTTCGC
ATTGCCGAAAAACTGCGCGATGCACTGGCC

Amino Acid Sequence Est2 wt

MPLDPVIQQVLDQLNRMPAPDYKHLSAQQFRSQQSLFPPVKKEPVAEVREFDMDLPGRTLKVRMYRPEGVEPPYP
ALVYYHGGGWVVGDLETHDPVCRVLAKDGRAVVFSVDYRLAPEHKFPAAVEDAYDALQWIAERAADFHLDPARIA
VGGDSAGGNLAAVTSILAKERGGPALAFQLLIYPSTGYDPAHPPASIEENAEGYLLTGGMMLWFRDQYLNSLEEL
THPWFSPVLYPDLSGLPPAYIATAQYDPLRDVGKLYAEALNKAGVKVEIENFEDLIHGFAQFYSLSPGATKALVR
IAEKLRDALA

References

1. Zeng, S.; Liu, J.; Anankanbil, S.; Chen, M.; Guo, Z.; Adams, J. P.; Snajdrova, R.; Li, Z., Amide Synthesis via Aminolysis of Ester or Acid with an Intracellular Lipase. *ACS Catal.* **2018,** *8*, 8856-8865.
2. Wang, J.; Wang, D.; Wang, B.; Mei, Z. H.; Liu, J.; Yu, H. W., Enhanced activity of *Rhizomucor miehei* lipase by directed evolution with simultaneous evolution of the propeptide. *Appl. Microbiol. Biotechnol.* **2012,** *96*, 443-50.
3. Contente, M. L.; Roura Padrosa, D.; Molinari, F.; Paradisi, F., A strategic Ser/Cys exchange in the catalytic triad unlocks an acyltransferase-mediated synthesis of thioesters and tertiary amides. *Nat. Catal.* **2020,** *3*, 1020-1026.

4. Jost, E.; Kazemi, M.; Mrkonjić, V.; Himo, F.; Winkler, C. K.; Kroutil, W., Variants of the Acyltransferase from *Mycobacterium smegmatis* Enable Enantioselective Acyl Transfer in Water. *ACS Catal.* **2020,** *10*, 10500-10507.
5. Hendil-Forssell, P. Rational engineering of esterases for improved amidase specificity in amide synthesis and hydrolysis. KTH Royal Institute of Technology, Stockholm, 2016.
6. Zadlo-Dobrowolska, A.; Hammerer, L.; Pavkov-Keller, T.; Gruber, K.; Kroutil, W., Rational Engineered C-Acyltransferase Transforms Sterically Demanding Acyl Donors. *ACS Catal.* **2020,** *10*, 1094-1101.
7. Cen, Y.; Singh, W.; Arkin, M.; Moody, T. S.; Huang, M.; Zhou, J.; Wu, Q.; Reetz, M. T., Artificial cysteine-lipases with high activity and altered catalytic mechanism created by laboratory evolution. *Nat. Commun.* **2019,** *10*, 3198.
8. Müller, H.; Godehard, S. P.; Palm, G. J.; Berndt, L.; Badenhorst, C. P. S.; Becker, A. K.; Lammers, M.; Bornscheuer, U. T., Discovery and Design of Family VIII Carboxylesterases as Highly Efficient Acyltransferases. *Angew. Chem., Int. Ed.* **2021,** *60*, 2013-2017.
9. Bollinger, A.; Molitor, R.; Thies, S.; Koch, R.; Coscolín, C.; Ferrer, M.; Jaeger, K.-E., Organic-Solvent-Tolerant Carboxylic Ester Hydrolases for Organic Synthesis. *Appl. Environ. Microbiol.* **2020,** *86*, e00106-20.
10. Müller, H.; Becker, A. K.; Palm, G. J.; Berndt, L.; Badenhorst, C. P. S.; Godehard, S. P.; Reisky, L.; Lammers, M.; Bornscheuer, U. T., Sequence-Based Prediction of Promiscuous Acyltransferase Activity in Hydrolases. *Angew. Chem., Int. Ed.* **2020,** *59*, 11607-11612.
11. Assaf, Z.; Eger, E.; Vitnik, Z.; Fabian, W. M. F.; Ribitsch, D.; Guebitz, G. M.; Faber, K.; Hall, M., Identification and Application of Enantiocomplementary Lactamases for Vince Lactam Derivatives. *ChemCatChem* **2014,** *6*, 2517-2521.
12. Evans, P., Scaling and assessment of data quality. *Acta Crystallogr. D: Struct. Biol.* **2006,** *62*, 72-82.
13. Evans, P. R., An introduction to data reduction: space-group determination, scaling and intensity statistics. *Acta Crystallogr. D Biol. Crystallogr.* **2011,** *67*, 282-292.
14. Krissinel, E.; Uski, V.; Lebedev, A.; Winn, M.; Ballard, C., Distributed computing for macromolecular crystallography. *Acta Crystallogr. D: Struct. Biol.* **2018,** *74*, 143-151.
15. Winn, M. D.; Ballard, C. C.; Cowtan, K. D.; Dodson, E. J.; Emsley, P.; Evans, P. R.; Keegan, R. M.; Krissinel, E. B.; Leslie, A. G. W.; McCoy, A.; McNicholas, S. J.; Murshudov, G. N.; Pannu, N. S.; Potterton, E. A.; Powell, H. R.; Read, R. J.; Vagin, A.; Wilson, K. S., Overview of the CCP4 suite and current developments. *Acta Crystallogr. D: Struct. Biol.* **2011,** *67*, 235-242.
16. Mccoy, A. J.; Grosse-Kunstleve, R. W.; Adams, P. D.; Winn, M. D.; Storoni, L. C.; Read, R. J., Phaser crystallographic software. *J. Appl. Crystallogr.* **2007,** *40*, 658-674.
17. Murshudov, G. N.; Skubak, P.; Lebedev, A. A.; Pannu, N. S.; Steiner, R. A.; Nicholls, R. A.; Winn, M. D.; Long, F.; Vagin, A. A., REFMAC5 for the refinement of macromolecular crystal structures. *Acta Crystallogr. D: Struct. Biol.* **2011,** *67*, 355-367.
18. Emsley, P.; Lohkamp, B.; Scott, W. G.; Cowtan, K., Features and development of Coot. *Acta Crystallogr. D Biol. Crystallogr.* **2010,** *66*, 486-501.
19. Pitzer, J.; Steiner, K.; Schmid, C.; Schein, V. K.; Prause, C.; Kniely, C.; Reif, M.; Geier, M.; Pietrich, E.; Reiter, T.; Selig, P.; Stückler, C.; Pöchlauer, P.; Steinkellner, G.; Gruber, K.; Schwab, H.; Glieder, A.; Kroutil, W., Racemization-free and scalable amidation of l-proline in organic media using ammonia and a biocatalyst only. *Green Chem.* **2022,** *24*, 5171-5180.
